# Prediction of B-cell epitopes using evolutionary information and propensity scales

**DOI:** 10.1186/1471-2105-14-S2-S10

**Published:** 2013-01-21

**Authors:** Scott Yi-Heng Lin, Cheng-Wei Cheng, Emily Chia-Yu Su

**Affiliations:** 1 School of Medicine, College of Medicine, Taipei Medical University, Taipei, Taiwan; 2 Institute of Information Sciences, Academia Sinica, Taipei, Taiwan; 3 Graduate Institute of Biomedical Informatics, Taipei Medical University, Taipei, Taiwan

## Abstract

**Background:**

Development of computational tools that can accurately predict presence and location of B-cell epitopes on pathogenic proteins has a valuable application to the field of vaccinology. Because of the highly variable yet enigmatic nature of B-cell epitopes, their prediction presents a great challenge to computational immunologists.

**Methods:**

We propose a method, BEEPro (B-cell epitope prediction by evolutionary information and propensity scales), which adapts a linear averaging scheme on 16 properties using a support vector machine model to predict both linear and conformational B-cell epitopes. These 16 properties include position specific scoring matrix (PSSM), an amino acid ratio scale, and a set of 14 physicochemical scales obtained via a feature selection process. Finally, a three-way data split procedure is used during the validation process to prevent over-estimation of prediction performance and avoid bias in our experiment results.

**Results:**

In our experiment, first we use a non-redundant linear B-cell epitope dataset curated by Sollner *et al. *for feature selection and parameter optimization. Evaluated by a three-way data split procedure, BEEPro achieves significant improvement with the area under the receiver operating curve (AUC) = 0.9987, accuracy = 99.29%, mathew's correlation coefficient (MCC) = 0.9281, sensitivity = 0.9604, specificity = 0.9946, positive predictive value (PPV) = 0.9042 for the Sollner dataset. In addition, the same parameters are used to evaluate performance on other independent linear B-cell epitope test datasets, BEEPro attains an AUC which ranges from 0.9874 to 0.9950 and an accuracy which ranges from 93.73% to 97.31%. Moreover, five-fold cross-validation on one benchmark conformational B-cell epitope dataset yields an accuracy of 92.14% and AUC of 0.9066.

**Conclusions:**

Compared with other current models, our method achieves a significant improvement with respect to AUC, accuracy, MCC, sensitivity, specificity, and PPV. Thus, we have shown that an appropriate combination of evolutionary information and propensity scales with a support vector machine model can significantly enhance the prediction performance of both linear and conformational B-cell epitopes.

## Background

### Introduction

The idea of using peptide-based vaccines to replace live or attenuated whole-pathogen vaccines has been an emerging field, as peptide-based vaccines can offer greater safety, potency, and elegance in drug design and delivery [1]. The development of these peptide-based vaccines requires first of all the identification of highly immunogenic regions within a given pathogen protein. These immunogenic regions, or more particularly the B-cell epitopes, are responsible for eliciting humoral immune response by inducing production of neutralizing antibodies.

Two types of B-cell epitopes have been defined: linear (continuous) and conformational (discontinuous). While the majority (~90%) of the B-cell epitopes is conformational, the difficulties in the design of such conformational epitopes have led to an emphasis on classification of linear B-cell epitopes [1].

As development of vaccines is critical in our protection against infectious diseases, effective screening methods to identify immunogenic epitopes from the pathogenic proteome will be necessary. Classical methods such as phage display system have successfully yielded peptides that have proceeded to clinical trials, yet these experimental techniques are labour-intensive and may not reflect *in vivo *binding conditions or the biological ability to stimulate antibody production [2,3]. The shortcomings of current experimental methods call for the development of new computational models that can more effectively predict the presence and location of immunogenic (protective) epitopes given a pathogenic protein sequence.

### Previous works

Sollner *et al. *have studied the use of conservation in post-translational modification and sequence variability to predict protective linear B-cell epitopes, i.e., linear B-cell epitopes associated with biological activity. Their results showed that focusing on conserved region and regions lacking post-translational modification sites may be beneficial [2]. Training the same dataset using a Naïve Bayes classifier, El-Manzalawy *et al. *also showed that position specific scoring matrix (PSSM) offered the best performance when compared with selected physicochemical scales and dipeptide composition representation [3]. Together, these two studies demonstrated that protective linear epitopes may have sequence conservations that explain their functional role, and that focusing on protective linear epitopes may improve the performance of current prediction models for linear B-cell epitopes [1].

In addition, Blythe and Flower have previously shown that simple propensity scale-based methods are only marginally better than random prediction [4]. Thus, researches since then have suggested the use of a combination of more than one propensity scale with a machine-learning algorithm to improve the prediction performance [5-7].

### Challenges and our contributions

As the current lack of success in B-cell prediction could be explained by inadequate or incomplete selection of appropriate propensity scales to reflect the complex patterns of B-cell epitopes, our study attempts to first reconfirm the belief that combinational approach outperforms single propensity scale approach. Then, twenty properties, including amino acid ratio scale, PSSM, and 18 physicochemical scales selected from AAIndex, are used to construct a hybrid propensity scale model by training a support vector machine (SVM) classifier on a protective linear B-cell epitope dataset [2]. Parameter optimization and feature selection are then applied to yield an optimal set of propensity scales with the best performance. We propose the final optimized model, BEEPro (B-cell Epitope prediction using Evolutionary information and Propensity scales). Six B-cell epitope datasets are used to evaluate the performance of BEEPro, with one of the datasets consisting of conformational B-cell epitopes.

## Methods

### Datasets

In this study, we applied seven datasets used in previous studies to allow unbiased validation of our method and to compare the performance of our model with others. Table 1 summarizes these datasets, which are detailed below and available in the supplementary material [Additional files 1, 2, 3, 4, 5, 6, 7].

**Table 1:** Summary of B-cell epitope datasets used in this study.

Dataset	Sollner	AntiJen#1	AntiJen#2	HIV	Pellequer	PC	Benchmark
**Number of proteins**	57	124	171	10	14	12	52
**Epitope residue**	2,317	5,529	11,249	1,018	858	1,852	858
**Non-epitope residue**	43,690	60,800	75,805	1,693	1,695	3,509	9,527
**Number of residues**	46,007	66,329	87,054	2,711	2,553	5,361	10,385
**Epitope density**	5.04%	8.34%	12.92%	37.55%	33.61%	34.55%	8.26%

#### Sollner dataset

This dataset was curated by Sollner *et al. *and contains 57 non-redundant pathogen proteins extracted from IEDB database [2]. Each antigen is annotated with a number of linear B-cell epitopes that are classified as "leading to biological activity." This is the first dataset that closely approximates protective linear B-cell epitopes [3]. The dataset is comprised of 2,317 residues labeled as part of an epitope (5.04%) and 43,690 non-epitope residues (94.96%). To evaluate the performance of B-cell epitope prediction, this non-redundant dataset is used for feature selection and parameter optimization based on a three-way data split procedure.

#### AntiJen #1 and #2 datasets

These two datasets were extracted from the AntiJen database [8]. AntiJen#1 is provided by Larsen *et al. *and contains 124 protein sequences (5,529 epitope residues, 8.34%; 60,800 non-epitope residues, 91.66%) [7]. AntiJen#2 is provided by Wang *et al. *and contains 171 protein sequences with 691 non-overlapping epitopes (11,249 epitope residues, 12.92%; 75,805 non-epitope residues, 87.08%) [9].

#### HIV dataset

This dataset was curated from the HIV Molecular Immunology Database of the Los Alamos National Laboratory, http://www.hiv.lanl.gov/[10]. The electronic copy of this dataset is provided by Larsen *et al. *and contains 10 HIV proteins (1,018 epitope residues, 37.55%; 1,693 non-epitope residues, 62.45%) [7].

#### Pellequer dataset

This dataset was first presented by Pellequer *et al. *[11], and the electronic version of this dataset was recreated by Lund *et al. *[7]. This dataset contains 14 proteins and 83 epitopes (858 epitope residues, 33.61%; 1,695 non-epitope residues, 66.39%).

#### PC dataset

This dataset was curated by Wang *et al. *and contains 12 protein sequences with 98 non-overlapping epitopes (1,852 epitope residues, 34.55%; 3,509 non-epitope residues, 65.45%) [9]. The epitopes in this dataset were experimentally identified with peptide scan methodology [9].

#### Benchmark dataset

The original benchmark dataset contains 161 protein chains from 144 antigen-antibody complex structures [12]. Ansari and Raghava applied CD-HIT at 40% cutoff value to yield a non-redundant dataset of 52 antigen chains (858 epitope residues, 8.26%; 9,527 non-epitope residues, 91.74%) [13,14]. Epitope residues in this dataset are defined as antigen residues where at least one of the atoms is distanced within 4Å from any antibody atom based on PDB structures [13].

### Calculation of amino acid ratio propensity scale

The amino acid ratio (AAR) propensity scale for each of the 20 types of amino acid *α_i _*is computed according to the following equation, where $f\left(\alpha_{i}^{+}\right)$ and $f\left(\alpha_{i}^{-}\right)$ represent the occurrence frequencies of amino acid *α_i _*in epitope and non-epitope peptide sequences, respectively.

$$p\left(\alpha_{i}\right)=\frac{f\left(\alpha_{i}^{+}\right)/\sum_{i}f\left(\alpha_{i}^{+}\right)}{f\left(\alpha_{i}^{-}\right)/\sum_{i}f\left(\alpha_{i}^{-}\right)}$$

Previous studies have used a similar equation to compute an amino acid dimer (or amino acid pairs, AAP) propensity scale [9,15]. In those studies, logarithm was taken of the AAP ratios before the normalization step. We do not, however, find significant changes in performance of our hybrid propensity scale model when logarithm is used. Considering that $f\left(\alpha_{i}^{+}\right)$ could become zero after data-split (for example, the Benchmark dataset contains only one cysteine epitope residue for the entire dataset), the logarithm step is neglected in this study.

To avoid dominance of any individual *ρ*(*α_i_*) values, the following equation is used to normalize the values to the range of [-1, 1].

$$\rho \left(\alpha_{i}\right)=2\left(\frac{p\left(\alpha_{i}\right)-\min\left\{p\left(\alpha_{i}\right)\right\}}{\max\left\{p\left(\alpha_{i}\right)\right\}-\min\left\{p\left(\alpha_{i}\right)\right\}}\right)-1$$

To avoid bias in our results, for all the methods below which involve amino acid ratio propensity scale, the scale values are re-calculated for each fold of cross-validation and three-way data split using only the data of training set.

### Generation of position specific scoring matrix

PSSM is used to reflect the evolutionary information of a peptide. Blast-2.2.26+ package are downloaded from ftp://ftp.ncbi.nih.gov/blast/, and the psiblast program of this package is used to generate multiple sequence alignment against non-redundant (*nr*) protein database, which is downloaded from ftp://ftp.ncbi.nih.gov/blast/db/. The *nr *database uploaded on July 9, 2012 is used for this study. PSSM is generated using the setting: e-value = 0.001, number of iterations = 3.

### Single propensity scale method

Each peptide of running window size *w *for a residue at position *i *is represented by a vector of size *w*: [*x_i-(w-1)/2_, ..., x_i_, ..., x_i+(w-1)/2_*], where *x_i _*is the propensity scale value at residue position *i*. *w *in this study ranges from 5 to 29 in steps of 2. For the residues at the N- or C-terminus of the peptide sequence, appropriate number of zeros is appended to the head or the tail of the vector, respectively, to make up a vector of the right size. In addition to the amino acid ratio propensity scale, four more representative physicochemical scales are also used for comparison: Parker's hydrophilicity [16], Karplus' flexibility [17], Grantham's polarity [18], and Janin's accessible area [19]. These scales have been used by previous studies for B-cell epitope prediction [3,5,7,13].

In the case of PSSM, each peptide of running window size *w *is represented by a vector of size [20 × *w*] because the amino acid at each residue position is represented by an evolutionary information vector of 20 log-likelihood values. As in the case of single propensity scale method, zeros are appended at either the head or the tail of the vector for residues at the N- or the C-terminus of the peptide, respectively, to account for the asymmetry at the two ends of peptide.

### Hybrid propensity scale method

Other than the amino acid ratio propensity scale and PSSM, additional 18 physicochemical scales selected from AAIndex [20] are considered for incorporation into a hybrid propensity scale model. These 18 scales include antigenicity [21], hydrophilicity [16,22], hydrophobicity [23], accessible surface area [19], flexibility [17,24], interactivity [25], buriability [26], composition [18], polarity [18], volume [18], charge transfer and donor capability [27], hydrogen-bond donor capability [28], secondary structure (i.e., alpha helix, beta structure, and coil) [29]. Initially, all 20 features are used. Feature selection is then applied to determine the most suitable combination of features that yields optimal performance.

For each residue *α_i _*at position *i*, a peptide of running window size *w *is represented by a vector of size equal to the number of features (*n*) used: [*avg_scale_1_, avg_scale_2_, ..., avg_scale_n_*]. *w *in this study ranges from 5 to 29 in steps of 2.

The average for each propensity scale is calculated by the following formula, where *i *is the position index of a residue in the running peptide window, *c *is the central residue position index in the peptide window, *|c - i| *is the distance in residue number between residue *i *and the central residue, *f *is the linear weighting factor (in this study, *f *= 0.00, 0.02, 0.04, 0.06, 0.08, 0.10), *s_i _*is the propensity scale value of the residue at position *i*. For the property of PSSM, *s_i _*is the sum of the 20 PSSM log-likelihood values of residue *i*.

$$avg_{scale}=\frac{\sum_{i}\left(1-f\left|c-i\right|\right)s_{i}}{w}$$

### Training and classification

SVM is a machine learning algorithm proposed by Vapnik based on structural risk minimization principle of statistics learning theory [30]. It can be used to solve classification and regression problems. As determining whether a residue in a protein sequence belongs to an epitope is a binary classification problem, SVM would be useful for our purpose. In this study, LIBSVM, a well-known and powerful SVM package developed by Chang and Lin, is used to decipher epitope residues from non-epitope residues [31]. In the process of model development, we use radial basis function (RBF) as the kernel function in SVM. The formulation of RBF is defined in the following equation, where *x_i _*and *x_j _*are two data vectors, and *γ *is a training parameter.

$$RBF\left(x_{i},x_{j}\right)=\exp\left(-\gamma {∥x_{i}-x_{j}∥}^{2}\right)$$

Each running peptide window is labeled as either +1 if the central residue of the window belongs to an epitope or -1 if the central residue is not part of any epitope. The profile generated either by single or hybrid propensity scale method is first scaled to the range of [-1, 1] using built-in scaling program of LIBSVM.

For single propensity scale method, five-fold cross-validation is applied to the Sollner dataset with the following parameters: -*w_+1 _*= 20, -*w_-1 _*= 1, -*c *= default, -*g *= default. The procedure is iterated five times.

For hybrid propensity scale method, we use a more stringent three-way data split procedure to train and evaluate performance of BEEPro. We first divide the Sollner dataset into five distinct, non-overlapping sets: three for training (classifier learning), one for validation (parameter optimization and feature selection), and one for testing (performance evaluation). The procedure is iterated five times. The steps of model optimization include (in this order): (1) selection of optimal running window size, (2) optimization of -*c*, -*g*, (3) optimization of -*w_+1_*, (4) feature selection, (5) optimization of linear weighting factor *f*, (6) re-optimization of -*w_+1_*.

To further evaluate the performance of BEEPro, we perform five-fold cross-validation on each of the other datasets using exactly the same optimized setting obtained during training and validation with the Sollner dataset.

### Performance evaluation

The area under the receiver operating curve (AUC) is used to assess performance during parameter selection. AUC is one of the most appropriate measures of performance as it is non-parametric and threshold independent. It is also independent of the number of positive and negative test cases. In addition, AUC is the recommended metric at a workshop organized by the National Institute of Allergy and Infectious Disease in 2006 [32]. AUC ranges from 0.5 to 1, with 0.5 being random predictor, and 1 being perfect predictor.

Other metrics are also computed to allow more comprehensive comparison against previous studies. Sensitivity (SEN) and specificity (SPE) measure how well the classifier detects epitopes as epitopes and non-epitopes as non-epitopes, respectively. Matthew's correlation coefficient (MCC) takes under-prediction and over-prediction into consideration and is useful even when positive and negative test cases differ in size. Accuracy (ACC) is the proportion of correctly predicted residue. Positive predictive value (PPV) is the percentage of detected epitope residues that truly belong to an epitope. The following equations define these statistics, where TP, TN, FP, and FN denote the number of true positives, true negatives, false positives, and false negatives, respectively.

$$SEN=\frac{TP}{TP+FN}$$

$$SPE=\frac{TN}{TN+FP}$$

$$MCC=\frac{TP\times TN-FP\times FN}{\sqrt{\left(TP+FP\right)\left(TP+FN\right)\left(TN+FP\right)\left(TN+FN\right)}}$$

$$ACC=\frac{TP+TN}{TP+TN+FP+FN}$$

$$PPV=\frac{TP}{TP+FP}$$

## Results

### Prediction based on single propensity scale or position specific scoring matrix

**Figure 1:**
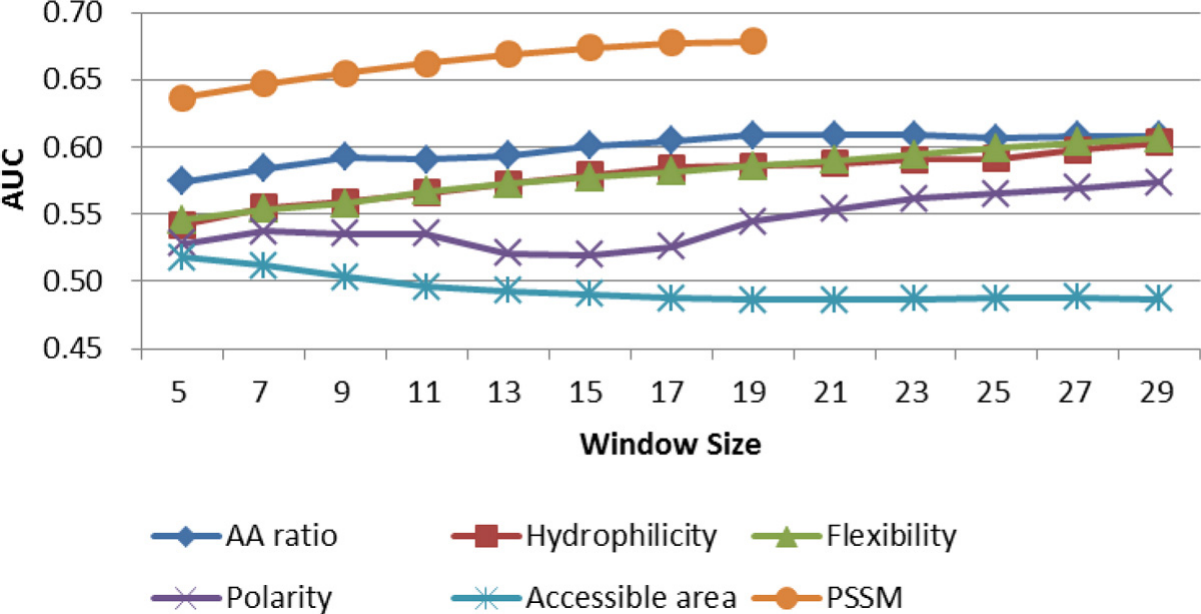
**AUC for single propensity scale methods across different window sizes**.

In general, the prediction performance of single propensity scale methods improves as the size of window increases, with the exception of the accessible surface area scale, which decreases as window size increases, and the polarity scale, which fluctuates across different window sizes (Figure 1, Additional File 8: Supplementary Table 1). Among the four physicochemical propensity scales, Parker's hydrophilicity (AUC = 0.5855 at *w *= 19) and Karplus' flexibility (AUC = 0.5859) scales have insignificant difference in performance, and both outperform Grantham's polarity (AUC = 0.5442) and Janin's accessible surface area scale (AUC = 0.4863). Accessible surface area has the worst performance of the four scales, with AUC value roughly being 0.49. The amino acid ratio propensity scale (AUC = 0.6090) outperforms the four physicochemical scales regardless of window size, and this gives us confidence to use this scale for the later hybrid model. PSSM outperforms amino acid ratio propensity scale and each of the four physicochemical properties, with AUC of 0.6786 at *w *= 19.

### Hybrid propensity scale method significantly improves prediction performance

**Figure 2:**
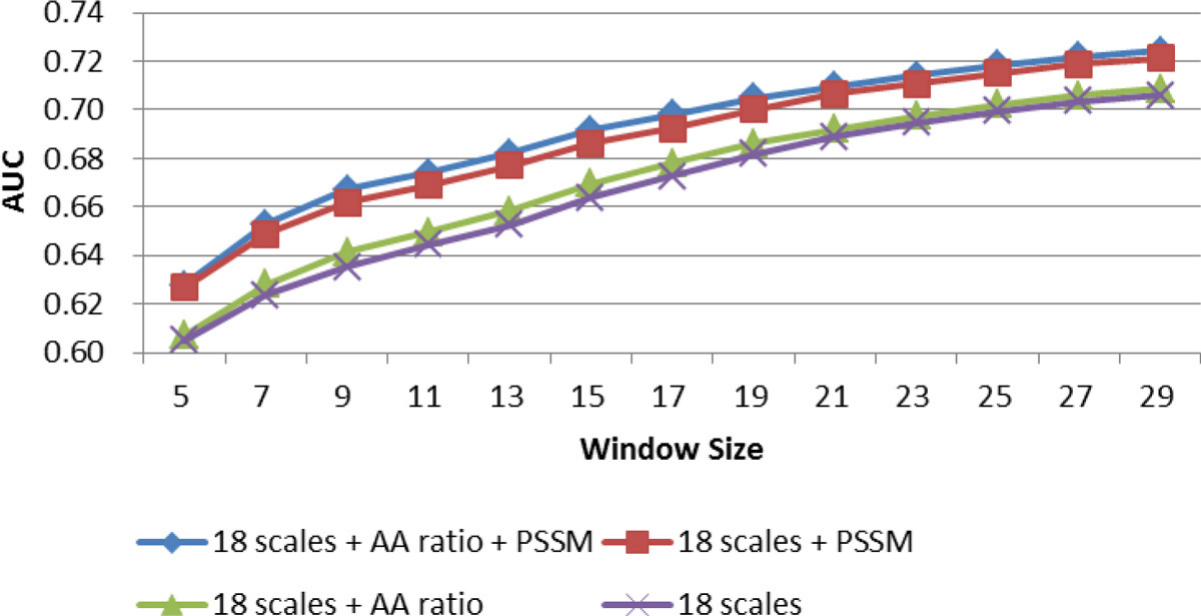
**AUC for hybrid propensity scale methods across different window sizes**.

There is a significant improvement when PSSM is incorporated into the hybrid propensity scale model and a slight improvement when amino acid ratio propensity scale is incorporated (Figure 2, Additional File 8: Supplementary Table 2). We also see here that the hybrid propensity scale method (AUC = 0.7049) offers significant improvement over the single scale methods (AUC = 6786 for PSSM). As with single propensity scale, the performance of the model improves as *w *increases. We choose *w *= 19 for further development as this is consistent with most literatures to date and would therefore allow more direct comparison between methods.

Feature selection allows us to determine the optimal parameter combination: -*w_+1 _*= 15, -*w_-1 _*= 1, -*c *= 1, -*g *= 10, linear weighting factor = 0.08. Finally, several features should be removed from feature list, including: Janin's accessible surface area, Grantham polarity, Bastolla's interactivity, and Zhou's buriability (Additional File: Supplementary Table 3). After parameter selection with the three-way data split approach, we arrived at the final performance AUC = 0.9987, ACC = 0.9929, SEN = 0.9604, SPE = 0.9946, MCC = 0.9281, PPV = 0.9042 for the Sollner dataset (Table 2).

**Table 2:** Performance comparison of different methods using five-fold cross-validation for different datasets.

Dataset	Method	AUC	ACC	SEN	SPE	*MCC*	PPV
**Sollner**	**BEEPro**	**0.9987**	**0.9929**	**0.9604**	**0.9946**	**0.9281**	**0.9042**

**AntiJen#1**	**BEEPro**	**0.9930**	**0.9731**	**0.9680**	**0.9735**	**0.8491**	**0.7688**

**AntiJen#2**	**BEEPro**	**0.9907**	**0.9580**	**0.9700**	**0.9562**	**0.8402**	**0.7668**
	**LEPS**	NA	0.7381	0.2672	0.8448	0.1010	0.2885
	**BepiPred**	NA	0.5552	0.5179	0.5761	0.0604	0.2202
	**ABCPred_0.8_**	NA	0.4470	0.6733	0.4040	0.0546	0.2183
	**BCPred**	NA	0.5392	0.5884	0.5487	0.0893	0.2334
	**FBCPred**	NA	0.5145	0.6031	0.5121	0.0673	0.2233

**HIV**	**BEEPro**	**0.9907**	**0.9454**	**0.9490**	**0.9433**	**0.8853**	**0.9098**
	**LEPS**	NA	0.6345	0.4833	0.7484	0.2276	0.7144
	**BepiPred**	0.6000	0.5672	0.5016	0.6085	0.0972	0.6122
	**ABCPred_0.7_**	NA	0.5659	0.8797	0.1465	0.0564	0.5633
	**BCPred**	NA	0.6657	0.8018	0.5457	0.2980	0.6555
	**FBCPred**	NA	0.6713	0.7320	0.5820	0.2781	0.6556

**Pellequer**	**BEEPro**	**0.9874**	**0.9373**	**0.9256**	**0.9435**	**0.8621**	**0.8935**
	**BepiPred**	0.6710	NA	NA	NA	NA	NA

**PC**	**BEEPro**	**0.9950**	**0.9550**	**0.9708**	**0.9468**	**0.9036**	**0.9058**
	**LEPS**	NA	0.6166	0.1278	0.8833	0.0365	0.4512
	**BepiPred**	NA	0.5533	0.4823	0.5972	0.0749	0.3819
	**ABCPred_0.8_**	NA	0.4889	0.6546	0.4026	0.0513	0.3621
	**BCPred**	NA	0.5283	0.5092	0.5935	0.0443	0.3607
	**FBCPred**	NA	0.5220	0.5103	0.5255	0.0317	0.3526

**Benchmark**	**BEEPro**	**0.9100**	**0.9200**	0.7100	**0.9400**	**0.5700**	**0.5200**
	**CBTOPE**	0.8900	0.8400	**0.8000**	0.8500	NA	0.3100
	**DiscoTope**	0.6000	0.7500	0.4200	0.7900	NA	0.1600
	**CEP**	0.5400	0.7400	0.3100	0.7800	NA	0.1100
	**ClusPro(DOT) best model**	0.6900	0.8900	0.4500	0.9300	NA	0.3900
	**Patch Dock best model**	0.6600	0.8500	0.4300	0.8900	NA	0.2600
	**PSI-PRED best patch**	0.6000	0.8200	0.3300	0.8600	NA	0.1900
	**ProMate**	0.5100	0.8400	0.0900	0.9200	NA	0.1000

^1^LEPS, BepiPred, ABCPred, BCPred, FBCPred performances were previously compiled by Wang *et al. *[9]

^2^CBTOPE, DiscoTope, CEP, ClusPro, Patch Dock, PSI-PRED, ProMate performances were previously compiled by Ansari and Raghava [13]

### Performance comparison with existing methods

Applying exactly the same feature extraction method and the same parameter setting as the optimized BEEPro model, we perform five-fold cross-validation on the independent datasets (Table 2).

For the linear epitope datasets AntiJen #1 and #2, HIV, Pellequer, PC, the AUC is 0.9930, 0.9907, 0.9907, 0.9874, and 0.9950, respectively. The accuracy is 0.9731, 0.9580, 0.9454, 0.9373, and 0.9550, respectively. BEEPro outperforms many other current linear epitope prediction methods, including LEPS [9], BepiPred [7], ABCPred [33], BCPred [15], and FBCPred [34] (Table 2).

Even for the conformational epitope dataset (Benchmark dataset), we are able to obtain AUC = 0.91, ACC = 0.92, SEN = 0.71, SPE = 0.94, MCC = 0.57, PPV = 0.52. BEEPro outperforms other conformational epitope predictors such as DiscoTope [35] and CEP [36] (Table 2). Although PPV value is not as high as for linear epitope datasets, it is still significantly higher than other current models. However, the sensitivity of BEEPro (0.71) for this Benchmark dataset is not as high as CBTOPE (0.80), and this could be explained by BEEPro's significantly higher specificity.

## Discussion

### Hybrid method performs better than single propensity scale method

Blythe and Flower determined in 2005 that the best single propensity scale performs only marginally better than random prediction [4]. Several studies have later demonstrated that the use of a combination of propensity scales for feature representation could have better results than using single propensity scale [5]. Our results agree with this statement, as we see a significant improvement in performance between single propensity scale methods and hybrid models. This reflects the complex nature of epitopes, as more than one property is needed to reflect the epitope peptide profile.

### Evolutionary information is effective for B-cell epitope identification

Evolutionary information, encoded as PSSM generated by PSI-BLAST, has been used to improve prediction performance of other biological prediction problems such as RNA binding sites and protein cellular localization [37-40]. In the field of B-cell epitope prediction, El-Manzalawy *et al. *have shown that PSSM outperforms selected single physicochemical scales, a result similarly observed in this study [3]. We extend this observation and demonstrate in this paper that while PSSM alone may be insufficient, combining PSSM with other propensity scales does improve the hybrid propensity scale model markedly, implicating the significance of evolutionary information and sequence conservation as an important determining factor for a peptide's immunogenic property.

### Effects of physicochemical propensity scales

We have constructed a prediction method for B-cell epitopes using support vector machine. The finalized feature list is based on amino acid ratio propensity scale, PSSM, and 14 physicochemical propensity scales that reflect properties of antigenicity, hydrophilicity, flexibility, composition, volume, charge transfer and donor capability, hydrogen bond donor capability, and secondary structure such as alpha helical structure, beta structure, and coil structure. We have included chemical properties such as charge transfer and donor, and hydrogen bond donor scales in the development of our model, which have not been attempted in previous literatures to the best of our knowledge. It is possible that these chemical properties could be relevant in determining the chemical behaviors between antigens and antibodies. We have also applied for the first time Bastolla's interactivity scale [25] and Zhou's buriability scale [26], but these two scales fail to improve our hybrid propensity scale model.

### Generality of BEEPro on different datasets

Sollner *et al. *curated their linear B-cell epitope dataset from the IEDB database using a series of filtering steps that allow correlation of annotated epitopes with functional relevance. This Sollner dataset is used for training and optimization of BEEPro in our study. Although we have trained our model using a more stringent protective linear B-cell epitope dataset, we have also shown that the same feature representation method and parameter setting can be extended to general linear B-cell epitopes and even to conformational B-cell epitopes with high performance. Compared with other current prediction systems of linear and conformational epitopes, our method has a superior performance in area under curve, accuracy, Matthew's correlation coefficient, positive predictive value, sensitivity, and specificity.

The observation that BEEPro does not perform as well in the Benchmark dataset, which consists of conformational epitopes, is not surprising as the model was originally optimized using a linear epitope dataset. However, BEEPro still outperforms current structure-based prediction methods in classifying conformational epitope positions, and this would echo previous report that sequence-based predictor can work as a complement to current structure-based prediction methods [13]. BEEPro can also be applied when structural information of antibody-antigen complex is not available.

It is promising to note that PPV of BEEPro is about 0.90~0.91 for linear epitopes, and 0.52 for conformational epitopes, as this could imply a more cost-effective approach to screen for possible peptide vaccine candidates. Also, BEEPro has been shown to excel in independent datasets of varying epitope density (from 5.04% to 37.55%), and this would reflect applications in real world where epitopes take up only a small portion of an antigen sequence.

## Conclusions

In this paper, we describe BEEPro, a method to predict B-cell epitopes using evolutionary information, amino acid ratio propensity scale, and 14 physicochemical propensity scales, for a total of 16 features. The 14 physicochemical propensity scales in BEEPro reflect information on antigenicity, hydrophilicity, flexibility, composition, volume, charge transfer and donor capability, hydrogen bond donor capability, and secondary structure such as alpha helical structure, beta structure, and coil structure. The five-fold cross-validation accuracy on six linear B-cell epitope datasets ranges from 93.73% to 99.29%, with AUC ranging from 0.9874 to 0.9987. In addition, the five-fold cross-validation accuracy on one benchmark conformational B-cell epitope dataset is 92.14%, with AUC of 0.9066. We have shown that BEEPro outperformed other sequence-based and structure-based prediction methods.

## Competing interests

The authors declare that they have no competing interests.

## Authors' contributions

SYHL designed and implemented the system, analyzed the data, and drafted the manuscript. CWC provided computational assistance. ECYS supervised the project and revised the manuscript. All the authors read and approved the final manuscript.

## Declarations

The publication costs for this article were funded by National Science Council under grant NSC101-2221-E-038-014.

This article has been published as part of *BMC Bioinformatics *Volume 14 Supplement 2, 2013: Selected articles from the Eleventh Asia Pacific Bioinformatics Conference (APBC 2013): Bioinformatics. The full contents of the supplement are available online at http://www.biomedcentral.com/bmcbioinformatics/supplements/14/S2.

## Supplementary Material

Additional file 1 **The Sollner dataset**. Click here for file

Additional file 2 **The AntiJen#1 dataset**. Click here for file

Additional file 3 **The AntiJen#2 dataset**. Click here for file

Additional file 4 **The HIV dataset**. Click here for file

Additional file 5 **The Pellequer dataset**. Click here for file

Additional file 6 **The PC dataset**. Click here for file

Additional file 7 **The Benchmark dataset**. Click here for file

Additional file 8 **Supplementary tables on optimization of hybrid propensity scale method**. Click here for file
