# Supplementary material for: Prediction of B-cell epitopes using evolutionary information and propensity scales
Source: BMC Bioinformatics. 2013 Jan 21;14(Suppl 2):S10. doi: 10.1186/1471-2105-14-S2-S10 (PMC3549808; doi:10.1186/1471-2105-14-S2-S10)
Supplement: Additional file 1 — The Sollner dataset. [file 1471-2105-14-S2-S10-S1.pdf]

Additional file 1. The Sollner dataset.

>14162008 phosphoprotein [rabies virus]. [mapped in Rabies virus (strain Ontario fox)]
MSKIFVNPSAIRAGLADLEMAEETVDLINKNIEDNQAHLQGEPIEVDNLPEDMKRLQLDDKKPSGL
GEMARAGEGKCREDFLMDEGEDPSLLFQSYLDNVGVQIVRQMRSGERFLKIWSQTVEEIISYVTVN
FPNPPGKSLEDKSTQTTGRELKKETTSISSQRDSQSSKARMVAQAASGPPALEWSATNEEDDLSVE
AEIAHQVAESFSKKYKFPSRSSGIFLYNFEQLKMNLDDIVKEAKNVPGVTRLAHDGSKLPLRCVLG
WVGLANSKKFQLLVEPDKLNKIMQDDLNRYTSS
000000000000000000000000000000000000000000000000000000000000000000
000000000000000000000000000000000000000000000000000000000000000000
000000000000000000000111111111111111111000000000000000000000000000
000000000000000000000000000000000000000000000000000000000000000000
000000000000000000000000000000000
>O89343 |HN_HENDV Hemagglutinin-neuraminidase 29468607 glycoprotein [Hendra virus]. [mapped in Hendra virus]
MMADSKLVSLNNNLSGKIKDQGKVIKNYYGTMDIKKINDGLLDSKILGAFNTVIALLGSIIIIVMN
IMIIQNYTRTTDNQALIKESLQSVQQQIKALTDKIGTEIGPKVSLIDTSSTITIPANIGLLGSKIS
QSTSSINENVNDKCKFTLPPLKIHECNISCPNPLPFREYRPISQGVSDLVGLPNQICLQKTTSTIL
KPRLISYTLPINTREGVCITDPLLAVDNGFFAYSHLEKIGSCTRGIAKQRIIGVGEVLDRGDKVPS
MFMTNVWTPPNPSTIHHCSSTYHEDFYYTLCAVSHVGDPILNSTSWTESLSLIRLAVRPKSDSGDY
NQKYIAITKVERGKYDKVMPYGPSGIKQGDTLYFPAVGFLPRTEFQYNDSNCPIIHCKYSKAENCR
LSMGVNSKSHYILRSGLLKYNLSLGGDIILQFIEIADNRLTIGSPSKIYNSLGQPVFYQASYSWDT
MIKLGDVDTVDPLRVQWRNNSVISRPGQSQCPRFNVCPEVCWEGTYNDAFLIDRLNWVSAGVYLNS
NQTAENPVFAVFKDNEILYQVPLAEDDTNAQKTITDCFLLENVIWCISLVEIYDTGDSVIRPKLFA
VKIPAQCSES
000000000000000000000000000000000000000000000000000000000000000000
000000000000000000000000000000000000000000000000000000000000000000
000000000000000000000000000000000000000000000000000000000011111000
000000000000000000000000000000000000000000000000000000000000000000
000000000000000000000000000000000000000000000000000000000000000000
000000000000000000000000000000000000000000000000000000000000000000
000000000000000000000000000000000000000000000000000000000000000000
000000000000000000000000000000000000000000000000000000000000000000
000000000000000000000000000000000000000000000000000000000000000000
0000000000
>P33478 Genome polyprotein [Contains: Capsid protein C (Core protein); Envelope protein M (Matrix protein); Major envelope protein E;

Non-structural protein 1 (NS1); Non-structural protein 2A (NS2A); Flavivirin protease NS2B regulatory subunit; Flavivirin protease NS3 catalytic subunit ; Non-structural protein 4A (NS4A); Non-structural protein 4B (NS4B); RNA-directed RNA polymerase (NS5)]. [mapped in Dengue virus type 1]

MNNQRKKTARPSFNMLKRARNRVSTGSQLAKRFSKGLLSGQGPMKLVMAFIAFLRFLAIPPTAGIL
ARWGSFKKNGAIKVLRGFKKEISNMLNIMNRRKRSVTMLLMLLPTALAFHLTTRGGEPHMIVSKQE
REKSLLFKTSVGVNMCTLIAMDLGELCEDTMTYKCPRITEAEPDDVDCWCNATDTWVTYGTCSQTG
EHRRDKRSVALAPHVGLGLETRTETWMSSEGAWKQIQRVETWALRHPGFTVIALFLAHAIGTSITQ
KGIIFILLMLVTPSMAMRCVGIGSRDFVEGLSGATWVDVVLEHGSCVTTMAKDKPTLDIELLKTEV
TNPAVLRKLCIEAKISNTTTDSRCPTQGEATLVEEQDANFVCRRTFVDRGWGNGCGLFGKGSLLTC
AKFKCVTKLEGKIVQYENLKYSVIVTVHTGDQHQVGNETTEHGTIATITPQAPTSEIQLTDYGALT
LDCSPRTGLDFNEMVLLTMKEKSWLVHKQWFLDLPLPWTSGASTSQETWNRQDLLVTFKTAHAKKQ
EVVVLGSQEGAMHTALTGATEIQTSGTTTIFAGHLKCRLKMDKLTLKGMSYVMCTGSFKLEKEVAE
TQHGTVLVQVKYEGTDAPCKIPFSTQDEKGVTQNRLITANPIVTDKEKPVNIETEPPFGESYIVVG
AGEKALKQCWFKKGSSIGKMFEATARGARRMAILGDTAWDFGSIGGVFTSVGKLVHQVFGTAYGVL
FSGVSWTMKIGIGILLTWLGLNSRSTSLSMTCIAVGMVTLYLGVMVQADSGCVINWKGRELKCGSG
IFVTNEVHTWTEQYKFQADSPKRLSAAIGKAWEEGVCGIRSATRLENIMWKQISNELNHILLENDM
KFTVVVGDVVGILAQGKKMIRPQPMEHKYSWKSWGKAKIIGADIQNTTFIIDGPDTPECPDDQRAW
NIWEVEDYGFGIFTTNIWLKLRDSYTQMCDHRLMSAAIKDSKAVHADMGYWIESEKNETWKLARAS
FIEVKTCVWPKSHTLWSNGVLESEMIIPKIYGGPISQHNYRPGYFTQTAGPWHLGKLELDFDLCEG
TTVVVDEHCGNRGPSLRTTTVTGKIIHEWCCRSCTLPPLRFKGEDGCWYGMEIRPVKEKEENLVKS
MVSAGSGEVDSFSLGLLCISIMIEEVMRSRWSRKMLMTGTLAVFLLLIMGQLTWNDLIRLCIMVGA
NASDRMGMGTTYLALMATFKMRPMFAVGLLFRRLTSREVLLLTIGLSLVASVELPNSLEELGDGLA
MGIMILKLLTDFQSHQLWATLLSLTFVKTTFSLHYAWKTMAMVLSIVSLFPLCLSTTSQKTTWLPV
LLGSLGCKPLTMFLIAENKIWGRKSWPLNEGIMAVGIVSILLSSLLKNDVPLAGPLIAGGMLIACY
VISGSSADLSLEKAAEVSWEEEAEHSGASHNILVEVQDDGTMKIKDEERDDTLTILLKATLLAVSG
VYPLSIPATLFVWYFWQKKKQRSGVLWDTPSPPEVERAVLDDGIYRIMQRGLLGRSQVGVGVFQDG
VFHTMWHVTRGAVLMYQGKRLEPSWASVKKDLISYGGGWRFQGSWNTGEEVQVIAVEPGKNPKNVQ
TAPGTFKTPEGEVGAIALDFKPGTSGSPIVNREGKIVGLYGNGVVTTSGTYVSAIAQAKASQEGPL
PEIEDEVFRKRNLTIMDLHPGSGKTRRYLPAIVREAIRRNVRTLILAPTRVVASEMAEALKGMPIR
YQTTAVKSEHTGKEIVDLMCHATFTMRLLSPVRVPNYNMIIMDEAHFTDPASIARRGYISTRVGMG
EAAAIFMTATPPGSVEAFPQSNAVIQDEERDIPERSWNSGYEWITDFPGKTVWFVPSIKSGNDIAN
CLRKNGKRVIQLSRKTFDTEYQKTKNNDWDYVVTTDISEMGANFRADRVIDPRRCLKPVILKDGPE
RVILAGPMPVTVASAAQRRGRIGRNQNKEGDQYVYMGQPLNNDEDHAHWTEAKMLLDNINTPEGII
PALFEPEREKSAAIDGEYRLRGEARKTFVELMRRGDLPVWLSYKVASEGFQYSDRRWCFDGERNNQ
VLEENMDVEMWTKEGERKKLRPRWLDARTYSDPLALREFKEFAAGRRSVSGDLILEIGKLPQHLTQ
RAQNALDNLVMLHNSEQGGRAYRHAMEELPDTIETLMLLALIAVLTGGVTLFFLSGKGLGKTSIGL

LCVMASSVLLWMASVEPHWIAASIILEFFLMVLLIPEPDRQRTPQDNQLAYVVIGLLFMILTVAAN
EMGLLETTKKDLGIGHVAAENHHHATMLDVDLRPASAWTLYAVATTVITPMMRHTIENTTANISLT
AIANQAAILMGLDKGWPISKMDIGVPLLALGCYSQVNPLTLTAAVLMLVAHYAIIGPGLQAKATRE
AQKRTAAGIMKNPTVDGIVAIDLDPVVYDAKFEKQLGQIMLLILCTSQILLMRTTWALCESITLAT
GPLTTLWEGSPGKFWNTTIAVSMANIFRGSYLAGAGLAFSLMKSLGGGRRGTGAKGKHWERNGKDR
LNQLSKSEFNTYKRSGIMEVDRSEAKEGLKRGETTKHAVSRGTAKLRWFVERNLVKPEGKVIDLGC
GRGGWSYYCAGLKKVTEVKGYTKGGPGHEEPIPMATYGWNLVKLYSGKDVFFTPPEKCDTLLCDIG
ESSPNPTIEEGRTLRVLKMVEPWLRGNQFCIKILNPYMPSVVETLEQMQRKHGGMLVRNPLSRNST
HEMYWVSCGTGNIVSAVNMTSRMLLNRFTMAHRKPTYERDVDLGAGTRHVAVEPEVANLDIIGQRI
ENIKHEHKSTWHYDEDNPYKTWAYHGSYEVKPSGSASSMVNGVVKLLTKPWDAIPMVTQIAMTDTT
PFGQQRVFKEKVDTRTPKAKRGTAQIMEVTARWLWGFLSRNKKPRICTREEFTRKVRSNAAIGAVF
VDENQWNSAKEAVEDERFWDLVHRERELHKQGKCATCVYNMMGKREKKLGEFGKAKGSRAIWYMWL
GARFLEFEALGFMNEDHWFSRENSLSGVEGEGLHKLGYILRDISKIPGGNMYADDTAGWDTRITED
DLQNEAKITDIMEPEHALLATSIFKLTYQNKVVRVQRPAKNGTVMDVISRRDQRGSGQVGTYGLNT
FTNMEAQLIRQMESEGIFSPSELETPNLAERVLDWLEKYGVERLKRMAISGDDCVVKPIDDRFATA
LTALNDMGKVRKDIPQWEPSKGWNDWQQVPFCSHHFHQLIMKDGREIVVPCRNQDELVGRARVSQG
AGWSLRETACLGKSYAQMWQLMYFHRRDLRLAANAICSAVPVDWVPTSRTTWSIHAHHQWMTTEDM
LSVWNRVWIEENPWMEDKTHVSSWEDVPYLGKREDQWCGSLIGLTARATWATNIQVAINQVRRLIG
NENYLDYMTSMKRFKNESDPKGHSGESTHL
000000000000000000000000000000000000000000000000000000000000000000
000000000000000000000000000000000000000000000000000000000000000000
000000000000000000000000000000000000000000000000000000000000000000
000000000000000000000000000000000000000000000000000000000000000000
000000000000000000000000000000000000000000000000000000000000000000
000000000000000000000000000000000000000000000000000000000000000000
000000000000000000000000000000000000000000000000000000000000000000
000000000000000000000000000000000000000000000000000000000000000000
000000000000000000000000000000000000000000000000000000000111111111
000000000000000000000000000000000000000000000000000000000000000000
011111111111111100000000000000000000000000000000000000000000000000
000000000000000000000000000000000000000000000000111111111111111000
000000000000000000000000000000000000000000000000000000000000000000
000000000000000000000000001111110000000000000000000000000000000000
000000000000000000000000000000000000000000000000000000000000000000
000000000000000000000000000000000000000000000000000000000000000000
000000000000000000000000000000000000000000000000000000000000000000
000000000000000000000000000000000000000000000000000000000000000000
000000000000000000000000000000000000000000000000000000000000000000

000000000000000000000000000000000000000000000000000000000000000000
000000000000000000000000000000000000000000000000000000000000000000
000000000000000000000000000000000000000000000000000000000000000000
000000000000000000000000000000000000000000000000000000000000000000
000000000000000000000000000000000000000000000000000000000000000000
000000000000000000000000000000000000000000000000000000000000000000
000000000000000000000000000000000000000000000000000000000000000000
000000000000000000000000000000000000000000000000000000000000000000
000000000000000000000000000000000000000000000000000000000000000000
000000000000000000000000000000000000000000000000000000000000000000
000000000000000000000000000000000000000000000000000000000000000000
000000000000000000000000000000000000000000000000000000000000000000
000000000000000000000000000000000000000000000000000000000000000000
000000000000000000000000000000000000000000000000000000000000000000
000000000000000000000000000000000000000000000000000000000000000000
000000000000000000000000000000000000000000000000000000000000000000
000000000000000000000000000000000000000000000000000000000000000000
000000000000000000000000000000000000000000000000000000000000000000
000000000000000000000000000000000000000000000000000000000000000000
000000000000000000000000000000000000000000000000000000000000000000
000000000000000000000000000000000000000000000000000000000000000000
000000000000000000000000000000000000000000000000000000000000000000
000000000000000000000000000000000000000000000000000000000000000000
000000000000000000000000000000000000000000000000000000000000000000
000000000000000000000000000000000000000000000000000000000000000000
000000000000000000000000000000000000000000000000000000000000000000
000000000000000000000000000000000000000000000000000000000000000000
000000000000000000000000000000000000000000000000000000000000000000
000000000000000000000000000000000000000000000000000000000000000000
000000000000000000000000000000000000000000000000000000000000000000
000000000000000000000000000000000000000000000000000000000000000000
000000000000000000000000000000000000000000000000000000000000000000
000000000000000000000000000000
>790646 Tc40 antigen. [mapped in Trypanosoma cruzi]
MTVTVDLFNHAKPSNNEGRVWSVDAATFNEVPEAQRVLADSQFYLAYTMKRRHVLRVVKRSNLLKG
TVRAHSKPIHAVKFVNYRSNVAASAGKGEFFVWVVTDETEASNGKPDLAARLTVKVYFKLQDPVTI
PCFSFFINAESQRPDLLVLYETQAAILDSSSLIERFDVESLEATLQRNCTTLRTLTQPVSENSLCS
VGSGGWFTFTTEPTMVAACTLRNRSTPSWACCEGEPVKALHLLDATVEENVSVLVAASTKGVYQWL

LTGVAEPNLLRKFVIDGSIVAMESSRETFAVFDDRKQLALVNMHSPHNFTCTHYMMPCQVQRNGFC
FNRTADGSCVLADMSNRLTIFHLRSSRREEQQPGQKTSVVATAKPGCVSSGTDAASSSHTNTTSAA
AASPASPPVSAPAKAAAPPAAARSAEPHVGSKIIANLVNQLGINVTQRSVVSTGAPATTRSTAVTS
TTTAPQRTSPYGHNGRPVTAGLVAANSGASAASSPTAAAKPTGEEKASAACETSSVAINATRPALH
NASLPQAPTDGVLAAAVYQSEGEVHQSLERLESVITNTSRVLKLLPDTIRRDHEQLLNLGLEAQMT
ELQQSRPTPQTQPRDTSSAKSSVFETYTLVLIADSLSRNITKGVKRGVNEAIMLHLDHEVRHAIGN
RLRQTQKNIIKSRLDEALKESTTQFTAQLTQTVENLVKRELAEVLGSINGSLTSLVKENASLQKEL
NSIMSSGVLDEMRRMREELCTLRESVAKRKATMPDSSLHATSSFQGRRSAPETILATALSMVREQQ
YRQGLEYMLMAQQPSLLLRFLSILTRENENAYSELIENVETPNDVWCSVLLQLIEAAATEAEKEVV
VGVAIDILSERDQIAQNGALGSKLTTAMRAFERQARSETTSRSFLQCLKNLEKLLQS
000000000000000000000000000000000000000000000000000000000000000000
000000000000000000000000000000000000000000000000000000000000000000
000000000000000000000000000000000000000000000000000000000000000000
000000000000000000000000000000000000000000000000000000000000000000
000000000000000000000000000000000000000000000000000000000000000000
000000000000000000000000000000000000000000000000000000000000000000
000000000001111111111000000000000000000000000000000000000000000000
000000000000000000000000000000000000000000000000000000000000000000
000000000000000000000000000000000000000000000000000000000000000000
000000000000000000000000000000000000000000000000000000000000000000
000000000000000000000000000000000000000000000000000000000000000000
000000000000000000000000000000000000000000000000000000000000000000
000000000000000000000000000000000000000000000000000000000000000000
000000000000000000000000000000000000000000000000000000000
>P19597 Circumsporozoite protein precursor (CS). [mapped in Plasmodium falciparum]
MMRKLAILSVSSFLFVEALFQEYQCYGSSSNTRVLNELNYDNAGTNLYNELEMNYYGKQENWYSLK
KNSRSLGENDDGNNEDNEKLRKPKHKKLKQPADGNPDPNANPNVDPNANPNVDPNANPNVDPNANP
NANPNANPNANPNANPNANPNANPNANPNANPNANPNANPNANPNANPNANPNANPNANPNANPNV
DPNANPNANPNANPNANPNANPNANPNANPNANPNANPNANPNANPNANPNANPNANPNANPNANP
NANPNANPNKNNQGNGQGHNMPNDPNRNVDENANANSAVKNNNNEEPSDKHIKEYLNKIQNSLSTE
WSPCSVTCGNGIQVRIKPGSANKPKDELDYANDIEKKICKMEKCSSVFNVVNSSIGLIMVLSFLFL
N
000000000000000000000000000000000000000000000000000000000000000000
000000000000000000000000000000000000000000000000000000000000001111
111111110000000000000000000000000000000000000000000000000000000000
000000000000000000000000000000000000000000000000000000000000000000
000000000000000000000000000000000000000000000000000000000000000000

000000000000000000000000000000000000000000000000000000000000000000
0
>P13664 Major surface antigen p30 precursor. [mapped in Toxoplasma gondii]
MSVSLHHFIISSGFLTSMFPKAVRRAVTAGVFAAPTLMSFLRCGVMASDPPLVANQVVTCPDKKST
AAVILTPTENHFTLKCPKTALTEPPTLAYSPNRQICPAGTTSSCTSKAVTLSSLIPEAEDSWWTGD
SASLDTAGIKLTVPIEKFPVTTQTFVVGCIKGDDAQSCMVTVTVQARASSVVNNVARCSYGADSTL
GPVKLSAEGPTTMTLVCGKDGVKVPQDNNQYCSGTTLTGCNEKSFKDILPKLTENPWQGNASSDKG
ATLTIKKEAFPAESKSVIIGCTGGSPEKHHCTVKLEFAGAAGSAKSAAGTASHVSIFAMVIGLIGS
IAACVA
000000000000000000000000000000000000000000000000000000000000000000
000000000000000000000000000000000000000000000000000000000000000000
000000000000000000000000000000000000000111111111111111111111111111
111111111111110000000000000000000000000000000000000000000000000000
000000000000000000000000000000000000000000000000000000000000000000
000000
>P35253 Structural glycoprotein precursor (Virion spike glycoprotein). [mapped in Lake Victoria marburgvirus - Musoke]
MKTTCFLISLILIQGTKNLPILEIASNNQPQNVDSVCSGTLQKTEDVHLMGFTLSGQKVADSPLEA
SKRWAFRTGVPPKNVEYTEGEEAKTCYNISVTDPSGKSLLLDPPTNIRDYPKCKTIHHIQGQNPHA
QGIALHLWGAFFLYDRIASTTMYRGKVFTEGNIAAMIVNKTVHKMIFSRQGQGYRHMNLTSTNKYW
TSSNGTQTNDTGCFGALQEYNSTKNQTCAPSKIPPPLPTARPEIKLTSTPTDATKLNTTDPSSDDE
DLATSGSGSGEREPHTTSDAVTKQGLSSTMPPTPSPQPSTPQQGGNNTNHSQDAVTELDKNNTTAQ
PSMPPHNTTTISTNNTSKHNFSTLSAPLQNTTNDNTQSTITENEQTSAPSITTLPPTGNPTTAKST
SSKKGPATTAPNTTNEHFTSPPPTPSSTAQHLVYFRRKRSILWREGDMFPFLDGLINAPIDFDPVP
NTKTIFDESSSSGASAEEDQHASPNISLTLSYFPNINENTAYSGENENDCDAELRIWSVQEDDLAA
GLSWIPFFGPGIEGLYTAVLIKNQNNLVCRLRRLANQTAKSLELLLRVTTEERTFSLINRHAIDFL
LTRWGGTCKVLGPDCCIGIEDLSKNISEQIDQIKKDEQKEGTGWGLGGKWWTSDWGVLTNLGILLL
LSIAVLIALSCICRIFTKYIG
000000000000000000000000000000000000000000000000000000000000000000
000000000000000000000000000000000000000000000000000000000000000000
000000000000000000000000000000000000000000000000000000000000000000
000000000000000000000000000000000000000000000000000000000000011111
111110000000000000000000000000000000000000000000111111111000000
000000000000000000000000000000000000000000000000000000000000000000
000000000000000000000000000000000000000000000000000000000000000000
000000000000000000000000000000000000000000000000000000000000000000
000000000000000000000000000000000000000000000000000000000000000000

0000000000000000000000000000000000000000000000000000000000000000000000
0000000000000000000000
>P13128 Listeriolysin O precursor (Thiol-activated cytolysin) (LLO). [mapped in Listeria monocytogenes EGD-e]
MKKIMLVFITLILVSLPIAQQTEAKDASAFNKENSISSMAPPASPPASPKTPIEKKHADEIDKYIQ
GLDYNKNNVLVYHGDAVTNVPPRKGYKDGNEYIVVEKKKKSINQNNADIQVVNAISSLTYPGALVK
ANSELVENQPDVLPVKRDSLTLSIDLPGMTNQDNKIVVKNATKSNVNNAVNTLVERWNEKYAQAYP
NVSAKIDYDDEMAYSESQLIAKFGTAFKAVNNSLNVNFGAISEGKMQEEVISFKQIYYNVNVNEPT
RPSRFFGKAVTKEQLQALGVNAENPPAYISSVAYGRQVYLKLSTNSHSTKVKAAFDAAVSGKSVSG
DVELTNIIKNSSFKAVIYGGSAKDEVQIIDGNLGDLRDILKKGATFNRETPGVPIAYTTNFLKDNE
LAVIKNNSEYIETTSKAYTDGKINIDHSGGYVAQFNISWDEVNYDPEGNEIVQHKNWSENNKSKLA
HFTSSIYLPGNARNINVYAKECTGLAWEWWRTVIDDRNLPLVKNRNISIWGTTLYPKYSNKVDNPI
E
0000000000000000000000000000000000000000000000000000000000000011111111
0000000000000000000000000000000000000000000000000000000000000000000000
0000000000000000000111111110000000000000000000000000000111111000000000
0000000000000000000000000000000000000000000000000000000000000000000000
0000001111111110000000000000000000000000000000000000000000000000000000
0000000000000000000000000000000000000000000000000000000000000000000000
0000000000000000000000000000000000000000000000000000000000000000000000
0000000000000000000000000000000000000000000000000000000000000000000000
0
>1930067 envelope protein [Japanese encephalitis virus]. [mapped in Japanese encephalitis virus strain Nakayama]
FNCLGMGNRDFIEGASGATWVDLVLEGDSCLTIMANDKPTLDVRMINIEASQLAEVRSYCYHASVT
DISTVARCPTTGEAHNEKQADSSYVCKQGFTDRGWGNGCGLFGKGSIDTCAKFSCTSKAIGRTIQP
ENIKYEVGIFVHGTTTSENHGNYSAQVGASQAAKFTVTPNAPSITLKLGDYGEVTLDCEPRSGLNT
EAFYVMTVGSKSFLVHREWFHDLALPWTSPSSTAWRNRELLMEFEEAHATKQSVVALGSQEGGLHQ
ALAGAIVVEYSSSVKLTSGHLKCRLKMDKLALKGTTYGMCTEKFSFAKNPADTGHGTVVIELSYSG
SDGPCKIPIVSVASLNDMTPVGRLVTVNPFVATSSANSKVLVEMEPPFGDSYIVVGMGDKQINHHW
HKAGSTLGKAFSTTLKGAQRLAALGDTAWDFGSIGGVFNSIGKAVHQVFGGAFRTLFGGMSWITQG
LMGALLLWMGVNARDRSIALAFLATGGVLVFLATNVHA
0000000000000000000000000000000000000000000000000000000000000000000000
0000000000001111111111111111111111111111111000000000000000000000000000
0000000000000000111111111111111000000000000000000000000000000000000000
0000000000000000000000000000000000000000000000000000000000000000000000
0000000000000000000000000000000000000000000000000000000000000000000000
0000000000000000000000000000000000000000000000111111111111111111111111

111000000000000000000000000000000000000000000000000000000000000000
00000000000000000000000000000000000000

>P59594 Spike glycoprotein precursor (S glycoprotein) (Peplomer protein) (E2) [Contains: Spike protein S1; Spike protein S2]. [mapped in SARS coronavirus BJ01]

MFIFLLFLTLTSGSDLDRCTTFDDVQAPNYTQHTSSMRGVYYPDEIFRSDTLYLTQDLFLPFYSNV
TGFHTINHTFGNPVIPFKDGIYFAATEKSNVVRGWVFGSTMNNKSQSVIIINNSTNVVIRACNFEL
CDNPFFAVSKPMGTQTHTMIFDNAFNCTFEYISDAFSLDVSEKSGNFKHLREFVFKNKDGFLYVYK
GYQPIDVVRDLPSGFNTLKPIFKLPLGINITNFRAILTAFSPAQDIWGTSAAAYFVGYLKPTTFML
KYDENGTITDAVDCSQNPLAELKCSVKSFEIDKGIYQTSNFRVVPSGDVVRFPNITNLCPFGEVFN
ATKFPSVYAWERKKISNCVADYSVLYNSTFFSTFKCYGVSATKLNDLCFSNVYADSFVVKGDDVRQ
IAPGQTGVIADYNYKLPDDFMGCVLAWNTRNIDATSTGNYNYKYRYLRHGKLRPFERDISNVPFSP
DGKPCTPPALNCYWPLNDYGFYTTTGIGYQPYRVVVLSFELLNAPATVCGPKLSTDLIKNQCVNFN
FNGLTGTGVLTPSSKRFQPFQQFGRDVSDFTDSVRDPKTSEILDISPCSFGGVSVITPGTNASSEV
AVLYQDVNCTDVSTAIHADQLTPAWRIYSTGNNVFQTQAGCLIGAEHVDTSYECDIPIGAGICASY
HTVSLLRSTSQKSIVAYTMSLGADSSIAYSNNTIAIPTNFSISITTEVMPVSMAKTSVDCNMYICG
DSTECANLLLQYGSFCTQLNRALSGIAAEQDRNTREVFAQVKQMYKTPTLKYFGGFNFSQILPDPL
KPTKRSFIEDLLFNKVTLADAGFMKQYGECLGDINARDLICAQKFNGLTVLPPLLTDDMIAAYTAA
LVSGTATAGWTFGAGAALQIPFAMQMAYRFNGIGVTQNVLYENQKQIANQFNKAISQIQESLTTTS
TALGKLQDVVNQNAQALNTLVKQLSSNFGAISSVLNDILSRLDKVEAEVQIDRLITGRLQSLQTYV
TQQLIRAAEIRASANLAATKMSECVLGQSKRVDFCGKGYHLMSFPQAAPHGVVFLHVTYVPSQERN
FTTAPAICHEGKAYFPREGVFVFNGTSWFITQRNFFSPQIITTDNTFVSGNCDVVIGIINNTVYDP
LQPELDSFKEELDKYFKNHTSPDVDLGDISGINASVVNIQKEIDRLNEVAKNLNESLIDLQELGKY
EQYIKWPWYVWLGFIAGLIAIVMVTILLCCMTSCCSCLKGACSCGSCCKFDEDDSEPVLKGVKLHY
T

000000000000000000000000000000000000000000000000000000000000000000
000000000000000000000000000000000000000000000000000000000000000000
000000000000000000000000000000000000000000000000000000000000000000
000000000000000000000000000000000000000000000000000000000000000000
000000000000011111111111111111111111111111100000000000000000000000
000000000000000000000000000000000000000000000000000000000000000000
000000000000000000000000000000000000000000000000000000000000000000
000000000000000000000000000000000000000000000000011111111111111111
111111111111111111111111111111111111111111111111111110111111111111
111111111111111111111111111111111111000000000000000000000000000000
000000000000000000000000000000000000000000000000000000000000000000
000000000000000000000000000000000000000000000000000000000001111111
111111111111111111111111111111111111000000000000000000000000000000

000000000000000000000000000000000000000000000000000000000000000000
000000000000000000000000000000000000000000000000000000000000000000
000000000000000000000000000000000000000000000000000000000000000000
000000000000000000000000000000000000000000000000000000000000000000
000000000000000000001111111111111110000000000000000000000000000000
000000000000000000000000000000000000000000000000000000000000000000
0
>13622466 protein GRAB (protein G-related alpha 2M-binding protein) [Streptococcus pyogenes M1 GAS]. [mapped in Streptococcus pyogenes M1 GAS]
MGKEIKVKCFLRRSAFGLVAVSASVLVGSTVSAVDSPIEQPRIIPNGGTLTNLLGNAPEKLALRNE
ERAIDELKKQAIEDKEATTAIEAASSDALEALADQTDALQSEEAAVVKADNAASDALEALADQTDA
LQSEEAEVVQSDNAASDAWEKAATPIALDVKKTKDTKPVVKKEERQNVNTLPTTGEESNPFFTAAA
LAIMVSTGVLVVSSKCKEN
000000000000000000000000000000000011111111111111111000000011111111
111111111111111100000000000000000000000000000000000000000000000000
000000000000000000000000000000111111111111111111000000000000000000
0000000000000000000
>2327073 polyprotein [Hepatitis C virus strain H77]. [mapped in Hepatitis C virus (isolate H77)]
MSTNPKPQRKTKRNTNRRPQDVKFPGGGQIVGGVYLLPRRGPRLGVRATRKTSERSQPRGRRQPIP
KARRPEGRTWAQPGYPWPLYGNEGCGWAGWLLSPRGSRPSWGPTDPRRRSRNLGKVIDTLTCGFAD
LMGYIPLVGAPLGGAARALAHGVRVLEDGVNYATGNLPGCSFSIFLLALLSCLTVPASAYQVRNSS
GLYHVTNDCPNSSIVYEAADAILHTPGCVPCVREGNASRCWVAVTPTVATRDGKLPTTQLRRHIDL
LVGSATLCSALYVGDLCGSVFLVGQLFTFSPRRHWTTQSCNCSIYPGHITGHRMAWDMMMNWSPTA
ALVVAQLLRIPQAIMDMIAGAHWGVLAGIAYFSMVGNWAKVLVVLLLFAGVDAETHVTGGSAGHTT
AGLVGLLTPGAKQNIQLINTNGSWHINSTALNCNDSLTTGWLAGLFYRHKFNSSGCPERLASCRRL
TDFAQGWGPISYANGSGLDERPYCWHYPPRPCGIVPAKSVCGPVYCFTPSPVVVGTTDRSGAPTYS
WGANDTDVFVLNNTRPPLGNWFGCTWMNSTGFTKVRGVPPCVIGGVGNNTLLCPTDCFRKHPEATY
SRCGSGPWITPRCMVDYPYRLWHYPCTINYTIFKVRMYVGGVEHRLEAACNWTRGGRCDLEDRDRS
ELSPLLLSTTQWQVLPCSFTTLPALSTGLIHLHQNIVDVQYLYGVGSSIASWAIKWEYVVLLFLLL
ADARVCSCLWMMLLISQAEAALENLVILNAASLAGTHGLVSFLVFFRFAWYLKGRWVPGAVYAFYG
MWPLLLLLLALPQRAYALDTEVAASCGGVVLVGLMALTLSPYYKRYISWCMWWLQYFLTRVEAQLH
VWVPPLNVRGGRDAVILLMCVVHPTLVFDITKLLLAIFGPLWILQASLLKVPYFVRVQGLLRICAL
ARKIAGGHYVQMAIIKLGALTGTYVYNHLTPLRDWAHNGLRDLAVAVEPVVFSRMETKLITWGADT
AACGDIINGLPVSARRGQEILLGPADGMVSKGWRLLAPITAYAQQTRGLLGCIITSLTSRDKNQVE
GEVQIVSTATQTFLATCINGVCWTVYHGAGTRTIASPKGPVIQMYTNVDQDLVGWPAPQGSRSLTP
CTCGSSDLYLVTRHADVIPVRRRGDSRGSLLSPRPISYLKGSSGGPLLCPAGHAVGLFRAAVCTRG

VAKAVDFIPVENLGTTMRSPVFTDNPSPPAVPQSFQVAHLHAPTGSGKSTKVPAAYAAQGYKVLVL
NPSVAATLGFGAYMSKAHGVDPNIRTGVRTITTGSPITYSTYGKFLADGGCSGGAYDIIICDECHS
TDATSILGIGTVLDQAETAGARLVVLATATPPGSVTVSHPNIEEVALSTTGEIPFYGKAIPLEVIK
GGRHLIFCHSKKKCDELAAKLVALGINAVAYYRGLDVSVIPTSGDVVVVSTDALMTGFTGDFDSVI
DCNTCVTQTVDFSLDPTFTIETTTLPQDAVSRTQRRGRTGRGKPGIYRFVAPGERPSGMFDSSVLC
ECYDAGCAWYELTPAETTVRLRAYMNTPGLPVCQDHLEFWEGVFTGLTHIDAHFLSQTKQSGENFP
YLVAYQATVCARAQAPPPSWDQMWKCLIRLKPTLHGPTPLLYRLGAVQNEVTLTHPITKYIMTCMS
ADLEVVTSTWVLVGGVLAALAAYCLSTGCVVIVGRIVLSGKPAIIPDREVLYQEFDEMEECSQHLP
YIEQGMMLAEQFKQKALGLLQTASRHAEVITPAVQTNWQKLEVFWAKHMWNFISGIQYLAGLSTLP
GNPAIASLMAFTAAVTSPLTTGQTLLFNILGGWVAAQLAAPGAATAFVGAGLAGAAIGSVGLGKVL
VDILAGYGAGVAGALVAFKIMSGEVPSTEDLVNLLPAILSPGALVVGVVCAAILRRHVGPGEGAVQ
WMNRLIAFASRGNHVSPTHYVPESDVAARVTAILSSLTVTQLLRRLHQWISSECTTPCSGSWLRDI
WDWICEVLSDFKTWLKAKLMPQLPGIPFVSCQRGYRGVWRGDGIMHTRCHCGAEITGHVKNGAMRI
VGPRTCRNMWSGTFLINAYTTGPCTPLPAPNYKFALWRVSAEEYVEIRRVGDFHYISGMTTDNLKC
PCQIPSPEFFTELDGVRLHRFAPPCKPLLREEVSFRVGLHEYPVGSQLPCEPEPDVAVLTSMLTDP
SHITAEEAGRRLARGSPPSMASSSASQLSAPSLKATCTANHDSPDAELIEANLLWRQEMGGNITRV
ESENKVVILDSFDPLVAEEDEREVSVPAEILRKSRRFAPALPVWARPDYNPPLVETWKKPDYEPPV
VHGCPLPPPRSPPVPPPRKKRTVVLTESTLSTALAELATKSFGSPSTSGITGDNTTTSSEPAPSGC
PPDSDVESYSSMPPLEGEPGDPDFSDGSWSTVSSGADTEDVVCCSMSYTWTGALVTPCAAEEQKLP
INALSNSLLRHHNLVYSTTSRSACQRQKKVTFDRLQVLDSHYQDVLKEVKAAASKVKANLLSVEEA
CSLTPPHSAKSKFGYGAKDVRCHARKAVAHINSVWKDLLEDSVTPIDTTIMAKNEVFCVQPEKGGR
KPARLIVFPDLGVRVCEKMALYDVVSKLPLAVMGSSYGFQYSPGQRVEFLVQAWKSKKTPMGFSYD
TRCFDSTVTESDIRTEEAIYQCCDLDPQARVAIKSLTERLYVGGPLTNSRGENCGYRRCRASGVLT
TSCGNTLTCYIKARAACRAAGLQDCTMLVCGDDLVVICESAGVQEDAASLRAFTEAMTRYSAPPGD
PPQPEYDLELITSCSSNVSVAHDGAGKRVYYLTRDPTTPLARAAWETARHTPVNSWLGNIIMFAPT
LWARMILMTHFFSVLIARDQLEQALNCEIYGACYSIEPLDLPPIIQRLHGLSAFSLHSYSPGEINR
VAACLRKLGVPPLRAWRHRARSVRARLLSRGGRAAICGKYLFNWAVRTKLKLTPIAAAGRLDLSGW
FTAGYSGGDIYHSVSHARPRWFWFCLLLLAAGVGIYLLPNR
000000000000000000000000000000000000000000000000000000000000
000000000000000000000000000000000000000000000000000000000000
000000000000000000000000000000000000000000000000000000000000
000000000000000000000000000000000000000000000000000000000000
000000000000000000000000000000000000000000000111111110000000
000000000000000000000000000000000000000000000000111111111111
111111111111111111111111000000000001111111111000000000000000
000000000000000011111111110000000000000000000000000000000000
000000000000011111111000000000000000000000000000000000000000
000000000000000000000000000000000000000000000000000000000000

000000000000000000000000000000000000000000000000000000000000000000
000000000000000000000000000000000000000000000000000000000000000000
000000000000000000000000000000000000000000000000000000000000000000
000000000000000000000000000000000000000000000000000000000000000000
000000000000000000000000000000000000000000000000000000000000000000
000000000000000000000000000000000000000000000000000000000000000000
000000000000000000000000000000000000000000000000000000000000000000
000000000000000000000000000000000000000000000000000000000000000000
000000000000000000000000000000000000000000000000000000000000000000
000000000000000000000000000000000000000000000000000000000000000000
000000000000000000000000000000000000000000000000000000000000000000
000000000000000000000000000000000000000000000000000000000000000000
000000000000000000000000000000000000000000000000000000000000000000
000000000000000000000000000000000000000000000000000000000000000000
000000000000000000000000000000000000000000000000000000000000000000
000000000000000000000000000000000000000000000000000000000000000000
000000000000000000000000000000000000000000000000000000000000000000
000000000000000000000000000000000000000000000000000000000000000000
000000000000000000000000000000000000000000000000000000000000000000
000000111111111111111111110000000000000000000000000000000000000000
000000000000000000000000000000000000000000000000000000000000000000
000000000000000000000000000000000000000000000000000000000000000000
000000000000000000000000000000000000000000000000000000000000000000
000000000000000000000000000000000000000000000000000000000000000000
000000000000000000000000000000000000000000000000000000000000000000
000000000000000000000000000000000000000000000000000000000000000000
000000000000000000000000000000000000000000000000000000000000000000
000000000000000000000000000000000000000000000000000000000000000000
000000000000000000000000000000000000000000000000000000000000000000
000000000000000000000000000000000000000000000000000000000000000000
000000000000000000000000000000000000000000000000000000000000000000
000000000000000000000000000000000000000000000000000000000000000000
000000000000000000000000000000000000000000000000000000000000000000
000000000000000000000000000000000000000000000000000000000000000000
000000000000000000000000000000000000000000000000000000000000000000
00000000000000000000000000000000000000000
>P08669 Glycoprotein polyprotein [Contains: Glycoprotein G1; Glycoprotein G2]. [mapped in Lassa virus]

MGQIVTFFQEVPHVIEEVMNIVLIALSVLAVLKGLYNFATCGLVGLVTFLLLCGRSCTTSLYKGVY
ELQTLELNMETLNMTMPLSCTKNNSHHYIMVGNETGLELTLTNTSIINHKFCNLSDAHKKNLYDHA
LMSIISTFHLSIPNFNQYEAMSCDFNGGKISVQYNLSHSYAGDAANHCGTVANGVLQTFMRMAWGG
SYIALDSGRGNWDCIMTSYQYLIIQNTTWEDHCQFSRPSPIGYLGLLSQRTRDIYISRRLLGTFTW
TLSDSEGKDTPGGYCLTRWMLIEAELKCFGNTAVAKCNEKHDEEFCDMLRLFDFNKQAIQRLKAEA
QMSIQLINKAVNALINDQLIMKNHLRDIMGIPYCNYSKYWYLNHTTTGRTSLPKCWLVSNGSYLNE
THFSDDIEQQADNMITEMLQKEYMERQGKTPLGLVDLFVFSTSFYLISIFLHLVKIPTHRHIVGKS
CPKPHRLNHMGICSCGLYKQPGVPVKWKR
000000000000000000000000000000000000000000000000000000000000000000
000000000000000000000000000000000000000000000000000011111111111111
100000000000000000000000000000000000000000000000000000000000000000
000000000000000000000000000000000000000000000000000000000000000000
000000000000000000000000000000000000000000000000000000000000000000
000000000000000000000000000000000000000000000000000000000000000000
000000000000000000000000000000000000000000000000000000000000000000
00000000000000000000000000000
>116774 Probable coat protein VP1. [mapped in Human parvovirus B19]
MSKKSGKWWESDDKFAKAVYQQFVEFYEKVTGTDLELIQILKDHYNISLDNPLENPSSLFDLVARI
KNNLKNSPDLYSHHFQSHGQLSDHPHALSSSSSHAEPRGENAVLSSEDLHKPGQVSVQLPGTNYVG
PGNELQAGPPQSAVDSAARIHDFRYSQLAKLGINPYTHWTVADEELLKNIKNETGFQAQVVKDYFT
LKGAAAPVAHFQGSLPEVPAYNASEKYPSMTSVNSAEASTGAGGGGSNSVKSMWSEGATFSANSVT
CTFSRQFLIPYDPEHHYKVFSPAASSCHNASGKEAKVCTISPIMGYSTPWRYLDFNALNLFFSPLE
FQHLIENYGSIAPDALTVTISEIAVKDVTDKTGGGVQVTDSTTGRLCMLVDHEYKYPYVLGQGQDT
LAPELPIWVYFPPQYAYLTVGDVNTQGISGDSKKLASEESAFYVLEHSSFQLLGTGGTASMSYKFP
PVPPENLEGCSQHFYEMYNPLYGSRLGVPDTLGGDPKFRSLTHEDHAIQPQNFMPGPLVNSVSTKE
GDSSNTGAGKALTGLSTGTSQNTRISLRPGPVSQPYHHWDTDKYVTGINAISHGQTTYGNAEDKEY
QQGVGRFPNEKEQLKQLQGLNMHTYFPNKGTQQYTDQIERPLMVGSVWNRRALHYESQLWSKIPNL
DDSFKTQFAALGGWGLHQPPPQIFLKILPQSGPIGGIKSMGITTLVQYAVGIMTVTMTFKLGPRKA
TGRWNPQPGVYPPHAAGHLPYVLYDPTATDAKQHHRHGYEKPEELWTAKSRVHPL
111111111111111111111111111111111111111111111111111111111111111111
100000000001111111111111111111111100000000001111111111111111111111
111111111111111111111111111111111111111111111111111111111111111111
111111111111111111111111111111111111111111111111111111111111100000
000000000000000000000000000000000000000000000000000000000000000000
000000000000000000000000000000000000000000000000000000000000000000
000000000000000000000000000000000000000000000000000000000000000000
000000000000000001111111111111111111100000000000000000000000000000
000000011111111111111111111111111111111111100000000000000111111111

1111111111111000000000000000000000000000000000000000000000000000000
000000000000000000000000000000000000000000000000000000000011111111
1111111111111111000000000000000000001111111111111111111
>153640 glucosyltransferase. [mapped in Streptococcus mutans]
MDKKVRYKLRKVKKRWVTVSVASAVMTLTTLSGGLVKADSNESKSQISNDSNTSVVTANEESNVIT
EATSKQEAASSQTNHTVTTSSSSTSVVNPKEVVSNPYTVGETASNGEKLQNQTTTVDKTSEAAANN
ISKQTTEADTDVIDDSNAANLQILEKLPNVKEIDGKYYYYDNNGKVRTNFTLIADGKILHFDETGA
YTDTSIDTVNKDIVTTRSNLYKKYNQVYDRSAQSFEHVDHYLTAESWYRPKYILKDGKTWTQSTEK
DFRPLLMTWWPDQETQRQYVNYMNAQLGINKTYDDTSNQLQLNIAAATIQAKIEAKITTLKNTDWL
RQTISAFVKTQSAWNSDSEKPFDDHLQNGAVLYDNEGKLTPYANSNYRILNRTPTNQTGKKDPRYT
ADNTIGGYEFLLANDVDNSNPVVQAEQLNWLHFLMNFGNIYANDPDANFDSIRVDAVDNVDADLLQ
IAGDYLKAAKGIHKNDKAANDHLSILEAWSDNDTPYLHDDGDNMINMDNKLRLSLLFSLAKPLNQR
SGMNPLITNSLVNRTDDNAETAAVPSYSFIRAHDSEVQDLIADIIKAEINPNVVGYSFTMEEIKKA
FEIYNKDLLATEKKYTHYNTALSYALLLTNKSSVPRVYYGDMFTDDGQYMAHKTINYEAIETLLKA
RIKYVSGGQAMRNQQVGNSEIITSVRYGKGALKATDTGDRTTRTSGVAVIEGNNPSLRLKASDRVV
VNMGAAHKNQAYRPLLLTTDNGIKAYHSDQEAAGLVRYTNDRGELIFTAADIKGYANPQVSGYLGV
WVPVGAALIKMFALRLARPHQQMASVHQNAALDSRVMFEGFSNFQAFATKKEEYTNVVIAKNVDKF
AEWGVTDFEMAPQYVSSTDGSFLDSVIQNGYAFTDRYDLGISKPNKYGTADDLVKAIKALHSKGIK
VMADWVPDQMYAFPEKEVVTATRVDKYGTPVAGSQIKNTLYVVDGKSSGKDQQAKYGGAFLEELQA
KYPELFARKQISTGVPMDPSVKIKQWSAKYFNGTNILGRGAGYVLKDQATNTYFNISDNKEINFLP
KTLLNQDSQVGFSYDGKGYVYYSTSGYQAKNTFISEGDKWYYFDNNGYMVTGAQSINGVNYYFLSN
GLQLRDAILKNEDGTYAYYGNDGRRYENGYYQFMSGVWRHFNNGEMSVGLTVIDGQVQYFDEMGYQ
AKGKFVTTADGKIRYFDKQSGNMYRNRFIENEEGKWLYLGEDGAAVTGSQTINGQHLYFRANGVQV
KGEFVTDHHGRISYYDGNSGDQIRNRFVRNAQGQWFYFDNNGYAVTGARTINGQLLYFRANGVQVK
GEFVTDRYGRISYYDGNSGDQIRNRFVRNAQGQWFYFDNNGYAVTGARTINGQHLYFRANGVQVKG
EFVTDRHGRISYYDGNSGDQIRNRFVRNAQGQWFYFDNNGYAVTGARTINGQHLYFRANGVQVKGE
FVTDRYGRISYYDANSGERVRIN
000000000000000000000000000000000000000000000000000000000000000000
000000000000000000000000000000000000000000000000000000000000000000
000000000000000000000000000000000000000000000000000000000000000000
000000000000000000000000000000000000000000000000000000000000000000
000000000000000000000000000000000000000000000000000000000000000000
000000000000000000000000000000000000000000000000000000000000000000
000000000000000000000000000000000000000000000000000000000000000000
000000000000000000000000000000000000000000000000000000000000000000
000000000000000000000001111111111111111111000000000000000000000000
000000000000000000000000000000000000000000000000000000000000000000
000000000000000000000000000000000000000000000000000000000000000000

000000000000000000000000000000000000000000000000000000000000000000
000000000000000000000000000000000000000000000000000000000000000000
000000000000000000000000000000000000000000000000000000000000000000
000000000000000000000000000000000000000000000000000000000000000000
000000000000000000000000000000000000000000000000000000000000000000
000000000000000000000000000000000000000000000000000000000000000000
000000000000000000000000000000000000000000000000000000000000000000
000000000000000000000000000000000000000000000000000000000000000000
000000000000000000000000000000000000000000000000000000000000000000
000000000000000000000000000000000000000000000000000000000000000000
000000000000000000000000000000000000000000000000000000000000000000
00000000000000000000000
>37724690 capsid protein [Hepatitis E virus]. [mapped in Hepatitis E virus]
LDWTKVTLDGRPLSTIQQYSKTFFVLPLRGKLSFWEAGTTKAGYPYNYNTTASDQLLVENAAGHRV
AISTYTTSLGAGPVSISAVAVLAPHSVLALLEDTMDYPARAHTFDDFCPECRPLGLQGCAFQSTVA
ELQRLKMKVVSPGG
000000000000000000000000000000000000000000000000000000000000000011
111111111111111111111111111100000000000000000000000000000000000000
00000000000000
>P02977 M protein, serotype 5 precursor. [mapped in Streptococcus pyogenes serotype M5]
MARENTNKHYWLRKLKKGTASVAVALSVLGAGLVVNTNEVSAAVTRGTINDPQRAKEALDKYELEN
HDLKTKNEGLKTENEGLKTENEGLKTENEGLKTEKKEHEAENDKLKQQRDTLSTQKETLEREVQNT
QYNNETLKIKNGDLTKELNKTRQELANKQQESKENEKALNELLEKTVKDKIAKEQENKETIGTLKK
ILDETVKDKIAKEQENKETIGTLKKILDETVKDKLAKEQKSKQNIGALKQELAKKDEANKISDASR
KGLRRDLDASREAKKQLEAEHQKLEEQNKISEASRKGLRRDLDASREAKKQLEAEQQKLEEQNKIS
EASRKGLRRDLDASREAKKQVEKALEEANSKLAALEKLNKELEESKKLTEKEKAELQAKLEAEAKA
LKEQLAKQAEELAKLRAGKASDSQTPDTKPGNKAVPGKGQAPQAGTKPNQNKAPMKETKRQLPSTG
ETANPFFTAAALTVMATAGVAAVVKRKEEN
111111111111111111111111111111111111111111111111111111111111111111
110000000000000000000000000000000000000000000000000000000000000000
000000000000000000000000000000000000000000000000000000000000000000
000000000000000000000000000000000000000000000000000000000000000000
000000000000000000000000000000000000000000000000000000000000000000
000000111111111111111111100000000000000000000000000000000000000000
000000000000000000000000000000000000000000000000000000000000000000
000000000000000000000000000000

>P13794 Outer membrane porin F precursor. [mapped in Pseudomonas aeruginosa]

MKLKNTLGVVIGSLVAASAMNAFAQGQNSVEIEAFGKRYFTDSVRNMKNADLYGGSIGYFLTDDVE
LALSYGEYHDVRGTYETGNKKVHGNLTSLDAIYHFGTPGVGLRPYVSAGLAHQNITNINSDSQGRQ
QMTMANIGAGLKYYFTENFFAKASLDGQYGLEKRDNGHQGEWMAGLGVGFNFGGSKAAPAPEPVAD
VCSDSDNDGVCDNVDKCPDTPANVTVDANGCPAVAEVVRVQLDVKFDFDKSKVKENSYADIKNLAD
FMKQYPSTSTTVEGHTDSVGTDAYNQKLSERRANAVRDVLVNEYGVEGGRVNAVGYGESRPVADNA
TAEGRAINRRVEAEVEAEAK
00000000000000000000000000000000000000000000000000000000000000000
00000000000000000000000000000000000000000000000000000000000000000
00000000000000000000000000000000000000000000000000000000000000000
00000000000000000000000000000000000000000000000000000000000000000
00000000000000000000000000000000000000000000000000000000000000000
01111111111100000000

>P13423 Protective antigen precursor (PA) (PA-83) (PA83) (Anthrax toxins translocating protein) [Contains: Protective antigen PA-20 (PA20); Protective antigen PA-63 (PA63)]. [mapped in Bacillus anthracis]

MKKRKVLIPLMALSTILVSSTGNLEVIQAEVKQENRLLNESESSSQGLLGYYFSDLNFQAPMVVTS
STTGDLSIPSSELENIPSENQYFQSAIWSGFIKVKKSDEYTFATSADNHVTMWVDDQEVINKASNS
NKIRLEKGRLYQIKIQYQRENPTEKGLDFKLYWTDSQNKKEVISSDNLQLPELKQKSSNSRKKRST
SAGPTVPDRDNDGIPDSLEVEGYTVDVKNKRTFLSPWISNIHEKKGLTKYKSSPEKWSTASDPYSD
FEKVTGRIDKNVSPEARHPLVAAYPIVHVDMENIILSKNEDQSTQNTDSQTRTISKNTSTSRTHTS
EVHGNAEVHASFFDIGGSVSAGFSNSNSSTVAIDHSLSLAGERTWAETMGLNTADTARLNANIRYV
NTGTAPIYNVLPTTSLVLGKNQTLATIKAKENQLSQILAPNNYYPSKNLAPIALNAQDDFSSTPIT
MNYNQFLELEKTKQLRLDTDQVYGNIATYNFENGRVRVDTGSNWSEVLPQIQETTARIIFNGKDLN
LVERRIAAVNPSDPLETTKPDMTLKEALKIAFGFNEPNGNLQYQGKDITEFDFNFDQQTSQNIKNQ
LAELNATNIYTVLDKIKLNAKMNILIRDKRFHYDRNNIAVGADESVVKEAHREVINSSTEGLLLNI
DKDIRKILSGYIVEIEDTEGLKEVINDRYDMLNISSLRQDGKTFIDFKKYNDKLPLYISNPNYKVN
VYAVTKENTIINPSENGDTSTNGIKKILIFSKKGYEIG
00000000000000000000000000000000000000000000000000000000000000000
00000000000000000000000000000000000000000000000000000000000000000
00000000000000000000000000000000000000000000000000000000000000000
00000000000000000000000000000000000000000000000000000000000000000
00000000000000000000000000000000000000000000000000000000000000000
00000000001111000000000000000000000000000000000000000000000000000
00000000000000000000000000000000000000000000000000000000000000000
00000000000000000000000000000000000000000000000000000000000000000

000000000000000000000000000000000000000000000000000000000000000000
000000000000000111111111111111111111000000000000000000000000000000
000000000000000000000000000000000000000000000000000000111111111000
00000000000000000000000000000000000000
>P13403 Dense granule protein 1 precursor (Protein GRA 1) (Major antigen p24). [mapped in Toxoplasma gondii]
MVRVSAIVGAAASVFVCLSAGAYAAEGGDNQSSAVSDRASLFGLLSGGTGQGLGIGESVDLEMMGN
TYRVERPTGNPDLLKIAIKASDGSYSEVGNVNVEEVIDTMKSMQRDEDIFLRALNKGETVEEAIED
VAQAEGLNSEQTLQLEDAVSAVASVVQDEMKVIDDVQQLEKDKQQLKDDIGFLTGERE
000000000000000000000000000000000000000000000000000000000000000000
000000000000000000000000000000000111111111111111000000000000000000
0000000000000000000000000001111111111111111111111111111110
>97812 fibronectin-binding protein - Staphylococcus aureus. [mapped in Staphylococcus aureus]
VKNNLRYGIRKHKLGAASVFLGTMIVVGMGQDKEAAASEQKTTTVEENGNSATDNKTSETQTTATN
VNHIEETQSYNATVTEQPSNATQVTTEEAPKAVQAPQTAQPANIETVKEEVVKEEAKPQVKETTQS
QDNSGDQRQVDLTPKKATQNQVAETQVEVAQPRTASESKPRVTRSADVAEAKEASNAKVETGTDVT
SKVTVEIGSIEGHNNTNKVEPHAGQRAVLKYKLKFENGLHQGDYFDFTLSNNVNTHGVSTARKVPE
IKNGSVVMATGEVLEGGKIRYTFTNDIEDKVDVTAELEINLFIDPKTVQTNGNQTITSTLNEEQTS
KELDVKYKDGIGNYYANLNGSIETFNKANNRFSHVAFIKPNNGKTTSVTVTGTLMKGSNQNGNQPK
VRIFEYLGNNEDIAKSVYANTTDTSKFKEVTSNMSGNLNLQNNGSYSLNIENLDKTYVVHYDGEYL
NGTDEVDFRTQMVGHPEQLYKYYYDRGYTLTWDNGLVLYSNKANGNEKNGPIIQNNKFEYKEDTIK
ETLTGQYDKNLVTTVEEEYDSSTLDIDYHTAIDGGGGYVDGYIETIEETDSSAIDIDYHTAVDSEA
GHVGGYTESSEESNPIDFEESTHENSKHHADVVEYEEDTNPGGGQVTTESNLVEFDEESTKGIVTG
AVSDHTTVEDTKEYTTESNLIELVDELPEEHGQAQGPVEEITKNNHHISHSGLGTENGHGNYDVIE
EIEENSHVDIKSELGYEGGQNSGNQSFEEDTEEDKPKYEQGGNIVDIDFDSVPQIHGQNKGNQSFE
EDTEKDKPKYEHGGNIIDIDFDSVPHIHGFNKHTEIIEEDTNKDKPSYQFGGHNSVDFEEDTLPKV
SGQNEGQQTIEEDTTPPIVPPTPPTPEVPSEPETPTPPTPEVPSEPETPTPPTPEVPSEPETPTPP
TPEVPAEPGKPVPPAKEEPKKPSKPVEQGKVVTPVIEINEKVKAVAPTKKPQSKKSELPETGGEES
TNKGMLFGGLFSILGLALLRRNKKNHKA
000000000000000000000000000000000000000000000000000000000000000000
000000000000000000000000000000000000000000000000000000000000000000
000000000000000000000000000000000000000000000000000000000000000000
000000000000000000000000000000000000000000000000000000000000000000
000000000000000000000000000000000000000000000000000000000000000000
000000000000000000000000000000000000000000000000000000000000000000
000000000000000000000000000000000000000000000000000000000000000000
000000000000000000000000000000000000000000000000000000000000000000

000000000000000000000000000000000000000000000000000000000000000000
000000000000000000000000000000000000000000000000000000000000000000
000000000000000000000000000000000000000000000000000000000000000000
000000000000000000000000000000000000000111111111111110000000000000
000000000000000000000000000000000000000000001111111111111111111110
000000000000000000000000000000000000000000000000000000000000000000
000000000000000000000000000000000000000000000000000000000000000000
0000000000000000000000000000

>P11224 Spike glycoprotein precursor (S glycoprotein) (Peplomer protein) (E2) [Contains: Spike protein S1 (90B); Spike protein S2 (90A)]. [mapped in Murine hepatitis virus strain A59]

MLFVFILFLPSCLGYIGDFRCIQLVNSNGANVSAPSISTETVEVSQGLGTYYVLDRVYLNATLLLT
GYYPVDGSKFRNLALRGTNSVSLSWFQPPYLNQFNDGIFAKVQNLKTSTPSGATAYFPTIVIGSLF
GYTSYTVVIEPYNGVIMASVCQYTICQLPYTDCKPNTNGNKLIGFWHTDVKPPICVLKRNFTLNVN
ADAFYFHFYQHGGTFYAYYADKPSATTFLFSVYIGDILTQYYVLPFICNPTAGSTFAPRYWVTPLV
KRQYLFNFNQKGVITSAVDCASSYTSEIKCKTQSMLPSTGVYELSGYTVQPVGVVYRRVANLPACN
IEEWLTARSVPSPLNWERKTFQNCNFNLSSLLRYVQAESLFCNNIDASKVYGRCFGSISVDKFAVP
RSRQVDLQLGNSGFLQTANYKIDTAATSCQLHYTLPKNNVTINNHNPSSWNRRYGFNDAGVFGKNQ
HDVVYAQQCFTVRSSYCPCAQPDIVSPCTTQTKPKSAFVNVGDHCEGLGVLEDNCGNADPHKGCIC
ANNSFIGWSHDTCLVNDRCQIFANILLNGINSGTTCSTDLQLPNTEVVTGICVKYDLYGITGQGVF
KEVKADYYNSWQTLLYDVNGNLNGFRDLTTNKTYTIRSCYSGRVSAAFHKDAPEPALLYRNINCSY
VFSNNISREENPLNYFDSYLGCVVNADNRTDEALPNCDLRMGAGLCVDYSKSRRAHRSVSTGYRLT
TFEPYTPMLVNDSVQSVDGLYEMQIPTNFTIGHHEEFIQTRSPKVTIDCAAFVCGDNTACRQQLVE
YGSFCVNVNAILNEVNNLLDNMQLQVASALMQGVTISSRLPDGISGPIDDINFSPLLGCIGSTCAE
DGNGPSAIRGRSAIEDLLFDKVKLSDVGFVEAYNNCTGGQEVRDLLCVQSFNGIKVLPPVLSESQI
SGYTTGATAAAMFPPWSAAAGVPFSLSVQYRINGLGVTMNVLSENQKMIASAFNNALGAIQDGFDA
TNSALGKIQSVVNANAEALNNLLNQLSNRFGAISASLQEILTRLEAVEAKAQIDRLINGRLTALNA
YISKQLSDSTLIKVSAAQAIEKVNECVKSQTTRINFCGNGNHILSLVQNAPYGLYFIHFSYVPISF
TTANVSPGLCISGDRGLAPKAGYFVQDDGEWKFTGSSYYYPEPITDKNSVIMSSCAVNYTKAPEVF
LNTSIPNPPDFKEELDKWFKNQTSIAPDLSLDFEKLNVTLLDLTYEMNRIQDAIKKLNESYINLKE
VGTYEMYVKWPWYVWLLIGLAGVAVCVLLFFICCCTGCGSCCFKKCGNCCDEYGGHQDSIVIHNIS
SHED

000000000000000000000000000000000000000000000000000000000000000000
000000000000000000000000000000000000000000000000000000000000000000
000000000000000000000000000000000000000000000000000000000000000000
000000000000000000000000000000000000000000000000000000000000000000
000000000000000000000000000000000000000000000000000000000000000000
000000000000000000000000000000000000000000000000000000000000000000

0000000000000000000000000000000000000000000000000000000000000000000000
0000000000000000000000000000000000000000000000000000000000000000000000
0000000000000000000000000000000000000000000000000000000000000000000000
0000000000000000000000000000000000000000000000000000000000000000000000
0000000000000000000000000000000000000000000000000000000000000000000000
0000000000000000000000000000000000000000000000000000000000000000000000
0000000000000000000000000000000000000000000000000000000001111111111111
0000000000000000000000000000000000000000000000000000000000000000000000
0000000000000000000000000000000000000000000000000000000000000000000000
0000000000000000000000000000000000000000000000000000000000000000000000
0000000000000000000000000000000000000000000000000000000000000000000000
0000000000000000000000000000000000000000000000000000000000000000000000
0000000000000000000000000000000000000000000000000000000000000000000000
0000000000000000000000000000000000000000000000000000000000000000000000
0000

>15675130 hypothetical protein SPy1154 [Streptococcus pyogenes M1 GAS]. [mapped in Streptococcus pyogenes M1 GAS]

MVKKQKRRKIKSMSWARKLLIAVLLILGLALLFNKPIRNTLIARNSNKYQVTKVSKKQIKKNKEAK
STFDFQAVEPVSTESVLQAQMAAQQLPVIGGIAIPELGINLPIFKGLGNTELIYGAGTMKEEQVMG
GENNYSLASHHIFGITGSSQMLFSPLERAQNGMSIYLTDKEKIYEYIIKDVFTVAPERVDVIDDTA
GLKEVTLVTCTDIEATERIIVKGELKTEYDFDKAPADVLKAFNHSYNQVST

0000000000000000000000000000000000000000000000000000000000000000000000
0000000000000111111111111111111110000000000000000000000000000000000000
0000000000000000000000000000000000000000000000000000000000000000000000
000000000000000000000000000000000000000000000000000000

>Q46221 Iota toxin component Ib precursor. [mapped in Clostridium perfringens]

MNIQIKNVFSFLTLTAMISQTLSYNVYAQTTTQNDTNQKEEITNENTLSSNGLMGYYFADEHFKDL
ELMAPIKNGDLKFEEKKVDKLLTEDNSSIKSIRWTGRIIPSEDGEYILSTDRNDVLMQINAKGDIA
KTLKVNMKKGQAYNIRIEIQDKNLGSIDNLSVPKLYWELNGNKTVIPEENLFFRDYSKIDENDPFI
PNNNFFDVRFFSAAWEDEDLDTDNDNIPDAYEKNGYTIKDSIAVKWNDSFAEQGYKKYVSSYLESN
TAGDPYTDYQKASGSIDKAIKLEARDPLVAAYPVVGVGMENLIISTNEHASSDQGKTVSRATTNSK
TDANTVGVSISAGYQNGFTGNITTSYSHTTDNSTAVQDSNGESWNTGLSINKGESAYINANVRYYN
TGTAPMYKVTPTTNLVLDGETLATIKAQDNQIGNNLSPNETYPKKGLSPLALNTMDQFNARLIPIN
YDQLKKLDSGKQIKLETTQVSGNYGTKNSQGQIITEGNSWSNYISQIDSVSASIILDTGSQTFERR
VAAKEQGNPEDKTPEITIGEAIKKAFSATKNGELLYFNGIPIDESCVELIFDDNTSEIIKEQLKYL
DDKKIYNVKLERGMNILIKVPSYFTNFDEYNNFPASWSNIDTKNQDGLQSVANKLSGETKIIIPMS
KLKPYKRYVFSGYSKDPSTSNSITVNIKSKEQKTDYLVPEKDYTKFSYEFETTGKDSSDIEITLTS

SGVIFLDNLSITELNSTPEILKEPEIKVPSDQEILDAHNKYYADIKLDTNTGNTYIDGIYFEPTQT
NKEALDYIQKYRVEATLQYSGFKDIGTKDKEIRNYLGDQNQPKTNYINFRSYFTSGENVMTYKKLR
IYAVTPDNRELLVLSVN
000000000000000000000000000000000000000000000000000000000000000000
000000000000000000000000000000000000000000000000000000000000000000
000000000000000000000000000000000000000000000000000000000000000000
000000000000000000000000000000000000000000000000000000000000000000
000000000000000000000000000000000000000000000000000000000000000000
000000000000000000000000000000000000000000000000000000000000000000
000000000000000000000000000000000000000000000000000000000000000000
000000000000000000000000000000000000000000000000000000000000000000
000000000000000000000000000000000000000000000000000000000000000000
000000000000000000000000000000000000000000000000000000000000000000
000000000000000000000000000000000000000000000000000000000000000000
000000000000000000000000000000000000000000000000000000000000000000
000000000000000000000000000000000000000000000000001111111111111111
11111111000000000
>13559809 nucleocapsid protein [Nipah virus]. [mapped in Nipah virus]
MSDIFEEAASFRSYQSKLGRDGRASAATATLTTKIRIFVPATNSPELRWELTLFALDVIRSPSAAE
SMKVGAAFTLISMYSERPGALIRSLLNDPDIEAVIIDVGSMVNGIPVMERRGDKAQEEMEGLMRIL
KTARDSSKGKTPFVDSRAYGLRITDMSTLVSAVITIEAQIWILIAKAVTAPDTAEESETRRWAKYV
QQKRVNPFFALTQQWLTEMRNLLSQSLSVRKFMVEILIEVKKGGSAKGRAVEIISDIGNYVEETGM
AGFFATIRFGLETRYPALALNEFQSDLNTIKSLMLLYREIGPRAPYMVLLEESIQTKFAPGGYPLL
WSFAMGVATTIDRSMGALNINRGYLEPMYFRLGQKSARHHAGGIDQNMANRLGLSSDQVAELAAAV
QETSAGRQESNVQAREAKFAAGGVLIGGSDQDIDEGEEPIEQSGRQSVTFKREMSISSLANSVPSS
SVSTSGGTRLTNSLLNLRSRLAAKAAKEAASSNATDDPAISNRTQGESEKKNNQDLKPAQNDLDFV
RADV
000000000000000000000000000000000000000000000000000000000000111111
000000000000000000000000000000000000000000000000000000000000000000
000000000000000000000000000000000000000000000000000000000000000000
000000000000000000000000000000000000000000000000000000000000000000
000000000000000000000000000000000000000000000000000011111111000000
000000000000000000000000000000000000000000000000000000000000000000
000000000000000000000000000000000000000000000000000000000000000000
000000000000000000000000000000000000000011111110000000000000000000
0000
>P27662 Spike glycoprotein precursor. [mapped in Viral hemorrhagic septicemia virus (STRAIN 07-71)]

MEWNTFFLVILIIIIKSTTPQITQRPPVENISTYHADWDTPLYTHPSNCRDDSFVPIRPAQLRCPH
EFEDINKGLVSVPTRIIHLPLSVTSVSAVASGHYLHRVTYRVTCSTSFFGGQTIEKTILEAKLSRQ
EATDEASKDHEYPFFPEPSCIWMKNNVHKDITHYYKTPKTVSVDLYSRKFLNPDFIEGVCTTSPCQ
THWQGVYWVGATPKAHCPTSETLEGHLFTRTHDHRVVKAIVAGHHPWGLTMACTVTFCGTEWIKTD
LGDLIQVTGPGGTRKLTPNKCVNTDIQMRGATDDFSYLNHLITNMAQRTECLDAHSDITASGKVSS
FLLSKFRPSHPGPGKAHYLLDGQIMRGDCDYEAVVSINYNRAQYKTMNNTWKSWKRVDNNTDGYDG
MIFGDKLIIPDIEKYQSVYDSGMLVQRNLVEVPHLSIVFVSNTSDLSTNHIHTNLIPSDWSFNWSL
WPSLSGMGVVGGAFLLLVLCCCCKASPPIPNYGIPMQQFSRSQTV
000000000000000000000000000000000000000000000000000000000000000000
000000000000000000000000000000000000000000000000000000000000000000
000000000000000000000000000000000000000000000000000000000000000000
000000000000000000000000000000000000000000000000000000000000000000
000000000000000000000000000000000000000000000000000000000000000000
000000000000000000000000000000000000000000000000000000000000000000
001111111111111110000000000000000000000000000000000000000000000000
000000000000000000000000000000000000000000000
>P16567 Major outer membrane porin precursor (MOMP). [mapped in Chlamydophila abortus]
MKKLLKSALLFAATGSALSLQALPVGNPAEPSLLIDGTMWEGASGDPCDPCSTWCDAISIRAGYYG
DYVFDRVLKVDVNKTITGMGAVPTGTAAANYKTPTDRPNIAYGKHLQDAEWFTNAAFLALNIWDRF
DIFCTLGASNGYFKASSAAFNLVGLIGVKGSSIAADQLPNVGITQGIVEFYTDTTFSWSVGARGAL
WECGCATLGAEFQYAQSNPKIEMLNVVSSPAQFVVHKPRGYKGTAFPLPLTAGTDQATDTKSATIK
YHEWQVGLALSYRLNMLVPYISVNWSRATFDADAIRIAQPKLAAAVLNLTTWNPTLLGEATALDTS
NKFADFLQIASIQINKMKSRKACGVAVGATLIDADKWSITGEARLINERAAHMNAQFRF
000000000000000000000000000000000000000000000000000000000000000000
000000000000000000000011111111111100000000000000000000000000000000
000000000000000000000000000001111111111000000000000000000000000000
000000000000000000000000000000000000000000000000000000000000000000
000000000000000000000000000000000000000000011111111111111000000000
00000000000000000000000000000000000000000000000000000000000
>138881 Non-capsid protein NS-1 (Non-structural protein NS1) (NCVP1). [mapped in Human parvovirus B19]
MELFRGVLQVSSNVLDCANDNWWCSLLDLDTSDWEPLTHTNRLMAIYLSSVASKLDFTGGPLAGCL
YFFQVECNKFEEGYHIHVVTGGPGLNPRNLTVCVEGLFNNVLYHLVTENVKLKFLPGMTTKGKYFR
DGEQFIENYLMKKIPLNVVWCVTNIDGYIDTCISATFRRGACHAKKPRITTAINDTSSDAGESSGT
GAEVVPFNGKGTKASIKFQTMVNWLCENRVFTEDKWKLVDFNQYTLLSSSHSGSFQIQSALKLAIY
KATNLVPTSTFLLHTDFEQVMCIKDNKIVKLLLCQNYDPLLVGQHVLKWIDKKCGKKNTLWFYGPP
STGKTNLAMAIAKSVPVYGMVNWNNENFPFNDVAGKSLVVWDEGIIKSTIVEAAKAILGGQPTRVD

QKMRGSVAVPGVPVVITSNGDITFVVSGNTTTTVHAKALKERMVKLNFTVRCSPDMGLLTEADVQQ
WLTWCNAQSWDHYENWAINYTFDFPGINADALHPDLQTTPIVTDTSISSSGGESSEELSESSFFNL
ITPGAWNTETPRSSTPIPGTSSGESFVGSPVSSEVVAASWEEAFYTPLADQFRELLVGVDYVWDGV
RGLPVCCVQHINNSGGGLGLCPHCINVGAWYNGWKFREFTPDLVRCSCHVGASNPFSVLTCKKCAY
LSGLQSFVDYE
000000000000000000000000000000000000000000000000000000000000000000
000000000000000000000000000000000000000000000000000000000000000000
000000000000000000000000000000000000000000000000000000000000000000
000000000000000000000000000000000000000000000000000000000000000000
000000000000000000000000000000000000000000000000000000000000000000
000000000000000000000000000000000000000000000000000000000000000000
000000000000000000000000000000000000000000000000000000000000000000
000000000000000000000000000000000000000000000000001111111111111111
110000000000000000000000000000000000000000000000000000000000000000
000000000000000000000000000000000000000000000000000000000000000000
00000000000
>P07946 Spike glycoprotein precursor (S glycoprotein) (Peplomer protein) (E2). [mapped in Porcine transmissible gastroenteritis coronavirus (STRAIN PURDUE)]
MKKLFVVLVVMPLIYGDNFPCSKLTNRTIGNQWNLIETFLLNYSSRLPPNSDVVLGDYFPTVQPWF
NCIRNNSNDLYVTLENLKALYWDYATENITWNHRQRLNVVVNGYPYSITVTTTRNFNSAEGAIICI
CKGSPPTTTTESSLTCNWGSECRLNHKFPICPSNSEANCGNMLYGLQWFADEVVAYLHGASYRISF
ENQWSGTVTFGDMRATTLEVAGTLVDLWWFNPVYDVSYYRVNNKNGTTVVSNCTDQCASYVANVFT
TQPGGFIPSDFSFNNWFLLTNSSTLVSGKLVTKQPLLVNCLWPVPSFEEAASTFCFEGAGFDQCNG
AVLNNTVDVIRFNLNFTTNVQSGKGATVFSLNTTGGVTLEISCYTVSDSSFFSYGEIPFGVTDGPR
YCYVHYNGTALKYLGTLPPSVKEIAISKWGHFYINGYNFFSTFPIDCISFNLTTGDSDVFWTIAYT
SYTEALVQVENTAITKVTYCNSHVNNIKCSQITANLNNGFYPVSSSEVGLVNKSVVLLPSFYTHTI
VNITIGLGMKRSGYGQPIASTLSNITLPMQDHNTDVYCIRSDQFSVYVHSTCKSALWDNIFKRNCT
DVLDATAVIKTGTCPFSFDKLNNYLTFNKFCLSLSPVGANCKFDVAARTRTNEQVVRSLYVIYEEG
DNIVGVPSDNSGVHDLSVLHLDSCTDYNIYGRTGVGIIRQTNRTLLSGLYYTSLSGDLLGFKNVSD
GVIYSVTPCDVSAQAAVIDGTIVGAITSINSELLGLTHWTTTPNFYYYSIYNYTNDRTRGTAIDSN
DVDCEPVITYSNIGVCKNGAFVFINVTHSDGDVQPISTGNVTIPTNFTISVQVEYIQVYTTPVSID
CSRYVCNGNPRCNKLLTQYVSACQTIEQALAMGARLENMEVDSMLFVSENALKLASVEAFNSSETL
DPIYKEWPNIGGSWLEGLKYILPSHNSKRKYRSAIEDLLFDKVVTSGLGTVDEDYKRCTGGYDIAD
LVCAQYYNGIMVLPGVANADKMTMYTASLAGGITLGALGGGAVAIPFAVAVQARLNYVALQTDVLN
KNQQILASAFNQAIGNITQSFGKVNDAIHQTSRGLATVAKALAKVQDVVNIQGQALSHLTVQLQNN
FQAISSSISDIYNRLDELSADAQVDRLITGRLTALNAFVSQTLTRQAEVRASRQLAKDKVNECVRS
QSQRFGFCGNGTHLFSLANAAPNGMIFFHTVLLPTAYETVTAWPGICASDGDRTFGLVVKDVQLTL

FRNLDDKFYLTPRTMYQPRVATSSDFVQIEGCDVLFVNATVSDLPSIIPDYIDINQTVQDILENFR
PNWTVPELTFDIFNATYLNLTGEIDDLEFRSEKLHNTTVELAILIDNINNTLVNLEWLNRIETYVK
WPWYVWLLIGLVVIFCIPLLLFCCCSTGCCGCIGCLGSCCHSICSRRQFENYEPIEKVHVH
000000000000000000000000000000000000000000000000000000000000000000
000000000000000000000000000000000000000000000000000000000000000000
000000000000000000000000000000000000000000000000000000000000000000
000000000000000000000000000000000000000000000000000000000000000000
000000000000000000000000000000000000000000000000000000000000000000
000000000000000000000000000000000000000000111111111111111111111111
110000000000000000000000000000000000000000000000000000000000000000
000000000000000000000000000000000000000000000000000000000000000000
000000000000000000000000000000000000000000000000000000000000000000
000000000000000000000000000000000000000000000000000000000000000000
000000000000000000000000000000000000000000000000000000000000000000
000000000000000000000000000000000000000000000000000000000000000000
000000000000000000000000000000000000000000000000000000000000000000
000000000000000000000000000000000000000000000000000000000000000000
000000000000000000000000000000000000000000000000000000000000000000
000000000000000000000000000000000000000000000000000000000000000000
000000000000000000000000000000000000000000000000000000000000000000
000000000000000000000000000000000000000000000000000000000000000000
000000000000000000000000000000000000000000000000000000000000000000
000000000000000000000000000000000000000000000000000000000000000000
000000000000000000000000000000000000000000000000000000000000000000
0000000000000000000000000000000000000000000000000000000000000
>P09592 Structural polyprotein (p130) [Contains: Capsid protein (Coat protein) (C); p62 (E3/E2); E3 protein (Spike glycoprotein E3); E2 envelope glycoprotein (Spike glycoprotein E2); 6K protein; E1 envelope glycoprotein (Spike glycoprotein E1)]. [mapped in Venezuelan equine encephalitis virus (STRAIN TRINIDAD DONKEY)]
MFPFQPMYPMQPMPYRNPFAAPRRPWFPRTDPFLAMQVQELTRSMANLTFKQRRDAPPEGPSAKKP
KKEASQKQKGGGQGKKKKNQGKKKAKTGPPNPKAQNGNKKKTNKKPGKRQRMVMKLESDKTFPIML
EGKINGYACVVGGKLFRPMHVEGKIDNDVLAALKTKKASKYDLEYADVPQNMRADTFKYTHEKPQG
YYSWHHGAVQYENGRFTVPKGVGAKGDSGRPILDNQGRVVAIVLGGVNEGSRTALSVVMWNEKGVT
VKYTPENCEQWSLVTTMCLLANVTFPCAQPPICYDRKPAETLAMLSVNVDNPGYDELLEAAVKCPG
RKRRSTEELFKEYKLTRPYMARCIRCAVGSCHSPIAIEAVKSDGHDGYVRLQTSSQYGLDSSGNLK
GRTMRYDMHGTIKEIPLHQVSLHTSRPCHIVDGHGYFLLARCPAGDSITMEFKKDSVTHSCSVPYE
VKFNPVGRELYTHPPEHGVEQACQVYAHDAQNRGAYVEMHLPGSEVDSSLVSLSGSSVTVTPPVGT

SALVECECGGTKISETINKTKQFSQCTKKEQCRAYRLQNDKWVYNSDKLPKAAGATLKGKLHVPFL
LADGKCTVPLAPEPMITFGFRSVSLKLHPKNPTYLTTRQLADEPHYTHELISEPAVRNFTVTEKGW
EFVWGNHPPKRFWAQETAPGNPHGLPHEVITHYYHRYPMSTILGLSICAAIATVSVAASTWLFCRS
RVACLTPYRLTPNARIPFCLAVLCCARTARAETTWESLDHLWNNNQQMFWIQLLIPLAALIVVTRL
LRCVCCVVPFLVMAGAAAGAYEHATTMPSQAGISYNTIVNRAGYAPLPISITPTKIKLIPTVNLEY
VTCHYKTGMDSPAIKCCGSQECTPTYRPDEQCKVFTGVYPFMWGGAYCFCDTENTQVSKAYVMKSD
DCLADHAEAYKAHTASVQAFLNITVGEHSIVTTVYVNGETPVNFNGVKLTAGPLSTAWTPFDRKIV
QYAGEIYNYDFPEYGAGQPGAFGDIQSRTVSSSDLYANTNLVLQRPKAGAIHVPYTQAPSGFEQWK
KDKAPSLKFTAPFGCEIYTNPIRAENCAVGSIPLAFDIPDALFTRVSETPTLSAAECTLNECVYSS
DFGGIATVKYSASKSGKCAVHVPSGTATLKEAAVELTEQGSATIHFSTANIHPEFRLQICTSYVTC
KGDCHPPKDHIVTHPQYHAQTFTAAVSKTAWTWLTSLLGGSAVIIIIGLVLATIVAMYVLTNQKHN
000000000000000000000000000000000000000000000000000000000000000000
000000000000000000000000000000000000000000000000000000000000000000
000000000000000000000000000000000000000000000000000000000000000000
000000000000000000000000000000000000000000000000000000000000000000
000000000000000000000000000000000000000000000000000000000000000000
000011111111111111111111111110000000000000000000000000000000000000
000000000000000000000000000000000000000000000000000000000000000000
000000000000000000000000000000000000000000000000000000000000000000
000000000000000000000000000000000000000000000000000000000000000000
000000000000000000000000000000000000000000000000000000000000000000
000000000000000000000000000000000000000000000000000000000000000000
000000000000000000000000000000000000000000000000000000000000000000
000000000000000000000000000000000000000000000000000000000000000000
000000000000000000000000000000000000000000000000000000000000000000
000000000000000000000000000000000000000000000000000000000000000000
000000000000000000000000000000000000000000000000000000000000000000
000000000000000000000000000000000000000000000000000000000000000000
000000000000000000000000000000000000000000000000000000000000000000
000000000000000000000000000000000000000000000000000000000000000000
>13621681 metal binding protein of ABC transporter (lipoprotein) [Streptococcus pyogenes M1 GAS]. [mapped in Streptococcus pyogenes M1 GAS]
MGKRMSLILGAFLSVFLLVACSSTGTKTAKSDKLKVVATNSIIADMTKAIAGDKIDLHSIVPIGQD
PHEYEPLPEDVEKTSNADVIFYNGINLEDGGQAWFTKLVKNAQKTKNKDYFAVSDGIDVIYLEGAS
EKGKEDPHAWLNLENGIIYSKNIAKQLIAKDPKNKETYEKNLKAYVAKLEKLDKEAKSKFDAIAEN
KKLIVTSEGCFKYFSKAYGVPSAYIWEINTEEEGTPDQISSLIEKLKVIKPSALFVESSVDRRPME
TVSKDSGIPIYSEIFTDSIAKKGKPGDSYYAMMKWNLDKISEGLAK

000000000000000000000000000000000000000000000000000000000000000000
000000000000000000000000000000000000000000000000000000000000000000
000000000000000000000000111111111111111110000000000000000000000000
000000000000000000000000001111111111111111111000000000001111111111
1111110000000000000000000000000000000000000000
>P03308 Genome polyprotein [Contains: Leader protease (P20A); Coat protein VP4; Coat protein VP3; Coat protein VP2; Coat protein VP1; Core protein X; Core protein p14; Core protein p41; Core protein p19; Genome-linked protein VPg1; Genome-linked protein VPg2; Genome-linked protein VPg3; Picornain 3C (Protease 3C) (P3C); RNA-directed RNA polymerase]. [mapped in Foot-and-mouth disease virus]
MNTTNCFIALVHAIREIRAFFLSRATGKMEFTLYNGERKTFYSRPNNHDNCWLNTILQLFRYVDEP
FFDWVYNSPENLTLAAIKQLEELTGLELHEGGPPALVIWNIKHLLQTGIGTASRPARCMVDGTNMC
LADFHAGIFLKEQEHAVFACVTSNGWYAIDDEDFYPWTPDPSDVLVFVPYDQEPLNGGWKANVQRK
LKGAGQSSPATGSQNQSGNTGSIINNYYMQQYQNSMDTQLGDNAISGGSNEGSTDTTSTHTTNTQN
NDWFSKLASSAFTGLFGALLADKKTEETTLLEDRILTTRNGHTTSTTQSSVGVTYGYSTEEDHVAG
PNTSGLETRVVQAERFFKKFLFDWTPDKPFGHRTKLELPTDHHGVFGHLVDSYAYMRNGWDVEVSA
VGNQFNGGCLLVAMVPEWKTFDTREEYQLTLFPHQFISPRTNMTAHITVPYLGVNRYDQYKKHKPW
TLVIMVLSPLTVSNTAATQIKVYANIAPTYVHVAGELPSKVGIFPVACSDGYGGLVTTDPKTADPV
YGKEYNPPKTNYPRRFTNLLDVAEACPTFLCFDDGKPYVVTRTDDTRLLAKFDVSLAAKHMSNTYL
SGIAQYYTQYSGTINLHFMFTGSTDSKARYMVAYIPPGVETPPETPEGAAHCIHAEWDTGLNSKFT
FSIPYVSAADYAYTASDTAETTNVQGWVCIYQITHGKAEDDTLVVSASAGKDFELRLPIDPRSQTT
ATGESADPVTTTVENYGGETQVQRRHHTDVSFIMDRFVKIKSLNPTHVIDLMQTHQHGLVGALLRA
ATYYFSDLEIVVRHDGNLTWVPNGAPEAALSNTGNPTAYNKAPFTRLALPYTAPHRVLATVYNGTN
KYSASGSGVRGDFGSLAPRVARQLPASFNYGAIKAETIHELLVRMKRAELYCPRPLLAIEVSSQDR
HKQKIIAPGKQLLNFDLLKLAGDVESNPRPFFFADVRSNFSKLVDTINQMQEDMSTKHGPDFNRLV
SAFEELATGVKAIRTGLDEAKPWYKLIKLLSRLSCMAAVAARTKDPVLVAIMLADTGLEILDSTFV
VKKISDSLSSLFHVPAPVFSFGAPVLLAGLVKVASSFLRSTPEDLERAEKQLKARDINDIFAILKN
GEWLVKLILAIRDWIKAWIASEEKFVTMTDLVLGILEKQRDLNDPSKYKEAKEWLDNARQACLKSG
NVHIANLCKVVAPAPSKSRPEPVVVCLRGKSGQGKSFLANVLAQAISTHFTGRTDSVWYCPPDPDH
FDGYNQQTVVVMDDLGQNPDGKDFKYFAQMVSTTGFIPPMASLEDKGKPFNSKVIIATTNLYSGFT
PRTMVCPDALNRRFHFDIDVSAKDGYKINNKLDIVKALEDTHTNPVAMFQYDCALLNGMAVEMKRM
QQDMFKPQPPLQNVYQLVQEVIERVELHEKVSSHPIFKQISIPSQKSVLYFLIEKGQHEAAIEFFE
GMVHDSIKEELRPLIQQTSFVKRAFKRLKENFEIVALCLTLLANIVIMIRETRKRQKMVDDAVNEY
IEKANITTDDTTLDEAEKNPLETSGASTVGFRERTLTGQRACNDVNSEPARPAEEQPQAEGPYTGP
LERQRPLKVRAKLPQQEGPYAGPLERQKPLKVKAKAPVVKEGPYEGPVKKPVALKVKAKNLIVTES
GAPPTDLQKMVMGNTKPVELILDGKTVAICCATGVFGTAYLVPRHLFAEKYDKIMLDGRAMTDSDY
RVFEFEIKVKGQDMLSDAALMVLHRGNRVRDITKHFRDTARMKKGTPVVGVVNNADVGRLIFSGEA

LTYKDIVVCMDGDTMPSLFAYKAATKAGYCGGAVLAKDGADTFIVGTHSAGGNGVGYCSCVSKSML
LRMKAHVDPEPQHEGLIVDTRDVEERVHVMRKTKLAPTVAHGVFNPEFGPAALSNKDPRLNEGVVL
DEVIFSKHKGDTKMSAEDKALFRACAADYASRLHSVLGTANAPLSIYEAIKGVDGLDAMESDTAPG
LPWAFQGKRRGALIDFENGTVGPEVEAALKLMEKREYKFVCQTFLKDEIRPMEKVRAGKTRIVDVL
PVEHILYTRMMIGRFCAQMHSNNGPQIGSAVGCNPDVDWQRFGTHFAQYRNVWDVDYSAFDTNHCS
DAMNIMFEEVFRTDFGFHPNAEWILKTLVNTEHAYENKRITVEGGMPSDCSATGIINTILNNIYVL
YALRRHYEGVELDTYTMISYGDDIVVASDYDLDFEALKPHFKSLGQTITPADKSDKGFVLGHSITD
VTFLKRHFHIDYGTGFYKPVMASKTLEAILSFARRGTIQEKLTSVAGLAVHSGPDEYRRLFEPFQG
LFEIPSYRSLYLRWVNAVCGDA
000000000000000000000000000000000000000000000000000000000000000000
000000000000000000000000000000000000000000000000000000000000000000
000000000000000000000000000000000000000000000000000000000000000000
000000000000000000000000000000000000000000000000000000000000000000
000000000000000000000000000000000000000000000000000000000000000000
000000000000000000000000000000000000000000000000000000000000000000
000000000000000000000000000000000000000000000000000000000000000000
000000000000000000000000000000000000000000000000000000000000000000
000000000000000000000000000000000000000000000000000000000000000000
000000000000000000000000000000000000000000000000000000000000000000
000000000000000000000000000000000000000000000000000000000000000000
000000000000000000000000000000000000000000000000000000000000000000
000000000000000000000000000000000000000000000000000000000000000000
000001111111111111111111000000000000000000000000000000000000000000
000000000000000000000000000000000000000000000000000000000000000000
000000000000000000000000000000000000000000000000000000000000000000
000000000000000000000000000000000000000000000000000000000000000000
000000000000000000000000000000000000000000000000000000000000000000
000000000000000000000000000000000000000000000000000000000000000000
000000000000000000000000000000000000000000000000000000000000000000
000000000000000000000000000000000000000000000000000000000000000000
000000000000000000000000000000000000000000000000000000000000000000
000000000000000000000000000000000000000000000000000000000000000000
000000000000000000000000000000000000000000000000000000000000000000
000000000000000000000000000000000000000000000000000000000000000000
000000000000000000000000000000000000000000000000000000000000000000
000000000000000000000000000000000000000000000000000000000000000000
000000000000000000000000000000000000000000000000000000000000000000
000000000000000000000000000000000000000000000000000000000000000000

000000000000000000000000000000000000000000000000000000000000000000
000000000000000000000000000000000000000000000000000000000000000000
000000000000000000000000000000000000000000000000000000000000000000
000000000000000000000000000000000000000000000000000000000000000000
000000000000000000000000000000000000000000000000000000000000000000
000000000000000000000000000000000000000000000000000000000000000000
0000000000000000000000
>Q05320 Envelope glycoprotein precursor (GP1,2) (GP) [Contains: GP1; GP2; GP2-delta]. [mapped in Ebola virus strain Zaire Mayinga]
MGVTGILQLPRDRFKRTSFFLWVIILFQRTFSIPLGVIHNSTLQVSDVDKLVCRDKLSSTNQLRSV
GLNLEGNGVATDVPSATKRWGFRSGVPPKVVNYEAGEWAENCYNLEIKKPDGSECLPAAPDGIRGF
PRCRYVHKVSGTGPCAGDFAFHKEGAFFLYDRLASTVIYRGTTFAEGVVAFLILPQAKKDFFSSHP
LREPVNATEDPSSGYYSTTIRYQATGFGTNETEYLFEVDNLTYVQLESRFTPQFLLQLNETIYTSG
KRSNTTGKLIWKVNPEIDTTIGEWAFWETKKNLTRKIRSEELSFTVVSNGAKNISGQSPARTSSDP
GTNTTTEDHKIMASENSSAMVQVHSQGREAAVSHLTTLATISTSPQSLTTKPGPDNSTHNTPVYKL
DISEATQVEQHHRRTDNDSTASDTPSATTAAGPPKAENTNTSKSTDFLDPATTTSPQNHSETAGNN
NTHHQDTGEESASSGKLGLITNTIAGVAGLITGGRRTRREAIVNAQPKCNPNLHYWTTQDEGAAIG
LAWIPYFGPAAEGIYIEGLMHNQDGLICGLRQLANETTQALQLFLRATTELRTFSILNRKAIDFLL
QRWGGTCHILGPDCCIEPHDWTKNITDKIDQIIHDFVDKTLPDQGDNDNWWTGWRQWIPAGIGVTG
VIIAVIALFCICKFVF
000000000000000000000000000000000000000000000000000000000000000000
000000000000000000000000000000000000000000000000000000000000000000
000000000000000000000000000000000000000000000000000000000000000000
000000000000000000000000000000000000000000000000000000000000000000
000000000000000000000000000000000000000000000000000000000000000000
000000000000000000000000000000000000000000000000000000000000001111
111110000000000000000000000000000000000000000000000000000000000000
000000000000000000000000000000000000000000000000000000000000000000
000000000000000000000000000000000000000000000000000000000000000000
000000000000000000000000000000000000000000000000000000000000000000
0000000000000000
>P12379 M protein, serotype 24 precursor. [mapped in Streptococcus pyogenes]
MTKNNTNRHYSLRKLKTGTASVAVALTVLGAGLVVNTNEVSAVATRSQTDTLEKVQERADKFEIEN
NTLKLKNSDLSFNNKALKDHNDELTEELSNAKEKLRKNDKSLSEKASKIQELEARKADLEKALEGA
MNFSTADSAKIKTLEAEKAALAARKADLEKALEGAMNFSTADSAKIKTLEAEKAALEARQAELEKA
LEGAMNFSTADSAKIKTLEAEKAALAARKADLEKALEGAMNFSTADSAKIKTLEAEKAALEARQAE
LEKALEGAMNFSTADSAKIKTLEAEKAALEAEKADLEHQSQVLNANRQSLRRDLDASREAKKQLEA

EHQKLEEQNKISEASRQSLRRDLDASREAKKQLEAEHQKLEEQNKISEASRQSLRRDLDASREAKK
QVEKALEEANSKLAALEKLNKELEESKKLTEKEKAELQAKLEAEAKALKEKLAKQAEELAKLRAGK
ASDSQTPDAKPGNKAVPGKGQAPQAGTKPNQNKAPMKETKRQLPSTGETANPFFTAAALTVMATAG
VAAVVKRKEEN
111111111111111111111111111111111111111111111111111111111111
110000000000000000000000000000000000000000000000000000000000
011111111111111111111111111111110000000000000000000000000000
000000000000000000000000000000000000000000000000000000000000
000000000000000000000000000000000000000000000000000000000000
000000000000000000000000000000000000000000000000111111111111
111110000000000000000000000000000000000000000000000000000000
000000000000000000000000000000000000000000000000000000000000
00000000000
>P05769 Genome polyprotein [Contains: Capsid protein C (Core protein); Envelope protein M (Matrix protein); Major envelope protein E; Non-structural protein 1 (NS1); Non-structural protein 2A (NS2A); Flavivirin protease NS2B regulatory subunit; Flavivirin protease NS3 catalytic subunit ; Non-structural protein 4A (NS4A); Non-structural protein 4B (NS4B); RNA-directed RNA polymerase (NS5)]. [mapped in Murray Valley encephalitis virus]
MSKKPGGPGKPRVVNMLKRGIPRVFPLVGVKRVVMNLLDGRGPIRFVLALLAFFRFTALAPTKALM
RRWKSVNKTTAMKHLTSFKKELGTLIDVVNKRGKKQKKRGGSETSVLMLIFMLIGFAAALKLSTFQ
GKIMMTVNATDIADVIAIPTPKGPNQCWIRAIDIGFMCDDTITYECPKLESGNDPEDIDCWCDKQA
VYVNYGRCTRARHSKRSRRSITVQTHGESTLVNKKDAWLDSTKATRYLTKTENWIIRNPGYALVAV
VLGWMLGSNTGQKVIFTVLLLLVAPAYSFNCLGMSSRDFIEGASGATWVDLVLEGDSCITIMAADK
PTLDIRMMNIEATNLALVRNYCYAATVSDVSTVSNCPTTGESHNTKRADHNYLCKRGVTDRGWGNG
CGLFGKGSIDTCAKFTCSNSAAGRLILPEDIKYEVGVFVHGSTDSTSHGNYSTQIGANQAVRFTIS
PNAPAITAKMGDYGEVTVECEPRSGLNTEAYYVMTIGTKHFLVHREWFNDLLLPWTSPASTEWRNR
EILVEFEEPHATKQSVVALGSQEGALHQALAGAIPVEFSSSTLKLTSGHLKCRVKMEKLKLKGTTY
GMCTEKFTFSKNPADTGHGTVVLELQYTGSDGPCKIPISSVASLNDMTPVGRMVTANPYVASSTAN
AKVLVEIEPPFGDSYIVVGRGDKQINHHWHKEGSSIGKAFSTTLKGAQRLAALGDTAWDFGSVGGV
FNSIGKAVHQVFGGAFRTLFGGMSWISPGLLGALLLWMGVNARDKSIALAFLATGGVLLFLATNVH
ADTGCAIDITRRELKCGSGIFIHNDVEAWIDRYKYLPETPKQLAKVVENAHKSGICGIRSVNRFEH
QMWESVRDELNALLKENAIDLSVVVEKQKGMYRAAPNRLRLTVEELDIGWKAWGKSLLFAAELANS
TFVVDGPETAECPNSKRAWNSFEIEDFGFGITSTRGWLKLREENTSECDSTIIGTAVKGNHAVHSD
LSYWIESGLNGTWKLERAIFGEVKSCTWPETHTLWGDAVEETELIIPVTLAGPRSKHNRREGYKVQ
VQGPWDEEDIKLDFDYCPGTTVTVSEHCGKRGPSVRTTTDSGKLVTDWCCRSCTLPPLRFTTASGC
WYGMEIRPMKHDESTLVKSRVQAFNGDMIDPFQLGLLVMFLATQEVLRKRWTARLTLPAAVGALLV

LLLGGITYTDLVRYLILVGSAFAESNNGGDVIHLALIAVFKVQPAFLVASLTRSRWTNQENLVLVL
GAAFFQMAASDLELTIPGLLNSAATAWMVLRAMAFPSTSAIAMPMLAMLAPGMRMLHLDTYRIVLL
LIGICSLLNERRRSVEKKKGAVLIGLALTSTGYFSPTIMAAGLMICNPNKKRGWPATEVLTAVGLM
FAIVGGLAELDIDSMSVPFTIAGLMLVSYVISGKATDMWLERAADVSWEAGAAITGTSERLDVQLD
DDGDFHLLNDPGVPWKIWVLRMTCLSVAAITPRAILPSAFGYWLTLKYTKRGGVFWDTPSPKVYPK
GDTTPGVYRIMARGILGRYQAGVGVMHEGVFHTLWHTTRGAAIMSGEGRLTPYWGNVKEDRVTYGG
PWKLDQKWNGVDDVQMIVVEPGKPAINVQTKPGIFKTAHGEIGAVSLDYPIGTSGSPIVNSNGEII
GLYGNGVILGNGAYVSAIVQGERVEEPVPEAYNPEMLKKRQLTVLDLHPGAGKTRRILPQIIKDAI
QKRLRTAVLAPTRVVAAEMAEALRGLPVRYLTPAVQREHSGNEIVDVMCHATLTHRLMSPLRVPNY
NLFVMDEAHFTDPASIAARGYIATRVEAGEAAAIFMTATPPGTSDPFPDTNSPVHDVSSEIPDRAW
SSGFEWITDYAGKTVWFVASVKMSNEIAQCLQRAGKRVIQLNRKSYDTEYPKCKNGDWDFVITTDI
SEMGANFGASRVIDCRKSVKPTILDEGEGRVILSVPSAITSASAAQRRGRVGRNPSQIGDEYHYGG
GTSEDDTMLAHWTEAKILLDNIHLPNGLVAQLYGPERDKTYTMDGEYRLRGEERKTFLELIKTADL
PVWLAYKVASNGIQYNDRKWCFDGPRSNIILEDNNEVEIITRIGERKVLKPRWLDARVYSDHQSLK
WFKDFAAGKRSAIGFFEVLGRMPEHFAGKTREALDTMYLVATSEKGGKAHRMALEELPDALETITL
IAALGVMTAGFFLLMMQRKGIGKLGLGALVLVVATFFLWMSDVSGTKIAGVLLLALLMMVVLIPEP
EKQRSQTDNQLAVFLICVLLVVGLVAANEYGMLERTKTDIRNLFGKSLIEENEVHIPPFDFFTLDL
KPATAWALYGGSTVVLTPLIKHLVTSQYVTTSLASINAQAGSLFTLPKGIPFTDFDLSVALVFLGC
WGQVTLTTLIMATILVTLHYGYLLPGWQAEALRAAQKRTAAGIMKNAVVDGIVATDVPELERTTPQ
MQKRLGQILLVLASVAAVCVNPRITTIREAGILCTAAALTLWDNNASAAWNSTTATGLCHVMRGSW
IAGASIAWTLIKNAEKPAFKRGRAGGRTLGEQWKEKLNAMGKEEFFSYRKEAILEVDRTEARRARR
EGNKVGGHPVSRGTAKLRWLVERRFVQPIGKVVDLGCGRGGWSYYAATMKNVQEVRGYTKGGPGHE
EPMLMQSYGWNIVTMKSGVDVFYKPSEISDTLLCDIGESSPSAEIEEQRTLRILEMVSDWLSRGPK
EFCIKILCPYMPKVIEKLESLQRRFGGGLVRVPLSRNSNHEMYWVSGASGNIVHAVNMTSQVLIGR
MDKKIWKGPKYEEDVNLGSGTRAVGKGVQHTDYKRIKSRIEKLKEEYAATWHTDDNHPYRTWTYHG
SYEVKPSGSASTLVNGVVRLLSKPWDAITGVTTMAMTDTTPFGQQRVFKEKVDTKAPEPPQGVKTV
MDETTNWLWAYLARNKKARLCTREEFVKKVNSHAALGAMFEEQNQWKNAREAVEDPKFWEMVDEER
ECHLRGECRTCIYNMMGKREKKPGEFGKAKGSRAIWFMWLGARFLEFEALGFLNEDHWMSRENSGG
GVEGAGIQKLGYILRDVAQKPGGKIYADDTAGWDTRITQADLENEAKVLELMEGEQRTLARAIIEL
TYRHKVVKVMRPAAGGKTVMDVISREDQRGSGQVVTYALNTFTNIAVQLVRLMEAEAVIGPDDIES
IERKKKFAVRTWLFENAEERVQRMAVSGDDCVVKPLDDRFSTALHFLNAMSKVRKDIQEWKPSQGW
YDWQQVPFCSNHFQEVIMKDGRTLVVPCRGQDELIGRARISPGSGWNVRDTACLAKAYAQMWLVLY
FHRRDLRLMANAICSSVPVDWVPTGRTTWSIHGKGEWMTTEDMLSVWNRVWILENEWMEDKTTVSD
WTEVPYVGKREDIWCGSLIGTRTRATWAENIYAAINQVRSVIGKEKYVDYVQSLRRYEETHVSEDR
VL

000000000000000000000000000000000000000000000000000000000000000000
000000000000000000000000000000000000000000000000000000000000000000
000000000000000000000000000000000000000000000000000000000000000000

000000000000000000000000000000000000000000000000000000000000000000
000000000000000000000000000000000000000000000000000000000000001111
111111111111000000000000000000000000000000000000000000000000000000
000000000000000000000000000000000000000000000000000000000000000000
000000000000000000000000000000111111111111111111111111000000000000
000000000000000000000000000000000000000000000000000000000000000000
000000000000000000000000000000000000000000000000000000000000000000
000000000000000000000000000000000000000000000000000000000000000000
000000000000000000000000000000000000000000000000000000000000000000
000000000000000000000000000000000000000000000000000000000000000000
000000000000000000000000000000000000000000000000000000000000000000
000000000000000000000000000000000000000000000000000000000000000000
000000000000000000000000000000000000000000000000000000000000000000
000000000000000000000000000000000000000000000000000000000000000000
000000000000000000000000000000000000000000000000000000000000000000
000000000000000000000000000000000000000000000000000000000000000000
000000000000000000000000000000000000000000000000000000000000000000
000000000000000000000000000000000000000000000000000000000000000000
000000000000000000000000000000000000000000000000000000000000000000
000000000000000000000000000000000000000000000000000000000000000000
000000000000000000000000000000000000000000000000000000000000000000
000000000000000000000000000000000000000000000000000000000000000000
000000000000000000000000000000000000000000000000000000000000000000
000000000000000000000000000000000000000000000000000000000000000000
000000000000000000000000000000000000000000000000000000000000000000
000000000000000000000000000000000000000000000000000000000000000000
000000000000000000000000000000000000000000000000000000000000000000
000000000000000000000000000000000000000000000000000000000000000000
000000000000000000000000000000000000000000000000000000000000000000
000000000000000000000000000000000000000000000000000000000000000000
000000000000000000000000000000000000000000000000000000000000000000
000000000000000000000000000000000000000000000000000000000000000000
000000000000000000000000000000000000000000000000000000000000000000
000000000000000000000000000000000000000000000000000000000000000000
000000000000000000000000000000000000000000000000000000000000000000
000000000000000000000000000000000000000000000000000000000000000000
000000000000000000000000000000000000000000000000000000000000000000
000000000000000000000000000000000000000000000000000000000000000000

0000000000000000000000000000000000000000000000000000000000000000000000000000
0000000000000000000000000000000000000000000000000000000000000000000000000000
0000000000000000000000000000000000000000000000000000000000000000000000000000
0000000000000000000000000000000000000000000000000000000000000000000000000000
0000000000000000000000000000000000000000000000000000000000000000000000000000
0000000000000000000000000000000000000000000000000000000000000000000000000000
0000000000000000000000000000000000000000000000000000000000000000000000000000
0000000000000000000000000000000000000000000000000000000000000000000000000000
0000000000000000000000000000000000000000000000000000000000000000000000000000
0000000000000000000000000000000000000000000000000000000000000000000000000000
0000000000000000000000000000000000000000000000000000000000000000000000000000
00
>P08617 Genome polyprotein [Contains: Coat protein VP4 (P1A); Coat protein VP2 (P1B); Coat protein VP3 (P1C); Coat protein VP1 (P1D); Picornain 2A (Core protein P2A); Core protein P2B; Core protein P2C; Probable protein P3A; Probable protein P3B; Picornain 3C (Protease 3C) (P3C); RNA-directed RNA polymerase (P3D)]. [mapped in Hepatitis A virus (STRAIN HM-175)]
MNMSRQGIFQTVGSGLDHILSLADIEEEQMIQSVDRTAVTGASYFTSVDQSSVHTAEVGSHQVEPL
RTSVDKPGSKKTQGEKFFLIHSADWLTTHALFHEVAKLDVVKLLYNEQFAVQGLLRYHTYARFGIE
IQVQINPTPFQQGGLICAMVPGDQSYGSIASLTVYPHGLLNCNINNVVRIKVPFIYTRGAYHFKDP
QYPVWELTIRVWSELNIGTGTSAYTSLNVLARFTDLELHGLTPLSTQMMRNEFRVSTTENVVNLSN
YEDARAKMSFALDQEDWKSDPSQGGGIKITHFTTWTSIPTLAAQFPFNASDSVGQQIKVIPVDPYF
FQMTNTNPDQKCITALASICQMFCFWRGDLVFDFQVFPTKYHSGRLLFCFVPGNELIDVSGITLKQ
ATTAPCAVMDITGVQSTLRFRVPWISDTPYRVNRYTKSAHQKGEYTAIGKLIVYCYNRLTSPSNVA
SHVRVNVYLSAINLECFAPLYHAMDVTTQVGDDSGGFSTTVSTEQNVPDPQVGITTMKDLKGKANR
GKMDVSGVQAPVGAITTIEDPVLAKKVPETFPELKPGESRHTSDHMSIYKFMGRSHFLCTFTFNSN
NKEYTFPITLSSTSNPPHGLPSTLRWFFNLFQLYRGPLDLTIIITGATDVDGMAWFTPVGLAVDTP
WVEKESALSIDYKTALGAVRFNTRRTGNIQIRLPWYSYLYAVSGALDGLGDKTDSTFGLVSIQIAN
YNHSDEYLSFSCYLSVTEQSEFYFPRAPLNSNAMLSTESMMSRIAAGDLESSVDDPRSEEDKRFES
HIECRKPYKELRLEVGKQRLKYAQEELSNEVLPPPRKMKGLFSQAKISLFYTEEHEIMKFSWRGVT
ADTRALRRFGFSLAAGRSVWTLEMDAGVLTGRLIRLNDEKWTEMKDDKIVSLIEKFTSNKYWSKVN
FPHGMLDLEEIAANSKDFPNMSETDLCFLLHWLNPKKINLADRMLGLSGVQEIKEQGVGLIAECRT
FLDSIAGTLKSMMFGFHHSVTVEIINTVLCFVKSGILLYVIQQLNQDEHSHIIGLLRVMNYADIGC
SVISCGKVFSKMLETVFNWQMDSRMMELRTQSFSNWLRDICSGITIFKNFKDAIYWLYTKLKDFYE
VNYGKKKDILNILKDNQQKIEKAIEEADEFCILQIQDVEKFEQYQKGVDLIQKLRTVHSMAQVDPN
LMVHLSPLRDCIARVHQKLKNLGSINQAMVTRCEPVVCYLYGKRGGGKSLTSIALATKICKHYGVE
PEKNIYTKPVASDYWDGYSGQLVCIIDDIGQNTTDEDWSDFCQLVSGCPMRLNMASLEEKGRHFSS

PFIIATSNWSNPSPKTVYVKEAIDRRLHFKVEVKPASFFKNPHNDMLNVNLAKTNDAIKDMSCVDL
IMDGHNVSLMDLLSSLVMTVEIRKQNMTEFMELWSQGISDDDNDSAVAEFFQSFPSGEPSNSKLSG
FFQSVTNHKWVAVGAAVGILGVLVGGWFVYKHFSRKEEEPIPAEGVYHGVTKPKQVIKLDADPVES
QSTLEIAGLVRKNLVQFGVGEKNGCVRWVMNALGVKDDWLLVPSHAYKFEKDYEMMEFYFNRGGTY
YSISAGNVVIQSLDVGFQDVVLMKVPTIPKFRDITQHFIKKGDVPRALNRLATLVTTVNGTPMLIS
EGPLKMEEKATYVHKKNDGTTVDLTVDQAWRGKGEGLPGMCGGALVSSNQSIQNAILGIHVAGGNS
ILVAKLVTQEMFQNIDKKIESQRIMKVEFTQCSMNVVSKTLFRKSPIYHHIDKTMINFPAAMPFSK
AEIDPMAVMLSKYSLPIVEEPEDYKEASIFYQNKIVGKTQLVDDFLDLDMAITGAPGIDAINMDSS
PGFPYVQEKLTKRDLIWLDENGLLLGVHPRLAQRILFNTVMMENCSDLDVVFTTCPKDELRPLEKV
LESKTRAIDACPLDYSILCRMYWGPAISYFHLNPGFHTGVAIGIDPDRQWDELFKTMIRFGDVGLD
LDFSAFDASLSPFMIREAGRIMSELSGTPSHFGTALINTIIYSKHLLYNCCYHVCGSMPSGSPCTA
LLNSIINNVNLYYVFSKIFGKSPVFFCQALKILCYGDDVLIVFSRDVQIDNLDLIGQKIVDEFKKL
GMTATSADKNVPQLKPVSELTFLKRSFNLVEDRIRPAISEKTIWSLIAWQRSNAEFEQNLENAQWF
AFMHGYEFYQKFYYFVQSCLEKEMIEYRLKSYDWWRMRFYDQCFICDLS
0000000000000000000000000000000000000000000000000000000000000000000000
0000000000000000000000000000000000000000000000000000000011111111111100
0000000000000000000000000000000000000000000000000000000000000000000000
0000000000000000000000000000000000000000000000000000000000000000000000
0000000000000000000000000000000000000000000000000000000000000000000000
0000000000000000000000000111111111111100000000000000000000000000000000
0000000000000000000000000000000000000000000000000000000000000000000000
0000000000000000000000000000000000000000011111111111111110000000000000
0000000000000000000000000000000000000000000000000000000000000000000000
0000000000000000000000000000000000000000000000000000000000000000000000
0000000000000000000000000000000000000000000000000000000000000000000000
0000000000000000000000000000000000000000000000000000000000000000000000
0000000000000000000000000000000000000000000000000000000000000000000000
0000000000000000000000000000000000000000000000000000000000000000000000
0000000000000000000000000000000000000000000000000000000000000000000000
0000000000000000000000000000000000000000000000000000000000000000000000
0000000000000000000000000000000000000000000000000000000000000000000000
0000000000000000000000000000000000000000000000000000000000000000000000
0000000000000000000000000000000000000000000000000000000000000000000000
0000000000000000000000000000000000000000000000000000000000000000000000
0000000000000000000000000000000000000000000000000000000000000000000000
0000000000000000000000000000000000000000000000000000000000000000000000
0000000000000000000000000000000000000000000000000000000000000000000000
0000000000000000000000000000000000000000000000000000000000000000000000

0000000000000000000000000000000000000000000000000000000000000000000000
0000000000000000000000000000000000000000000000000000000000000000000000
0000000000000000000000000000000000000000000000000000000000000000000000
0000000000000000000000000000000000000000000000000000000000000000000000
0000000000000000000000000000000000000000000000000000000000000000000000
0000000000000000000000000000000000000000000000000000000000000000000000
0000000000000000000000000000000000000000000000000000000000000000000000
0000000000000000000000000000000000000000000000000000000000000000000000
0000000000000000000000000000000000000000000000000000000000000000000000
0000000000000000000000000000000000000000000000000000
>42374894 merozoite surface protein 6 [Plasmodium falciparum]. [mapped in Plasmodium falciparum 3D7]
MNKIYNITFLFILLNLYINENNFIRNELINEKNHNLRNGSMYNNDKILSKNEVDTNIESNENSIHE
SGHKIDGEEVLKANVDDITYKKKNVDDSEIPFSGYDIQATYQFPSTSGGNNVIPLPIKQSGENQYT
VTSISGIQKGANGLTGATENITQVVQANSETNKNPTSHSNSTTTSLNNNILGWEFGGGAPQNGAAE
DKKTEYLLEQIKIPSWDRNNIPDENEQVKEDPQEDNKDEDEDEETETENLETEDDNNEEIEENEED
DIDEESVEEKEEEEEKKEEEEKKEEKKEEKKPDNEITNEVKEEQKYSSPSDINAQNLISNKNKKND
ETKKTAENIVKTLVGLFNEKNEIDSTINNLVQEMIHLFSNN
0000000000000000000000000000000000000000000000000000000000000000000000
0000000000000000000000000000000000000000000000000000000000000000000000
0000000000000000000000000000001111111111111111111111111111111111111111
1111111111111111111111111111000000000000000000000000000000000000000000
0000000000000000000111111111111111111111111111111111111111111111000000
0000000000000000000000000000000000000000000
>13622584 hypothetical protein SPy1494 [Streptococcus pyogenes M1 GAS]. [mapped in Streptococcus pyogenes M1 GAS]
MTSKKACLSSIIVLASLTCGNDTVSANHLSATGDKFDDCSTLVEKDVAPKDELEMLAWSSSQTTDD
ADRDYEDFLDDDSFISQNETDKMFENLTDDRLLNELDELDEENEEDEEDTIEPEQNVIMPSDDELF
DLTDAVETRLTVSSAPHLEAELPKPHLRSLSDTALRSGEIRGHLDNKLDALSVTATKLALTMAQKF
DLTTHVYSIGESFSEVLAAHYEDRKAESAFSKKKRFHLPIATPDVVIEELRRLVSSIGSSKEDVSV
PYSRKLGMAVAKRKIALPQTGERFSYYPVLLGLMILGLTPIMIPKKINN
0000000000000000000000000000000000000000011111111111111111111100000000
0000000000000000000000000000000000000000000000000000000000000000000000
0000000000000000000000000000000000000000000000000000000000000000000000
0000000000000000000000000000000000000000000000000000000000000000000000
0000000000000000000000000000000000000000000000000000
>P03524 Spike glycoprotein precursor. [mapped in Rabies virus (strain ERA)]

MVPQALLFVPLLVFPLCFGKFPIYTILDKLGPWSPIDIHHLSCPNNLVVEDEGCTNLSGFSYMELK
VGYILAIKMNGFTCTGVVTEAETYTNFVGYVTTTFKRKHFRPTPDACRAAYNWKMAGDPRYEESLH
NPYPDYRWLRTVKTTKESLVIISPSVADLDPYDRSLHSRVFPSGKCSGVAVSSTYCSTNHDYTIWM
PENPRLGMSCDIFTNSRGKRASKGSETCGFVDERGLYKSLKGACKLKLCGVLGLRLMDGTWVAMQT
SNETKWCPPDQLVNLHDFRSDEIEHLVVEELVRKREECLDALESIMTTKSVSFRRLSHLRKLVPGF
GKAYTIFNKTLMEADAHYKSVRTWNEILPSKGCLRVGGRCHPHVNGVFFNGIILGPDGNVLIPEMQ
SSLLQQHMELLESSVIPLVHPLADPSTVFKDGDEAEDFVEVHLPDVHNQVSGVDLGLPNWGKYVLL
SAGALTALMLIIFLMTCCRRVNRSEPTQHNLRGTGREVSVTPQSGKIISSWESHKSGGETRL
000000000000000000000000000000000000000000000000000000000000000000
000000000000000000000000000000000000000000000000000000000000000000
000000000000000000000000000000000000000000000000000000000000000000
000000000000000000000000000000000000000000000011111100000000000000
000111111111111111111111111111000000000000000000000000000000000000
000000111111000000000000000000000000000000000000000000000000000000
000000000000000000000000000000000000000000000000000000000000000000
00000000000000000000000000000000000000000000000000000000000000
>P10845 Botulinum neurotoxin type A precursor (BoNT/A) (Bontoxilysin-A) (BOTOX) [Contains: Botulinum neurotoxin A light-chain; Botulinum neurotoxin A heavy-chain]. [mapped in Clostridium botulinum]
MPFVNKQFNYKDPVNGVDIAYIKIPNVGQMQPVKAFKIHNKIWVIPERDTFTNPEEGDLNPPPEAK
QVPVSYYDSTYLSTDNEKDNYLKGVTKLFERIYSTDLGRMLLTSIVRGIPFWGGSTIDTELKVIDT
NCINVIQPDGSYRSEELNLVIIGPSADIIQFECKSFGHEVLNLTRNGYGSTQYIRFSPDFTFGFEE
SLEVDTNPLLGAGKFATDPAVTLAHELIHAGHRLYGIAINPNRVFKVNTNAYYEMSGLEVSFEELR
TFGGHDAKFIDSLQENEFRLYYYNKFKDIASTLNKAKSIVGTTASLQYMKNVFKEKYLLSEDTSGK
FSVDKLKFDKLYKMLTEIYTEDNFVKFFKVLNRKTYLNFDKAVFKINIVPKVNYTIYDGFNLRNTN
LAANFNGQNTEINNMNFTKLKNFTGLFEFYKLLCVRGIITSKTKSLDKGYNKALNDLCIKVNNWDL
FFSPSEDNFTNDLNKGEEITSDTNIEAAEENISLDLIQQYYLTFNFDNEPENISIENLSSDIIGQL
ELMPNIERFPNGKKYELDKYTMFHYLRAQEFEHGKSRIALTNSVNEALLNPSRVYTFFSSDYVKKV
NKATEAAMFLGWVEQLVYDFTDETSEVSTTDKIADITIIIPYIGPALNIGNMLYKDDFVGALIFSG
AVILLEFIPEIAIPVLGTFALVSYIANKVLTVQTIDNALSKRNEKWDEVYKYIVTNWLAKVNTQID
LIRKKMKEALENQAEATKAIINYQYNQYTEEEKNNINFNIDDLSSKLNESINKAMININKFLNQCS
VSYLMNSMIPYGVKRLEDFDASLKDALLKYIYDNRGTLIGQVDRLKDKVNNTLSTDIPFQLSKYVD
NQRLLSTFTEYIKNIINTSILNLRYESNHLIDLSRYASKINIGSKVNFDPIDKNQIQLFNLESSKI
EVILKNAIVYNSMYENFSTSFWIRIPKYFNSISLNNEYTIINCMENNSGWKVSLNYGEIIWTLQDT
QEIKQRVVFKYSQMINISDYINRWIFVTITNNRLNNSKIYINGRLIDQKPISNLGNIHASNNIMFK
LDGCRDTHRYIWIKYFNLFDKELNEKEIKDLYDNQSNSGILKDFWGDYLQYDKPYYMLNLYDPNKY
VDVNNVGIRGYMYLKGPRGSVMTTNIYLNSSLYRGTKFIIKKYASGNKDNIVRNNDRVYINVVVKN
KEYRLATNASQAGVEKILSALEIPDVGNLSQVVVMKSKNDQGITNKCKMNLQDNNGNDIGFIGFHQ

FNNIAKLVASNWYNRQIERSSRTLGCSWEFIPVDDGWGERPL
000000000000000000000000000111111111111111111111111111110000000000
000000000000000000000000000000000000000000000000000000000000000000
000000000000000000000000000000000000000000000000000000000000000000
000000000000000000000000000000000000000000000000000000000000000000
000000000000000000000000000000000000000000000000000000000000000000
000000000000000000000000000000000000000000000000000000000000000000
000000000000000000000000000000000000000000000000000000000000000000
000000000000000000000000000000000000000000000000000000000000000000
000000000000000000000000000000000000000000000000000000000000000000
000000000000000000000000000000000000000000000000000000000000000000
000000000000000000000000000000000000000000000000000000000000000000
000000000000000000000000000000000000000000000000000000000000000000
000000000000000000000000000000000000000000000000000000000000000000
000000000000000000000000000000000000000000000000000000000000000000
000000000000000000000000000000000000000000000000000000000000000000
000000000000000000000000000000000000000000000000000000000000000000
000000000000000000000000000000000000000000000000000000000000000000
000000000000000000000000000000000000000000000000000000000000000000
000000000000000000000000000000000000000000000000000000000000000000
000000000000000000000000000000000000000000
>Q69091 Glycoprotein D precursor. [mapped in Human herpesvirus 1]
MGGAAARLGAVILFVVIVGLHGVRSKYALVDASLKMADPNRFRGKDLPVLDQLTDPPGVRRVYHIQ
AGLPDPFQPPSLPITVYYAVLERACRSVLLNAPSEAPQIVRGASEDVRKQPYNLTIAWFRMGGNCA
IPITVMEYTECSYNKSLGACPIRTQPRWNYYDSFSAVSEDNLGFLMHAPAFETAGTYLRLVKINDW
TEITQFILEHRAKGSCKYALPLRIPPSACLSPQAYQQGVTVDSIGMLPRFIPENQRTVAVYSLKIA
GWHGPKAPYTSTLLPPELSETPNATQPELAPEDPEDSALLEDPVGTVAPQIPPNWHIPSIQDAATP
YHPPATPNNMGLIAGAVGGSLLAALVICGIVYWMRRHTQKAPKRIRLPHIREDDQPSSHQPLFY
000000000000000000000000000000000001111111100000000000000000000000
000000000000000000000000000000000000000000000000000000000000000000
000000000000000000000000000000000000000000000000000000000000000000
000000000000000000000000000000000000000000000000000000000000000000
000000000000000000000000000000000000000000000000000000000000000000
0000000000000000000000000000000000000000000000000000000000000000
>15644702 urease beta subunit (urea amidohydrolase) (ureB) [Helicobacter pylori 26695]. [mapped in Helicobacter pylori 26695]
MKKISRKEYVSMYGPTTGDKVRLGDTDLIAEVEHDYTIYGEELKFGGGKTLREGMSQSNNPSKEEL
DLIITNALIVDYTGIYKADIGIKDGKIAGIGKGGNKDMQDGVKNNLSVGPATEALAGEGLIVTAGG

IDTHIHFISPQQIPTAFASGVTTMIGGGTGPADGTNATTITPGRRNLKWMLRAAEEYSMNLGFLAK
GNASNDASLADQIEAGAIGFKIHEDWGTTPSAINHALDVADKYDVQVAIHTDTLNEAGCVEDTMAA
IAGRTMHTFHTEGAGGGHAPDIIKVAGEHNILPASTNPTIPFTVNTEAEHMDMLMVCHHLDKSIKE
DVQFADSRIRPQTIAAEDTLHDMGIFSITSSDSQAMGRVGEVITRTWQTADKNKKEFGRLKEEKGD
NDNFRIKRYLSKYTINPAIAHGISEYVGSVEVGKVADLVLWSPAFFGVKPNMIIKGGFIALSQMGD
ANASIPTPQPVYYREMFAHHGKAKYDANITFVSQAAYDKGIKEELGLERQVLPVKNCRNITKKDMQ
FNDTTAHIEVNPETYHVFVDGKEVTSKPANKVSLAQLFSIF
000000000000000000000000000000000000000000000000000000000000000000
000000000000000000000000000000000000000000000000000000000000000000
000000000000000000000000000000000000000000000000000000000000000000
000000000000000000000000000000000000000000000000000000000000000000
000000000000000000000000000000000000000000000000000000000000001111
111100000000000000000000000000000000000000000000000000000000000000
000000000000000000000000000000000000000000000000000000000000000000
000000000000000000000000000000000000000000000000000000000000000000
00000000000000000000000000000000000000000

>13621499 hypothetical protein SPy0210 [Streptococcus pyogenes M1 GAS]. [mapped in Streptococcus pyogenes M1 GAS]
MGVMMKQKIKILTVIGLMTVGMSACHNTSKPSNTDSVFSLTGKKRQQIVKQVRQRYYFQQLSKTEQ
ENYLTLYDSLAQFREIISLTPASKKSLIKTIDAFVMDNPEFYWITSADYRFEFSDQTVFVTFPIPE
DAKNIYQDLQAIGNDIVANTPSKDRYEQVKYFYEVIIRDTDYNKKAFEAYQSGSQAQVASNQDIKS
VFIDHLSVCNGYAQAFQFLCQKAGIPVAYIRGTGTSQQPQQSFAHAWNAVQINNTYYGVDVTWGDP
VFDNHLSHQKQGTINYSFLCLPDYLMALSHQPSKDIAFNTKERFENVWTIPSCTDDSLLYSKRHQS
YISTFDSDAILASLENQLLNRQEPLSLQFAHQDDYQQMVTDLTTNQTGYHNLFNQYWNNYTGFTYG
LLPETLSISFASRN
000000000000000000000001111111111111111111100000000000000000000000
000000000000000000000000000000000000000000000000000000000000000000
000000000000000000000000000000000000000000000000000000000000000000
000000000000000000000000000000000000000000000000000000000000000000
000000000000000000000000000000000000000000000000000000000000000000
000000000000000000000000000000000000000000000000000000000000000000
00000000000000

>13623184 conserved hypothetical protein [Streptococcus pyogenes M1 GAS]. [mapped in Streptococcus pyogenes M1 GAS]
MKNNNKWIIAGLASFLFPLSIIFIILLSMGIYYNSDKTILASDAFHQYVIFAQNFRNIMHGSDSFF
YTFTSGLGINFYALMCYYLGSFFSPLLFFFNLTSMPDAIYLFTLIKFGLIGLAACYSFHRLYPKIS
AFLMISISVFYSLMSFLTSQMELNSWLDVFILLPLVILGLNKLITENKTRTYYLSISLLFIQNYYF
GYMIALFCILYALVCLLRLNDFNKMFIAFVRFTAVSICAALTSALVILPTYLDLSTYGENLSPIKQ

LVTNNAWFLDIPAKLSIGVYDTTKFNALPMIYVGLFPLMLSVIYFTLESIPLKIKLANACLLTFII
ISFYLQPLDLFWQGMHSPNMFLHRYAWSFSIVILLLACETLSRLKEVTQIKAGFAFIFLIILTSLP
YSFSQQYNFLPLTLFLLSVFLLLGYTISLFSFRNSQIPSTFISAFILIFSLLESGLNTYYQLQGIN
KEWGFPSRQIYNSQLKDINNLVNSVSKNSQPFFRMERLLPQTGNDSMKFNYYGISQFSSVRNRLSS
SLLDRLGFQSKGTNLNLRYQNNTIIMDSLLGIKYNLSEGPPNKFGFTKLKTSGNTTLYQNHYSSPL
AILTRNVYKDVNLNVNTLDNQTKLLNQLSGKSLTYFNLQPAQLISGANQFNGQISAQASDYQNSVT
LNYQINIPKHSQLYVSIPNIIFSNPDAKEMRIQTDNHNFIYTTDNAYSFFDLGYFADAKVATFSFV
FPKNKQISFKEPHFYSLSIESYLEAMNSIKQKNVHTYAKSNTVITDYNSKTKGSLIFTLPYDKGWS
AQKDGKNLPVKKAQGGFLSVTIPKGKGRVILTFIPNGFKLGLSLSCVGIIAYMLLYKYIDIKSKLL
000000000000000000000000000000000000000000000000000111111111111111
111110000000000000000000000000000000000000000000000000000000000000
000000000000000000000000000000000000000000000000000000000000000000
000000000000000000000000000000000000000000000000000000000000000000
000000000000000000000000000000000000000000000000000000000000000000
000000000000000000000000000000000000000000000000000000000000000000
000000000000000000000000000000000000000000000000000000000000000000
000000000000000000000000000000000000000000000000000000000000000000
000000000000000000000000000000000000000000000000000000000000000000
000000000000000000000000000000000000000000000000000000000000000000
000000000000000000000000000000000000000000000000000000000000000000
000000000000000000000000000000000000000000000000000000000000000000
000000000000000000000000000000000000000000000000000000000000000000
>P01552 Enterotoxin type B precursor (SEB). [mapped in Staphylococcus aureus]
MYKRLFISHVILIFALILVISTPNVLAESQPDPKPDELHKSSKFTGLMENMKVLYDDNHVSAINVK
SIDQFLYFDLIYSIKDTKLGNYDNVRVEFKNKDLADKYKDKYVDVFGANYYYQCYFSKKTNDINSH
QTDKRKTCMYGGVTEHNGNQLDKYRSITVRVFEDGKNLLSFDVQTNKKKVTAQELDYLTRHYLVKN
KKLYEFNNSPYETGYIKFIENENSFWYDMMPAPGDKFDQSKYLMMYNDNKMVDSKDVKIEVYLTTK
KK
000000000000000000000000000000000000000000000000000000000000000000
000000000000000000000000000001111111100000000000000000000000000000
000000000000000000000000000000000000000000000000000000000000000000
000000000000000000000000000000000000000000000000000001111111111000
00
>P21206 Virulence-associated V antigen (Low calcium response locus protein V). [mapped in Yersinia pestis]
MIRAYEQNPQHFIEDLEKVRVEQLTGHGSSVLEELVQLVKDKNIDISIKYDPRKDSEVFANRVITD
DIELLKKILAYFLPEDAILKGGHYDNQLQNGIKRVKEFLESSPNTQWELRAFMAVMHFSLTADRID

DDILKVIVDSMNHHGDARSKLREELAELTAELKIYSVIQAEINKHLSSSGTININHDKSINLMDKNL
YGYTDEEIFKASAEYKILEKMPQTTIQVDGSEKKIVSIKDFLGSENKRTGALGNLKNSYSYNKDNN
ELSHFATTCSDKSRPLNDLVSQKTTQLSDITSRFNSAIEALNRFIQKYDSVMQRLLDDTSGK
111111111111111111111111111111000000000000000000000000000000000000
000000000000000000000000000000000000000000000000000000000000000000
000000000000000000000000000000000000000000000000000000000000000000
000000000000000000000000000000000000000000000000000000000000000000
00000000000000000000000000000000000000000000000000000000000000
>P55128 RTX-I toxin determinant A from serotypes 1/9 (ApxI) (APX-IA) (Hemolysin IA) (HLY-IA) (Cytolysin IA) (CLY-IA). [mapped in Actinobacillus pleuropneumoniae serovar 1 str. 4074]
MANSQLDRVKGLIDSLNQHTKSAAKSGAGALKNGLGQVKQAGQKLILYIPKDYQASTGSSLNDLVK
AAEALGIEVHRSEKNGTALAKELFGTTEKLLGFSERGIALFAPQFDKLLNKNQKLSKSLGGSSEAL
GQRLNKTQTALSALQSFLGTAIAGMDLDSLLRRRRNGEDVSGSELAKAGVDLAAQLVDNIASATGT
VDAFAEQLGKLAMPYLTLALSGLASKLNNLPDLSLAGPGFDAVSGILSVVSASFILSNKDADAGTK
AAAGIEISTKILGNIGKAVSQYIIAQRVAAGLSTTAATGGLIGSVVALAISPLSFLNVADKFERAK
QLEQYSERFKKFGYEGDSLLASFYRETGAIEAALTTINSVLSARSAGVGAAATGSLVGAPVAALVS
AITGIISGILDASKQAIFERVATKLANKIDEWEKKHGKNYFENGYDARHSAFLEDTFELLSQYNKE
YSVERVVAITQQRWDVNIGELAGITRKGSDTKSGKAYVDFFEEGKLLEKEPDRFDKKVFDPLEGKI
DLSSINKTTLLKFVTPVFTAGEEIRERKQTGKYQYMTELFVKGKEKWVVTGVQSHNAIYDYTNLIQ
LAIDKKGEKRQVTIESHLGEKNDRIYLSSGSSIVYAGNGHDVAYYDKTDTGYLTFDGQSAQKAGEY
IVTKELKADVKVLKEVVKTQDISVGKTCSEKLEYRDYELSPFELGNGIRAKDELHSVEEIIGSNRK
DKFFGSRFTDIFHGAKGDDEIYGNDGHDILYGDDGNDVIHGGDGNDHLVGGNGNDRLIGGKGNNFL
NGGDGDDELQVFEGQYNVLLGGAGNDILYGSDGTNLFDGGVGNDKIYGGLGKDIYRYSKEYGRHII
IEKGGDDDTLLLSDLSFKDVGFIRIGDDLLVNKRIGGTLYYHEDYNGNALTIKDWFKEGKEGQNNK
IEKIVDKDGAYVLSQYLTELTAPGRGINYFNGLEEKLYYGEGYNALPQLRKDIEQIISSTGAFTGD
HGKVSVGSGGPLVYNNSANNVANSLSYSLAQAA
000000000000000000000000000000000000000000000000000000000000000000
000000000000000000000000000000000000000000000000000000000000000000
000000000000000000000000000000000000000000000000000000000000000000
000000000000000000000000000000000000000000000000000000000000000000
000000000000000000000000000000000000000000000000000000000000000000
000000000000000000000000000000000000000000000000000000000000000000
000000000000000000000000000000000000000000000000000000000000000000
000000000000000000000000000000000000000000000000000000000000000000
000000000000000000000000000000000000000000000000000000000000000000
000000000000000000000000000000000000000000000000000000000000000000
000000000000000000000000000000000000000000000000000000000000000000

000000000000000001111111111100000000000000000000000000000000000000
000000000000000000000000000000000000000000000000000000000000000000
000000000000000000000000000000000000000000000000000000000000000000
000000000000000000000000000000000000000000000000000000000000000000
000000000000000000000000000000000
>Q02938 56 kDa type-specific antigen precursor (TSA) (56 kDa scrub typhus antigen) (STA56) (TSB56) (Bor56). [mapped in Orientia tsutsugamushi]
MKKIMLIASAMSALSLPFSASAIELEDEVGLECGPYAKVGVVGGMITGAESTRLDSTDSEGKKHLS
LTTGLPFGGTLAAGMTIAPGFRAELGVMYLRNISAEVEVGKGEVDSKGEIKADSGGGTDAPIRKPF
KLTPPQPTMSPISIADRDFGIDIPNIPQAQRQAAQPPLNDQKRAAARIAWLKNCAGIDYMVKDPNN
PGHMMVNPVLLNIPQGNPNPVGQPPQRANQPANFAIHNHEQWRSLVVGLAALSNANKPSASPVKVL
SDKIIQIYSDIKPFADIAGINVPDTGLPNSASIEQIQSKIQELGDTLEELRDSFDGYINNAFVNQI
HLNFVMPPQAQQQQGQGQQQQAQATAQEAVAAAAVRLLNGSDQIAQLYKDLVKLQRHAGIRKAMEK
LAAQQEEDAKNQGKGDCKQQQGASEKSKEGKVKETEFDLSMVVGQVKLYADLVTTESFSIYGGVGA
GLGSYTYAKIDNKDVKGYTGMVASGALGVAINAAEGVCVDLEAGYMHSFSKVEDKYQVNAFIASAC
VRYNF
000000000000000000000000000000000000000000000000000000000000000000
000000000000000000000000000000000000000000000000000000000000000000
000000011111111111111111111100000000000000000000000000111111111111
111111111111111000000000000000000000000000000000000000000000000000
000000000000000000000000000000000000000000000000000000000000000000
000000000000000000000000000000000000000000000000000000000000000000
000000000000000000000000000000000000000000000000000000000000000000
000000000000000000000000000000000000000000000000000000000000000000
00000
>P03449 Hemagglutinin precursor [Contains: Hemagglutinin HA1 chain; Hemagglutinin HA2 chain]. [mapped in Influenza A virus (A/Memphis/1/71(H3N2))]
MKTIIALSHIFCLVLGQYLPGNDNSTATLCLGHHAVPNGTLVKTITNDQIEVTNATELVQSSSTGK
ICNNPHRILDGIDCTLIDALLGDPHCDGFQNETWDLFVERSKAFSNCYPYDVPDYASLRSLVASSG
TLEFITEGFTWTGVTQNGGSNACKRGPGSGFFSRLNWLTKSESTYPVLNVTMPNNDNFDKLYIWGV
HHPSTNQEQTSLYVQASGRVTVSTRRSQQTIIPNIGSRPWVRGLSSRISIYWTIVKPGDVLVINSN
ANLIAPRGYFKMRTGKSSIMRSDAPIDTCISECITPNGSIPNDKPFQNVNKITYGACPKYVKQNTL
KLATRMRNVPEKQTRGLFGAIAGFIENGWEGMIDGWYGFRHQNSEGTGQAADLKSTQAAIDQINGK
LNRVIEKTNEKFHQIEKEFSEVEGRIQDLEKYVEDTKIDLWSYNAELLVALENQHTIDLTDSEMNK
LFEKTRRQLRENAEDMGNGCFKIYDKCDNACIESIRNGTYDHDVYRDEALNNRFQIKGVELKSGYK
DWILWISFAISCFLLCVVLLGFIMWACQRGNIRCNICI

000000000000000000000000000000000000000000000000000000000000000000
000000000000000000000000000000000000000011111111111111111100000000
111111110011111110000000000000000000000000000000000000000000000000
111111111111100000000000011111110000000000000000000000000000000000
000000000000000000000000000000000000000000000000000000000000000001
111101111111110000000000000000000000000000000000000000000000000000
000000000000000000000000000000000000000000000000000000000000000000
000000000000000000000000000000000000000000000000000000000000000000
00000000000000000000000000000000000000
>P30129 Coat protein VP2. [mapped in Canine parvovirus]
MSDGAVQPDGGQPAVRNERATGSGNGSGGGGGGGSGGVGISTGTFNNQTEFKFLENGWVYITANSS
RLVHLNMPESENYRRVVVNNMDKTAVNGNMALDDIHAEIVTPWSLVDANAWGVWFNPGDWQLIVNT
MSELHLVSFEQEIFNVVLKTVSESATQPPTKVYNNDLTASLMVALDSNNTMPFTPAAMRSETLGFY
PWKPTIPTPWRYYFQWDRTLIPSHTGTSGTPTNIYHGTDPDDVQFYTIENSVPVHLLRTGDEFATG
TFFFDCKPCRLTHTWQTNRALGLPPFLNSLPQSEGATNFGDIGVQQDKRRGVTQMGNTNYITEATI
MRPAEVGYSAPYYSFEASTQGPFKTPIAAGRGGAQTDENQAADGNPRYAFGRQHGKKTTTTGETPE
RFTYIAHQDTGRYPEGDWIQNINFNLPVTNDNVLLPTDPIGGKTGINYTNIFNTYGPLTALNNVPP
VYPNGQIWDKEFDTDLKPRLHVNAPFVCQNNCPGQLFVKVAPNLTNQYDPDASANMSRIVTYSDFW
WKGKLVFKAKLRASHTWNPIQQMSINVDNQFNYVPSNIGGMKIVYEKSQLAPRKLY
111111111111111111111100000000000000000000000000000000000000000000
000000000000000000000000000000000000000000000000000000000000000000
000000000000000000000000000000000000000000000000000000000000000000
000000000000000000000000000000000000000000000000000000000000000000
000000000000000000000000000000000000000000000000000000000000000000
000000000000000000000000000000000000000000000000000000000000000000
000000000000000000000000000000000000000000000000000000000000000000
000000000000000000000000000000000000000000000000000000000000000000
00000000000000000000000000000000000000000000000000000000
>P01558 Heat-labile enterotoxin B chain. [mapped in Clostridium perfringens]
MLSNNLNPMVFENAKEVFLISEDLKTPINITNSNSNLSDGLYVIDKGDGWILGEPSVVSSQILNPN
ETGTFSQSLTKSKEVSINVNFSVGFTSEFIQASVEYGFGITIGEQNTIERSVSTTAGPNEYVYYKV
YATYRKYQAIRISHGNISDDGSIYKLTGIWLSKTSADSLGNIDQGSLIETGERCVLTVPSTDIEKE
ILDLAAATERLNLTDALNSNPAGNLYDWRSSNSYPWTQKLNLHLTITATGQKYRILASKIVDFNIY
SNNFNNLVKLEQSLGDGVKDHYVDISLDAGQYVLVMKANSSYSGNYPYSILFQKF
000000000000000000000000000000000000000000000000000000000000000000
000000000000000000000000000000000000000000000000000000000000000000
000000000000000000000000000000000000000000000000000000000000000000

000000000000000000000000000000000000000000000000000000000000000000
0000000000000000000000000111111111111111111111111111111
>P0A0L2 Enterotoxin type A precursor (SEA). [mapped in Staphylococcus aureus]
MKKTAFTLLLFIALTLTTSPLVNGSEKSEEINEKDLRKKSELQGTALGNLKQIYYYNEKAKTENKE
SHDQFLQHTILFKGFFTDHSWYNDLLVDFDSKDIVDKYKGKKVDLYGAYYGYQCAGGTPNKTACMY
GGVTLHDNNRLTEEKKVPINLWLDGKQNTVPLETVKTNKKNVTVQELDLQARRYLQEKYNLYNSDV
FDGKVQRGLIVFHTSTEPSVNYDLFGAQGQYSNTLLRIYRDNKTINSENMHIDIYLYTS
000000000000000000000000111111100000000000000000000000000000000000
000000000000000000000000000000000000000000000000000000000000000000
000000000000000000000000000000000000000000000111111100000000000000
00000000000000000000000000000000000000000000000000000000000
>P26948 F1 capsule antigen precursor. [mapped in Yersinia pestis]
MKKISSVIAIALFGTIATANAADLTASTTATATLVEPARITLTYKEGAPITIMDNGNIDTELLVGT
LTLGGYKTGTTSTSVNFTDAAGDPMYLTFTSQDGNNHQFTTKVIGKDSRDFDISPKVNGENLVGDD
VVLATGSQDFFVRSIGSKGGKLAAGKYTDAVTVTVSNQ
000000000000000000000000000000000000000000000000000000000000000000
000000000000000000000000000000000000000000000000000000000000000000
00000000011111111111111111111111100000
>13622014 hypothetical protein SPy0843 [Streptococcus pyogenes M1 GAS]. [mapped in Streptococcus pyogenes M1 GAS]
MKKHLKTVALTLTTVSVVTHNQEVFSLVKEPILKQTQASSSISGADYAESSGKSKLKINETSGPVD
DTVTDLFSDKRTTPEKIKDNLAKGPREQELKAVTENTESEKQITSGSQLEQSKESLSLNKTVPSTS
NWEICDFITKGNTLVGLSKSGVEKLSQTDHLVLPSQAADGTQLIQVASFAFTPDKKTAIAEYTSRA
GENGEISQLDVDGKEIINEGEVFNSYLLKKVTIPTGYKHIGQDAFVDNKNIAEVNLPESLETISDY
AFAHLALKQIDLPDNLKAIGELAFFDNQITGKLSLPRQLMRLAERAFKSNHIKTIEFRGNSLKVIG
EASFQDNDLSQLMLPDGLEKIESEAFTGNPGDDHYNNRVVLWTKSGKNPSGLATENTYVNPDKSLW
QESPEIDYTKWLEEDFTYQKNSVTGFSNKGLQKVKRNKNLEIPKQHNGVTITEIGDNAFRNVDFQN
KTLRKYDLEEVKLPSTIRKIGAFAFQSNNLKSFEASDDLEEIKEGAFMNNRIETLELKDKLVTIGD
AAFHINHIYAIVLPESVQEIGRSAFRQNGANNLIFMGSKVKTLGEMAFLSNRLEHLDLSEQKQLTE
IPVQAFSDNALKEVLLPASLKTIREEAFKKNHLKQLEVASALSHIAFNALDDNDGDEQFDNKVVVK
THHNSYALADGEHFIVDPDKLSSTIVDLEKILKLIEGLDYSTLRQTTQTQFRDMTTAGKALLSKSN
LRQGEKQKFLQEAQFFLGRVDLDKAIAKAEKALVTKKATKNGQLLERSINKAVLAYNNSAIKKANV
KRLEKELDLLTGLVEGKGPLAQATMVQGVYLLKTPLPLPEYYIGLNVYFDKSGKLIYALDMSDTIG
EGQKDAYGNPILNVDEDNEGYHALAVATLADYEGLDIKTILNSKLSQLTSIRQVPTAAYHRAGIFQ
AIQNAAAEAEQLLPKPGTHSEKSSSSESANSKDRGLQSNPKTNRGRHSAILPRTGSKGSFVYGILG
YTSVALLSLITAIKKKKY
000000000000000000001111111111111111111000000000000000000000000000

000000000000000000000000000000000000000000000000000000000000000000
000000000000000000000000000000000000000000000000000000000000000000
000000000000000000000000000000000000000000000000000000000000000000
000000000000000000000000000000000000000000000000000000000000000000
000000000000000000000000000000000000000000000000000000000000000000
000000000000000000000000000000000000000000000000000000000000000000
000000000000000000000000000000000000000000000000000000000000000000
000000000000000000000000000000000000000000000000000000000000000000
000000000000000000000000000000000000000000000000000000000000000000
000000000000000000000000000000000000000000000000000000000000000000
000000000000000000000000000000000000000000000000000000000000000000
000000000000000000000000000000000000000000000000000000000000000000
000000000000000000000000000000000000000000000000000000000000000000
000000000000000000000000000000000000000000000000000000000000000000
000000000000000000
>P06886 Toxic shock syndrome toxin-1 precursor (TSST-1). [mapped in Staphylococcus aureus]
MNKKLLMNFFIVSPLLLATTATDFTPVPLSSNQIIKTAKASTNDNIKDLLDWYSSGSDTFTNSEVL
DNSLGSMRIKNTDGSISLIIFPSPYYSPAFTKGEKVDLNTKRTKKSQHTSEGTYIHFQISGVTNTE
KLPTPIELPLKVKVHGKDSPLKYGPKFDKKQLAISTLDFEIRHQLTQIHGLYRSSDKTGGYWKITM
NDGSTYQSDLSKKFEYNTEKPPINIDEIKTIEAEIN
000000000000000000000000000000000000000000000000000000000000000000
000000000000000000001111111111111111110000000000000000000000000000
000000000000000000000000000000000000000000000000000000000000000000
000000000000000000000000000000000000
>P15917 Lethal factor precursor (LF) (Anthrax lethal toxin endopeptidase component). [mapped in Bacillus anthracis]
MNIKKEFIKVISMSCLVTAITLSGPVFIPLVQGAGGHGDVGMHVKEKEKNKDENKRKDEERNKTQE
EHLKEIMKHIVKIEVKGEEAVKKEAAEKLLEKVPSDVLEMYKAIGGKIYIVDGDITKHISLEALSE
DKKKIKDIYGKDALLHEHYVYAKEGYEPVLVIQSSEDYVENTEKALNVYYEIGKILSRDILSKINQ
PYQKFLDVLNTIKNASDSDGQDLLFTNQLKEHPTDFSVEFLEQNSNEVQEVFAKAFAYYIEPQHRD
VLQLYAPEAFNYMDKFNEQEINLSLEELKDQRMLARYEKWEKIKQHYQHWSDSLSEEGRGLLKKLQ
IPIEPKKDDIIHSLSQEEKELLKRIQIDSSDFLSTEEKEFLKKLQIDIRDSLSEEEKELLNRIQVD
SSNPLSEKEKEFLKKLKLDIQPYDINQRLQDTGGLIDSPSINLDVRKQYKRDIQNIDALLHQSIGS
TLYNKIYLYENMNINNLTATLGADLVDSTDNTKINRGIFNEFKKNFKYSISSNYMIVDINERPALD
NERLKWRIQLSPDTRAGYLENGKLILQRNIGLEIKDVQIIKQSEKEYIRIDAKVVPKSKIDTKIQE
AQLNINQEWNKALGLPKYTKLITFNVHNRYASNIVESAYLILNEWKNNIQSDLIKKVTNYLVDGNG
RFVFTDITLPNIAEQYTHQDEIYEQVHSKGLYVPESRSILLHGPSKGVELRNDSEGFIHEFGHAVD

DYAGYLLDKNQSDLVTNSKKFIDIFKEEGSNLTSYGRTNEAEFFAEAFRLMHSTDHAERLKVQKNA
PKTFQFINDQIKFIINS
000000000000000000000000000000000000000000000000000000000000000000
000000000000000000000000000000000000000000000000000000000000000000
000000000000000000000000000000000000000000000000000000000000000000
000000000000000000000000000000000000000000000000000000000000000000
000000000000000000000000000000000000000000000000000000000000000000
000000000000000000000000000000000000000000000000011111111111111111
100000000000000000000000000000000000000000000000000000000000000000
000000000000000000000000000000000000000000000000000000000000000000
000000000000000000000000000000000000000000000000000000000000000000
000000000000000000000000000000000000000000000000000000000000000000
000000000000000000000000000000000000000000000000000000000000000000
000000000000000000000000000000000000000000000000000000000000000000
00000000000000000
>30260755 gi|30260755|ref|NP_843132.1| neutral protease [Bacillus anthracis str. Ames]
MKKKSLALVLATGMAVTTFGGTGSAFADSKNVLSTKKYNETVQSPEFISGDLTEATGKKAESVVFD
YLNAAKGDYKLGEKSAQDSFKVKQAKKDAVTDSTVLRLQQVYEGVPVWGSTQVAHVSKDGSLKVLS
GTVAPDLDKKEKLKNKNKIEGAKAIEIAQKDLGVTPKYEVEPKADLYVYQNGEETTYAYVVNLNFL
EPSPGNYYYFIEADSGKVLNKYNKLDHVANEDKSPVKQEAPKQEAKPAVKPVTGTNAVGTGKGVLG
DTKSLNTTLSASSYYLQDNTRGATIFTYDAKNRSTLPGTLWVDADNVFNAAYDAAAVDAHYYAGRT
YDYYKATFNRNSINDAGAPLKSTVHYGSRYNNAFWNGSQMVYGDGDGVTFTSLSGGIDVIGHELTH
AVTEYSSDLIYQNESGALNEAISDVFGTLVEYYDNRNPDWEIGEDIYTPGKAGDALRSMSDPTKYG
DPDHYSKRYTGTGDNGGVHTNSGIINKAAYLLANGGTHYGVTVNGIGKDKVGAIYYRANTQYFTQS
TTFSQARAGLVQAAADLYGASSAEVAAVKQSYSAVGVN
000000000000000000000000000000000000000000000000000000000000000000
000000000000000000000000000000000000000000000000000000000000000000
000000000000000000000000000000000000000000000000000000000000000000
000000000000000000000000000000000000000000000000000000000000000000
000000000000000000000000000000000000000000000000000000000000000000
000000000000000000000000000000000000000000000000000000000111111111
111100000000000000000000000000000000000000000000000000000000000000
000000000000000000000000000000000000000000000000000000000000000000
00000000000000000000000000000000000000
