# Supplementary material for: Prediction of B-cell epitopes using evolutionary information and propensity scales
Source: BMC Bioinformatics. 2013 Jan 21;14(Suppl 2):S10. doi: 10.1186/1471-2105-14-S2-S10 (PMC3549808; doi:10.1186/1471-2105-14-S2-S10)
Supplement: Additional file 2 — The AntiJen#1 dataset. [file 1471-2105-14-S2-S10-S2.pdf]

**Additional file 2. AntiJen dataset #1.**

>2505 CAC1A_HUMAN O00555 Voltage-dependent P/Q-type calcium channel alpha-1A subunit (Voltage- gated calcium channel alpha subunit Cav2.1) (Calcium channel, L type, alpha-1 polypeptide isoform 4) (Brain calcium channel I) (BI). - Homo sapiens (Human).
MARFGDEMPARYGGGGSGAAAGVVVGSGGGRGAGGSRQGGQPGAQRMYKQSMAQRARTMALYNPIP
VRQNCLTVNRSLFLFSEDNVVRKYAKKITEWPPFEYMILATIIANCIVLALEQHLPDDDKTPMSER
LDDTEPYFIGIFCFEAGIKIIALGFAFHKGSYLRNGWNVMDFVVVLTGILATVGTEFDLRTLRAVR
VLRPLKLVSGIPSLQVVLKSIMKAMIPLLQIGLLLFFAILIFAIIGLEFYMGKFHTTCFEEGTDDI
QGESPAPCGTEEPARTCPNGTKCQPYWEGPNNGITQFDNILFAVLTVFQCITMEGWTDLLYNSNDA
SGNTWNWLYFIPLIIIGSFFMLNLVLGVLSGEFAKERERVENRRAFLKLRRQQQIERELNGYMEWI
SKAEEVILAEDETDGEQRHPFDGALRRTTIKKSKTDLLNPEEAEDQLADIASVGSPFARASIKSAK
LENSTFFHKKERRMRFYIRRMVKTQAFYWTVLSLVALNTLCVAIVHYNQPEWLSDFLYYAEFIFLG
LFMSEMFIKMYGLGTRPYFHSSFNCFDCGVIIGSIFEVIWAVIKPGTSFGISVLRALRLLRIFKVT
KYWASLRNLVVSLLNSMKSIISLLFLLFLFIVVFALLGMQLFGGQFNFDEGTPPTNFDTFPAAIMT
VFQILTGEDWNEVMYDGIKSQGGVQGGMVFSIYFIVLTLFGNYTLLNVFLAIAVDNLANAQELTKD
EQEEEEAANQKLALQKAKEVAEVSPLSAANMSIAVKEQQKNQKPAKSVWEQRTSEMRKQNLLASRE
ALYNEMDPDERWKAAYTRHLRPDMKTHLDRPLVVDPQENRNNNTNKSRAAEPTVDQRLGQQRAEDF
LRKQARYHDRARDPSGSAGLDARRPWAGSQEAELSREGPYGRESDHHAREGSLEQPGFWEGEAERG
KAGDPHRRHVHRQGGSRESRSGSPRTGADGEHRRHRAHRRPGEEGPEDKAERRARHREGSRPARGG
EGEGEGPDGGERRRRHRHGAPATYEGDARREDKERRHRRRKENQGSGVPVSGPNLSTTRPIQQDLG
RQDPPLAEDIDNMKNNKLATAESAAPHGSLGHAGLPQSPAKMGNSTDPGPMLAIPAMATNPQNAAS
RRTPNNPGNPSNPGPPKTPENSLIVTNPSGTQTNSAKTARKPDHTTVDIPPACPPPLNHTVVQVNK
NANPDPLPKKEEEKKEEEEDDRGEDGPKPMPPYSSMFILSTTNPLRRLCHYILNLRYFEMCILMVI
AMSSIALAAEDPVQPNAPRNNVLRYFDYVFTGVFTFEMVIKMIDLGLVLHQGAYFRDLWNILDFIV
VSGALVAFAFTGNSKGKDINTIKSLRVLRVLRPLKTIKRLPKLKAVFDCVVNSLKNVFNILIVYML
FMFIFAVVAVQLFKGKFFHCTDESKEFEKDCRGKYLLYEKNEVKARDREWKKYEFHYDNVLWALLT
LFTVSTGEGWPQVLKHSVDATFENQGPSPGYRMEMSIFYVVYFVVFPFFFVNIFVALIIITFQEQG
DKMMEEYSLEKNERACIDFAISAKPLTRHMPQNKQSFQYRMWQFVVSPPFEYTIMAMIALNTIVLM
MKFYGASVAYENALRVFNIVFTSLFSLECVLKVMAFGILNYFRDAWNIFDFVTVLGSITDILVTEF
GNNFINLSFLRLFRAARLIKLLRQGYTIRILLWTFVQSFKALPYVCLLIAMLFFIYAIIGMQVFGN
IGIDVEDEDSDEDEFQITEHNNFRTFFQALMLLFRSATGEAWHNIMLSCLSGKPCDKNSGILTREC
GNEFAYFYFVSFIFLCSFLMLNLFVAVIMDNFEYLTRDSSILGPHHLDEYVRVWAEYDPAAWGRMP
YLDMYQMLRHMSPPLGLGKKCPARVAYKRLLRMDLPVADDNTVHFNSTLMALIRTALDIKIAKGGA
DKQQMDAELRKEMMAIWPNLSQKTLDLLVTPHKSTDLTVGKIYAAMMIMEYYRQSKAKKLQAMREE
QDRTPLMFQRMEPPSPTQEGGPGQNALPSTQLDPGGALMAHESGLKESPSWVTQRAQEMFQKTGTW
SPEQGPPTDMPNSQPNSQSVEMREMGRDGYSDSEHYLPMEGQGRAASMPRLPAENQRRRGRPRGNN

LSTISDTSPMKRSASVLGPKARRLDDYSLERVPPEENQRHHQRRRDRSHRASERSLGRYTDVDTGL
GTDLSMTTQSGDLPSKERDQERGRPKDRKHRQHHHHHHHHHPPPPDKDRYAQERPDHGRARARDQ
RWSRSPSEGREHMAHRQGSSSVSGSPAPSTSGTSTPRRGRRQLPQTPSTPRPHVSYSPVIRKAGGS
GPPQQQQQQQQQQAVARPGRAATSGPRRYPGPTAEPLAGDRPPTGGHSSGRSPRMERRVPGPARS
ESPRACRHGGARWPASGPHVSEGPPGPRHHGYYRGSDYDEADGPGSGGGEEAMAGAYDAPPPVRHA
SSGATGRSPRTPRASGPACASPSRHGRRLPNGYYPAHGLARPRGPGSRKGLHEPYSESDDDWC
000000000000000000000000000000000000000000000000000000000000000000
000000000000000000000000000000000000000000000000000000000000000000
000000000000000000000000000000000000000000000000000000000000000000
000000000000000000000000000000000000000000000000000000000000000000
000000000000000000000000000000000000000000000000000000000000000000
000000000000000000000000000000000000000000000000000000000000000000
000000000000000000000000000000000000000000000000000000000000000000
000000000000000000000000000000000000000000000000000000000000000000
000000000000000000000000000000000000000111111111110000000000000000
000000000000000000000000000000000000000000111111111110000000000000
000000000000000000000000000000000000000000000000000000000000000000
000000000000000000000000000000000000000000000000000000000000000000
000000000000000000000000000000000000000000000000000000000000000000
000000000000000000000000000000000000000000000000000000000000000000
000000000000000000000000000000000000000000000000000000000000000000
000000000000000000000000000000000000000000000000000000000000000000
000000000000000000000000000000000000000000000000000000000000000000
000000000000000000000000000000000000000000000000000000000000000000
000000000000000000000000000000000000000000000000000000000000000000
000000000000000000000000000000000000000000000000000000000000000000
000000000000000000000000000000000000000000000000000000000000000000
000000000000000000000000000000000000000000000000000000000000000000
000000000000000000000000000000000000000000000000000000000000000000
000000000000000000000000000000000000000000000000000000000000000000
000000000000000000000000000000000000000000000000000000000000000000
000000000000000000000000000000000000000000000000000000000000000011
111111111111111111100000000000000000000000000000000000000000000000
000000000000000000000000000000000000000000000000000000000000000000
000000000000000000000000000000000000000000000000000000000000000000
000000000000000000000000000000000000000000000000000000000000000000
000000000000000000000000000000000000000000000000000000000000000000
000000000000000000000000000000000000000000000000000000000000000000

000000000000000000000000000000000000000000000000000000000000000000
000000000000000000000000000000000000000000000000000000000000000000
000000000000000000000000000000000000000000000000000000000000000000
000000000000000000000000000000000000000000000000000000000000000000
000000000000000000000000000000000000000000000000000000000000000000
000000000000000000000000000000000000000000000000000000000000000
>192 RAC3_MOUSE  P60764 Ras-related C3 botulinum toxin substrate 3 (p21-Rac3). - Mus musculus (Mouse).
MQAIKCVVVGDGAVGKTCLLISYTTNAFPGEYIPTVFDNYSANVMVDGKPVNLGLWDTAGQEDYDR
LRPLSYPQTDVFLICFSLVSPASFENVRAKWYPEVRHHCPHTPILLVGTKLDLRDDKDTIERLRDK
KLAPITYPQGLAMAREIGSVKYLECSALTQRGLKTVFDEAIRAVLCPPPVKKPGKKCTVF
000000000000000000000000000001111110000000000000000000000000000000
000000000000000000000000000000000000000000000000000000000000000000
000000000000000000000000000000000000000000000000000000000000
>274 TPM_PANST  O61379 Tropomyosin (Allergen Pan s 1) (Pan s I). - Panulirus stimpsoni (Spiny lobster).
MKLEKDNAMDRADTLEQQNKEANIRAEKAEEEVHNLQKRMQQLENDLDQVQESLLKANTQLEEKDK
ALSNAEGEVAALNRRIQLLEEDLERSEERLNTATTKLAEASQAADESERMRKVLENRSLSDEERMD
ALENQLKEARFLAEEADRKYDEVARKLAMVEADLERAEERAETGESKFVELEEELRVVGNNLKSLE
VSEEKANQREEAYKEQIKTLTNKLKAAEARAEFAERSVQKLQKEVDRLEDELVNEKEKYKSITDEL
DQTFSELSGY
000000000000000000000000000000000000000111111111111111110000000000
000000000000000000000000000000000000000000000000000000000000000000
000000000011111111100000000000000000000000000000000000000000000000
000000000000000000000000000000000000000000000000000000000000000000
0000000000
>204 SRPP_HEVBR  O82803 Small rubber particle protein (SRPP) (22 kDa rubber particle protein) (22 kDa RPP) (Latex allergen Hev b 3) (27 kDa natural rubber allergen). - Hevea brasiliensis (Para rubber tree).
MAEEVEEERLKYLDFVRAAGVYAVDSFSTLYLYAKDISGPLKPGVDTIENVVKTVVTPVYYIPLEA
VKFVDKTVDVSVTSLDGVVPPVIKQVSAQTYSVAQDAPRIVLDVASSVFNTGVQEGAKALYANLEP
KAEQYAVITWRALNKLPLVPQVANVVVPTAVYFSEKYNDVVRGTTEQGYRVSSYLPLLPTEKITKV
FGDEAS
011111111111111110000000000011111111110011111111110000000001111111
111100000000000000111111111100000000111111111100000111111111100000
000000000000000000000000001111111111000000000000000000000000000000
000000
>414 CPXA_PSEPU  P00183 Cytochrome P450-cam (EC 1.14.15.1) (Camphor

5-monooxygenase) (P450cam). - Pseudomonas putida.
TTETIQSNANLAPLPPHVPEHLVFDFDMYNPSNLSAGVQEAWAVLQESNVPDLVWTRCNGGHWIAT
RGQLIREAYEDYRHFSSECPFIPREAGEAYDFIPTSMDPPEQRQFRALANQVVGMPVVDKLENRIQ
ELACSLIESLRPQGQCNFTEDYAEPFPIRIFMLLAGLPEEDIPHLKYLTDQMTRPDGSMTFAEAKE
ALYDYLIPIIEQRRQKPGTDAISIVANGQVNGRPITSDEAKRMCGLLLVGGLDTVVNFLSFSMEFL
AKSPEHRQELIERPERIPAACEELLRRFSLVADGRILTSDYEFHGVQLKKGDQILLPQMLSGLDER
ENACPMHVDFSRQKVSHTTFGHGSHLCLGQHLARREIIVTLKEWLTRIPDFSIAPGAQIQHKSGIV
SGVQALPLVWDPATTKAV
000000111111100000000000000000000000000000000000000000000000000000
000000000000000000000001111110111111000000000000000000000000000000
000000000000000111111000000000000000000000000000000011111100000000
000000000000000000000000000011111100000000000000000000000000000000
111111000000000000000000000000000000000000000011111111000000000000
000000000000000000000000000000000000000000000000000000000000000000
000000000000000000
>189 RASN_HUMAN  P01111 Transforming protein N-Ras. - Homo sapiens (Human).
MTEYKLVVVGAGGVGKSALTIQLIQNHFVDEYDPTIEDSYRKQVVIDGETCLLDILDTAGQEEYSA
MRDQYMRTGEGFLCVFAINNSKSFADINLYREQIKRVKDSDDVPMVLVGNKCDLPTRTVDTKQAHE
LAKSYGIPFIETSAKTRQGVEDAFYTLVREIRQYRMKKLNSSDDGTQGCMGLPCVVM
000001111111111111110000001111111111111000000000000000000000000000
000000000000000000000000000000000000000000000000000000000000000000
000000000000000000000000000000000000000000000000000000000
>266 ETXB_STAAU  P01552 Enterotoxin type B precursor (SEB). - Staphylococcus aureus.
MYKRLFISHVILIFALILVISTPNVLAESQPDPKPDELHKSSKFTGLMENMKVLYDDNHVSAINVK
SIDQFLYFDLIYSIKDTKLGNYDNVRVEFKNKDLADKYKDKYVDVFGANYYYQCYFSKKTNDINSH
QTDKRKTCMYGGVTEHNGNQLDKYRSITVRVFEDGKNLLSFDVQTNKKKVTAQELDYLTRHYLVKN
KKLYEFNNSPYETGYIKFIENENSFWYDMMPAPGDKFDQSKYLMMYNDNKMVDSKDVKIEVYLTTK
KK
000000000000000000000000000000000000000000000000000000000000000000
000000000000000000000000000000000000000000000000000000000000000000
000000000000000000000000000000000000000000000000000000000000000000
000000000000000000000000000000000000000000000001111111111111111111
11
>1464 CO1A1_HUMAN P02452 Collagen alpha 1(I) chain precursor. - Homo sapiens (Human).
MFSFVDLRLLLLLAATALLTHGQEEGQVEGQDEDIPPITCVQNGLRYHDRDVWKPEPCRICVCDNG

KVLCDDVICDETKNCPGAEVPEGECCPVCPDGSESPTDQETTGVEGPKGDTGPRGPRGPAGPPGRD
GIPGQPGLPGPPGPPGPPGPPGLGGNFAPQLSYGYDEKSTGGISVPGPMGPSGPRGLPGPPGAPGP
QGFQGPPGEPGEPGASGPMGPRGPPGPPGKNGDDGEAGKPGRPGERGPPGPQGARGLPGTAGLPGM
KGHRGFSGLDGAKGDAGPAGPKGEPGSPGENGAPGQMGPRGLPGERGRPGAPGPAGARGNDGATGA
AGPPGPTGPAGPPGFPGAVGAKGEAGPQGPRGSEGPQGVRGEPGPPGPAGAAGPAGNPGADGQPGA
KGANGAPGIAGAPGFPGARGPSGPQGPGGPPGPKGNSGEPGAPGSKGDTGAKGEPGPVGVQGPPGP
AGEEGKRGARGEPGPTGLPGPPGERGGPGSRGFPGADGVAGPKGPAGERGSPGPAGPKGSPGEAGR
PGEAGLPGAKGLTGSPGSPGPDGKTGPPGPAGQDGRPGPPGPPGARGQAGVMGFPGPKGAAGEPGK
AGERGVPGPPGAVGPAGKDGEAGAQGPPGPAGPAGERGEQGPAGSPGFQGLPGPAGPPGEAGKPGE
QGVPGDLGAPGPSGARGERGFPGERGVQGPPGPAGPRGANGAPGNDGAKGDAGAPGAPGSQGAPGL
QGMPGERGAAGLPGPKGDRGDAGPKGADGSPGKDGVRGLTGPIGPPGPAGAPGDKGESGPSGPAGP
TGARGAPGDRGEPGPPGPAGFAGPPGADGQPGAKGEPGDAGAKGDAGPPGPAGPAGPPGPIGNVGA
PGAKGARGSAGPPGATGFPGAAGRVGPPGPSGNAGPPGPPGPAGKEGGKGPRGETGPAGRPGEVGP
PGPPGPAGEKGSPGADGPAGAPGTPGPQGIAGQRGVVGLPGQRGERGFPGLPGPSGEPGKQGPSGA
SGERGPPGPMGPPGLAGPPGESGREGAPGAEGSPGRDGSPGAKGDRGETGPAGPPGAPGAPGAPGP
VGPAGKSGDRGETGPAGPAGPVGPAGARGPAGPQGPRGDKGETGEQGDRGIKGHRGFSGLQGPPGP
PGSPGEQGPSGASGPAGPRGPPGSAGAPGKDGLNGLPGPIGPPGPRGRTGDAGPVGPPGPPGPPGP
PGPPSAGFDFSFLPQPPQEKAHDGGRYYRADDANVVRDRDLEVDTTLKSLSQQIENIRSPEGSRKN
PARTCRDLKMCHSDWKSGEYWIDPNQGCNLDAIKVFCNMETGETCVYPTQPSVAQKNWYISKNPKD
KRHVWFGESMTDGFQFEYGGQGSDPADVAIQLTFLRLMSTEASQNITYHCKNSVAYMDQQTGNLKK
ALLLKGSNEIEIRAEGNSRFTYSVTVDGCTSHTGAWGKTVIEYKTTKTSRLPIIDVAPLDVGAPDQ
EFGFDVGPVCFL
000000000000000000000000000000000000000000000000000000000000000000
000000000000000000000000000000000000000000000000000000000000000000
000000000000000000000000000000000000000000000000000000000000000000
000000000000000000000000000000000000000000000000000000000000000000
000000000000000000000000000000000000000000000000000000000000000000
000000000000000000000000000000000000000000000000000000000000000000
000000001111111111100000000000000000000000000000000000000000000000
000000000000000000000000000000000000000000000000000000000000000000
000000000000000000000000000000000000000000000000000000000000000000
000000000000000000000000000000000000000000000000000000000000000000
000000000000000000000000000000000000000000000000000000000000000000
000000000000000000000000000000000000000000000000000000000000000000
000000000000000000000000000000000000000000000000000000000000000000
000000000000000000000000000000000000000000000000000000000000000000
000000000000000000000000000000000000000000000000000000000000000000
000000000000000000000000000000000000000000000000000000000000000000

000000000000000000000000000000000000000000000000000000000000000000
000000000000000000000000000000000000000000000000000000000000000000
000000000000000000000000000000000000000000000000000000000000000000
000000000000000000000000000000000000000000000000000000000000000000
000000000000000000000000000000000000000000000000000000000000000000
000000000000000000000000000000000000000000000000000000000000000000
000000000000

>1418 CO2A1_HUMAN P02458 Collagen alpha 1(II) chain precursor [Contains: Chondrocalcin]. - Homo sapiens (Human).

MIRLGAPQSLVLLTLLVAAVLRCQGQDVRQPGPKGQKGEPGDIKDIVGPKGPPGPQGPAGEQGPRG
DRGDKGEKGAPGPRGRDGEPGTLGNPGPPGPPGPPGPPGLGGNFAAQMAGGFDEKAGGAQLGVMQG
PMGPMGPRGPPGPAGAPGPQGFQGNPGEPGEPGVSGPMGPRGPPGPPGKPGDDGEAGKPGKAGERG
PPGPQGARGFPGTPGLPGVKGHRGYPGLDGAKGEAGAPGVKGESGSPGENGSPGPMGPRGLPGERG
RTGPAGAAGARGNDGQPGPAGPPGPVGPAGGPGFPGAPGAKGEAGPTGARGPEGAQGPRGEPGTPG
SPGPAGASGNPGTDGIPGAKGSAGAPGIAGAPGFPGPRGPPDPQGATGPLGPKGQTGKPGIAGFKG
EQGPKGEPGPAGPQGAPGPAGEEGKRGARGEPGGVGPIGPPGERGAPGNRGFPGQDGLAGPKGAPG
ERGPSGLAGPKGANGDPGRPGEPGLPGARGLTGRPGDAGPQGKVGPSGAPGEDGRPGPPGPQGARG
QPGVMGFPGPKGANGEPGKAGEKGLPGAPGLRGLPGKDGETGAEGPPGPAGPAGERGEQGAPGPSG
FQGLPGPPGPPGEGGKPGDQGVPGEAGAPGLVGPRGERGFPGERGSPGAQGLQGPRGLPGTPGTDG
PKGASGPAGPPGAQGPPGLQGMPGERGAAGIAGPKGDRGDVGEKGPEGAPGKDGGRGLTGPIGPPG
PAGANGEKGEVGPPGPAGSAGARGAPGERGETGPPGTSGIAGPPGADGQPGAKGEQGEAGQKGDAG
APGPQGPSGAPGPQGPTGVTGPKGARGAQGPPGATGFPGAAGRVGPPGSNGNPGPPGPPGPSGKDG
PKGARGDSGPPGRAGEPGLQGPAGPPGEKGEPGDDGPSGAEGPPGPQGLAGQRGIVGLPGQRGERG
FPGLPGPSGEPGQQGAPGASGDRGPPGPVGPPGLTGPAGEPGREGSPGADGPPGRDGAAGVKGDRG
ETGAVGAPGAPGPPGSPGPAGPTGKQGDRGEAGAQGPMGPSGPAGARGIQGPQGPRGDKGEAGEPG
ERGLKGHRGFTGLQGLPGPPGPSGDQGASGPAGPSGPRGPPGPVGPSGKDGANGIPGPIGPPGPRG
RSGETGPAGPPGNPGPPGPPGPPGPGIDMSAFAGLGPREKGPDPLQYMRADQAAGGLRQHDAEVDA
TLKSLNNQIESIRSPEGSRKNPARTCRDLKLCHPEWKSGDYWIDPNQGCTLDAMKVFCNMETGETC
VYPNPANVPKKNWWSSKSKEKKHIWFGETINGGFHFSYGDDNLAPNTANVQMTFLRLLSTEGSQNI
TYHCKNSIAYLDEAAGNLKKALLIQGSNDVEIRAEGNSRFTYTALKDGCTKHTGKWGKTVIEYRSQ
KTSRLPIIDIAPMDIGGPEQEFGVDIGPVCFL

000000000000000000000000000000000000000000000000000000000000000000
000000000000000000000000000000000000000000000000000000000000000000
000000000000000000000000000000000000000000000000000000000000000000
000000000000000000000000000000000000000000000000000000000000001111
111111000000000000000000000111111110000000000000000000000000000000
011111111000000000000000000000000000000000000000000000000000000000
000000000000000000000000000000000000000000000000000000000000000000

000000000000000000000000000000000000111111111111111000000000000000
000000000000000000000000000000000000000000000000000000000000000000
000000000000000000000000000000000000000000000000000000000000000000
000000000000000000000000000000000000000000000000000000000000000111
111110000000000000000000000000000000000000000000000000000000000000
000000000000000000000000000000000000000000000000000000000000000000
000000000000000000000000000000000000000000000000000000000000000000
000000000000000000000000000000000000000000000000000000000000000000
000000000000000000000000000000000000000000000000000000000000000000
000000000000000000000000000000000000000000000000000000000000000000
000000000000000000000000000000000000000000000000000000000000000000
000000000000000000000000000000000000000000000000000000000000000000
000000000000000000000000000000000000000000000000000000000000000000
000000000000000000000000000000000000000000000000000000000000000000
00000000000000000000000000000000
>150 GLPA_HUMAN  P02724 Glycophorin A precursor (PAS-2) (Sialoglycoprotein alpha) (MN sialoglycoprotein) (CD235a antigen). - Homo sapiens (Human).
MYGKIIFVLLLSAIVSISASSTTGVAMHTSTSSSVTKSYISSQTNDTHKRDTYAATPRAHEVSEIS
VRTVYPPEEETGERVQLAHHFSEPEITLIIFGVMAGVIGTILLISYGIRRLIKKSPSDVKPLPSPD
TDVPLSSVEIENPETSDQ
000000000000000000000000000000000000000000000000000001111111111100
011111111111100000000000000000000000000000000000000000000000000000
000111111111100000
>178 LACB_BOVIN  P02754 Beta-lactoglobulin precursor (Beta-LG) (Allergen Bos d 5). - Bos taurus (Bovine).
MKCLLLALALTCGAQALIVTQTMKGLDIQKVAGTWYSLAMAASDISLLDAQSAPLRVYVEELKPTP
EGDLEILLQKWENGECAQKKIIAEKTKIPAVFKIDALNENKVLVLDTDYKKYLLFCMENSAEPEQS
LACQCLVRTPEVDDEALEKFDKALKALPMHIRLSFNPTQLEEQCHI
000000000000000000000000000001111111111110000000001111111111111111
111111111110000000000000000001111111111111111111111111111000000000
1111111111111111111110000000000001111111111111
>362 OMPF_ECOLI  P02931 Outer membrane protein F precursor (Porin ompF) (Outer membrane protein 1A) (Outer membrane protein IA) (Outer membrane protein B). - Escherichia coli.
MMKRNILAVIVPALLVAGTANAAEIYNKDGNKVDLYGKAVGLHYFSKGNGENSYGGNGDMTYARLG
FKGETQINSDLTGYGQWEYNFQGNNSEGADAQTGNKTRLAFAGLKYADVGSFDYGRNYGVVYDALG
YTDMLPEFGGDTAYSDDFFVGRVGGVATYRNSNFFGLVDGLNFAVQYLGKNERDTARRSNGDVGG

SISYEYEGFGIVGAYGAADRTNLQEAQPLGNGKKAEQWATGLKYDANNIYLAANYGETRNATPITN
KFTNTSGFANKTQDVLLVAQYQFDFGLRPSIAYTKSKAKDVEGIGDVDLVNYFEVGATYYFNKNMS
TYVDYIINQIDSDNKLGVGSDDTVAVGIVYQF
000000000000000000000000000000000000000000000000000000000000000000
111111111111111111110000000000000000000000000000001111111111111111
111111111111111111111111000000000000000000000000000000000000000000
000000000000000000000000000000000000000000000000000000000000000000
001111111111111111111100000000001111111111111111111100000000001111
11111111111111110000000000000000
>170 FMC1_ECOLI P02971 CFA/I fimbrial subunit B precursor (Colonization factor antigen I subunit B) (CFA/I pilin) (CFA/I antigen). - Escherichia coli.
MKFKKTIGAMALTTMFVAVSASAVEKNITVTASVDPVIDLLQADGNALPSAVKLAYSPASKTFESY
RVMTQVHTNDATKKVIVKLADTPQLTDVLNSTVQMPISVSWGGQVLSTTAKEFEAAALGYSASGVN
GVSSSQELVISAAPKTAGTAPTAGNYSGVVSLVMTLGS
000000000000000000000000000000000001111111100000000000000000000000
000000000000000000000000000000000000000000000000000000000000000000
00000000000000000000000000000000000000
>508 VL1_HPV1A P03099 Major capsid protein L1. - Human papillomavirus type 1a.
MYNVFQMAVWLPAQNKFYLPPQPITRILSTDEYVTRTNLFYHATSERLLLVGHPLFEISSNQTVTI
PKVSPNAFRVFRVRFADPNRFAFGDKAIFNPETERLVWGLRGIEIGRGQPLGIGITGHPLLNKLDD
AENPTNYINTHANGDSRQNTAFDAKQTQMFLVGCTPASGEHWTSSRCPGEQVKLGDCPRVQMIESV
IEDGDMMDIGFGAMDFAALQQDKSDVPLDVVQATCKYPDYIRMNHEAYGNSMFFFARREQMYTRHF
FTRGGSVGDKEAVPQSLYLTADAEPRTTLATTNYVGTPSGSMVSSDVQLFNRSYWLQRCQGQNNGI
CWRNQLFITVGDNTRGTSLSISMKNNASTTYSNANFNDFLRHTEEFDLSFIVQLCKVKLTPENLAY
IHTMDPNILEDWQLSVSQPPTNPLEDQYRFLGSSLAAKCPEQAPPEPQTDPYSQYKFWEVDLTERM
SEQLDQFPLGRKFLYQSGMTQRTATSSTTKRKTVRVSTSAKRRRKA
000000000000000000000000000000000000000000000000000000000000000000
000000000000000000000000000000000000000000000000000000000000000000
000000000000000000000000000000000000000000000000000000000000000000
000000000000000000000000000000000000000000000000000000000000000000
000000000000000000000000000000000000000000000000000000000000000000
000000000000000000000000000000000000000000000000000000000000000000
000000000000000000000000000000111111111111111111111100000000000000
0000000000000000000000000000000000000000000000
>500 VL1_HPV6B P69899 Major capsid protein L1. - Human papillomavirus type 6b.

MWRPSDSTVYVPPPNPVSKVVATDAYVTRTNIFYHASSSRLLAVGHPYFSIKRANKTVVPKVSGYQ
YRVFKVVLPDPNKFALPDSSLFDPTTQRLVWACTGLEVGRGQPLGVGVSGHPFLNKYDDVENSGSG
GNPGQDNRVNVGMDYKQTQLCMVGCAPPLGEHWGKGKQCTNTPVQAGDCPPLELITSVIQDGDMVD
TGFGAMNFADLQTNKSDVPIDICGTTCKYPDYLQMAADPYGDRLFFFLRKEQMFARHFFNRAGEVG
EPVPDTLIIKGSGNRTSVGSSIYVNTPSGSLVSSEAQLFNKPYWLQKAQGHNNGICWGNQLFVTVV
DTTRSTNMTLCASVTTSSTYTNSDYKEYMRHVEEYDLQFIFQLCSITLSAEVMAYIHTMNPSVLED
WNFGLSPPPNGTLEDTYRYVQSQAITCQKPTPEKEKPDPYKNLSFWEVNLKEKFSSELDQYPLGRK
FLLQSGYRGRSSIRTGVKRPAVSKASAAPKRKRAKTKR
000000000000000000000000000000000000000000000000000000000000000000
000000000000000000000000000000000000000000000000000000000000000000
000000000000000000000000000000000000000000000000000000000000111111
111111111111111111111100000000000000000000000000000000000000000000
000000000000000000000000000000000000000000000000000000000000000000
000000000000000000000000000000000000000000000000000000000000000000
000000000000000000001111111111111111111100000000000000000000000000
00000000000000000000000000000000000000
>531 VL1_HPV16    P03101 Major capsid protein L1. - Human papillomavirus type 16.
MQVTFIYILVITCYENDVNVYHIFFQMSLWLPSEATVYLPPVPVSKVVSTDEYVARTNIYYHAGTS
RLLAVGHPYFPIKKPNNNKILVPKVSGLQYRVFRIHLPDPNKFGFPDTSFYNPDTQRLVWACVGVE
VGRGQPLGVGISGHPLLNKLDDTENASAYAANAGVDNRECISMDYKQTQLCLIGCKPPIGEHWGKG
SPCTNVAVNPGDCPPLELINTVIQDGDMVDTGFGAMDFTTLQANKSEVPLDICTSICKYPDYIKMV
SEPYGDSLFFYLRREQMFVRHLFNRAGAVGENVPDDLYIKGSGSTANLASSNYFPTPSGSMVTSDA
QIFNKPYWLQRAQGHNNGICWGNQLFVTVVDTTRSTNMSLCAAISTSETTYKNTNFKEYLRHGEEY
DLQFIFQLCKITLTADVMTYIHSMNSTILEDWNFGLQPPPGGTLEDTYRFVTSQAIACQKHTPPAP
KEDPLKKYTFWEVNLKEKFSADLDQFPLGRKFLLQAGLKAKPKFTLGKRKATPTTSSTSTTAKRKK
RKL
000000000000000000000000000000000000000000000000000000000000000000
000000000000000000000000000000000000000011111111111111111111000000
000000000000000000111111111111111011111111111111111111111111111111
111111111111111111000000111111111111111111111111111111111111111000
000000000000000000000000001111111111111111111100000000000000000000
111111111111111011111111111111111111000000000000000000000000000000
000000000000000000000000000000000000000011111111111111111111111111
111111111000000001111111111111111111111111111111111111000000000000
000
>505 VL1_CRPVK   P03102 Major capsid protein L1. - Cottontail rabbit (shope) papillomavirus (strain Kansas) (CRPV).

MAVWLSTQNKFYLPPQPVTKIPSTDEYVTRTNVFYYASSDRLLTVGHPYYEIRDKGTMLVPKVSPN
QYRVFRIKLPDPNKFAFGDKQLYDPEKERLVWCLRGIEVNRGQPLGVSVTGNPIFNKFDDVENPTK
YYNNHADQQDYRKSMAFDPKQVQLLMLGCVPATGEHWAQAKQCAEDPPQQTDCPPIELVNTVIEDG
DMCEIGFGAMDHKTLQASLSEVPLELAQSISKYPDYLKMQKDQFGDSMFFYARREQMYARHFFSRA
GGDKENVKSRAYIKRTQMQGEANANIATDNYCITPSGSLVSSDSQVFNRAYWLQKAQGMNNGVCWD
NQIFVTVVDNTRGTILSLVTKSKEQIKKTHGKTVHFSSYLRHVEEYELQFVLQLCKVKLTPENLSY
LHSMHPTIIDNWQLSVSAQPSGTLEDQYRYLQSIATKCPPPEPPKENTDPYKNYKFWEVDLSEKLS
DQLDQYPLGRKFLNQSGLQRIGTKRPAPAPVSIVKSSKRKRRT
000000000000000000000000000000000000000000000000000000000000000000
000000000000000000000000000000000000000000000000000000000000000000
000000000000000000000000000000000000000000000000000000000000000000
000000000000000000000000000000000000000000000000000000000000000000
000000001111110000000000000000000000000000000000000000000000000000
000000000000000000000000000000000000000000000000000000000000000000
000000000000000000000000000000000000000000000000000000000000000000
0000000000000000000000000000000000000000000
>495 VL1_BPV1    P03103 Major capsid protein L1. - Bovine
papillomavirus type 1.
MALWQQGQKLYLPPTPVSKVLCSETYVQRKSIFYHAETERLLTIGHPYYPVSIGAKTVPKVSANQY
RVFKIQLPDPNQFALPDRTVHNPSKERLVWAVIGVQVSRGQPLGGTVTGHPTFNALLDAENVNRKV
TTQTTDDRKQTGLDAKQQQILLLGCTPAEGEYWTTARPCVTDRLENGACPPLELKNKHIEDGDMME
IGFGAANFKEINASKSDLPLDIQNEICLYPDYLKMAEDAAGNSMFFFARKEQVYVRHIWTRGGSEK
EAPTTDFYLKNNKGDATLKIPSVHFGSPSGSLVSTDNQIFNRPYWLFRAQGMNNGIAWNNLLFLTV
GDNTRGTNLTISVASDGTPLTEYDSSKFNVYHRHMEEYKLAFILELCSVEITAQTVSHLQGLMPSV
LENWEIGVQPPTSSILEDTYRYIESPATKCASNVIPAKEDPYAGFKFWNIDLKEKLSLDLDQFPLG
RRFLAQQGAGCSTVRKRRISQKTSSKPAKKKKK
000000000000000000000000000000000000000000000000000000000000000000
000000000000000000000000000000000000000000000000000000000000000000
000000000000000000000000000000000000000000000000000000000000000000
000000000000000000000000000000000000000000000000000000000000000000
000011111100000000000000000000000000000000000000000000000000000000
000000000000000000000000000000000000000000000000000000000000000000
000000000000000000000000000000000000000000000000000000000000000000
000000000000000000000000000000000
>507 VL2_HPV1A    P03105 Minor capsid protein L2. - Human papillomavirus
type 1a.
MYRLRRKRAAPKDIYPSCKISNTCPPDIQNKIEHTTIADKILQYGSLGVFLGGLGIGTARGSGGRI
GYTPLGEGGGVRVATRPTPVRPTIPVETVGPSEIFPIDVVDPTGPAVIPLQDLGRDFPIPTVQVIA

EIHPISDIPNIVASSTNEGESAILDVLRGNATIRTVSRTQYNNPSFTVASTSNISAGEASTSDIVF
VSNGSGDRVVGEDIPLVELNLGLETDTSSVVQETAFSSSTPIAERPSFRPSRFYNRRLYEQVQVQD
PRFVEQPQSMVTFDNPAFEPELDEVSIIFQRDLDALAQTPVPEFRDVVYLSKPTFSREPGGRLRVS
RLGKSSTIRTRLGTAIGARTHFFYDLSSIAPEDSIELLPLGEHSQTTVISSNLGDTAFIQGETAED
DLEVISLETPQLYSEEELLDTNESVGENLQLTITNSEGEVSILDLTQSRVRPPFGTEDTSLHVYYP
NSSKGTPIINPEESFTPLVIIALNNSTGDFELHPSLRKRRKRAYV
000000000000000000000000000000000000000000000000000000000000000000
000000000000000000000000000000000001111110000000000000000000000000
000000000000000000000000000000000000000000000000000000000000000000
000000000000000000000000000000000000000000000000000000000000000000
000000000000000000000000000000000000000000000000000000000000000000
000000000000000000000000000000000000000000000000000000000000000000
000000000000000000000000000000000000000000000000000000000000000000
000000000000000000000000000000000000000000000
>459 VL2_HPV6B    P03106 Minor capsid protein L2. - Human papillomavirus type 6b.
MAHSRARRRKRASATQLYQTCKLTGTCPPDVIPKVEHNTIADQILKWGSLGVFFGGLGIGTGSGTG
GRTGYVPLQTSAKPSITSGPMARPPVVVEPVAPSDPSIVSLIEESAIINAGAPEIVPPAHGGFTIT
SSETTTPAILDVSVTSHTTTSIFRNPVFTEPSVTQPQPPVEANGHILISAPTVTSHPIEEIPLDTF
VVSSSDSGPTSSTPVPGTAPRPRVGLYSRALHQVQVTDPAFLSTPQRLITYDNPVYEGEDVSVQFS
HDSIHNAPDEAFMDIIRLHRPAIASRRGLVRYSRIGQRGSMHTRSGKHIGARIHYFYDISPIAQAA
EEIEMHPLVAAQDDTFDIYAESFEPGINPTQHPVTNISDTYLTSTPNTVTQPWGNTTVPLSLPNDL
FLQSGPDITFPTAPMGTPFSPVTPALPTGPVFITGSGFYLHPAWYFARKRRKRIPLFFSDVAA
000000000000000000000000000000000000000000000000000000000000000000
000000000000000000000000000000000000000111111111111111111111110000
000000000000000000111111111111111111111110111111111111111111111111
101111111111111111100000000000000000000000000000000000000000000000
000000000000000000000000000000000000000000000000000000000000000000
000000000000000000001111111111111111100000000000000000000000000000
000000000000000000000000000000000000000000000000000000000000000
>473 VL2_HPV16    P03107 Minor capsid protein L2. - Human papillomavirus type 16.
MRHKRSAKRTKRASATQLYKTCKQAGTCPPDIIPKVEGKTIAEQILQYGSMGVFFGGLGIGTGSGT
GGRTGYIPLGTRPPTATDTLAPVRPPLTVDPVGPSDPSIVSLVEETSFIDAGAPTSVPSIPPDVSG
FSITTSTDTTPAILDINNTVTTVTTHNNPTFTDPSVLQPPTPAETGGHFTLSSSTISTHNYEEIPM
DTFIVSTNPNTVTSSTPIPGSRPVARLGLYSRTTQQVKVVDPAFVTTPTKLITYDNPAYEGIDVDN
TLYFSSNDNSINIAPDPDFLDIVALHRPALTSRRTGIRYSRIGNKQTLRTRSGKSIGAKVHYYYDL
STIDPAEEIELQTITPSTYTTTSHAASPTSINNGLYDIYADDFITDTSTTPVPSVPSTSLSGYIPA

NTTIPFGGAYNIPLVSGPDIPINITDQAPSLIPIVPGSPQYTIIADAGDFYLHPSYYMLRKRRKRL
PYFFSDVSLAA
000000000000000000000000000000000000000000000000000000000000000000
000000000000000000000000000000000000000001111111111111000000000000
000000000000000000000000000000000000000000000000000000000000000000
000000000000000000000000000000000000000000000000000000000000000000
000000000000000000000000000000000000000000000000000000000000000000
000000000000000000000000000000000000000000000000000000000000000000
000000000000000000000000000000000000000000000000000000000000000000
00000000000
>649 VE1_HPV16   P03114 Replication protein E1. - Human papillomavirus type 16.
MADPAGTNGEEGTGCNGWFYVEAVVEKKTGDAISDDENENDSDTGEDLVDFIVNDNDYLTQAETET
AHALFTAQEAKQHRDAVQVLKRKYLVSPLSDISGCVDNNISPRLKAICIEKQSRAAKRRLFESEDS
GYGNTEVETQQMLQVEGRHETETPCSQYSGGSGGGCSQYSSGSGGEGVSERHTICQTPLTNILNVL
KTSNAKAAMLAKFKELYGVSFSELVRPFKSNKSTCCDWCIAAFGLTPSIADSIKTLLQQYCLYLHI
QSLACSWGMVVLLLVRYKCGKNRETIEKLLSKLLCVSPMCMMIEPPKLRSTAAALYWYKTGISNIS
EVYGDTPEWIQRQTVLQHSFNDCTFELSQMVQWAYDNDIVDDSEIAYKYAQLADTNSNASAFLKSN
SQAKIVKDCATMCRHYKRAEKKQMSMSQWIKYRCDRVDDGGDWKQIVMFLRYQGVEFMSFLTALKR
FLQGIPKKNCILLYGAANTGKSLFGMSLMKFLQGSVICFVNSKSHFWLQPLADAKIGMLDDATVPC
WNYIDDNLRNALDGNLVSMDVKHRPLVQLKCPPLLITSNINAGTDSRWPYLHNRLVVFTFPNEFPF
DENGNPVYELNDKNWKSFFSRTWSRLSLHEDEDKENDGDSLPTFKCVSGQNTNTL
000000000000000000000000000000000000000000000000000000000000000000
000000000000000000000000000000000000000000000000000000000000000000
000000000000000000000000000000000000000000000000000000000000000000
000000000000000000000000000000000000000000000000000000000000000000
000000000000000000000000000000000000000000000000000000000000000000
000000000000000000000000000000000000000000000000000000000000000000
000000000000000000000000000000000000000000000000000000000000000000
000000000000000000000000000000000000000001111111111111111111100000
000000000000000000000000000000000000000000000000000000000000000001
1111111111111111100000000000000000000000000000000000000
>365 VE2_HPV16   P03120 Regulatory protein E2. - Human papillomavirus type 16.
METLCQRLNVCQDKILTHYENDSTDLRDHIDYWKHMRLECAIYYKAREMGFKHINHQVVPTLAVSK
NKALQAIELQLTLETIYNSQYSNEKWTLQDVSLEVYLTAPTGCIKKHGYTVEVQFDGDICNTMHYT
NWTHIYICEEASVTVVEGQVDYYGLYYVHEGIRTYFVQFKDDAEKYSKNKVWEVHAGGQVILCPTS
VFSSNEVSSPEIIRQHLANHPAATHTKAVALGTEETQTTIQRPRSEPDTGNPCHTTKLLHRDSVDS

APILTAFNSSHKGRINCNSNTTPIVHLKGDANTLKCLRYRFKKHCTLYTAVSSTWHWTGHNVKHKS
AIVTLTYDSEWQRDQFLSQVKIPKTITVSTGFMSI
000000000000000000000000000000000000000000000000000000000000000000
000000000000000000000000000000000000000000000000000000111111111111
111111110000000000000000000000000000000000000000111111111111111111
110000000000000000000000000000000000000000111111111111111111110000
000000000000000000000000000000000000000000000000000000000000000000
00000000000000000000000000000000000
>158 VE6_HPV16   P03126 E6 protein. - Human papillomavirus type 16.
MHQKRTAMFQDPQERPRKLPQLCTELQTTIHDIILECVYCKQQLLRREVYDFAFRDLCIVYRDGNP
YAVCDKCLKFYSKISEYRHYCYSLYGTTLEQQYNKPLCDLLIRCINCQKPLCPEEKQRHLDKKQRF
HNIRGRWTGRCMSCCRSSRTRRETQL
000111111111111111000000000000000000000000000000000000000000000000
000000000000000000000000000000000000000000000000000000000000000000
00000000000000000000000000
>504 COA3_AAV2   P03135 Probable coat protein 3. - Adeno-associated virus 2 (AAV2).
MATGSGAPMADNNEGADGVGNSSGNWHCDSTWMGDRVITTSTRTWALPTYNNHLYKQISSQSGASN
DNHYFGYSTPWGYFDFNRFHCHFSPRDWQRLINNNWGFRPKRLNFKLFNIQVKEVTQNDGTTTIAN
NLTSTVQVFTDSEYQLPYVLGSAHQGCLPPFPADVFMVPQYGYLTLNNGSQAVGRSSFYCLEYFPS
QMLRTGNNFTFSYTFEDVPFHSSYAHSQSLDRLMNPLIDQYLYYLSRTNTPSGTTTQSRLQFSQAG
ASDIRDQSRNWLPGPCYRQQRVSKTSADNNNSEYSWTGATKYHLNGRDSLVNPAMASHKDDEEKFF
PQSGVLIFGKQGSEKTNVNIEKVMITDEEEIGTTNPVATEQYGSVSTNLQRGNRQAATADVNTQGV
LPGMVWQDRDVYLQGPIWAKIPHTDGHFHPSPLMGGFGLKHPPPQILIKNTPVPANPSTTFSAAKF
ASFITQYSTGHGQRGDRVGAAEGKQQTLESRNSVHFQLQQVC
000000000000000000000000000000000000001111111111111111111100000000
000000000000000000000000000000000000000000000000000000000000000000
000000000000000000000000000000000000000000000000000000000000000000
000000000000000000000000000000000000000000000000000000000000000011
111111111111111111111111110000000000000000000000000000000000000000
000000000000000000000000000000000000000000000000000000000000000000
000000000000000000000000000000000000000000000000000000000000000000
000000000000000000000000000000000000000000
>183 CORA_HPBVY   P03146 Core antigen. - Hepatitis B virus (subtype ayw).
MDIDPYKEFGATVELLSFLPSDFFPSVRDLLDTASALYREALESPEHCSPHHTALRQAILCWGELM
TLATWVGVNLEDPASRDLVVSYVNTNMGLKFRQLLWFHISCLTFGRETVIEYLVSFGVWIRTPPAY
RPPNAPILSTLPETTVVRRRGRSPRRRTPSPRRRRSQSPRRRRSQSRESQC
000000000000000000000000000000000000000000000000000000000000000000

00000001111111111000000000000000000000000000000000000000000000000
000000000000000000000000000000000000000000000000000

>641 EBN1_EBV   P03211 Epstein-Barr nuclear antigen-1 (EBNA-1). - Epstein-Barr virus (strain B95-8) (HHV-4) (Human herpesvirus 4).

MSDEGPGTGPGNGLGEKGDTSGPEGSGGSGPQRRGGDNHGRGRGRGRGGGRPGAPGGSGSGPRH
RDGVRRPQKRPSCIGCKGTHGGTGAGAGAGGAGAGGAGAGGGAGAGGGAGGAGGAGGAGAGGGAGA
GGGAGGAGGAGAGGGAGAGGGAGGAGAGGGAGGAGGAGAGGGAGAGGGAGGAGAGGGAGGAGGAGA
GGGAGAGGAGGAGGAGAGGAGAGGGAGGAGAGGAGAGGAGAGGAGAGGAGGAGAGGAGGAGAG
GAGGAGAGGGAGGAGAGGGAGGAGAGGAGGAGAGGAGGAGAGGAGGAGAGGGAGAGGAGAGGGGRG
RGGSGGRGRGGSGGRGRGGSGGRRGRGRERARGGSRERARGRGRGRGEKRPRSPSSQSSSSGSPPR
RPPPGRRPFFHPVGEADYFEYHQEGGPDGEPDVPPGAIEQGPADDPGEGPSTGPRGQGDGGRRKKG
GWFGKHRGQGGSNPKFENIAEGLRALLARSHVERTTDEGTWVAGVFVYGGSKTSLYNLRRGTALAI
PQCRLTPLSRLPFGMAPGPGPQPGPLRESIVCYFMVFLQTHIFAEVLKDAIKDLVMTKPAPTCNIR
VTVCSFDDGVDLPPWFPPMVEGAAAEGDDGDDGDEGGDGDEGEEGQE

000000000000000000000000000000000000000000000000000111111111111111
111111111111111111111111111111111110000000000000000000000000000000
000000000000000000000000000000000000000000111111111111111111110000
000000000000000000000000000000000000000000000000000000000000000000
000000000000000000000000000000000000000000000001111111111111111111
100000000000000000000000000000011111111111111111111111111111111111
111111111111111000000000000000000000000000000000000000000000000000
000000000000000000000000000000000000000111111111111111111110000000
000000000000000000000000000000000000000000000000000000000000000000
000000000000000000000000000000000000000000000000000000000000000000
00000000000000000000000000000000000000000000000

>198 VCO7_ADE05  P68951 Major core protein precursor (Protein VII) (pVII). - Human adenovirus 5 (HAdV-5).

MSILISPSNNTGWGLRFPSKMFGGAKKRSDQHPVRVRGHYRAPWGAHKRGRTGRTTVDDAIDAVVE
EARNYTPTPPPVSTVDAAIQTVVRGARRYAKMKRRRRRVARRHRRRPGTAAQRAAAALLNRARRTG
RRAAMRAARRLAAGIVTVPPRSRRRAAAAAAAAISAMTQGRRGNVYWVRDSVSGLRVPVRTRPPRN

000000000000000000000000000000000000000000000000000000000000000011
111111111111000000000000000000000000000000000000000000000000000000
000000000000000000000000000000000000000000000000000000000000000000

>2332 POLG_FMDVO  P03305 Genome polyprotein [Contains: Leader protease (EC 3.4.22.46) (P20A); Coat protein VP4; Coat protein VP2; Coat protein VP3; Coat protein VP1; Core protein p12; Core protein p34; Core protein p14; Genome- linked protein VPG; Protease (EC 3.4.22.-); RNA-directed RNA polymerase (EC 2.7.7.48)]. - Foot-and-mouth disease virus (strain O1) (Aphthovirus O) (FMDV).

MNTTDCFIALVQAIREIKALFLSRTTGKMELTLYNGEKKTFYSRPNNHDNCWLNAILQLFRYVEEP
FFDWVYSSPENLTLEAIKQLEDLTGLELHEGGPPALVIWNIKHLLHTGIGTASRPSEVCMVDGTDM
CLADFHAGIFLKGQEHAVFACVTSNGWYAIDDEDFYPWTPDPSDVLVFVPYDQEPLNGEWKAKVQR
KLKGAGQSSPATGSQNQSGNTGSIINNYYMQQYQNSMDTQLGDNAISGGSNEGSTDTTSTHTTNTQ
NNDWFSKLASSAFSGLFGALLADKKTEETTLLEDRILTTRNGHTTSTTQSSVGVTYGYATAEDFVS
GPNTSGLETRVVQAERFFKTHLFDWVTSDSFGRCHLLELPTDHKGVYGSLTDSYAYMRNGWDVEVT
AVGNQFNGGCLLVAMVPELYSIQKRELYQLTLFPHQFINPRTNMTAHITVPFVGVNRYDQYKVHKP
WTLVVMVVAPLTVNTEGAPQIKVYANIAPTNVHVAGEFPSKEGIFPVACSDGYGGLVTTDPKTADP
VYGKVFNPPRNQLPGRFTNLLDVAEACPTFLRFEGGVPYVTTKTDSDRVLAQFDMSLAAKQMSNTF
LAGLAQYYTQYSGTINLHFMFTGPTDAKARYMVAYAPPGMEPPKTPEAAAHCIHAEWDTGLNSKFT
FSIPYLSAADYAYTASGVAETTNVQGWVCLFQITHGKADGDALVVLASAGKDFELRLPVDARAETT
SAGESADPVTTTVENYGGETQIQRRQHTDVSFIMDRFVKVTPQNQINILDLMQIPSHTLVGALLRA
STYYFSDLEIAVKHEGDLTWVPNGAPEKALDNTTNPTAYHKAPLTRLALPYTAPHRVLATVYNGEC
RYNRNAVPNLRGDLQVLAQKVARTLPTSFNYGAIKATRVTELLYRMKRAETYCPRPLLAIHPTEAR
HKQKIVAPVKQTLNFDLLKLAGDVESNPGPFFFSDVRSNFSKLVETINQMQEDMSTKHGPDFNRLV
SAFEELAIGVKAIRTGLDEAKPWYKLIKLLSRLSCMAAVAARSKDPVLVAIMLADTGLEILDSTFV
VKKISDSLSSLFHVPAPVFSFGAPVLLAGLVKVASSFFRSTPEDLERAEKQLKARDINDIFAILKN
GEWLVKLILAIRDWIKAWIASEEKFVTMTDLVPGILEKQRDLNDPSKYKEAKEWLDNARQACLKSG
NVHIANLCKVVAPAPSKSRPEPVVVCLRGKSGQGKSFLANVLAQAISTHFTGRIDSVWYCPPDPDH
FDGYNQQTVVVMDDLGQNPDGKDFKYFAQMVSTTGFIPPMASLEDKGKPFNSKVIIATTNLYSGFT
PRTMVCPDALNRRFHFDIDVSAKDGYKINSKLDIIKALEDTHANPVAMFQYDCALLNGMAVEMKRM
QQDMFKPQPPLQNVYQLVQEVIDRVELHEKVSSHPIFKQISIPSQKSVLYFLIEKGQHEAAIEFFE
GMVHDSIKEELRPLIQQTSFVKRAFKRLKENFEIVALCLTLLANIVIMIRETRKRQKMVDDAVNEY
IEKANITTDDKTLDEAEKSPLETSGASTVGFRERTLPGQKACDDVNSEPAQPVEEQPQAEGPYAGP
LERQKPLKVRAKLPQQEGPYAGPMERQKPLKVKAKAPVVKEGPYEGPVKKPVALKVKAKNLIVTES
GAPPTDLQKMVMGNTKPVELILDGKTVAICCATGVFGTAYLVPRHLFAEKYDKIMVDGRAMTDSDY
RVFEFEIKVKGQDMLSDAALMVLHRGNRVRDITKHFRDTARMKKGTPVVGVINNADVGRLIFSGEA
LTYKDIVVCMDGDTMPGLFAYRAATKAGYCGGAVLAKDGADTFIVGTHSAGGNGVGYCSCVSRSML
LKMKAHIDPEPHHEGLIVDTRDVEERVHVMRKTKLAPTVAHGVFNPEFGPAALSNKDPRLNEGVVL
DEVIFSKHKGDTKMSEEDKALFRRCAADYASRLHSVLGTANAPLSIYEAIKGVDGLDAMEPDTAPG
LPWALQGKRRGALIDFENGTVGPEVEAALKLMEKREYKFVCQTFLKDEIRPLEKVRAGKTRIVDVL
PVEHILYTRMMIGRFCAQMHSNNGPQIGSAVGCNPDVDWQRFGTHFAQYRNVWDVDYSAFDANHCS
DAMNIMFEEVFRTEFGFHPNAEWILKTLVNTEHAYENKRITVGGGMPSGCSATSIINTILNNIYVL
YALRRHYEGVELDTYTMISYGDDIVVASDYDLDFEALKPHFKSLGQTITPADKSDKGFVLGHSITD
VTFLKRHFHMDYGTGFYKPVMASKTLEAILSFARRGTIQEKLISVAGLAVHSGPDEYRRLFEPFQG
LFEIPSYRSLYLRWVNAVCGDA
000000000000000000000000000000000000000000000000000000000000000000
000000000000000000000000000000000000000000000000000000000000000000

000000000000000000000000000000000000000000000000000000000000000000
000000000000000000000000000000000000000000000000000000000000000000
000000000000000000000000000000000000000000000000000000000000000000
000000000000000000000000000000000000000000000000000000000000000000
000000000000000000000000000000000000000000000000000000000000000000
000000000000000000000000000000000000000000000000000000000000000000
000000000000000000000000000000000000000000000000000000000000000000
000000000000000000000000000000000000000000000000000000000000000000
000000000000000000000000000000000000000000000000000000000000000000
000000000000000000000000000000000000000000000000000000000000000000
000000000000000000000000000000000000000000000000000000000000000000
111111111111111111111111110000000000000000000000000000000000000000
000000000000000000000000000000000000000000000000000000000000000000
000000000000000000000000000000000000000000000000000000000000000000
000000000000000000000000000000000000000000000000000000000000000000
000000000000000000000000000000000000000000000000000000000000000000
000000000000000000000000000000000000000000000000000000000000000000
000000000000000000000000000000000000000000000000000000000000000000
000000000000000000000000000000000000000000000000000000000000000000
000000000000000000000000000000000000000000000000000000000000000000
000000000000000000000000000000000000000000000000000000000000000000
000000000000000000000000000000000000000000000000000000000000000000
000000000000000000000000000000000000000000000000000000000000000000
000000000000000000000000000000000000000000000000000000000000000000
000000000000000000000000000000000000000000000000000000000000000000
000000000000000000000000000000000000000000000000000000000000000000
000000000000000000000000000000000000000000000000000000000000000000
000000000000000000000000000000000000000000000000000000000000000000
000000000000000000000000000000000000000000000000000000000000000000
000000000000000000000000000000000000000000000000000000000000000000
000000000000000000000000000000000000000000000000000000000000000000
000000000000000000000000000000000000000000000000000000000000000000
000000000000000000000000000000000000000000000000000000000000000000
0000000000000000000000
>308 YPX1_BLVJ P03412 Hypothetical PXBL-I protein (Fragment). - Bovine leukemia virus (Japanese isolate BLV-1) (BLV).
ASVVGWGPHSLHACPALVLSNDVTIDAWCPLCGPHERLQFERIDTTLTCETHRINWTADGRPCGLN
GTLFPRLHVSETRPQGPRRLWINCPLPAVRAQPGPVSLSPFERSPFQPYQCQLPSASSDGCPIIGH

GLLPWNNLVTHPVLGKVLILNQMANFSLLPSFDTLLVDPLRLSVFAPDTRGAIRYLSTLLTLCPAT
CILPLGEPFSPNVPICRFPRDSNEPPLSEFELPPIQTPGLSWSVPAIDLFLTGPPSPCDRLHVWSS
PQALQRFLHDPTLTWSELVASRKIRLDSPLKLQLLENEWLSRLF
000000000000000000000000000000000000000000000000000000000000000000
000000000000000000000000000000000000000000000000000000000000000111
111111111111111110000000000000000000000000000000000000000000000000
000000000000000000000000000000000000000000000000000000000000011111
11111111111111100000000000000000000000000000
>501 VL1_HPV11    P04012 Major capsid protein L1. - Human papillomavirus type 11.
MWRPSDSTVYVPPPNPVSKVVATDAYVKRTNIFYHASSSRLLAVGHPYYSIKKVNKTVVPKVSGYQ
YRVFKVVLPDPNKFALPDSSLFDPTTQRLVWACTGLEVGRGQPLGVGVSGHPLLNKYDDVENSGGY
GGNPGQDNRVNVGMDYKQTQLCMVGCAPPLGEHWGKGTQCSNTSVQNGDCPPLELITSVIQDGDMV
DTGFGAMNFADLQTNKSDVPLDICGTVCKYPDYLQMAADPYGDRLFFYLRKEQMFARHFFNRAGTV
GEPVPDDLLVKGGNNRSSVASSIYVHTPSGSLVSSEAQLFNKPYWLQKAQGHNNGICWGNHLFVTV
VDTTRSTNMTLCASVSKSATYTNSDYKEYMRHVEEFDLQFIFQLCSITLSAEVMAYIHTMNPSVLE
DWNFGLSPPPNGTLEDTYRYVQSQAITCQKPTPEKEKQDPYKDMSFWEVNLKEKFSSELDQFPLGR
KFLLQSGYRGRTSARTGIKRPAVSKPSTAPKRKRTKTKK
000000000000000000000000000000000000000000000000000000000000000000
000000000000000000000000000000000000000000000000000000000000000000
000000000000000000000000000000000000000000000000000000000000000000
000000000000000000000000000000000000000000000000000000000000000000
000000000000000000000000000000000000000000000000000000000000000000
000000000000000000000000000000000000000000000000000000000000000000
000000000000000000000111111111111111111111000000000000000000000000
000000000000000000000000000000000000000
>455 VL2_HPV11    P04013 Minor capsid protein L2. - Human papillomavirus type 11.
MKPRARRRKRASATQLYQTCKATGTCPPDVIPKVEHTTIADQILKWGSLGVFFGGLGIGTGAGSGG
RAGYIPLGSSPKPAITGGPAARPPVLVEPVAPSDPSIVSLIEESAIINAGAPEVVPPTQGGFTITS
SESTTPAILDVSVTNHTTTSVFQNPLFTEPSVIQPQPPVEASGHILISAPTITSQHVEDIPLDTFV
VSSSDSGPTSSTPLPRAFPRPRVGLYSRALQQVQVTDPAFLSTPQRLVTYDNPVYEGEDVSLQFTH
ESIHNAPDEAFMDIIRLHRPAITSRRGLVRFSRIGQRGSMYTRSGQHIGARIHYFQDISPVTQAAE
EIELHPLVAAENDTFDIYAEPFDPIPDPVQHSVTQSYLTSTPNTLSQSWGNTTVPLSIPSDWFVQS
GPDITFPTASMGTPFSPVTPALPTGPVFITGSDFYLHPTWYFARRRRKRIPLFFTDVAA
000000000000000000000000000000000000000000000000111111111111111111
111111111111111111000000000000000001111111111111111111111111100000
000000000000011111111111111111000000000000000011111111111111111111

110000000000000000000000000000000000000000000000000000000000000000
000000000000000000000000000000000000000000000000000000000000000000
000000000000000000000000111111111111000000000000000000000000000000
00000000000000000000000000000000000000000000000000000000000
>139 UMUD_ECOLI  P04153 UmuD protein (EC 3.4.21.-) [Contains: UmuD' protein]. - Escherichia coli, - Escherichia coli O157:H7, and - Shigella flexneri.
MLFIKPADLREIVTFPLFSDLVQCGFPSPAADYVEQRIDLNQLLIQHPSATYFVKASGDSMIDGGI
SDGDLLIVDSAITASHGDIVIAAVDGEFTVKKLQLRPTVQLIPMNSAYSPITISSEDTLDVFGVVI
HVVKAMR
000000000000000000000000000000000111111111111100000000000000000000
000000000000000000000000000000000000000000000000000000000000000000
0000000
>176 RNMG_ASPRE  P67876 Ribonuclease mitogillin precursor (EC 3.1.27.-) (Restrictocin). - Aspergillus restrictus.
MVAIKNLFLLAATAVSVLAAPSPLDARATWTCINQQLNPKTNKWEDKRLLYSQAKAESNSHHAPLS
DGKTGSSYPHWFTNGYDGNGKLIKGRTPIKFGKADCDRPPKHSQNGMGKDDHYLLEFPTFPDGHDY
KFDSKKPKEDPGPARVIYTYPNKVFCGIVAHQRGNQGDLRLCSH
000000000000000000000000000001111111111111111111101111111111000000
111111111111111111110000111111111101111111111001111111111111111111
00000000001111111111111110111111111111111111
>128 GLPC_HUMAN  P04921 Glycophorin C (PAS-2') (Glycoprotein beta) (GLPC) (Glycoconnectin) (Sialoglycoprotein D) (Glycophorin D) (GPD). - Homo sapiens (Human).
MWSTRSPNSTAWPLSLEPDPGMASASTTMHTTTIAEPDPGMSGWPDGRMETSTPTIMDIVVIAGVI
AAVAIVLVSLLFVMLRYMYRHKGTYHTNEAKGTEFAESADAALQGDPALQDAGDSSRKEYFI
000000000000111111111111000000000000000000000000000000000000000000
00000000000000000000000000000000000000000111111111100000000000
>1630 MSP1_PLAFK  P04932 Merozoite surface protein 1 precursor (Merozoite surface antigens) (PMMSA) (P190). - Plasmodium falciparum (isolate K1 / Thailand).
MKIIFFLCSFLFFIINTQCVTHESYQELVKKLEALEDAVLTGYSLFHKEKMILNEEEITTKGASAQ
SGTSGTSGTSGPSGPSGTSPSSRSNTLPRSNTSSGASPPADASDSDAKSYADLKHRVRNYLLTIKE
LKYPQLFDLTNHMLTLCDNIHGFKYLIDGYEEINELLYKLNFYFDLLRAKLNDVCANDYCQIPFNL
KIRANELDVLKKLVFGYRKPLDNIKDNVGKMEDYIKKNKKTIENINELIEESKKTIDKNKNATKEE
EKKKLYQAQYDLSIYNKQLEEAHNLISVLEKRIDTLKKNENIKELLDKINEIKNPPPANSGNTPNT
LLDKNKKIEEHEKEIKEIAKTIKFNIDSLFTDPLELEYYLREKNKNIDISAKVETKESTEPNEYPN
GVTYPLSYNDINNALNELNSFGDLINPFDYTKEPSKNIYTDNERKKFINEIKEKIKIEKKKIESDK

KSYEDRSKSLNDITKEYEKLLNEIYDSKFNNNIDLTNFEKMMGKRYSYKVEKLTHHNTFASYENSK
HNLEKLTKALKYMEDYSLRNIVVEKELKYYKNLISKIENEIETLVENIKKDEEQLFEKKITKDENK
PDEKILEVSDIVKVQVQKVLLMNKIDELKKTQLILKNVELKHNIHVPNSYKQENKQEPYYLIVLKK
EIDKLKVFMPKVESLINEEKKNIKTEGQSDNSEPSTEGEITGQATTKPGQQAGSALEGDSVQAQAQ
EQKQAQPPVPVPVPEAKAQVPTPPAPVNNKTENVSKLDYLEKLYEFLNTSYICHKYILVSHSTMNE
KILKQYKITKEEESKLSSCDPLDLLFNIQNNIPVMYSMFDSLNNSLSQLFMEIYEKEMVCNLYKLK
DNDKIKNLLEEAKKVSTSVKTLSSSSMQPLSLTPQDKPEVSANDDTSHSTNLNNSLKLFENILSLG
KNKNIYQELIGQKSSENFYEKILKDSDTFYNESFTNFVKSKADDINSLNDESKRKKLEEDINKLKK
TLQLSFDLYNKYKLKLERLFDKKKTVGKYKMQIKKLTLLKEQLESKLNSLNNPKHVLQNFSVFFNK
KKEAEIAETENTLENTKILLKHYKGLVKYYNGESSPLKTLSEESIQTEDNYASLENFKVLSKLEGK
LKDNLNLEKKKLSYLSSGLHHLIAELKEVIKNKNYTGNSPSENNTDVNNALESYKKFLPEGTDVAT
VVSESGSDTLEQSQPKKPASTHVGAESNTITTSQNVDDEVDDVIIVPIFGESEEDYDDLGQVVTGE
AVTPSVIDNILSKIENEYEVLYLKPLAGVYRSLKKQLENNVMTFNVNVKDILNSRFNKRENFKNVL
ESDLIPYKDLTSSNYVVKDPYKFLNKEKRDKFLSSYNYIKDSIDTDINFANDVLGYYKILSEKYKS
DLDSIKKYINDKQGENEKYLPFLNNIETLYKTVNDKIDLFVIHLEAKVLNYTYEKSNVEVKIKELN
YLKTIQDKLADFKKNNNFVGIADLSTDYNHNNLLTKFLSTGMVFENLAKTVLSNLLDGNLQGMLNI
SQHQCVKKQCPQNSGCFRHLDEREECKCLLNYKQEGDKCVENPNPTCNENNGGCDADAKCTEEDSG
SNGKKITCECTKPDSYPLFDGIFCSSSNFLGISFLLILMLILYSFI
000000000000000000000000000000000000000000000000000000000000000000
000000000000000000000000000000000000000000000000000000000000000000
000000000000000000000000000000000000000000000000000000000000000000
000000000000000000000000000000000000000000000000000000000000000000
000000000000000000000000000000000000000000000000000000000000000000
000000000000000000000000000000000000000000000000000000000000000000
000000000000000000000000000000000000000000000000000000000000000000
000000000000000000000000000000000000000000000000000000000000000000
000000000000000000000000000000000000000000000000000000000000000000
000000000000000000000000000000000000000000000000000000000000000000
000000000000000000000000000000000000000000000000000000000000000000
000000000000000000000000000000000000000000000000000000000000000000
000000000000000000000000000000000000000000000000000000000000000000
000000000000000000000000000000000000000000000000000000000000000000
000000000000000000000000000000000000000000000000000000000000000000
000000000000000000000000000000000000000000000000000000000000000000
000000000000000000000000000000000000000000000000000000000000000000
000000000000000000000000000000000000000000000000000000000000000000
000000000000000000000000000000000000000000000000000000000000000000
000000000000000000000000000000000000000000000000000000000000000000

000000000000000000000000000000000000000000000000000000000000000000
000000000000000000000000000000000000000000000000000000000000000000
000000000000000000000000000000000000000000000000000000000000000000
000000000000011111111111111111000000000000000000000111111111111111
111110111111111111111111111111111111110000000000
>482 K2C8_HUMAN  P05787 Keratin, type II cytoskeletal 8 (Cytokeratin 8) (K8) (CK 8). - Homo sapiens (Human).
SIRVTQKSYKVSTSGPRAFSSRSYTSGPGSRISSSSFSRVGSSNFRGGLGGGYGGASGMGGITAVT
VNQSLLSPLVLEVDPNIQAVRTQEKEQIKTLNNKFASFIDKVRFLEQQNKMLETKWSLLQQQKTAR
SNMDNMFESYINNLRRQLETLGQEKLKLEAELGNMQGLVEDFKNKYEDEINKRTEMENEFVLIKKD
VDEAYMNKVELESRLEGLTDEINFLRQLYEEEIRELQSQISDTSVVLSMDNSRSLDMDSIIAEVKA
QYEDIANRSRAEAESMYQIKYEELQSLAGKHGDDLRRTKTEISEMNRNISRLQAEIEGLKGQRASL
EAAIADAEQRGELAIKDANAKLSELEAALQRAKQDMARQLREYQELMNVKLALDIEIATYRKLLEG
EESRLESGMQNMSIHTKTTGGYAGGLSSAYGGLTSPGLSYSLGSSFGSGAGSSSFSRTSSSRAVVV
KKIETRDGKLVSESSDVLPK
000000000000000000000000000000000000000000000000000000000000000000
000000000000000000000000000000000000000000000000000000000000000000
000000000000000000000000000000000000000000000000000000000000000000
000000000000000000000000000000000000000000000000000000000000000000
000000000000000000000000000000000000000000000000000000000000000000
000000111111111111111111111111000000000000000000000000000000000000
000000000000000000000000000000000000000000000000000000000000000000
00000000000000000000
>497 VL1_BPV2    P06458 Major capsid protein L1. - Bovine papillomavirus type 2.
MALWQQGQKLYLPPTPVSKVLCSETYVQRKSIFYHAETERLLTVGHPYYQVTVGDKTVPKVSANQF
RVFKIQLPDPNQFALPDRTVHNPSKERLVWAVIGVQVSRGQPLGGTVTGHPTFNALLDAENVNRKV
TAQTTDDRKQTGLDAKQQQILLLGCTPAEGEYWTTARPCVTDRLENGACPPLELKNKHIEDGDMME
IGFGAADFKTLNASKSDLPLDIQNEICLYPDYLKMAEDAAGNSMFFFARKEQVYVRHIWTRGGSEK
EAPSKDFYLKNGRGEETLKIPSVHFGSPSGSLVSTDNQIFNRPYWLFRAQGMNNGIAWNNLLFLTV
GDNTRGTNLSISVAADGNALSEYDTGKFNLYHRHMEEYKLAFILELCSVEITAQTLSHLQGLMPSV
LQNWEIGVQPPASSILEDTYRYIESPATKCASNVIPPKEDPYAGLKFWSIDLKEKLSLDLDQFPLG
RRFLAQQGAGCSTVRKRAVATRNSSKPAKRKKIKA
000000000000000000000000000000000000000001111111111111110000000000
000000000000000000000000000000000000000000000000000000000000000000
000000000000000000000000000000000000000000000000000000000000000000
000000000000000000000000000000000000000000000000000000000000000000
000000000000000000000000000000000000000000000000000000000000000000

000000000000000000000000000000000000000000000000000000000000000000
000000000000000000000000000000000000111111111111111000000000000000
00000000000000000000111111111111111
>238 VGLG_HHV11  P06484 Glycoprotein G. - Human herpesvirus 1 (strain 17) (HHV-1) (Human herpes simplex virus - 1).
MSQGAMRAVVPIIPFLLVLVGVSGVPTNVSSTTQPQLQTTGRPSHEAPNMTQTGTTDSPTAISLTT
PDHTPPMPSIGLEEEEEEEGAGDGEHLEGGDGTRDTLPQSPGPAFPLAEDVEKDKPNRPVVPSPDP
NNSPARPETSRPKTPPTIIGPLATRPTTRLTSKGRPLVPTPQHTPLFSFLTASPALDTLFVVSTVI
HTLSFLCIGAMATHLCGGWSRRGRRTHPSVRYVCLPSERG
000000000000000000000000000000000000000000000000000000000000000000
000000000000000111111111111111111100111111111111111111111111111100
000000000000000000000000000111111111111111100000000000000000000000
0000000000000000000000000000000000000000
>394 OM1M_CHLTR  P06597 Major outer membrane protein, serovar L2 precursor (MOMP). - Chlamydia trachomatis.
MKKLLKSVLVFAALSSASSLQALPVGNPAEPSLMIDGILWEGFGGDPCDPCTTWCDAISMRMGYYG
DFVFDRVLQTDVNKEFQMGAKPTTATGNAAAPSTCTARENPAYGRHMQDAEMFTNAAYMALNIWDR
FDVFCTLGATSGYLKGNSASFNLVGLFGDNENHATVSDSKLVPNMSLDQSVVELYTDTTFAWSAGA
RAALWECGCATLGASFQYAQSKPKVEELNVLCNAAEFTINKPKGYVGQEFPLDLKAGTDGVTGTKD
ASIDYHEWQASLALSYRLNMFTPYIGVKWSRASFDADTIRIAQPKSATTVFDVTTLNPTIAGAGDV
KASAEGQLGDTMQIVSLQLNKMKSRKSCGIAVGTTIVDADKYAVTVETRLIDERAAHVNAQFRF
000000000000000000000000000000000000000000000000000000000000000000
000000000000000000000000000000000000000000000000000000000000000000
000000000000000000000000000011111111111111000000000000000000000000
000000000000000000000000000000000000000000000000000000000000000000
000000000000000000000000000000000000000000000111111111111111111111
1111111100000000000000000000000000000000000000000000000000000000
>396 APOA4_HUMAN P06727 Apolipoprotein A-IV precursor (Apo-AIV) (ApoA-IV). - Homo sapiens (Human).
MFLKAVVLTLALVAVAGARAEVSADQVATVMWDYFSQLSNNAKEAVEHLQKSELTQQLNALFQDKL
GEVNTYAGDLQKKLVPFATELHERLAKDSEKLKEEIGKELEELRARLLPHANEVSQKIGDNLRELQ
QRLEPYADQLRTQVNTQAEQLRRQLTPYAQRMERVLRENADSLQASLRPHADELKAKIDQNVEELK
GRLTPYADEFKVKIDQTVEELRRSLAPYAQDTQEKLNHQLEGLTFQMKKNAEELKARISASAEELR
QRLAPLAEDVRGNLKGNTEGLQKSLAELGGHLDQQVEEFRRRVEPYGENFNKALVQQMEQLRQKLG
PHAGDVEGHLSFLEKDLRDKVNSFFSTFKEKESQDKTLSLPELEQQQEQQQEQQQEQVQMLAPLES
000000000000000000000000000000000000000000000000000000000000000000
000000000000000000000000000000000000000000000000000000000000000000
000000000000000000000000000000000000000000000000000000000000000000

000000000000000000000000000000000000000000000000000000000011111111
111111000000000000000000000000000000000000000000000000000000000000
000000000000000000000000000000000000000000000000000000000000000000
>193 RHOA_HUMAN  P61586 Transforming protein RhoA (H12). - Homo sapiens (Human).
MAAIRKKLVIVGDGACGKTCLLIVFSKDQFPEVYVPTVFENYVADIEVDGKQVELALWDTAGQEDY
DRLRPLSYPDTDVILMCFSIDSPDSLENIPEKWTPEVKHFCPNVPIILVGNKKDLRNDEHTRRELA
KMKQEPVKPEEGRDMANRIGAFGYMECSAKTKDGVREVFEMATRAALQARRGKKKSGCLVL
000000000000000000000000000000011111110000000000000000000000000000
000000000000000000000000000000000000000000000000000000000000000000
0000000000000000000000000000000000000000000000000000000000000
>192 RHO2_YEAST  P06781 RHO2 protein. - Saccharomyces cerevisiae (Baker's yeast).
MSEKAVRRKLVIIGDGACGKTSLLYVFTLGKFPEQYHPTVFENYVTDCRVDGIKVSLTLWDTAGQE
EYERLRPFSYSKADIILIGFAVDNFESLINARTKWADEALRYCPDAPIVLVGLKKDLRQEAHFKEN
ATDEMVPIEDAKQVARAIGAKKYMECSALTGEGVDDVFEVATRTSLLMKKEPGANCCIIL
000000000000000000000000000000000111111100000000000000000000000000
000000000000000000000000000000000000000000000000000000000000000000
000000000000000000000000000000000000000000000000000000000000
>568 VL1_HPV18   P06794 Major capsid protein L1. - Human papillomavirus type 18.
MCLYTRVLILHYHLLPLYGPLYHPRPLPLHSILVYMVHIIICGHYIILFLRNVNVFPIFLQMALWR
PSDNTVYLPPPSVARVVNTDDYVTPTSIFYHAGSSRLLTVGNPYFRVPAGGGNKQDIPKVSAYQYR
VFRVQLPDPNKFGLPDTSIYNPETQRLVWACAGVEIGRGQPLGVGLSGHPFYNKLDDTESSHAATS
NVSEDVRDNVSVDYKQTQLCILGCAPAIGEHWAKGTACKSRPLSQGDCPPLELKNTVLEDGDMVDT
GYGAMDFSTLQDTKCEVPLDICQSICKYPDYLQMSADPYGDSMFFCLRREQLFARHFWNRAGTMGD
TVPQSLYIKGTGMPASPGSCVYSPSPSGSIVTSDSQLFNKPYWLHKAQGHNNGVCWHNQLFVTVVD
TTPSTNLTICASTQSPVPGQYDATKFKQYSRHVEEYDLQFIFQLCTITLTADVMSYIHSMNSSILE
DWNFGVPPPPTTSLVDTYRFVQSVAITCQKDAAPAENKDPYDKLKFWNVDLKEKFSLDLDQYPLGR
KFLVQAGLRRKPTIGPRKRSAPSATTSSKPAKRVRVRARK
000000000000000000000000000000000000000000000000000000000000000000
000000000000000000000000000000000000000000000000000000000000000000
000000000000000000000000000000000000000000000000000000000000000000
000000000000000000000000000000000000000000000000000000000000000000
000000000000000000000000000000000000000000000000000000000000000000
000111111000000000000000000000000000000000000000000000000000000000
000000000000000000000000000000000000000000000000000000000000000000
000000000000000000000111111111111111111111000000000000000000000000

0000000000000000000000000000000000000000
>617 HEMA_MEASH  P06830 Hemagglutinin-neuraminidase (EC 3.2.1.18). - Measles virus (strain Halle) (Subacute sclerose panencephalitis - virus).
MSPQRDRINAFYKDNPHPKGSRIVINREHLMIDRPYVLLAVLFVMFLSLIGLLAIAGIRLHRAAIY
TAEIHKSLSTNLDVTNSIEHQVKDVLTPLFKIIGDEVGLRTPQRFTDLVKFISDKIKFLNPDREYD
FRDLTWCINPPERIKLDYDQYCADVAAEELMNALVNSTLLETRTTNQFLAVSKGNCSGPTTIRGQF
SNMSLSLLDLYLGRGYNVSSIVTMTSQGMYGGTYPVEKPNLSSKRSELSQLSMYRVFEVSVIRNPG
LGAPVFHMTNYLEQPVSNDLSNCMVALGELKLAALCHGEDSITIPYQGSGKGVSFQLVKLGVWKSP
TGMQSWVPLSTDDPVIDRLYLSSHRGVIADNQAKWAVPTTRTDDKLRMETCFQQACKGKIQALCEN
PECVPLKDNRIPSYGVLSVDLSLTVELKIKIASGFGPLITHGSGMDLYKSNHNNVYWLTIPPMKNL
ALGVINTLEWIPRFKVSPYLFTVPIKEAGEDCHAPTYLPAEVDGDVKLSSNLVILPGQDLQYVLAT
YDTSRVEHAVVYYVYSPGRSFSYFYPFRLPIKGVPIELQVECFTWDQKLWCRHFCVLADSESGGHI
THSGMVGMGVSCTVTREDGTNRR
000000000000000000000000000000000000000000000000000000000000000000
000000000000000000000000000000000000000000000000000000000000000000
000000000000000000000000000000000000000000000000000000000000000000
000000000000000000000000000000000000000000000000000000000000000000
000000000000000000000000000000000000000000000000000000000000000000
000000000000000000000000000000000000000000000000000000000000000000
000000000000000000000000000000000000000000000000000000000000000000
000000000000000000000000000000000000000000000000000000000000000000
000000000000000000000000000000000011111111111111111111111110000000
00000000000000000000000
>3391 POLG_DEN2J  P07564 Genome polyprotein [Contains: Capsid protein C (Core protein); Envelope protein M (Matrix protein); Major envelope protein E; Nonstructural protein 1 (NS1); Nonstructural protein 2A (NS2A); Flavivirin protease NS2B regulatory subunit; Flavivirin protease NS3 catalytic subunit (EC 3.4.21.91); Nonstructural protein 4A (NS4A); Nonstructural protein 4B (NS4B); RNA-directed RNA polymerase (EC 2.7.7.48) (NS5)]. - Dengue virus type 2 (strain Jamaica).
MNNQRKKARSTPFNMLKRERNRVSTVQQLTKRFSLGMLQGRGPLKLFMALVAFLRFLTIPPTAGIL
KRWGTIKKSKAINVLRGFRKEIGRMLNILNRRRRTAGVIIMLIPTAMAFHLTTRNGEPHMIVGRQE
KGKSLLFKTEDGVNMCTLMAIDLGELCEDTITYKCPLLRQNEPEDIDCWCNSTSTWVTYGTCATTG
EHRREKRSVALVPHVGMGLETRTETWMSSEGAWKHVQRIETWILRHPGFTIMAAILAYTIGTTHFQ
RALIFILLTAVAPSMTMRCIGISNRDFVEGVSGGSWVDIVLEHGSCVTTMAKNKPTLDFELIKTEA
KQPATLRKYCIEAKLTNTTTESRCPTQGEPSLNEEQDKRFLCKHSMVDRGWGNGCGLFGKGGIVTC
AMFTCKKNMEGKVVLPENLEYTIVITPHSGEEHAVGNDTGKHGKEIKITPQSSITEAELTGYGTVT

MECSPRTGLDFNEMVLLQMEDKAWLVHRQWFLDLPLPWLPGADTQGSNWIQKETLVTFKNPHAKKQ
DVVVLGSQEGAMHTALTGATEIQMSSGNLLFTGHLKCRLRMDKLQLKGMSYSMCTGKFKIVKEIAE
TQHGTIVIRVQYEGDGSPCKIPFEIMDLEKRHVLGRLITVNPIVTEKDSPVNIEAEPPFGDSYIII
GVEPGQLKLNWFKKGSSIGQMFETTMRGAKRMAILGDTAWDFGSLGGVFTSIGKALHQVFGAIYGA
AFSGVSWTMKILIGVIITWIGMNSRSTSLSVSLVLVGVVTLYLGAMVQADSGCVVSWKNKELKCGS
GIFITDNVHTWTEQYKFQPESPSKLASAIQKAHEEGICGIRSVTRLENLMWKQITPELNHILSENE
VKLTIMTGDIKGIMQAGKRSLRPQPTELKYSWKTWGKAKMLSTESHNQTFLIDGPETAECPNTNRA
WNSLEVEDYGFGVFTTNIWLKLREKQDVFCDSKLMSAAIKDNRAVHADMGYWIESALNDTWKMEKA
SFIEVKSCHWPKSHTLWSNGVLESEMIIPKNFAGPVSQHNYRPGYHTQTAGPWHLGKLEMDFDFCE
GTTVVVTEDCGNRGPSLRTTTASGKLITEWCCRSCTLPPLRYRGEDGCWYGMEIRPLKEKEENLVN
SLVTAGHGQIDNFSLGVLGMALFLEEMLRTRVGTKHAILLVAVSFVTLITGNMSFRDLGRVMVMVG
ATMTDDIGMGVTYLALLAAFKVRPTFAAGLLLRKLTSKELMMATIGIALLSQSTIPETILELTDAL
ALGMMVLKIVRNMEKYQLAVTIMAILCVPNAVILQNAWKVSCTILAAVSVSPLLLTSSQQKADWIP
LALTIKGLNPTAIFLTTLSRTSKKRSWPLNEAIMAVGMVSILASSLLKNDIPMTGPLVAGGLLTVC
YVLTGRSADLELERAADVKWEDQAEISGSSPILSITISEDGSMSIKNEEEEQTLTILIRTGLLVIS
GVFPVSIPITAAAWYLWEVKKQRAGVLWDVPSPPPVGKAELEDGAYRIKQRGILGYSQIGAGVYKE
GTFHTMWHVTRGAVLMHKGKRIEPSWADVKKDLISYGGGWKLEGEWKEGEEVQVLALEPGKNPRAV
QTKPGLFKTNTGTIGAVSLDFSPGTSGSPIVDRKGKVVGLYGNGVVTRSGAYVSAIAQTEKSIEDN
PEIEDDIFRKKRLTIMDLHPGAGKTKRYLPAIVREAIKRGLRTLILAPTRVVAAEMEEALRGLPIR
YQTPAIRAEHTGREIVDLMCHATFTMRLLSPVRVPNYNLIIMDEAHFTDPASIAARGYISTRVEMG
EAAGIFMTATPPGSRDPFPQSNAPIMDEEREIPERSWNSGHEWVTDFKGKTVWFVPSIKAGNDIAA
CLRKNGKKVIQLSRKTFDSEYVKTRANDWDFVVTTDISEMGANFKAERVIDPRRCMKPVILTDGEE
RVILAGPMPVTHSSAAQRRGRIGRNPKNENDQYIYMGEPLENDEDCAHWKEAKMLLDNINTPEGII
PSMFEPEREKVDAIDGEYRLRGEARKTFVDLMRRGDLPVWLAYRVAAEGINYADRRWCFDGIKNNQ
ILEENVEVEIWTKEGERKKLKPRWLDARIYSDPLALKEFKEFAAGRKSLTLNLITEMGRLPTFMTQ
KARDALDNLAVLHTAEAGGRAYNHALSELPETLETLLLLTLLATVTGGIFLFLMSGKGIGKMTLGM
CCIITASILLWYAQIQPHWIAASIILEFFLIVLLIPEPEKQRTPQDNQLTYVVIAILTVVAATMAN
EMGFLEKTKKDLGLGSITTQESESNILDIDLRPASAWTLYAVATTFVTPMLRHSIENSSVNVSLTA
IANQATVLMGLGKGWPLSKIHIGVPLLAIGCYSQVNPITLTAALLLLVAHYAIIGPGLQAKATREA
QKRAAAGIMKNPTVDGITVIDLDPIPYDPKFEKQLGQVMLLILCVTQVLMMRTTWALCEALTLATG
PISTLWEGNPGRFWNTTIAVSMANIFRGSYLAGAGLLFSIMKNTTNTRRGTGNIGETLGEKWKSRL
NALGKSEFQIYKKSGIQEVDRTLAKEGIKRGETDHHAVSRGSAKLRWFVERNMVTPEGKVVDLGCG
RGGWSYYCGGLKNVREVKGLTKGGPGHEEPIPMSTYGWNLVRLQSGVDVFFTPPEKCDTLLCDIGE
SSPNPTIEAGRTLRVLNLVENWLNNNTQFCIKVLNPYMPSVIEKMETLQRKYGGALVRNPLSRNST
HEMYWVSNASGNIVSSVNMISRMLINRFTMKHKKATYETDVDLGSGTRNIGIESEIPNLDIIGKRI
EKIKQEHETSWHYDQDHPYKTWAYHGSYETKQTGSASSMVNGVVRLLTKPWDVVPMVTQMAMTDTT
PFGQQRVFKEKVDTRTQEPKEGTKKLMKITAEWLWKELGKKKTPRMCTREEFTRKVRSNAALGAIF
TDENKWKSAREAVEDSRFWELVDRERNLHLEGKCETCVYNMMGKREKKLGEFGKAKGSRAIWYMWL

GARFLEFEALGFLNEDHWFSRGNSLSGVEGEGLHKLGYILRDVSKKEGGAMYADDTAGWDTRITLE
DLKNEEMVTNHMEGEHKKLAEAIFKLTYQNKVVRVQRPTPRGTVMDIISRRDQRGSGQVGTYGLNT
FTNMEAQLIRQMEGEGIFKSIQHLTVTEEIAVQNWLARVGRERLSRMAISGDDCVVKPLDDRFASA
LTALNDMGKVRKDIQQWEPSRGWNDWTQVPFCSHHFHELVMKDGRVLVVPCRNQDELIGRARISQG
AGWSLKETACLGKSYAQMWTLMYFHRRDLRLAANAICSAVPSHWVPTSRTTWSIHAKHEWMTTEDM
LAVWNRVWIQENPWMEDKTPVESWEEVPYLGKREDQWCGSLIGLTSRATWAKNIQTAINQVRSLIG
NEEYTDYMPSMKRFRREEEEAGVLW
000000000000000000000000000000000000000000000000000000000000000000
000000000000000000000000000000000000000000000000111111111100000000
000000000000000000000000000000000000000000000000000000000000000000
000000000000000000000000000000000000000000000011111111110000000000
000000000000000000000000000000000000000000000000000000000000000000
000000000000000000000000000000000000000000000000000000000000000000
000000000000000000000000000000000000000000000000000000000000000000
000000000000000000000000000000000000000000000000000000000000000000
000000000000000000000000011111111100000000000000000000000000000000
000000000000000000000000000000000011111111111000000000000000000000
000000000000000000000000000000000000000000000000000000000000000000
000000000000000000000000000000000000000000000000000000000000000000
000000000000000000000000000000000000000000000000000000000000000000
000000000000000000000000000000000000000000000000000000000000000000
000000000000000000000000000000000000000000000000000000000000000000
000000000000000000000000000000000000000000000000000000000000000000
000000000000000000000000000000000000000000000000000000000000000000
000000000000000000000000000000000000000000000000000000000000000000
000000000000000000000000000000000000000000000000000000000000000000
000000000000000000000000000000000000000000000000000000000000000000
000000000000000000000000000000000000000000000000000000000000000000
000000000000000000000000000000000000000000000000000000000000000000
000000000000000000000000000000000000000000000000000000000000000000
000000000000000000000000000000000000000000000000000000000000000000
000000000000000000000000000000000000000000000000000000000000000000
000000000000000000000000000000000000000000000000000000000000000000
000000000000000000000000000000000000000000000000000000000000000000
000000000000000000000000000000000000000000000000000000000000000000
000000000000000000000000000000000000000000000000000000000000000000
000000000000000000000000000000000000000000000000000000000000000000
000000000000000000000000000000000000000000000000000000000000000000

000000000000000000000000000000000000000000000000000000000000000000
000000000000000000000000000000000000000000000000000000000000000000
000000000000000000000000000000000000000000000000000000000000000000
000000000000000000000000000000000000000000000000000000000000000000
000000000000000000000000000000000000000000000000000000000000000000
000000000000000000000000000000000000000000000000000000000000000000
000000000000000000000000000000000000000000000000000000000000000000
000000000000000000000000000000000000000000000000000000000000000000
000000000000000000000000000000000000000000000000000000000000000000
000000000000000000000000000000000000000000000000000000000000000000
000000000000000000000000000000000000000000000000000000000000000000
000000000000000000000000000000000000000000000000000000000000000000
000000000000000000000000000000000000000000000000000000000000000000
000000000000000000000000000000000000000000000000000000000000000000
000000000000000000000000000000000000000000000000000000000000000000
000000000000000000000000000000000000000000000000000000000000000000
000000000000000000000000000000000000000000000000000000000000000000
000000000000000000000000000000000000000000000000000000000000000000
000000000000000000000000000000000000000000000000000000000000000000
000000000000000000000000000000000000000000000000000000000000000000
0000000000000000000000000
>357 VL2_BPV4    P08342 Minor capsid protein L2. - Bovine papillomavirus type 4.
MVRAARRKRASEDDLYRGCRMGQDCPIDIKNKYEHNTLADRILKWVSSFLYFGQLGISSGKGTGGS
TGYTPLGGRGGGGVTSGKGANVVRPTVIVDALGPTGVPIDPAVPDSSIVPLLESSGGSTTLDATPG
AEIEIIAEVHPPPVYEGPEVTIGDIEEPPILEVVPETHPTSRVRSTTSKHDNPAFTAYVASAQLPG
ETSASDNVYILHGFNGDFVGQADPEGDTIFEEIPLEEFGVPDMPPSTSTPTSSFRSVLNKFQRRLY
NRKLVQQVKITNRNTFLKQPSQFVQWEFDNPAYVDDSLSLIFQQDLDEVSAAPDADFQDIVKLSRP
VFTTKEGLVRLSRLGQRGTIKTRMACK
000000000000000000001111111111111111111100000000000000000000000000
000000000000001111111111111111111111111111111111111111000000000011
111111111111111111111111111111111111110000000000000000000000111111
111111111000000000000000000000000000000000000000000000000000000000
000000000000000000000000000000000000000000000000000000000000000000
000000000000000000000000000
>138 PA2A_CRODU  P08878 Crotoxin acid chain precursor (CA) (Crotapotin). - Crotalus durissus terrificus (South American rattlesnake).
MRALWIVAVLLVGVEGSLVEFETLMMKIAGRSGISYYSSYGCYCGAGGQGWPQDASDRCCFEHDCC

YAKLTGCDPTTDVYTYRQEDGEIVCGEDDPCGTQICECDKAAAICFRNSMDTYDYKYLQFSPENCQ
GESQPC
00000000000000000000000000000000000000111111111111111111111000000000
00000000000000000000000000000000000000011111111111111110000000000000
000000
>623 VGLE_VZVD   P09259 Glycoprotein E precursor (Glycoprotein GI). - Varicella-zoster virus (strain Dumas) (VZV).
MGTVNKPVVGVLMGFGIITGTLRITNPVRASVLRYDDFHTDEDKLDTNSVYEPYYHSDHAESSWVN
RGESSRKAYDHNSPYIWPRNDYDGFLENAHEHHGVYNQGRGIDSGERLMQPTQMSAQEDLGDDTGI
HVIPTLNGDDRHKIVNVDQRQYGDVFKGDLNPKPQGQRLIEVSVEENHPFTLRAPIQRIYGVRYTE
TWSFLPSLTCTGDAAPAIQHICLKHTTCFQDVVVDVDCAENTKEDQLAEISYRFQGKKEADQPWIV
VNTSTLFDELELDPPEIEPGVLKVLRTEKQYLGVYIWNMRGSDGTSTYATFLVTWKGDEKTRNPTP
AVTPQPRGAEFHMWNYHSHVFSVGDTFSLAMHLQYKIHEAPFDLLLEWLYVPIDPTCQPMRLYSTC
LYHPNAPQCLSHMNSGCTFTSPHLAQRVASTVYQNCEHADNYTAYCLGISHMEPSFGLILHDGGTT
LKFVDTPESLSGLYVFVVYFNGHVEAVAYTVVSTVDHFVNAIEERGFPPTAGQPPATTKPKEITPV
NPGTSPLLRYAAWTGGLAAVVLLCLVIFLICTAKRMRVKAYRVDKSPYNQSMYYAGLPVDDFEDSE
STDTEEEFGNAIGGSHGGSSYTVYIDKTR
00000000000000000000000000000000000000000000000000000000011111111111
11111111111111111111111111111111111111110000000000111111111111111111
11100000000000000000000000000000000000000000000000000000000000000000
00000000000000000000000000000000000000000000000000000000000000000000
00000000000000000000000000000000000000000000000000000000000000000000
00000000000000000000000000000000000000000000000000000000000000000000
00000000000000000000000000000000000000000000000000000000000000000000
00000000000000000000000000000000000000000000000000000000000000000000
00000000000000000000000000000000000000000000000000000000000000000000
00000000000000000000000000000
>99 CH10_MYCTU  P09621 10 kDa chaperonin (Protein Cpn10) (groES protein) (BCG-A heat shock protein) (10 kDa antigen). - Mycobacterium tuberculosis.
AKVNIKPLEDKILVQANEAETTTASGLVIPDTAKEKPQEGTVVAVGPGRWDEDGEKRIPLDVAEGD
TVIYSKYGGTEIKYNGEEYLILSARDVLAVVSK
00000000000000000000000000000000000000000000000111111111111100000000
000000000000000000000000000000000
>402 OM1E_CHLPS  P10332 Major outer membrane protein precursor (MOMP). - Chlamydia psittaci (Chlamydophila psittaci).
MKKLLKSALLFAATGSALSLQALPVGNPAEPSLLIDGTMWEGASGDPCDPCATWCDAISIRAGYYG
DYVFDRVLKVDVNKTFSGMAATPTQATGNASNTNQPEANGRPNIAYGRHMQDAEWFSNAAFLALNI

WDRFDIFCTLGASNGYFKSSSAAFNLVGLIGFSATSSTSTELPMQLPNVGITQGVVEFYTDTSFSW
SVGARGALWECGCATLGAEFQYAQSNPKIEVLNVTSSPAQFVIHKPRGYKGASSNFPLPITAGTTE
ATDTKSATIKYHEWQVGLALSYRLNMLVPYIGVNWSRATFDADTIRIAQPKLKSEILNITTWNPSL
LGSTTTLPNNGGKDVLSDVLQIASIQINKMKSRKACGVAVGATLIDADKWSITGEARLINERAAHM
NAQFRF
000000000000000000000000000000000000000000000000000000000000000000
000000000000000000000000000000000000000000000000000000000000000000
000000000000000000000000000000000000000000000000000000000000000000
000000000000000000000000000000000000000000000000000000000000000000
000000000000000000000000000000000000000000000000000000000000000000
000000000000000001111111111110000000000000000000000000000000000000
000000
>336 FLA1_BORBU  P11089 Flagellar filament 41 kDa core protein (Flagellin) (P41) (41 kDa antigen). - Borrelia burgdorferi (Lyme disease spirochete).
MIINHNTSAINASRNNGINAANLSKTQEKLSSGYRINRASDDAAGMGVSGKINAQIRGLSQASRNT
SKAINFIQTTEGNLNEVEKVLVRMKELAVQSGNGTYSDADRGSIQIEIEQLTDEINRIADQAQYNQ
MHMLSNKSASQNVRTAEELGMQPAKINTPASLSGSQASWTLRVHVGANQDEAIAVNIYAANVANLF
SGEGAQTAQAAPVQEGVQQEGAQQPAPATAPSQGGVNSPVNVTTTVDANTSLAKIENAIRMISDQR
ANLGAFQNRLESIKDSTEYAIENLKASYAQIKDATMTDEVVAATTNSILTQSAMAMIAQANQVPQY
VLSLLR
000000000000000000000000000000000000000000000000000000000000000000
000000000000000000000000000000000000000000000000000000000000000000
000000000000000000000000000000000000000000000000000000000000000000
000000000000001111111111110000000000000000000000000000000000000000
000000000000000000000000000000000000000000000000000000000000000000
000000
>765 TOP1_HUMAN  P11387 DNA topoisomerase I (EC 5.99.1.2). - Homo sapiens (Human).
MSGDHLHNDSQIEADFRLNDSHKHKDKHKDREHRHKEHKKEKDREKSKHSNSEHKDSEKKHKEKEK
TKHKDGSSEKHKDKHKDRDKEKRKEEKVRASGDAKIKKEKENGFSSPPQIKDEPEDDGYFVPPKED
IKPLKRPRDEDDADYKPKKIKTEDTKKEKKRKLEEEEDGKLKKPKNKDKDKKVPEPDNKKKKPKKE
EEQKWKWWEEERYPEGIKWKFLEHKGPVFAPPYEPLPENVKFYYDGKVMKLSPKAEEVATFFAKML
DHEYTTKEIFRKNFFKDWRKEMTNEEKNIITNLSKCDFTQMSQYFKAQTEARKQMSKEEKLKIKEE
NEKLLKEYGFCIMDNHKERIANFKIEPPGLFRGRGNHPKMGMLKRRIMPEDIIINCSKDAKVPSPP
PGHKWKEVRHDNKVTWLVSWTENIQGSIKYIMLNPSSRIKGEKDWQKYETARRLKKCVDKIRNQYR
EDWKSKEMKVRQRAVALYFIDKLALRAGNEKEEGETADTVGCCSLRVEHINLHPELDGQEYVVEFD
FLGKDSIRYYNKVPVEKRVFKNLQLFMENKQPEDDLFDRLNTGILNKHLQDLMEGLTAKVFRTYNA

SITLQQQLKELTAPDENIPAKILSYNRANRAVAILCNHQRAPPKTFEKSMMNLQTKIDAKKEQLAD
ARRDLKSAKADAKVMKDAKTKKVVESKKKAVQRLEEQLMKLEVQATDREENKQIALGTSKLNYLDP
RITVAWCKKWGVPIEKIYNKTQREKFAWAIDMADEDYEF
0000000000000000000000000000000000000000000000000000000000000000000000
0000000000000000000000000000000000000000000000000000000000000000000000
0000000000000000000000000000000000000000000000000000000000000000000000
0000001111111111111111111100000000000000000000000000000000000000000000
0000000000000000000000000000000000000000000000000000000000000000000000
0000000000000000001111111111111111111100000000000000000000000000000000
1111111111111111111100000000000000000000000000000000000000000000000000
0000000000000000000000000000000000000000000000000000000000111111111111
1111111100000000000000000000000000000000000000000000000000000000000000
0000000000000000000000000000000000000000000000000000000000000000000000
0000000000000000000000000000000000000000000000000000000000000000000000
00000000000000000000000000000000000000000
>932 VGLB_BHV1C  P12640 Glycoprotein I precursor (Glycoprotein GVP-6) (Glycoprotein 11A) (Glycoprotein 16) (Glycoprotein G130) (Glycoprotein B). - Bovine herpesvirus 1.1 (strain Cooper) (BoHV-1) (Infectious bovine - rhinotracheitis virus).
MAARGGAERAAGAGDGRRGQRRHLRPGRVLAALRGPAAPGAGGARAALAAALLWATWALLLAAPAA
GRPATTPPAPPPEEAASPAPPASPSPPGPDGDDAASPDNSTDVRAALRLAQAAGENSRFFVCPPPS
GATVVRLAPARPCPEYGLGRNYTEGIGVIYKENIAPYTFKAYIYKNVIVTTTWAGSTYAAITNQYT
DRVPVGMGEITDLVDKKWRCLSKAEYLRSGRKVVAFDRDDDPWEAPLKPARLSAPGVRGWHTTDDV
YTALGSAGLYRTGTSVNCIVEEVEARSVYPYDSFALSTGDIIYMSPFYGLREGAHREHTSYSPERF
QQIEGYYKRDMATGRRLKEPVSRNFLRTQHVTVAWDWVPKRKNVCSLAKWREADEMLRDESRGNFR
FTARSLSATFVSDSHTFALQNVPLSDCVIEEAEAAVERVYRERYNGTHVLSGSLETYLARGGFVVA
FRPMLSNELAKLYLQELARSNGTLEGLFAAAAPKPGPRRARRAAPSAPGGPGAANGPAGDGDAGGR
VTTVSSAEFAALQFTYDHIQDHVNTMFSRLATSWCLLQNKERALWAEAAKLNPSAAASAALDRRAA
ARMLGDAMAVTYCHELGEGRVFIENSMRAPGGVCYSRPPVSFAFGNESEPVEGQLGEDNELLPGRE
LVEPCTANHKRYFRFGADYVVYENYAYVRRVPLAELEVISTFVDLNLTVLEDREFLPLEVYTRAEL
ADTGLLDYSEIQRRNQLHELRFYDIDRVVKTDGNMAIMRGLANFFQGLGAVGQAVGTVVLGAAGAA
LSTVSGIASFIANPFGALATGLLVLAGLVAAFLAYRYISRLRSNPMKALYPITTRALKDDARGATA
PGEEEEEFDAAKLEQAREMIKYMSLVSAVERQEHKAKKSNKGGPLLATRLTQLALRRRAPPEYQQL
PMADVGGA
0000000000000000000000000000000000000000000000000000000000000000000000
0000000000000000000000000000000000000000000000000000000000000000000000
0000000000000000000000000000000000000000000000000000000000000000000000
0000000000000000000000000000000000000000000000000000000000000000000000

000000000000000000000000000000000000000000000000000000000000000000
111111111111111111111000000000000000000000000000000000000000000000
000000000000000000000000000000000000000000000000000000000000000000
000000000000111111111111111111111111111111111000000000000000000000
000000000000000000000000000000000000000000000000000000000000000000
000000000000000000000000000000000000000000000000000000000000000000
000000000000000000000000000000000000000000000000000000000000000000
000000000000000000000000000000000000000000000000000000000000000000
000000000000000000000000000000000000000000000000000000000000000000
000000000000000000000000000000000000000000000000000000000000000000
00000000
>699 VGLG_HHV2H  P13290 Glycoprotein G. - Human herpesvirus 2 (strain HG52) (HHV-2) (Human herpes simplex virus - 2).
MHAIAPRLLLLFVLSGLPGTRGGSGVPGPINPPNSDVVFPGGSPVAQYCYAYPRLDDPGPLGSADA
GRQDLPRRVVRHEPLGRSFLTGGLVLLAPPVRGFGAPNATYAARVTYYRLTRACRQPILLRQYGGC
RGGEPPSPKTCGSYTYTYQGGGPPTRYALVNASLLVPIWDRAAETFEYQIELGGELHVGLLWVEVG
GEGPGPTAPPQAARAEGGPCVPPVPAGRPWRSVPPVWYSAPNPGFRGLRFRERCLPPQTPAAPSDL
PRVAFAPQSLLVGITGRTFIRMARPTEDVGVLPPHWAPGALDDGPYAPFPPRPRFRRALRTDPEGV
DPDVRAPRTGRRLMALTEDTSSDSPTSAPEKTPLPVSATAMAPSVDPSAEPTAPATTTPPDEMATQ
AATVAVTPEETAVASPPATASVESSPLPAAAAATPGAGHTNTSSASAAKTPPTTPAPTTPPPTSTH
ATPRPTTPGPQTTPPGPATPGPVGASAAPTADSPLTASPPATAPGPSAANVSVAATTATPGTRGTA
RTPPTDPKTHPHGPADAPPGSPAPPPPEHRGGPEEFEGAGDGEPPEDDDSATGLAFRTPNPNKPPP
ARPGPIRPTLPPGILGPLAPNTPRPPAQAPAKDMPSGPTPQHIPLFWFLTASPALDILFIISTTIH
TAAFVCLVALAAQLWRGRAGRRRYAHPSVRYVCLPPERD
000000000000000000000000000000000000000000000000000000000000000000
000000000000000000000000000000000000000000000000000000000000000000
000000000000000000000000000000000000000000000000000000000000000000
001111111111111110000000000000000000000000000000000000000000000000
000000000000000000000000000001111111111111000011111111111111100000
000000000000000000000000000000000000000000000000000000000000000000
000000000000000000000000000000000000000000000000000000000000000000
000000000000000000000000000000000000000000000000000000000000001111
111111111111111111111111111111111111111111101111111111111111111110
001111111111111111111111111100000000000000000000000000000000000000
000000000000000000000000000000000000000
>393 OMPA1_NEIMC P13415 Major outer membrane protein P.IA precursor (Protein IA) (PIA) (Class 1 protein). - Neisseria meningitidis (serogroup C).

MRKKLTALVLSALPLAAVADVSLYGEIKAGVEGRNIQAQLTEQPQVTNGVQGNQVKVTKAKSRIRT
KISDFGSFIGFKGSEDLGEGLKAVWQLEQDVSVAGGGASQWGNRESFIGLAGEFGTLRAGRVANQF
DDASQAINPWDSNNDVASQLGIFKRHDDMPVSVRYDSPEFSGFSGSVQFVPAQNSKSAYKPAYYTK
DTNNNLTLVPAVVGKPGSDVYYAGLNYKNGGFAGNYAFKYARHANVGRNAFELFLIGSATSDEAKG
TDPLKNHQVHRLTGGYEEGGLNLALAAQLDLSENGDKAKTKNSTTEIAATASYRFGNAVPRISYAH
GFDLIERGKKGENTSYDQIIAGVDYDFSKRTSAIVSGAWLKRNTGIGNYTQINAASVGLRHKF
000000000000000000000000000000000000000000000000000000000000000000
000000000000000000000000000000000000000000000000000000000000000000
000000000000000000000000000000000000000000000000000000000000011111
111111110000000000000000000000000000000000000000000000000000000000
000000000000000000000000000000000000000000000000000000000000000000
000000000000000000000000000000000000000000000000000000000000000
>1455 GTFC_STRMU  P13470 Glucosyltransferase-SI precursor (EC 2.4.1.5) (GTF-SI) (Dextransucrase) (Sucrose 6-glucosyltransferase). - Streptococcus mutans.
MEKKVRFKLRKVKKRWVTVSVASAVVTLTSLSGSLVKADSTDDRQQAVTESQASLVTTSEAAKETL
TATDTSTATSATSQPTATVTDNVSTTNQSTNTTANTANFDVKPTTTSEQSKTDNSDKIIATSKAVN
RLTATGKFVPANNNTAHSRTVTDKIVPIKPKIGKLKQPSSLSQDDIAALGNVKNIRKVNGKYYYYK
EDGTLQKNYALNINGKTFFFDETGALSNNTLPSKKGNITNNDNTNSFAQYNQVYSTDAANFEHVDH
YLTAESWYRPKYILKDGKTWTQSTEKDFRPLLMTWWPDQETQRQYVNYMNAQLGIHQTYNTATSPL
QLNLAAQTIQTKIEEKITAEKNTNWLRQTISAFVKTQSAWNSDSEKPFDDHLQKGALLYSNNSKLT
SQANSNYRILNRTPTNQTGKKDPRYTADRTIGGYEFLLANDVDNSNPVVQAEQLNWLHFLMNFGNI
YANDPDANFDSIRVDAVDNVDADLLQIAGDYLKAAKGIHKNDKAANDHLSILEAWSYNDTPYLHDD
GDNMINMDNRLRLSLLYSLAKPLNQRSGMNPLITNSLVNRTDDNAETAAVPSYSFIRAHDSEVQDL
IRNIIRAEINPNVVGYSFTMEEIKKAFEIYNKDLLATEKKYTHYNTALSYALLLTNKSSVPRVYYG
DMFTDDGQYMAHKTINYEAIETLLKARIKYVSGGQAMRNQQVGNSEIITSVRYGKGALKATDTGDR
TTRTSGVAVIEGNNPSLRLKASDRVVVNMGAAHKNQAYRPLLLTTDNGIKAYHSDQEAAGLVRYTN
DRGELIFTAADIKGYANPQVSGYLGVWVPVGAAADQDVRVAASTAPSTDGKSVHQNAALDSRVMFE
GFSNFQAFATKKEEYTNVVIAKNVDKFAEWGVTDFEMAPQYVSSTDGSFLDSVIQNGYAFTDRYDL
GISKPNKYGTADDLVKAIKALHSKGIKVMADWVPDQMYALPEKEVVTATRVDKYGTPVAGSQIKNT
LYVVDGKSSGKDQQAKYGGAFLEELQAKYPELFARKQISTGVPMDPSVKIKQWSAKYFNGTNILGR
GAGYVLKDQATNTYFSLVSDNTFLPKSLVNPNHGTSSSVTGLVFDGKGYVYYSTSGNQAKNAFISL
GNNWYYFDNNGYMVTGAQSINGANYYFLSNGIQLRNAIYDNGNKVLSYYGNDGRRYENGYYLFGQQ
WRYFQNGIMAVGLTRIHGAVQYFDASGFQAKGQFITTADGKLRYFDRDSGNQISNRFVRNSKGEWF
LFDHNGVAVTGTVTFNGQRLYFKPNGVQAKGEFIRDADGHLRYYDPNSGNEVRNRFVRNSKGEWFL
FDHNGIAVTGTRVVNGQRLYFKSNGVQAKGELITERKGRIKYYDPNSGNEVRNRYVRTSSGNWYYF
GNDGYALIGWHVVEGRRVYFDENGVYRYASHDQRNHWDYDYRRDFGRGSSSAVRFRHSRNGFFDNF
FRF

000000000000000000000000000000000000000000000000000000000000000000
000000000000000000000000000000000000000000000000000000000000000000
000000000000000000000000000000000000000000000000000000000000000000
000000000000000000000000000000000000000000000000000000000000000000
000000000000000000000000000000000000000000000000000000000000000000
000000000000000000000000000000000000000000000000000000000000000000
000000000000000000000000000000000000001111111111111111111000000000
000000000000000000000000000000000000000000000000000000000000000000
000000000000000000000000000000000000000000000000000000000000000000
000000000000000000000000000000000000000000000000000000000000000000
000000000000000000000000000000000000000000000000000000000000000000
000000000000000000000000000000000000000000000000000000000000000000
000000000000000000000000000000000000000000000000000000000000000000
000000000000000000000000000000000000000000000000000000000000000000
000000000000000000000000000000000000000000000000000000000000000000
000000000000000000000000000000000000000000000000000000000000000000
000000000000000000000000000000000000000000000000000000000000000000
000000000000000000000000000000000000000000000000000000000000000000
000000000000000000000000000000000000000000000000000000000000000000
000000000000000000000000000000000000000000000000000000000000000000
000000000000000000000000000000000000000000000000000000000000000000
000000000000000000000000000000000000000000000000000000000000000000
000
>350 PORF_PSEAE  P13794 Outer membrane porin F precursor. - Pseudomonas aeruginosa.
MKLKNTLGVVIGSLVAASAMNAFAQGQNSVEIEAFGKRYFTDSVRNMKNADLYGGSIGYFLTDDVE
LALSYGEYHDVRGTYETGNKKVHGNLTSLDAIYHFGTPGVGLRPYVSAGLAHQNITNINSDSQGRQ
QMTMANIGAGLKYYFTENFFAKASLDGQYGLEKRDNGHQGEWMAGLGVGFNFGGSKAAPAPEPVAD
VCSDSDNDGVCDNVDKCPDTPANVTVDANGCPAVAEVVRVQLDVKFDFDKSKVKENSYADIKNLAD
FMKQYPSTSTTVEGHTDSVGTDAYNQKLSERRANAVRDVLVNEYGVEGGRVNAVGYGESRPVADNA
TAEGRAINRRVEAEVEAEAK
000000000000000000000000000000000000000000000000000000000000000000
000000000000000000000000000000000000000000000000000000000000000000
000000000000000000000000000000000000000000000000000000000000000000
000000000000000000000000000000000000000000000000000000000000000000
000000000000000000001111111111111100000000000000000000000000000011
11111111111100000000
>217 OS25_PLAFO  P13829 25 kDa ookinete surface antigen precursor

(Pfs25). - Plasmodium falciparum (isolate NF54).
MNKLYSLFLFLFIQLSIKYNNAKVTVDTVCKRGFLIQMSGHLECKCENDLVLVNEETCEEKVLKCD
EKTVNKPCGDFSKCIKIDGNPVSYACKCNLGYDMVNNVCIPNECKNVTCGNGKCILDTSNPVKTGV
CSCNIGKVPNVQDQNKCSKDGETKCSLKCLKENETCKAVDGIYKCDCKDGFIIDNESSICTAFSAY
NILNLSIMFILFSVCFFIM
000000000000000000000000000000000000000000000000000000000000
000000000000000000000000000000000000000000001111111111111111
100000000000000000000000000000000000000000000000000000000000
0000000000000000000
>272 RSR1_YEAST  P13856 Ras-related protein RSR1. - Saccharomyces cerevisiae (Baker's yeast).
MRDYKLVVLGAGGVGKSCLTVQFVQGVYLDTYDPTIEDSYRKTIEIDNKVFDLEILDTAGIAQFTA
MRELYIKSGMGFLLVYSVTDRQSLEELMELREQVLRIKDSDRVPMVLIGNKADLINERVISVEEGI
EVSSKWGRVPFYETSALLRSNVDEVFVDLVRQIIRNEMESVAVKDARNQSQQFSKIESPSTRLPSS
AKQDTKQSNNKQSSKGLYNKSSQGQAKVKQSTPVNEKHKPSHAVPKSGSSNRTGISATSQQKKKKK
NASTCTIL
000000000000000000000000001111110000000000000000000000000000
000000000000000000000000000000000000000000000000000000000000
000000000000000000000000000000000000000000000000000000000000
000000000000000000000000000000000000000000000000000000000000
00000000
>910 PERT_BORPE  P14283 Pertactin precursor (P.93) [Contains: Outer membrane protein P.69]. - Bordetella pertussis.
MNMSLSRIVKAAPLRRTTLAMALGALGAAPAAHADWNNQSIVKTGERQHGIHIQGSDPGGVRTASG
TTIKVSGRQAQGILLENPAAELQFRNGSVTSSGQLSDDGIRRFLGTVTVKAGKLVADHATLANVGD
TWDDDGIALYVAGEQAQASIADSTLQGAGGVQIERGANVTVQRSAIVDGGLHIGALQSLQPEDLPP
SRVVLRDTNVTAVPASGAPAAVSVLGASELTLDGGHITGGRAAGVAAMQGAVVHLQRATIRRGDAP
AGGAVPGGAVPGGAVPGGFGPGGFGPVLDGWYGVDVSGSSVELAQSIVEAPELGAAIRVGRGARVT
VSGGSLSAPHGNVIETGGARRFAPQAAPLSITLQAGAHAQGKALLYRVLPEPVKLTLTGGADAQGD
IVATELPSIPGTSIGPLDVALASQARWTGATRAVDSLSIDNATWVMTDNSNVGALRLASDGSVDFQ
QPAEAGRFKVLTVNTLAGSGLFRMNVFADLGLSDKLVVMQDASGQHRLWVRNSGSEPASANTLLLV
QTPLGSAATFTLANKDGKVDIGTYRYRLAANGNGQWSLVGAKAPPAPKPAPQPGPQPPQPPQPQPE
APAPQPPAGRELSAAANAAVNTGGVGLASTLWYAESNALSKRLGELRLNPDAGGAWGRGFAQRQQL
DNRAGRRFDQKVAGFELGADHAVAVAGGRWHLGGLAGYTRGDRGFTGDGGGHTDSVHVGGYATYIA
DSGFYLDATLRASRLENDFKVAGSDGYAVKGKYRTHGVGASLEAGRRFTHADGWFLEPQAELAVFR
AGGGAYRAANGLRVRDEGGSSVLGRLGLEVGKRIELAGGRQVQPYIKASVLQEFDGAGTVHTNGIA
HRTELRGTRAELGLGMAAALGRGHSLYASYEYSKGPKLAMPWTFHAGYRYSW
000000000000000000000000000000000000000000000000000000000000

000000000000000000000000000000000000000000000000000000000000000000
000000000000000000000000000000000000000000000000000000000000000000
000000000000000000000000000000000000000000000000000000000111111111
111111111111111111111111111111111111000000000000000000000000000000
000000000000000000000000000000000000000000000000000000000000000000
000000000000000000000000000000000000000000000000000000000000000000
000000000000000000000000000000000000000000000000000000000000000000
000000000000000000000000000000000000000000000000000000000000000000
000000000000000000000000000000000000000000000000000000000000000000
000000000000000000000000000000000000000000000000000000000000000000
000000000000000000000000000000000000000000000000000000000000000000
000000000000000000000000000000000000000000000000000000000000000000
0000000000000000000000000000000000000000000000000000
>569 URE2_HELPY  P69996 Urease beta subunit (EC 3.5.1.5) (Urea amidohydrolase). - Helicobacter pylori (Campylobacter pylori).
MKKISRKEYVSMYGPTTGDKVRLGDTDLIAEVEHDYTIYGEELKFGGGKTLREGMSQSNNPSKEEL
DLIITNALIVDYTGIYKADIGIKDGKIAGIGKGGNKDMQDGVKNNLSVGPATEALAGEGLIVTAGG
IDTHIHFISPQQIPTAFASGVTTMIGGGTGPADGTNATTITPGRRNLKWMLRAAEEYSMNLGFLAK
GNASNDASLADQIEAGAIGFKIHEDWGTTPSAINHALDVADKYDVQVAIHTDTLNEAGCVEDTMAA
IAGRTMHTFHTEGAGGGHAPDIIKVAGEHNILPASTNPTIPFTVNTEAEHMDMLMVCHHLDKSIKE
DVQFADSRIRPQTIAAEDTLHDMGIFSITSSDSQAMGRVGEVITRTWQTADKNKKEFGRLKEEKGD
NDNFRIKRYLSKYTINPAIAHGISEYVGSVEVGKVADLVLWSPAFFGVKPNMIIKGGFIALSQMGD
ANASIPTPQPVYYREMFAHHGKAKYDANITFVSQAAYDKGIKEELGLERQVLPVKNCRNITKKDMQ
FNDTTAHIEVNPETYHVFVDGKEVTSKPANKVSLAQLFSIF
000000000000000000000000000000000000000000000000000000000000000000
000000000000000000000000000000000000000000000000000000000000000000
000000000000000000000000000000000000000000000000000000000000000000
000000000000000000000000000000000000000000000000000000000000000000
000000000000000000000000000000000000000000000000000000001111111111
111111111000000000000000000000000000000000000000000000000000000000
000000000000000000000000000000000000000000000000000000000000000000
000000000000000000000000000000000000000000000000000000000000000000
00000000000000000000000000000000000000000
>137 REF_HEVBR   P15252 Rubber elongation factor protein (REF) (Allergen Hev b 1). - Hevea brasiliensis (Para rubber tree).
AEDEDNQQGQGEGLKYLGFVQDAATYAVTTFSNVYLFAKDKSGPLQPGVDIIEGPVKNVAVPLYNR
FSYIPNGALKFVDSTVVASVTIIDRSLPPIVKDASIQVVSAIRAAPEAARSLASSLPGQTKILAKV
FYGEN

111111111100001111111100000000000011111111111111111111000001111111
111111100000000000000011111111111111111110000000000000000000000000
00000
>205 RHOQ_HUMAN  P17081 Rho-related GTP-binding protein RhoQ (Ras-related GTP-binding protein TC10). - Homo sapiens (Human).
MAHGPGALMLKCVVVGDGAVGKTCLLMSYANDAFPEEYVPTVFDHYAVSVTVGGKQYLLGLYDTAG
QEDYDRLRPLSYPMTDVFLICFSVVNPASFQNVKEEWVPELKEYAPNVPFLLIGTQIDLRDDPKTL
ARLNDMKEKPICVEQGQKLAKEIGACCYVECSALTQKGLKTVFDEAIIAILTPKKHTVKKRIGSRC
INCCLIT
000000000000000000000000000000000001111111000000000000000000000000
000000000000000000000000000000000000000000000000000000000000000000
000000000000000000000000000000000000000000000000000000000000000000
0000000
>204 RRAS2_MOUSE P62071 Ras-related protein R-Ras2. - Mus musculus (Mouse).
MAAAGWRDGSGQEKYRLVVVGGGGVGKSALTIQFIQSYFVTDYDPTIEDSYTKQCVIDDRAARLDI
LDTAGQEEFGAMREQYMRTGEGFLLVFSVTDRGSFEEIYKFQRQILRVKDRDEFPMILIGNKADLD
HQRQVTQEEGQQLARQLKVTYMEASAKIRMNVDQAFHELVRVIRKFQEQECPPSPEPTRKEKDKKG
CHCVIF
000000000000000000000000000000000000000011111110000000000000000000
000000000000000000000000000000000000000000000000000000000000000000
000000000000000000000000000000000000000000000000000000000000000000
000000
>400 VMSA_HPBV9  P17101 Major surface antigen precursor. - Hepatitis B virus (subtype adw / strain 991).
MGGWSSKPRKGMGTNLSVPNPLGFFPDHQLDPVFGANSNNPDWDFNPIKDHWPAANQVGVGAFGPG
FTPPHGGVLGWSPQAQGMLTPVSTIPPPASANRQSGRQPTPISPPLRDSHPQAMQWNSTAFHQALQ
DPRVRGLYFPAGGSSSGTVNPAPNIASHISSISARTGDPVTNMENITSGFLGPLPVLQAGFFLLTR
ILTIPQSLDSWWTSLNFLGGSPVCLGQNSRSPTSNHSPTSCPPICPGYRWMCLRRFIIFLFILLLC
LIFLLVLLDYQGMLPVCPLILGSTTTSTGPCKTCTTPAQGNSMFPSCCCTKPTDGNCTCIPIPSSW
AFAKYLWEWASVRFSWLSLLVPFVQWFVGLSPTVWLSAIWMMWYWGPSLYSIVSSFIPLLPIFFCL
WVYI
000000000000000000000000000000000000000000000000000000000000000000
000000000000000000000000000000000000000000000000000000000000000000
000000000000000000000000000000000000000000000000000000000000000000
000000000000000000000000000000000000000000000000000000000000000000
000000000000000000000000000000000000000000000000000000000000000000
000000000000000000111111111111111000000000000000000000000000000000

0000
>504 VL1_HPV31   P17388 Major capsid protein L1. - Human papillomavirus type 31.
MSLWRPSEATVYLPPVPVSKVVSTDEYVTRTNIYYHAGSARLLTVGHPYYSIPKSDNPKKIVVPKV
SGLQYRVFRVRLPDPNKFGFPDTSFYNPETQRLVWACVGLEVGRGQPLGVGISGHPLLNKFDDTEN
SNRYAGGPGTDNRECISMDYKQTQLCLLGCKPPIGEHWGKGSPCSNNAITPGDCPPLELKNSVIQD
GDMVDTGFGAMDFTALQDTKSNVPLDICNSICKYPDYLKMVAEPYGDTLFFYLRREQMFVRHFFNR
SGTVGESVPTDLYIKGSGSTATLANSTYFPTPSGSMVTSDAQIFNKPYWMQRAQGHNNGICWGNQL
FVTVVDTTRSTNMSVCAAIANSDTTFKSSNFKEYLRHGEEFDLQFIFQLCKITLSADIMTYIHSMN
PAILEDWNFGLTTPPSGSLEDTYRFVTSQAITCQKTAPQKPKEDPFKDYVFWEVNLKEKFSADLDQ
FPLGRKFLLQAGYRARPKFKAGKRSAPSASTTTPAKRKKTKK
000000000000000000000000000000000000000000000000000000000000000000
000000000000000000000000000000000000000000000000000000000000000000
000000000000000000000000000000000000000000000000000000000000000000
000000000000000000000000000000000000000000000000000000000000000000
000000000000000000000000000000000000000000000000000000000000000000
000000000000000000000000000000000000000000000000000000000000000000
000000000000000000000000001111111111111111111100000000000000000000
000000000000000000000000000000000000000000
>393 OM1E_CHLTR  P17451 Major outer membrane protein, serovar E precursor (MOMP). - Chlamydia trachomatis.
MKKLLKSVLVFAALSSASSLQALPVGNPAEPSLMIDGILWEGFGGDPCDPCTTWCDAISMRMGYYG
DFVFDRVLKTDVNKEFQMGDKPTSTTGNATAPTTLTARENPAYGRHMQDAEMFTNAACMALNIWDR
FDVFCTLGASSGYLKGNSASFNLVGLFGDNENQSTVKTNSVPNMSLDQSVVELYTDTAFSWSVGAR
AALWECGCATLGASFQYAQSKPKVEELNVLCNAAEFTINKPKGYVGQEFPLALIAGTDAATGTKDA
SIDYHEWQASLALSYRLNMFTPYIGVKWSRASFDADTIRIAQPKSATAIFDTTTLNPTIAGAGDVK
ASAEGQLGDTMQIVSLQLNKMKSRKSCGIAVGTTIVDADKYAVTVETRLIDERAAHVNAQFRF
000000000000000000000000000000000000000000000000000000000000000000
000000000000000000000000000000000000000000000000000000000000000000
000000000000000000000000000000000000000000000000000000000000000000
000000000000000000000000000000000000000000000000000000000000000000
000000000000000000000000000000000000000000001111111001111111000000
000000000000000000000000000000000000000000000000000000000000000
>890 ADHE_ECOLI  P17547 Aldehyde-alcohol dehydrogenase [Includes: Alcohol dehydrogenase (EC 1.1.1.1) (ADH); Acetaldehyde dehydrogenase [acetylating] (EC 1.2.1.10) (ACDH); Pyruvate-formate-lyase deactivase (PFL deactivase)]. - Escherichia coli, and - Escherichia coli O157:H7.
AVTNVAELNALVERVKKAQREYASFTQEQVDKIFRAAALAAADARIPLAKMAVAESGMGIVEDKVI

KNHFASEYIYNAYKDEKTCGVLSEDDTFGTITIAEPIGIICGIVPTTNPTSTAIFKSLISLKTRNA
IIFSPHPRAKDATNKAADIVLQAAIAAGAPKDLIGWIDQPSVELSNALMHHPDINLILATGGPGMV
KAAYSSGKPAIGVGAGNTPVVIDETADIKRAVASVLMSKTFDNGVICASEQSVVVVDSVYDAVRER
FATHGGYLLQGKELKAVQDVILKNGALNAAIVGQPAYKIAELAGFSVPENTKILIGEVTVVDESEP
FAHEKLSPTLAMYRAKDFEDAVEKAEKLVAMGGIGHTSCLYTDQDNQPARVSYFGQKMKTARILIN
TPASQGGIGDLYNFKLAPSLTLGCGSWGGNSISENVGPKHLINKKTVAKRAENMLWHKLPKSIYFR
RGSLPIALDEVITDGHKRALIVTDRFLFNNGYADQITSVLKAAGVETEVFFEVEADPTLSIVRKGA
ELANSFKPDVIIALGGGSPMDAAKIMWVMYEHPETHFEELALRFMDIRKRIYKFPKMGVKAKMIAV
TTTSGTGSEVTPFAVVTDDATGQKYPLADYALTPDMAIVDANLVMDMPKSLCAFGGLDAVTHAMEA
YVSVLASEFSDGQALQALKLLKEYLPASYHEGSKNPVARERVHSAATIAGIAFANAFLGVCHSMAH
KLGSQFHIPHGLANALLICNVIRYNANDNPTKQTAFSQYDRPQARRRYAEIADHLGLSAPGDRTAA
KIEKLLAWLETLKAELGIPKSIREAGVQEADFLANVDKLSEDAFDDQCTGANPRYPLISELKQILL
DTYYGRDYVEGETAAKKEAAPAKAEKKAKKSA
000000000000000000000000000000000000000000000000000000000000000000
000000000000000000000000000000000000000000000000000000000000000000
000000000000000000000000000000000000000000000000000000000000000000
000000000000000000000000000000000000000000000000000000000000000000
000000000000000000000000000000000000000000000000000000000000000000
000000000000000000000000000000000000000000000000000000000000000000
000000000000000000000000000000000000000000000000000000000000000000
000000000000000000000000000000000000000000000000000000000000000000
000000000000000000000000000000000000000000000000000000000000000000
000000000000000000000000000000000000000000000000000000000000000000
000000000000000000000000000000000000000000000000000000000000000000
000000000000000000000000000000000000000000000000000000000000000000
000000000000000000000000000000000000000000000000000000111111111111
11000000000000000000000000000000
>659 DNAK_CHLTR  P17821 Chaperone protein dnaK (Heat shock protein 70) (Heat shock 70 kDa protein) (HSP70) (75 kDa membrane protein). - Chlamydia trachomatis.
SEKRKSNKIIGIDLGTTNSCVSVMEGGQPKVIASSEGTRTTPSIVAFKGGETLVGIPAKRQAVTNP
EKTLASTKRFIGRKFSEVESEIKTVPYKVAPNSKGDAVFDVEQKLYTPEEIGAQILMKMKETAEAY
LGETVTEAVITVPAYFNDSQRASTKDAGRIAGLDVKRIIPEPTAAALAYGIDKEGDKKIAVFDLGG
GTFDISILEIGDGVFEVLSTNGDTHLGGDDFDGVIINWMLDEFKKQEGIDLSKDNMALQRLKDAAE
KAKIELSGVSSTEINQPFITIDANGPKHLALTLTRAQFEHLASSLIERTKQPCAQALKDAKLSASD
IDDVLLVGGMSRMPAVQAVVKEIFGKEPNKGVNPDEVVAIGAAIQGGVLGGEVKDVLLLDVIPLSL
GIETLGGVMTPLVERNTTIPTQKKQIFSTAADNQPAVTIVVLQGERPMAKDNKEIGRFDLTDIPPA
PRGHPQIEVTFDIDANGILHVSAKDAASGREQKIRIEASSGLKEDEIQQMIRDAELHKEEDKQRKE

ASDVKNEADGMIFRAEKAVKDYHDKIPAELVKEIEEHIEKVRQAIKEDASTTAIKAASDELSTHMQ
KIGEAMQAQSASAAASSAANAQGGPNINSEDLKKHSFSTRPPAGGSASSTDNIEDADVEIVDKPE
000000000000000000000000000000000000000000000000000000000000000000
000000000000000000000000000000000000000000000000000000000000000000
000000000000000000000000000000000000000000000000000000000000000000
000000000000000000000000000000000000000000000000000000000000000000
000000000000000000000000000000000000000000000000000000000000000000
000000000000000000000000000011111111111110000000000000000000000000
000000000000000000000000000000000000000000000000000000000000000000
000000000000000000000000000000000000000000000000000000000000000000
000000000000000000000000000000000000000000000000000000000000000000
00000000000000000000000000000000000000000000000000000000000000000
>183 RAP2B_RAT P61227 Ras-related protein Rap-2b. - Rattus norvegicus (Rat).
MREYKVVVLGSGGVGKSALTVQFVTGSFIEKYDPTIEDFYRKEIEVDSSPSVLEILDTAGTEQFAS
MRDLYIKNGQGFILVYSLVNQQSFQDIKPMRDQIIRVKRYERVPMILVGNKVDLEGEREVSYGEGK
ALAEEWSCPFMETSAKNKASVDELFAEIVRQMNYAAQPNGDEGCCSACVIL
000000000000000000000000000001111110000000000000000000000000000000
000000000000000000000000000000000000000000000000000000000000000000
000000000000000000000000000000000000000000000000000
>209 TNNI3_HUMAN P19429 Troponin I, cardiac muscle (Cardiac troponin I). - Homo sapiens (Human).
ADGSSDAAREPRPAPAPIRRRSSNYRAYATEPHAKKKSKISASRKLQLKTLLLQIAKQELEREAEE
RRGEKGRALSTRCQPLELAGLGFAELQDLCRQLHARVDKVDEERYDIEAKVTKNITEIADLTQKIF
DLRGKFKRPTLRRVRISADAMMQALLGARAKESLDLRAHLKQVKKEDTEKENREVGDWRKNIDALS
GMEGRKKKFES
000000000001111111111111111111111111111111000000000000000000000000
000000000000000001111111111111111000000000011111111110000000000000
000000000000001111111111111111111000000000000000000001111111111111
00000000000
>393 OM1L_CHLTR P19542 Major outer membrane protein, serovar L1 precursor (MOMP). - Chlamydia trachomatis.
MKKLLKSVLVFAALSSASSLQALPVGNPAEPSLMIDGILWEGFGGDPCDPCTTWCDAISMRMGYYG
DFVFDRVLQTDVNKEFQMGAKPTATTGNAAAPSTCTARENPAYGRHMQDAEMFTNAAYMALNIWDR
FDVFCTLGATSGYLKGNSASFNLVGLFGDNENQSTVKKDAVPNMSFDQSVVELYTDTTFAWSVGAR
AALWECGCATLGASFQYAQSKPKVEELNVLCNAAEFTINKPKGYVGKEFPLDLTAGTDAATGTKDA
SIDYHEWQASLALSYRLNMFTPYIGVKWSRASFDADTIRIAQPKLATAIFDTTTLNPTIAGAGEVK
ANAEGQLGDTMQIVSLQLNKMKSRKSCGIAVGTTIVDADKYAVTETRLIDERAAHVNAQFRF

000000000000000000000000000000000000000000000000000000000000000000
000000000000000000000000000000000000000000000000000000000000000000
000000000000000000000000000000000000111111110000000000000000000000
000000000000000000000000000000000000000000000000000000000000000000
000000000000000000000000000000000000000000000011111111111110000000
000000000011111111000000000000000000000000000000000000000000000
>338 G3P_SCHMA   P20287 Glyceraldehyde-3-phosphate dehydrogenase (EC 1.2.1.12) (GAPDH) (Major larval surface antigen) (P-37). - Schistosoma mansoni (Blood fluke).
MSRAKVGINGFGRIGRLVLRAAFLKNTVDVVSVNDPFIDLEYMVYMIKRDSTHGTFPGEVSTENGK
LKVNGKLISVHCERDPANIPWDKDGAEYVVESTGVFTTIDKAQAHIKNNRAKKVIISAPSADAPMF
VVGVNENSYEKSMSVVSNASCTTNCLAPLAKVIHDKFEIVEGLMTTVHSFTATQKVVDGPSSKLWR
DGRGAMQNIIPASTGAAKAVGKVIPALNGKLTGMAFRVPTPDVSVVDLTCRLGKGASYEEIKAAVK
AAASGPLKGILEYTEDEVVSSDFVGSTSSSIFDAKAGISLNNNFVKLVSWYDNEFGYSCRVVDLIT
HMHKVDHA
000000000000000000000000000000111111111111100000000000001111111111
111111111111111100000000000000000111111111111111000000000000000000
000000000000000000000000000000111111111100000000000000000000000000
000000000000000000000000000000000000000000000000000000000000000000
000111111111111111111111100000000000000000000000000000000000000000
00000000
>360 PGS2_BOVIN  P21793 Decorin precursor (Bone proteoglycan II) (PG-S2). - Bos taurus (Bovine).
MKATIIFLLVAQVSWAGPFQQKGLFDFMLEDEASGIGPEEHFPEVPEIEPMGPVCPFRCQCHLRVV
QCSDLGLEKVPKDLPPDTALLDLQNNKITEIKDGDFKNLKNLHTLILINNKISKISPGAFAPLVKL
ERLYLSKNQLKELPEKMPKTLQELRVHENEITKVRKSVFNGLNQMIVVELGTNPLKSSGIENGAFQ
GMKKLSYIRIADTNITTIPQGLPPSLTELHLDGNKITKVDAASLKGLNNLAKLGLSFNSISAVDNG
SLANTPHLRELHLNNNKLAKVPGGVADHKYIQVVYLHNNNISAIGSNDFCPPGYNTKKASYSGVSL
FSNPVQYWEIQPSTFRCVYVRAAVQLGNYK
000000000000000000000000000000001111111111100000000000000000000000
000000000000000000000000000000000000000000000000000000000000000000
000000000000000001111111111000000000000111111111111000000000000000
000000000000000000000000000000000000000000000000000000000000000000
001111111111111100000000000000000000000000000000000000000000000000
000000000000000000000000000000
>397 OM1N_CHLTR  P23114 Major outer membrane protein, serovar L3 precursor (MOMP). - Chlamydia trachomatis.
MKKLLKSVLVFAALSSASSLQALPVGNPAEPSLMIDGILWEGFGGDPCDPCTTWCDAISMRVGYYG

DFVFDRVLKTDVNKEFQMGAEPTTSDTAGLSNDPTTNVARPNPAYGKHMQDAEMFTNAAYMALNIW
DRFDVFCTLGATTGYLKGNSASFNLVGLFGTKTQSTNFNTAKLVPNTALNQAVVELYTDTTFAWSV
GARAALWECGCATLGASFQYAQSKPKVEELNVLCDASEFTINKPKGYVGAEFPLDITAGTEAATGT
KDASIDYHEWQASLALSYRLNMFTPYIGVKWSRVSFDADTIRIAQPKLAEAVLDVTTLNPTIAGKG
SVVASGSENELADTMQIVSLQLNKMKSRKSCGIAVGTTIVDADKYAVTVETRLIDERAAHVNAQFR
F
000000000000000000000000000000000000000000000000000000000000000000
000000000000000000000000000000000000000000000000000000000000000000
000000000000000000000000000000000000000000000000000000000000000000
000000000000000000000000000000000000000000000000011111110000000000
000000000000000000000000000000000000000000000000000000011111111000
000000000000000000000000000000000000000000000000000000000000000000
0
>394 OM1B_CHLTR  P23421 Major outer membrane protein, serovar B
precursor (MOMP). - Chlamydia trachomatis.
MKKLLKSVLVFAALSSASSLQALPVGNPAEPSLMIDGILWEGFGGDPCDPCTTWVDAISMRMGYYG
DFVFDRVLKTDVNKEFQMGAKPTTTTGNAVAPSTLTARENPAYGRHMQDAEMFTNAACMALNIWDR
FDVFCTLGASSGYLKGNSASFNLVGLFGNNENQTKVSNGAFVPNMSLDQSVVELYTDTAFAWSVGA
RAALWECGCATLGASFQYAQSKPKVEELNVLCNAAEFTINKPKGYVGKELPLDLTAGTDAATGTKD
ASIDYHEWQASLALSYRLNMFTPYIGVKWSRASFDADTIRIAQPKSAETIFDVTTLNPTIAGAGDV
KTSAEGQLGDTMQIVSLQLNKMKSRKSCGIAVGTTIVDADKYAVTVETRLIDERAAHVNAQFRF
000000000000000000000000000000000000000000000000000000000000000000
000000000000000000000000111111111111111100000000000000000000000000
000001111111111110000000111111111111111111111000000000000000000000
000000000000000000000000000000000000011111111111100000000000000000
000000000000000000000000000000001111111111110111111111111111111111
0000000000000000000000000000000000000000000000000000000000000000
>396 OM1A_CHLTR  P23732 Major outer membrane protein, serovar A
precursor (MOMP). - Chlamydia trachomatis.
MKKLLKSVLVFAALSSASSLQALPVGNPAEPSLMIDGILWEGFGGDPCDPCTTWCDAISMRMGYYG
DFVFDRVLKTDVNKEFQMGAAPTTRDVAGLEKDPVVNVARPNPAYGKHMQDAEMFTNAAYMALNIW
DRFDVFCTLGATTGYLKGNSASFNLVGLFGTKTQSSGFDTANIVPNTALNQAVVELYTDTTFAWSV
GARAALWECGCATLGASFQYAQSKPKVEELNVLCNASEFTINKPKGYVGAEFPLDITAGTEAATGT
KDASIDYHEWQASLALSYRLNMFTPYIGVKWSRVSFDADTIRIAQPKLAKPVLDTTTLNPTIAGKG
TVVSSAENELADTMQIVSLQLNKMKSRKSCGIAVGTTIVDADKYAVTVETRLIDERAAHVNAQFRF
000000000000000000000000000000000000000000000000000000000000000000
000000000000000000111111111111111111111000000000000000000000000000
000000000000000000000000000000000000000000000000000000000000000000

000000000000000000000000000000000000000000000000000000000000000000
000000000000000000000000000000000000000000000001111111111111111111
111111111100000000000000000000000000000000000000000000000000000000
>389 VMSA_HPBVA  P24025 Major surface antigen precursor. - Hepatitis B virus (strain alpha1).
MGQNLSTSNPLGFFPDHQLDPAFRANTANPDWDFNPNKDSWPDANKVGAGAFGLGFTPPHGGLLGW
SPQAQGILQTLPANPPPASTNRQSGRQPTPLSPPLRNTHPQAMQWNSTTFHQTLQDPRVRGLYLPA
GGSSSGTVNPVPTTVSPISSIFSRIGDPALNMENITSGFLGPLLVLQAGFFLLTKILTIPKSLDSW
WTSLNFLGGTTVCLGQNSQSPTSNHSPTSCPPTCPGYRWMCLRRFIIFLFILLLCLIFLLVLLDYQ
GMLPVCPLIPGSSTTSTGPCRTCTTPAQGTSMYPSCCCTKPSDGNCTCIPIPSSWAFGKFLWEWAS
ARFSWLSLLVPFVQWFVGLSPTVWLLVIWMMWYWGPKLFTILSPFLPLLPIFFCLWVYI
000000000000000000000000000000000000000000000000000000000000000000
000000000000000000000000000000000000000000111111111111000000000000
000000000000000000000000000000000000000000000000000000000000000000
000000000000000000000000000000000000000000000000000000000000000000
000000000000000000000000000000000000000000000000000000000000000000
00000000000000000000000000000000000000000000000000000000000
>510 VL1_HPV2A   P25486 Major capsid protein L1. - Human papillomavirus type 2a.
MSCGLNDVNVSTISLQMALWRPNESKVYLPPTPVSKVISTDVYVTRTNVYYHGGSSRLLTVGHPYY
SIKKSNNKVAVPKVSGYQYRVFHVKLPDPNKFGLPDADLYDPDTQRLLWACVGVEVGRGQPLGVGV
SGHPYYNRLDDTENAHTPDTADDGRENISMDYKQTQLFILGCKPPIGEHWSKGTTCNGSSAAGDCP
PLQFTNTTIEDGDMVETGFGALDFATLQSNKSDVPLDICTNTCKYPDYLKMAAEPYGDSMFFSLRR
EQMFTRHFFNLGGKMGDTIPDELYIKSTSVPTPGSHVYTSTPSGSMVSSEQQLFNKPYWLRRAQGH
NNGMCWGNRVFLTVVDTTRSTNVSLCATEASDTNYKATNFKEYLRHMEEYDLQFIFQLCKITLTPE
IMAYIHNMDPQLLEDWNFGVPPPPSASLQDTYRYLQSQAITCQKPTPPKTPTDPYASLTFWDVDLS
ESFSMDLDQFPLGRKFLLQRGAMPTVSRKRAAVSGTTPPTSKRKRVRR
000000000000000000000000000000000000000000000000000000000000000000
000000000000000000000000000000000000000000000000000000000000000000
000000000000000000000000000000000000000000000000000000000000000000
000000000000000000000000000000000000000000000000000000000000000000
000000000000000000001111110000000000000000000000000000000000000000
000000000000000000000000000000000000000000000000000000000000000000
000000000000000000000000000000000000000000000000000000000000000000
000000000000000000000000000000000000000000000000
>3010 POLG_HCVBK  P26663 Genome polyprotein [Contains: Capsid protein C (Core protein) (p21); Envelope glycoprotein E1 (gp32) (gp35); Envelope glycoprotein E2 (gp68) (gp70) (NS1); p7; Protease NS2 (EC

3.4.22.-) (p23) (NS2-3 proteinase); Protease/helicase NS3 (EC 3.4.21.98) (p70) (Hepacivirin); Nonstructural protein 4A (NS4A) (p8); Nonstructural protein 4B (NS4B) (p27); Nonstructural protein 5A (NS5A) (Membrane-associated serine phosphoprotein) (p56) (p58); RNA-directed RNA polymerase (EC 2.7.7.48) (p68) (NS5B)]. - Hepatitis C virus (isolate BK) (HCV).

MSTNPKPQRKTKRNTNRRPQDVKFPGGGQIVGGVYLLPRRGPRLGVRAPRKTSERSQPRGRRQPIP
KARRPEGRTWAQPGYPWPLYGNEGLGWAGWLLSPRGSRPSWGPTDPRRRSRNLGKVIDTLTCGFAD
LMGYIPLVGAPLGGAARALAHGVRVLEDGVNYATGNLPGCSFSIFLLALLSCLTTPASAYEVHNVS
GIYHVTNDCSNASIVYEAADLIMHTPGCVPCVREGNSSRCWVALTPTLAARNVTIPTTTIRRHVDL
LVGAAAFCSAMYVGDLCGSVFLVSQLFTFSPRRHVTLQDCNCSIYPGHVSGHRMAWDMMMNWSPTT
ALVVSQLLRIPQAVVDMVAGAHWGVLAGLAYYSMAGNWAKVLIVMLLFAGVDGDTHVTGGAQAKTT
NRLVSMFASGPSQKIQLINTNGSWHINRTALNCNDSLQTGFLAALFYTHSFNSSGCPERMAQCRTI
DKFDQGWGPITYAESSRSDQRPYCWHYPPPQCTIVPASEVCGPVYCFTPSPVVVGTTDRFGVPTYR
WGENETDVLLLNNTRPPQGNWFGCTWMNSTGFTKTCGGPPCNIGGVGNNTLTCPTDCFRKHPEATY
TKCGSGPWLTPRCMVDYPYRLWHYPCTVNFTIFKVRMYVGGVEHRLNAACNWTRGERCDLEDRDRP
ELSPLLLSTTEWQVLPCSFTTLPALSTGLIHLHQNIVDVQYLYGIGSAVVSFAIKWEYVLLLFLLL
ADARVCACLWMMLLIAQAEAALENLVVLNSASVAGAHGILSFLVFFCAAWYIKGRLVPGATYALYG
VWPLLLLLLALPPRAYAMDREMAASCGGAVFVGLVLLTLSPYYKVFLARLIWWLQYFTTRAEADLH
VWIPPLNARGGRDAIILLMCAVHPELIFDITKLLIAILGPLMVLQAGITRVPYFVRAQGLIHACML
VRKVAGGHYVQMAFMKLGALTGTYIYNHLTPLRDWPRAGLRDLAVAVEPVVFSDMETKIITWGADT
AACGDIILGLPVSARRGKEILLGPADSLEGRGLRLLAPITAYSQQTRGLLGCIITSLTGRDKNQVE
GEVQVVSTATQSFLATCVNGVCWTVYHGAGSKTLAAPKGPITQMYTNVDQDLVGWPKPPGARSLTP
CTCGSSDLYLVTRHADVIPVRRRGDSRGSLLSPRPVSYLKGSSGGPLLCPFGHAVGIFRAAVCTRG
VAKAVDFVPVESMETTMRSPVFTDNSSPPAVPQSFQVAHLHAPTGSGKSTKVPAAYAAQGYKVLVL
NPSVAATLGFGAYMSKAHGIDPNIRTGVRTITTGAPVTYSTYGKFLADGGCSGGAYDIIICDECHS
TDSTTILGIGTVLDQAETAGARLVVLATATPPGSVTVPHPNIEEVALSNTGEIPFYGKAIPIEAIR
GGRHLIFCHSKKKCDELAAKLSGLGINAVAYYRGLDVSVIPTIGDVVVVATDALMTGYTGDFDSVI
DCNTCVTQTVDFSLDPTFTIETTTVPQDAVSRSQRRGRTGRGRRGIYRFVTPGERPSGMFDSSVLC
ECYDAGCAWYELTPAETSVRLRAYLNTPGLPVCQDHLEFWESVFTGLTHIDAHFLSQTKQAGDNFP
YLVAYQATVCARAQAPPPSWDQMWKCLIRLKPTLHGPTPLLYRLGAVQNEVTLTHPITKYIMACMS
ADLEVVTSTWVLVGGVLAALAAYCLTTGSVVIVGRIILSGRPAIVPDRELLYQEFDEMEECASHLP
YIEQGMQLAEQFKQKALGLLQTATKQAEAAAPVVESKWRALETFWAKHMWNFISGIQYLAGLSTLP
GNPAIASLMAFTASITSPLTTQSTLLFNILGGWVAAQLAPPSAASAFVGAGIAGAAVGSIGLGKVL
VDILAGYGAGVAGALVAFKVMSGEMPSTEDLVNLLPAILSPGALVVGVVCAAILRRHVGPGEGAVQ
WMNRLIAFASRGNHVSPTHYVPESDAAARVTQILSSLTITQLLKRLHQWINEDCSTPCSGSWLRDV
WDWICTVLTDFKTWLQSKLLPQLPGVPFFSCQRGYKGVWRGDGIMQTTCPCGAQITGHVKNGSMRI
VGPKTCSNTWHGTFPINAYTTGPCTPSPAPNYSRALWRVAAEEYVEVTRVGDFHYVTGMTTDNVKC

PCQVPAPEFFSEVDGVRLHRYAPACRPLLREEVTFQVGLNQYLVGSQLPCEPEPDVAVLTSMLTDP
SHITAETAKRRLARGSPPSLASSSASQLSAPSLKATCTTHHVSPDADLIEANLLWRQEMGGNITRV
ESENKVVVLDSFDPLRAEEDEREVSVPAEILRKSKKFPAAMPIWARPDYNPPLLESWKDPDYVPPV
VHGCPLPPIKAPPIPPPRRKRTVVLTESSVSSALAELATKTFGSSESSAVDSGTATALPDQASDDG
DKGSDVESYSSMPPLEGEPGDPDLSDGSWSTVSEEASEDVVCCSMSYTWTGALITPCAAEESKLPI
NALSNSLLRHHNMVYATTSRSAGLRQKKVTFDRLQVLDDHYRDVLKEMKAKASTVKAKLLSVEEAC
KLTPPHSAKSKFGYGAKDVRNLSSKAVNHIHSVWKDLLEDTVTPIDTTIMAKNEVFCVQPEKGGRK
PARLIVFPDLGVRVCEKMALYDVVSTLPQVVMGSSYGFQYSPGQRVEFLVNTWKSKKNPMGFSYDT
RCFDSTVTENDIRVEESIYQCCDLAPEARQAIKSLTERLYIGGPLTNSKGQNCGYRRCRASGVLTT
SCGNTLTCYLKASAACRAAKLQDCTMLVNGDDLVVICESAGTQEDAASLRVFTEAMTRYSAPPGDP
PQPEYDLELITSCSSNVSVAHDASGKRVYYLTRDPTTPLARAAWETARHTPVNSWLGNIIMYAPTL
WARMILMTHFFSILLAQEQLEKALDCQIYGACYSIEPLDLPQIIERLHGLSAFSLHSYSPGEINRV
ASCLRKLGVPPLRVWRHRARSVRARLLSQGGRAATCGKYLFNWAVKTKLKLTPIPAASRLDLSGWF
VAGYSGGDIYHSLSRARPRWFMLCLLLLSVGVGIYLLPNR
000000000000000000000000000000000000000000000000000000000000000000
000000000000000000000000000000000000000000000000000000000000000000
000000000000000000000000000000000000000000000000000000000000000000
000000000000000000000000000000000000000000000000000000000000000000
000000000000000000000000000000000000000000000000000000000000000000
000000000000000000000000000000000000000000000000000000000000000000
000000000000000000000000000000000000000000000000000000000000000000
000000000000000000000000000000000000000000000000000000000000000000
111111111111111111000000000000000000000000000000000000000000000000
000000000000000000000000000000000000000000000000000000000000000000
000000000000000000000000000000000000000000000000000000000000000000
000000000000000000000000000000000000000000000000000000000000000000
000000000000000000000000000000000000000000000000000000000000000000
000000000000000000000000000000000000000000000000000000000000000000
000000000000000000000000000000000000000000000000000000000000000000
000000000000000000000000000000000000000000000000000000000000000000
000000000000000000000000000000000000000000000000000000000000000000
000000000000000000000000000000000000000000000000000000000000000000
000000000000000000000000000000000000000000000000000000000000000000
000000000000000000000000000000000000000000000000000000000000000000
000000000000000000000000000000000000000000000000000000000000000000
000000000000000000000000000000000000000000000000000000000000000000
000000000000000000000000000000000000000000000000000000000000000000
000000000000000000000000000000000000000000000000000000000000000000

000000000000000000000000000000000000000000000000000000000000000000000000
000000000000000000000000000000000000000000000000000000000000000000000000
000000000000000000000000000000000000000000000000000000000000000000000000
000000000000000000000000000000000000000000000000000000000000000000000000
000000000000000000000000000000000000000000000000000000000000000000000000
000000000000000000000000000000000000000000000000000000000000000000000000
000000000000000000000000000000000000000000000000000000000000000000000000
000000000000000000000000000000000000000000000000000000000000000000000000
000000000000000000000000000000000000000000000000000000000000000000000000
000000000000000000000000000000000000000000000000000000000000000000000000
000000000000000000000000000000000000000000000000000000000000000000000000
000000000000000000000000000000000000000000000000000000000000000000000000
000000000000000000000000000000000000000000000000000000000000000000000000
000000000000000000000000000000000000000000000000000000000000000000000000
000000000000000000000000000000000000000000000000000000000000000000000000
000000000000000000000000000000000000000000000000000000000000000000000000
000000000000000000000000000000000000000000000000000000000000000000000000
000000000000000000000000000000000000000000000000000000000000000000000000
000000000000000000000000000000000000000000000000000000000000000000000000
000000000000000000000000000000000000000000000000000000000000000000000000
000000000000000000000000000000000000000000000000000000000000000000000000
0000000000000000000000000000000000000000
>3011 POLG_HCV1   P26664 Genome polyprotein [Contains: Capsid protein C (Core protein) (p21); Envelope glycoprotein E1 (gp32) (gp35); Envelope glycoprotein E2 (gp68) (gp70) (NS1); p7; Protease NS2 (EC 3.4.22.-) (p23) (NS2-3 proteinase); Protease/helicase NS3 (EC 3.4.21.98) (p70) (Hepacivirin); Nonstructural protein 4A (NS4A) (p8); Nonstructural protein 4B (NS4B) (p27); Nonstructural protein 5A (NS5A) (Membrane-associated serine phosphoprotein) (p56) (p58); RNA-directed RNA polymerase (EC 2.7.7.48) (p68) (NS5B)]. - Hepatitis C virus (isolate 1) (HCV).
MSTNPKPQKKNKRNTNRRPQDVKFPGGGQIVGGVYLLPRRGPRLGVRATRKTSERSQPRGRRQPIP
KARRPEGRTWAQPGYPWPLYGNEGCGWAGWLLSPRGSRPSWGPTDPRRRSRNLGKVIDTLTCGFAD
LMGYIPLVGAPLGGAARALAHGVRVLEDGVNYATGNLPGCSFSIFLLALLSCLTVPASAYQVRNST
GLYHVTNDCPNSSIVYEAADAILHTPGCVPCVREGNASRCWVAMTPTVATRDGKLPATQLRRHIDL
LVGSATLCSALYVGDLCGSVFLVGQLFTFSPRRHWTTQGCNCSIYPGHITGHRMAWDMMMNWSPTT
ALVMAQLLRIPQAILDMIAGAHWGVLAGIAYFSMVGNWAKVLVVLLLFAGVDAETHVTGGSAGHTV
SGFVSLLAPGAKQNVQLINTNGSWHLNSTALNCNDSLNTGWLAGLFYHHKFNSSGCPERLASCRPL

TDFDQGWGPISYANGSGPDQRPYCWHYPPKPCGIVPAKSVCGPVYCFTPSPVVVGTTDRSGAPTYS
WGENDTDVFVLNNTRPPLGNWFGCTWMNSTGFTKVCGAPPCVIGGAGNNTLHCPTDCFRKHPDATY
SRCGSGPWITPRCLVDYPYRLWHYPCTINYTIFKIRMYVGGVEHRLEAACNWTRGERCDLEDRDRS
ELSPLLLTTTQWQVLPCSFTTLPALSTGLIHLHQNIVDVQYLYGVGSSIASWAIKWEYVVLLFLLL
ADARVCSCLWMMLLISQAEAALENLVILNAASLAGTHGLVSFLVFFCFAWYLKGKWVPGAVYTFYG
MWPLLLLLLALPQRAYALDTEVAASCGGVVLVGLMALTLSPYYKRYISWCLWWLQYFLTRVEAQLH
VWIPPLNVRGGRDAVILLMCAVHPTLVFDITKLLLAVFGPLWILQASLLKVPYFVRVQGLLRFCAL
ARKMIGGHYVQMVIIKLGALTGTYVYNHLTPLRDWAHNGLRDLAVAVEPVVFSQMETKLITWGADT
AACGDIINGLPVSARRGREILLGPADGMVSKGWRLLAPITAYAQQTRGLLGCIITSLTGRDKNQVE
GEVQIVSTAAQTFLATCINGVCWTVYHGAGTRTIASPKGPVIQMYTNVDQDLVGWPAPQGSRSLTP
CTCGSSDLYLVTRHADVIPVRRRGDSRGSLLSPRPISYLKGSSGGPLLCPAGHAVGIFRAAVCTRG
VAKAVDFIPVENLETTMRSPVFTDNSSPPVVPQSFQVAHLHAPTGSGKSTKVPAAYAAQGYKVLVL
NPSVAATLGFGAYMSKAHGIDPNIRTGVRTITTGSPITYSTYGKFLADGGCSGGAYDIIICDECHS
TDATSILGIGTVLDQAETAGARLVVLATATPPGSVTVPHPNIEEVALSTTGEIPFYGKAIPLEVIK
GGRHLIFCHSKKKCDELAAKLVALGINAVAYYRGLDVSVIPTSGDVVVVATDALMTGYTGDFDSVI
DCNTCVTQTVDFSLDPTFTIETITLPQDAVSRTQRRGRTGRGKPGIYRFVAPGERPSGMFDSSVLC
ECYDAGCAWYELTPAETTVRLRAYMNTPGLPVCQDHLEFWEGVFTGLTHIDAHFLSQTKQSGENLP
YLVAYQATVCARAQAPPPSWDQMWKCLIRLKPTLHGPTPLLYRLGAVQNEITLTHPVTKYIMTCMS
ADLEVVTSTWVLVGGVLAALAAYCLSTGCVVIVGRVVLSGKPAIIPDREVLYREFDEMEECSQHLP
YIEQGMMLAEQFKQKALGLLQTASRQAEVIAPAVQTNWQKLETFWAKHMWNFISGIQYLAGLSTLP
GNPAIASLMAFTAAVTSPLTTSQTLLFNILGGWVAAQLAAPGAATAFVGAGLAGAAIGSVGLGKVL
IDILAGYGAGVAGALVAFKIMSGEVPSTEDLVNLLPAILSPGALVVGVVCAAILRRHVGPGEGAVQ
WMNRLIAFASRGNHVSPTHYVPESDAAARVTAILSSLTVTQLLRRLHQWISSECTTPCSGSWLRDI
WDWICEVLSDFKTWLKAKLMPQLPGIPFVSCQRGYKGVWRVDGIMHTRCHCGAEITGHVKNGTMRI
VGPRTCRNMWSGTFPINAYTTGPCTPLPAPNYTFALWRVSAEEYVEIRQVGDFHYVTGMTTDNLKC
PCQVPSPEFFTELDGVRLHRFAPPCKPLLREEVSFRVGLHEYPVGSQLPCEPEPDVAVLTSMLTDP
SHITAEAAGRRLARGSPPSVASSSASQLSAPSLKATCTANHDSPDAELIEANLLWRQEMGGNITRV
ESENKVVILDSFDPLVAEEDEREISVPAEILRKSRRFAQALPVWARPDYNPPLVETWKKPDYEPPV
VHGCPLPPPKSPPVPPPRKKRTVVLTESTLSTALAELATRSFGSSSTSGITGDNTTTSSEPAPSGC
PPDSDAESYSSMPPLEGEPGDPDLSDGSWSTVSSEANAEDVVCCSMSYSWTGALVTPCAAEEQKLP
INALSNSLLRHHNLVYSTTSRSACQRQKKVTFDRLQVLDSHYQDVLKEVKAAASKVKANLLSVEEA
CSLTPPHSAKSKFGYGAKDVRCHARKAVTHINSVWKDLLEDNVTPIDTTIMAKNEVFCVQPEKGGR
KPARLIVFPDLGVRVCEKMALYDVVTKLPLAVMGSSYGFQYSPGQRVEFLVQAWKSKKTPMGFSYD
TRCFDSTVTESDIRTEEAIYQCCDLDPQARVAIKSLTERLYVGGPLTNSRGENCGYRRCRASGVLT
TSCGNTLTCYIKARAACRAAGLQDCTMLVCGDDLVVICESAGVQEDAASLRAFTEAMTRYSAPPGD
PPQPEYDLELITSCSSNVSVAHDGAGKRVYYLTRDPTTPLARAAWETARHTPVNSWLGNIIMFAPT
LWARMILMTHFFSVLIARDQLEQALDCEIYGACYSIEPLDLPPIIQRLHGLSAFSLHSYSPGEINR
VAACLRKLGVPPLRAWRHRARSVRARLLARGGRAAICGKYLFNWAVRTKLKLTPIAAAGQLDLSGW

FTAGYSGGDIYHSVSHARPRWIWFCLLLLAAGVGIYLLPNR
000000000000000000000000000000000000000000000000000000000000000000
000000000000000000000000000000000000000000000000000000000000000000
000000000000000000000000000000000000000000000000000000000000000000
000000000000000000000000000000000000000000000000000000000000000000
000000000000000000000000000000000000000000000000001111111111000000
000000000000000000000000000000000000000000000000000000000000000000
000000000000000000000000000000000000000000000000000000000000000000
000000000000000000000000000000000000000000000000000000000000000000
000000000000000000000000000000000000000000000000000000000000000000
000000000000000000000000000000000000000000000000000000000000000000
000000000000000000000011111111100000000000000000000000000000000000
000000000000000000000000000000000000000000000000000000000000000000
000000000000000000000000000000000000000000000000000000000000000000
000000000000000000000000000000000000000000000000000000000000000000
000000000000000000000000000000000000000000000000000000000000000000
000000000000000000000000000000000000000000000000000000000000000000
000000000000000000000000000000000000000000000000000000000000000000
000000000000000000000000000000000000000000000000000000000000000000
000000000000000000000000000000000000000000000000000000000000000000
000000000000000000000000000000000000000000000000000000000000000000
000000000000000000000000000000000000000000000000000000000000000000
000000000000000000000000000000000000000000000000000000000000000000
000000000000000000000000000000000000000000000000000000000000000000
000000000000000000000000000000000000000000000000000000000000000000
000000000000000000000000000000000000000000000000000000000000000000
000000000000000000000000000000000000000000000000000000000000000000
000000000000000000000000000000000000000000000000000000000000000000
000000000000000000000000000000000000000000000000000000000000000000
000000000000000000000000000000000000000000000000000000000000000000
000000000000000000000000000000000000000000000000000000000000000000
000000000000000000000000000000000000000000000000000000000000000000
000000000000000000000000000000000000000000000000000000000000000000
000000000000000000000000000000000000000000000000000000000000000000
000000000000000000000000000000000000000000000000000000000000000000
000000000000000000000000000000000000000000000000000000000000000000
000000000000000000000000000000000000000000000000000000000000000000
000000000000000000000000000000000000000000000000000000000000000000

00000000000000000000000000000000000000000000000000000000000000000
00000000000000000000000000000000000000000000000000000000000000000
00000000000000000000000000000000000000000000000000000000000000000
00000000000000000000000000000000000000000000000000000000000000000
00000000000000000000000000000000000000000000000000000000000000000
00000000000000000000000000000000000000000000000000000000000000000
00000000000000000000000000000000000000000000000000000000000000000
00000000000000000000000000000000000000000000000000000000000000000
0000000000000000000000000000000000000000
>170 CAF1_YERPE  P26948 F1 capsule antigen precursor. - Yersinia pestis.
MKKISSVIAIALFGTIATANAADLTASTTATATLVEPARITLTYKEGAPITIMDNGNIDTELLVGT
LTLGGYKTGTTSTSVNFTDAAGDPMYLTFTSQDGNNHQFTTKVIGKDSRDFDISPKVNGENLVGDD
VVLATGSQDFFVRSIGSKGGKLAAGKYTDAVTVTVSNQ
00000000000000000000000000000000000000000000000000000000000000000
00000000000000000000000000000000000000000000000000000000000000000
00000000011111111111111111111111100000
>433 NCAP_PUUMS  P27313 Nucleocapsid protein (Nucleoprotein). - Puumala virus (strain Sotkamo/V-2969/81).
MSDLTDIQEDITRHEQQLIVARQKLKDAERAVEVDPDDVNKNTLQARQQTVSALEDKLADYKRRMA
DAVSRKKMDTKPTDPTGIEPDDHLKERSSLRYGNVLDVNAIDIEEPSGQTADWYTIGVYVIGFTLP
IILKALYMLSTRGRQTVKENKGTRIRFKDDTSFEDINGIRRPKHLYVSMPTAQSTMKAEELTPGRF
RTIVCGLFPTQIQVRNIMSPVMGVIGFSFFVKDWSERIREFMEKECPFIKPEVKPGTPAQEIEMLK
RNKIYFMQRQDVLDKNHVADIDKLIDYAASGDPTSPDNIDSPNAPWVFACAPDRCPPTCIYVAGMA
ELGAFFSILQDMRNTIMASKTVGTAEEKLKKKSSFYQSYLRRTQSMGIQLDQRIILLFMLEWGKEM
VDHFHLGDDMDPELRGLAQALIDQKVKEISNQEPLKI
00000000011111111111111111111110000000000000000000111111111111111
11111000000011111111111111011111111111110000000000000000000111111
11111111000000011111111111111000000000000000000000000000000000000
00000000000000000000000000000000000000000000000111111111111111110
11111111111111111111000000000000000000000000000000000000000000000
00000000000000000000000000000000000000000000000000000000000000000
0000000000001111111111111110000000000
>668 COAT_FCVC6  P27404 Capsid protein precursor (Coat protein). - Feline calicivirus (strain CFI/68 FIV) (FCV).
MCSTCANVLKYYDWDPHIKLVINPNKFLHVGFCDNPLMCCYPELLPEFGTMWDCDQSPLQVYLESI
LGDDEWSSTHEAIDPVVPPMHWDEAGKIFQPHPGVLMHHLICKVAEGWDPNLPLFRLEADDGSITT
PEQGTMVGGVIAEPNAQMSTAADMATGKSVDSEWEAFFSFHTSVNWSTSETQGKILFKQSLGPLLN

PYLTHLAKLYVAWSGSVDVRFSISGSGVFGGKLAAIVVPPGIDPVQSTSMLQYPHVLFDARQVEPV
IFSIPDLRSTLYHLMSDTDTTSLVIMVYNDLINPYANDSNSSGCIVTVETKPGPDFKFHLLKPPGS
MLTHGSIPSDLIPKSSSLWIGNRFWSDITDFVIRPFVFQANRHFDFNQETAGWSTPRFRPITITIS
VKESAKLGIGVATDYIVPGIPDGWPDTTIPGELVPVGDYAITNGTNNDITTAAQYDAATEIRNNTN
FRGMYICGSLQRAWGDKKISNTAFITTGTVDGAKLIPSNTIDQTKIAVFQDTHANKHVQTSDDTLA
LLGYTGIGEEAIGADRDRVVRISVLPERGARGGNHPIFHKNSIKLGYVIRSIDVFNSQILHTSRQL
SLNHYLLSPDSFAVYRIIDSNGSWFDIGIDNDGFSFVGVSSIGKLEFPLTASYMGIQLAKIRLASN
IRSVMTKL
000000000000000000000000000000000000000000000000000000000000000000
000000000000000000000000000000000000000000000000000000000000000000
000000000000000000000000000000000000000000000000000000000000000000
000000000000000000000000000000000000000000000000000000000000000000
000000000000000000000000000000000000000000000000000000000000000000
000000000000000000000000000000000000000000000000000000000000000000
000000000000000011111111111000000000000000000001111111111111111000
000000001111111111111000000000000000000000000000000000000000000000
000000000000000000000000000000000000000000000000000000000000000000
000000000000000000000000000000000000000000000000000000000000000000
00000000
>620 HEMA_MEASY  P28081 Hemagglutinin-neuraminidase (EC 3.2.1.18). - Measles virus (strain Yamagata-1) (Subacute sclerose panencephalitis - virus).
MSPQRDRTNAFYKDNPHPKGSRIVINREHLMIDRPYVLLAILFVMFLSLIGLLAIAGIRLHQAAIH
TAEIHKSLSTNLDVTNSIEHQVKDVLTPLFKIIGDEVGLRTPQRFTDLVKFISDKIKFLNPDREYD
FRDLNWCINPPERIKLDYDQYCADVAAEELMNALVNSTLLETRTTNQFLAVSKGNCSGPTTIRGQF
SNMSTSLLDLYLSRGYNVSSIVTMTSQGMYGGTYLVEKPNLSSKRSELSQLSMYRVFEVGVIRNPG
LGAPVFHMTNYFEQPVSNDLSNCMVALGEFKLAALCHREDSITIPYQGSGKGVSFQLVNLGVWKSP
TDMQSWIPLSTDDPVIDRLYLSSHRGVIADNQAKWAVPTTRTDDKLRMETCFQQACKGKIQALCEN
PEWAPLKDNRIPSYGVLSVDLSPTVELKIKIASGFGPLITHGSGMDLYKSNHNNVYWLTIPPMKNL
ALGVINTLEWIPRFKVSPNLFTVPIKEAGKDCHAPTYLPAEVDGDVKLSSNLVILPGQDLQYVLAT
YDTSRVEHAVVYYVYSPGRSFSYFYPFRLPIRGVPIELQVECFTWDQKLWCRHFCVLANSESGGHI
THSGMVGMGVSCTVTREDGTNRRQSC
000000000000000000000000000000000000000000000000000000000000000000
000000000000000000000000000000000000000000000000000000000000000000
000000000000000000000000000000000000000000000000000000000000000000
000000000000000000000000000000000000000000000000000000000000000000
000000000000000000000000000000000000000000000000000000000000000000
000000000000000000000000000000000000000000000000011111111111111111

111100000000000000000000000000000000000000000000000000000000000000
000000000000000000000000000000000000000000000000000000000000000000
000000000000000000000000000000000000000000000000000000000000000000
00000000000000000000000000
>1459 CO2A1_MOUSE P28481 Collagen alpha 1(II) chain precursor [Contains: Chondrocalcin]. - Mus musculus (Mouse).
MIRLGAPQSLVLLTLLIAAVLRCQGQDAQEAGSCLQNGQRYKDKDVWKPSSCRICVCDTGNVLCDD
IICEDPDCLNPEIPFGECCPICPADLATASGRKLGPKGQKGEPGDIRDGAPGPRGRDGEPGTPGNP
GPAGPPGPPGPPGLSAGNFAAQMAGGYDEKAGGAQMGVMQGPMGPMGPRGPPGPAGAPGPQGFQGN
PGEPGEPGVSGPMGPRGPPGPAGKPGDDGEAGKPGKSGERGLPGPMGARGFPGTPGLPGVKGHRGY
PGLDGAKGEAGAPGVKGESGSPGENGSPGPMGPRGLPGERGRTGPAGAAGARGNDGQPGPAGPPGP
VGPAGGPGFPGAPGAKGEAGPTGARGPEGAQGSRGEPGNPGSPGPAGASGNPGTDGIPGAKGSAGA
PGIAGAPGFPGPRGPPGPQGATGPLGPKGQAGEPGIAGFKGDQGPKGETGPAGPQGAPGPAGEEGK
RGARGEPGGAGPIGPPGERGAPGNRGFPGQDGLAGPKGAPGERGPSGLAGPKGANGDPGRPGEPGL
PGARGLTGRPGDAGPQGKVGPSGAPGEDGRPGPPGPQGARGQPGVMGFPGPKGANGEPGKAGEKGL
AGAPGLRGLPGKDGETGAAGPPGPSGPAGERGEQGAPGPSGFQGLPGPPGPPGEGGKQGDQGIPGE
AGAPGLVGPRGERGFPGERGSPGAQGLQGPRGLPGTPGTDGPKGAAGPDGPPGAQGPPGLQGMPGE
RGAAGIAGPKGDRGDVGEKGPEGAPGKDGGRGLTGPIGPPGPAGANGEKGEAGPPGPSGSTGARGA
PGEPGETGPPGPAGFAGPPGADGQPGAKGDQGEAGQKGDAGAPGPQGPSGAPGPQGPTGVTGPKGA
RGAQGPPGATGFPGAAGRVGPPGANGNPGPAGPPGPAGKDGPKGVRGDSGPPGRAGDPGLEGPAGA
PGEKGEPGDDGPSGLDGPPGPQGLAGQRGIVGLPGQRGERGFPGLPGPSGEPGKQGAPGASGDRGP
PGPVGPPGLTGPAGEPGREGSPGADGPPGRDGAAGVKGDRGETGALGAPGAPGPPGSPGPAGPTGK
QGDRGEAGAQGPMGPSGPAGARGIAGPQGPRGDKGESGEQGERGLKGHRGFTGLQGLPGPPGPSGD
QGASGPAGPSGPRGPPGPVGPSGKDGSNGIPGPIGPPGPRGRSGETGPVGPPGSPGPPGPPGPPGP
GIDMSAFAGLGQREKGPDPMQYMRADEADSTLRQHDVEVDATLKSLNNQIESIRSPDGSRKNPART
CQDLKLCHPEWKSGDYWIDPNQGCTLDAMKVFCNMETGETCVYPNPATVPRKNWWSSKSKEKKHIW
FGETMNGGFHFSYGDGNLAPNTANVQMTFLRLLSTEGSQNITYHCKNSIAYLDEAAGNLKKALLIQ
GSNDVEMRAEGNSRFTYTALKDGCTKHTGKWGKTVIEYRSQKTSRLPIIDIAPMDIGGAEQEFGVD
IGPVCFL
000000000000000000000000000000000000000000000000000000000000000000
000000000000000000000000000000000000000000000000000000000000000000
000000000000000000000000000000000000000000000000000000000000000000
000000000000000000000000000000000000000000000000000000000000000000
000000000000000000000000000000000000000000000000000000000000000000
001111111100000000000000000000000000000000000000000000000000000000
000000000000000000000000000000000000000000000000000000000000000000
000000000000000000000000000000000000000000000000000000000000000000
000000000000000000000000000000000000000000000000000000000000000000

000000000000000000000000000000000000000000000000000000000000000000
000000000000000000000000000000000000000000000000000000000000000000
000000000000000000000000000000000000000000000000000000000000000000
000000000000000000000000000000000000000000000000000000000000000000
000000000000000000000000000000000000000000000000000000000000000000
000000000000000000000000000000000000000000000000000000000000000000
000000000000000000000000000000000000000000000000000000000000000000
000000000000000000000000000000000000000000000000000000000000000000
000000000000000000000000000000000000000000000000000000000000000000
000000000000000000000000000000000000000000000000000000000000000000
000000000000000000000000000000000000000000000000000000000000000000
000000000000000000000000000000000000000000000000000000000000000000
000000000000000000000000000000000000000000000000000000000000000000
0000000
>398 CARP2_CANAL P28871 Candidapepsin 2 precursor (EC 3.4.23.24) (Aspartate protease 2) (ACP 2) (Secreted aspartic protease 2). - Candida albicans (Yeast).
MFLKNIFIALAIALLVDATPTTTKRSAGFVALDFSVVKTPKAFPVTNGQEGKTSKRQAVPVTLHNE
QVTYAADITVGSNNQKLNVIVDTGSSDLWVPDVNVDCQVTYSDQTADFCKQKGTYDPSGSSASQDL
NTPFKIGYGDGSSSQGTLYKDTVGFGGVSIKNQVLADVDSTSIDQGILGVGYKTNEAGGSYDNVPV
TLKKQGVIAKNAYSLYLNSPDAATGQIIFGGVDNAKYSGSLIALPVTSDRELRISLGSVEVSGKTI
NTDNVDVLLDSGTTITYLQQDLADQIIKAFNGKLTQDSNGNSFYEVDCNLSGDVVFNFSKNAKISV
PASEFAASLQGDDGQPYDKCQLLFDVNDANILGDNFLRSAYIVYDLDDNEISLAQVKYTSASSISA
LT
000000000000000000000000000000000000000000000000000000000000000000
000000000000000000000000000000000000000000000000000000000000000000
000000000000000000001111111111111100000000000000000000000000000000
000000000000000000000000000000000000000000000000000000000000000000
000000000000000000000000000000000000000000000000000000000000000000
000000000000000000000000000000000000000000000000000000000000000000
00
>331 OMPB1_NEIMB P30690 Major outer membrane protein P.IB precursor (Protein IB) (PIB) (Porin) (Class 3 protein). - Neisseria meningitidis (serogroup B).
MKKSLIALTLAALPVAAMADVTLYGTIKAGVETSRSVFHQNGQVTEVTTATGIVDLGSKIGFKGQE
DLGNGLKAIWQVEQKASIAGTDSGWGNRQSFIGLKGGFGKLRVGRLNSVLKDTGDINPWDSKSDYL
GVNKIAEPEARLISVRYDSPEFAGLSGSVQYALNDNAGRHNSESYHAGFNYKNGGFFVQYGGAYKR
HHQVQEGLNIEKYQIHRLVSGYDNDALYASVAVQQQDAKLTDASNSHNSQTEVAATLAYRFGNVTP

RVSYAHGFKGLVDDADIGNEYDQVVVGAEYDFSKRTSALVSAGWLQEGKGENKFVATAGGVGLRHK
F
000000000000000000000000000000011111111111111111100000000000000000
011111111111111111111111111111000000000000000000000000000000000000
000000000000011111111111111111000000111111111111100000000000000000
000000000000000000000000000000000000000000000000000000011111111111
111111111111100000000000000000000000000000000000000000000000000000
0
>942 ENV_CAEVG   P31627 Env polyprotein precursor (Coat polyprotein) [Contains: Surface protein; Transmembrane protein]. - Caprine arthritis encephalitis virus (strain G63) (CAEV).
MDAGASYMRLTGEENWVEVTMDEEKERKGKDVQQGKYRPQVSKPIINRDTNTSFAYKGIFLWGIQI
TMWILLWTNMCVRAEDYITLISDPYGFSPIKNVSGVPVTCVTKEFARWGCQPLGAYPDPEIEYRNV
SQEIVKEVYQENWPWNTYHWPLWQMENVRYWLKENIAENKKRKNSTKKGIEELLAGTIRGRFCVPY
PFALLKCTKWCWYPAEIDQETGRARKIKINCTEARAVSCTEEMPLASIHRAYWDEKDRESMAFMNI
RACDSNLRCQKRPGGCVEGYPIPVGANIIPENMKYLRGQKSQYGGIKDKNGELKLPLTVRVWVKLA
NVSTWVNGTPPYWQNRINGSKGINGTLWGQLSGMHHLGFNLSQTGKWCNYTGKIKIGQETFSYHYK
PNWNCTGNWTQHPVWQVMRDLDMVEHMTGECVQRPQRHNITVDRNQTITGNCSVTNWDGCNCSRSG
NYLYNSTTGGLLVIICRNNNTITGIMGTNTNWTTMWRIYRNCSGCENATLDRKETGTLGGVANKNC
SLPHKNESNKWTCAPRQREGKTDSLYIAGGKKFWTREKAQYSCENNIGELDGMLHQQILLQKYQVI
KVRAYTYGVIEMPENYAKTRIINRRKRELSHTRKKRGVGLVIMLVIMAIVAAAGASLGVANAIQQS
YTKAAVQTLANATAAQQDALEATYAMVQHVAKGVRILEARVARVEAITDRIMLYQELDCWHYHQYC
VTSTRADVAKYINWTRFKDNCTWQQWERELQGYDGNLTMLLRESARQTQLAEEQVRRIPDVWESLK
EVFDWSGWFSWLKYIPIIVVGLVGCILIRAVICVCQPLVQIYRTLSTPTYQRVTVIMEKRADVAGE
NQDFGDGLEESDDSKTDQKVTVQKAWSRAWELWQNSPWKEPWKRSLLKLLILPLTMGIWINGRLGE
HLKNKKERVDCETWGKGD
000000000000000000000000000000000000000000000000000000000000000000
000000000000000000000000000000000000000000000000011111111111111000
000000000000000000000000000000000000000000000000000000000000000000
000000000000000000000000000000000000000000000000000000000000000000
000001111111111111100000000000000111111111111110000000000000000000
000000000000000000000000000000000000000000000000000000000000000000
000000000000000000000000000000000000000000000000011111111111111000
000011111111111110000000011111111111111111111100000000000000111111
111111111111111111111111111111111111000000000000000000000000000000
000000000000000000000000000000000000000000000000000000000000000000
000000000000000000000000000000000000000000000000000000000000000000
000000000000000000000000000000000000000000000000000000000000000000

000000000000000000000000000000000000000000000000000000000000000000
000000000000000000000000000000000000000000000000000000000000000000
000000000000000000
>1060 VP2_AHSV4   P32553 Outer capsid protein VP2. - African horse sickness virus 4 (AHSV-4) (African horse sickness virus - (serotype 4)).
MASEFGILMTNEKFDPSLEKTICDVIVTKKGRVKHKEVDGVCGYEWDETNHRFGLCEVEHDMSISE
FMYNEIRCEGAYPIFPRYIIDTLKYEKFIDRNDHQIRVDRDDNEMRKILIQPYAGEMYFSPECYPS
VFLRREARSQKLDRIRNYIGKRVEFYEEESKRKAILDQNKMSKVEQWRDAVNERIVSIEPKRGECY
DHGTDIIYQFIKKLRFGMMYPHYYVLHSDYCIVPNKGGTSIGSWHIRKRTEGDAKASAMYSGKGPL
NDLRVKIERDDLSRETIIQIIEYGKKFNSSAGDKQGNISIEKLVEYCDFLTTFVHAKKKEEGEDDT
ARQEIRKAWVKGMPYMDFSKPMKITRGFNRNMLFFAALDSFRKRNGVDVDPNKGKWKEHIKEVTEK
LKKAQTENGGQPCQVSIDGVNVLTNVDYGTVNHWIDWVTDIIMVVQTKRLVKEYAFKKLKSENLLA
GMNSLVGVLRCYMYCLALAIYDFYEGTIDGFKKGSNASAIIETVAQMFPDFRRELVEKFGIDLRMK
EITRELFVGKSMTSKFMEEGEYGYKFAYGWRRDGFAVMEDYGEILTEKVEDLYKGVLLGRKWEDEV
DDPESYFYDDLYTNEPHRVFLSAGKDVDNNITLRSISQAETTYLSKRFVSYWYRISQVEVTKARNE
VLDMNEKQKPYFEFEYDDFKPCSIGELGIHASTYIYQNLLVGRNRGEEILDSKELVWMDMSLLNFG
AVRSHDRCWISSSVAIEVNLRHALIVRIFSRFDMMSERETFSTILEKVMEDVKKLRFFPTYRHYYL
ETLQRVFNDERRLEVDDFYMRLYDVQTREQALNTFTDFHRCVESELLLPTLKLNFLLWIVFEMENV
EVNAAYKRHPLLISTAKGLRVIGVDIFNSQLSISMSGWIPYVERMCAESKVQTKLTADELKLKRWF
ISYYTTLKLDRRAEPRMSFKFEGLSTWIGSNCGGVRDYVIQMLPTRKPKPGALMVVYARDSRIEWI
EAELSQWLQMEGSLGLILVHDSGIINKSVLRARTLKIYNRGSMDTLILISSGVYTFGNKFLLSKLL
AKTE
000000000000000000000000000000000000000000000000000000000000000000
000000000000000000000000000000000000000000000000000000000000000000
000000000000000000000000000000000000000000000000000000000000000000
000000000000000000000000000000000000000000000000000000000000000000
000000011111111110000000000000000000000000000000000000000000000000
000000001111110000000000000000000000000000000000000011111100000000
000000000000000000000000000000000000000000000000000000000000000000
000000000000000000000000000000000000000000000000000000000000000000
000000000000000000000000000000000000000000000000000000000000000000
000000000000000000000000000000000000000000000000000000000000000000
000000000000000000000000000000000000000000000000000000000000000000
000000000000000000000000000000000000000000000000000000000000000000
000000000000000000000000000000000000000000000000000000000000000000
000000000000000000000000000000000000000000000000000000000000000000
000000000000000000000000000000000000000000000000000000000000000000

000000000000000000000000000000000000000000000000000000000000000000
0000
>395 VGLD_CHV1   P36342 Glycoprotein D precursor. - Cercopithecine herpesvirus 1 (CeHV-1) (Simian herpes B virus).
MGSGIAAVLLSLAVALARVPAGEGEYVPVERSLTRVNPGRFRGAHLPPLEQKTDPPDVRRVYHVQP
FVENPFQTPSVPVAVYYAVLERACRSVLLWAPTEAVQVVRGAPEATRSDARYNLTVAWYRTSDDCA
IPILVMEYAECQYDKPLGACPVRNLPRWSFYDSFSATGDDDLGLLMHAPAFETAGTYVRLVKVNGW
VEVTQFIFEHRGKGPCRYTLPLRILPAACLRAPVFEQGVTVDAIGMLPRFIPENQRIVAVYSLQAA
GWHGPKAPFTSTLLPPEVVETANVTRPELAPEERGTSRTPGDEPAPAVAAQLPPNWHVPEASDVTI
QGPAPAPSGHTGAVVGALAGAGLAAGVVVLAVYLVRRRGRAAGKHVRLPELLEEAHGPARRGAPY
000000000000000000000000000000000000000000000000000000000000000000
000000000000000000000000000000000000000000000000000000000000000000
000000000000000000000000000000000000000000000000000000000000000000
000000000000000000000000000000000000000000000000000000000000000000
000000000000000000000000000000000000000000000000000000000000000000
00000000000000000000000000000000000000000000000000000000111111111
>337 TALDO_HUMAN P37837 Transaldolase (EC 2.2.1.2). - Homo sapiens (Human).
MSSSPVKRQRMESALDQLKQFTTVVADTGDFHAIDEYKPQDATTNPSLILAAAQMPAYQELVEEAI
AYGRKLGGSQEDQIKNAIDKLFVLFGAEILKKIPGRVSTEVDARLSFDKDAMVARARRLIELYKEA
GISKDRILIKLSSTWEGIQAGKELEEQHGIHCNMTLLFSFAQAVACAEAGVTLISPFVGRILDWHV
ANTDKKSYEPLEDPGVKSVTKIYNYYKKFSYKTIVMGASFRNTGEIKALAGCDFLTISPKLLGELL
QDNAKLVPVLSAKAAQASDLEKIHLDEKSFRWLHNEDQMAVEKLSDGIRKFAADAVKLERMLTERM
FNAENGK
000000000011111111111111111111111111111111111000000000000000000000
000011111111111111111111111110000011111111111111100000000000000000
000000000000000000000000000000000000000000000000000000000000000000
001111111111111110000000000000001111111111111110000000000000001111
11111111111111111111100000000000000000000000011111111111111100000
0000000
>609 HEMA_RINDR  P41355 Hemagglutinin-neuraminidase (EC 3.2.1.18). - Rinderpest virus (strain RBOK) (RDV).
MSPPRDRVDAYYKDNFQFKNTRVVLNKEQLLIERPCMLLTVLFVMFLSLVGLLAIAGIRLHRAAVN
TAKINNDLTTSIDITKSIEYQVKDVLTPLFKIIGDEVGLRTPQRFTDLTKFISDKIKFLNPDKEYD
FRDINWCINPPERIKIDYDQYCAHTAAEDLITMLVNSSLTGTTVPRTSLVNLGRNCTGPTTTKGQF
SNISLTLSGIYSGRGYNISSMITITGKGMYGSTYLVGKYNQRARRPSKVWHQDYRVFEVGIIRELG
VGTPGFHMTNYLELPRQPELETCMLALGESKLAALCLADSPVALHYGRVGDDNKIRFVKLGVWASP
ADRDTLATLSAIDPTLDGLYITTHRGIIAAGTAIWAVPVTRTDDQVKMGKCRLEACRDRPPPFCNS

TDWEPLEAGRIPAYGVLTIKLGLADEPKVDIISEFGPLITHDSGMDLYTSFDGTKYWLTTPPLQNS
ALGTVNTLVLEPSLKISPNILTLPIRSGGGDCYIPTYLSDRADDDVKLSSNLVILPSRDLQYVSAT
YDISRVEHAIVYHIYSTGRLSSYYYPFKLPIKGDPVSLQIECFPWDRKLWCHHFCSVVDSGTGEQV
THIGVVGIKITCNGK
000000000000000000000000000000000000000000000000000000000000000000
000000000000000000000000000000000000000000000000000000000000000000
000000000000000000000000000000000000000000000000000000000000000000
000000000000000000000000000000000000000000000000000000000000000000
000000000000000000000000000000000000000000000000000000000000000000
000000000000000000000000000000000000000000000000000000000000000000
000000000000000000000000000000000000000000000000000000000000000000
000000000000000000000000000000000000000000000000000000000000000000
000000000000000000000000000000000000000011111111111111000000000000
000000000000000
>536 SPM1_MAGGR  P58371 Subtilisin-like proteinase Spm1 precursor (EC 3.4.21.-) (Serine protease of Magnaporthe 1). - Magnaporthe grisea (Rice blast fungus) (Pyricularia grisea).
MKSVILLSLAACAVAAPTAGVETIHDGAAPILSSSNAEAIPNAYIIKFKKHVDHKSAADHQMWIQK
VHGEREDERLELRKRGLFDSVNDAFTGLKHTYNVGSGFLGYAGHFDEETIEKVRRHPDVEAIERDT
IVHTMRYEEVKKDECNPDLEKGAPWGLSRVSHRESLSFSTYNKYLYSAEGGEGVDAYVIDTGTNID
HVDFEGRAHWGKTIPANDQDIDGNGHGTHCSGTVAGKKYGVAKKAQVYAVKVLKSNGSGTMSDVIA
GVDFAAKSHKAQVSAAKDGKRKGFKGSVANMSLGGGKTTLLDAAVNAAVDAGIHFAVAAGNDNADA
CNYSPAAAAKAVTVGASALDDSRAYFSNWGKCTDIFAPGLNIQSTWIGSKTAINTISGTSMASPHI
AGLLAYYLSLQPASDSEYSLATITPEKLKADLIKVGTVGILTDIPKDTPNVLAWNGGGCSNYFEIV
SKGGYKAKAQADKSSSLLDSVTELEKAIEHDFRVISGKVVKEASSMTGQAEKLSEKIHQAVDEELK
HFFGEARV
000000000000000000000000000000000000000000000000000000000000000000
000000000000000000000000000000000000000000000000000000000000000000
000000000000000000000000000000000000000000000000000011111111111111
111100000000111111111111111111111111000000000000000011111111111111
111100000011111111111111111111110000000000000000000000000000000000
000000000000000000000000000000000000000001111111111111111111110000
000000000000000000000000000011111111111111111111111111111111111111
111110000000000000000000000000000000000000000000000000000000000000
00000000
>310 ALL2_ASPFU  P79017 Major allergen Asp f 2 precursor (Asp f II). - Aspergillus fumigatus (Sartorya fumigata).
MAALLRLAVLLPLAAPLVATLPTSPVPIAARATPHEPVFFSWDAGAVTSFPIHSSCNATQRRQIEA

GLNEAVELARHAKAHILRWGNESEIYRKYFGNRPTMEAVGAYDVIVNGDKANVLFRCDNPDGNCAL
EGWGGHWRGANATSETVICDRSYTTRRWLVSMCSQGYTVAGSETNTFWASDLMHRLYHVPAVGQGW
VDHFADGYDEVIALAKSNGTESTHDSEAFEYFALEAYAFDIAAPGVGCAGESHGPDQGHDTGSASA
PASTSTSSSSSGSGSGATTTPTDSPSATIDVPSNCHTHEGGQLHCT
000000000000000000000000000000000000000000000000000000111111111111
100000000000000000000111111111111111000000000000000000000000000011
111111111111111001111111111111110000000000001111111111111111111111
111111111100000000000000001111111111111111000000000000000000000000
0000000000000000000000000000000011111111111111
>394 CARP_CANTR  Q00663 Candidapepsin precursor (EC 3.4.23.24)
(Aspartate protease) (ACP). - Candida tropicalis (Yeast).
MATIFLFTKNVFIALAFALFAQGLTIPDGIEKRTDKVVSLDFTVIRKPFNATAHRLIQKRSDVPTT
LINEGPSYAADIVVGSNQQKQTVVIDTGSSDLWVVDTDAECQVTYSGQTNNFCKQEGTFDPSSSSS
AQNLNQDFSIEYGDLTSSQGSFYKDTVGFGGISIKNQQFADVTTTSVDQGIMGIGFTAVEAGYNLY
SNVPVTLKKQGIINKNAYSCDLNSEDASTGKIIFGGVDNAKYTGTLTALPVTSSVELRVHLGSINF
DGTSVSTNADVVLDSGTTITYFSQSTADKFARIVGATWDSRNEIYRLPSCDLSGDAVVNFDQGVKI
TVPLSELILKDSDSSICYFGISRNDANILGDNFLRRAYIVYDLDDKTISLAQVKYTSSSDISAL
000000000000000000000000000000000000000000000000000000000000000000
000000000000000000000000000000000000000000000000000000000000000000
000000000000000000000000111111111111110000000000000000000000000000
000000000000000000000000000000000000000000000000000000000000000000
000000000000000000000000000000000000000000000000000000000000000000
0000000000000000000000000000000000000000000000000000000000000000
>212 OSPC2_BORBU Q08137 Outer surface protein C precursor (PC). -
Borrelia burgdorferi (Lyme disease spirochete).
MKKNTLSAILMTLFLFISCNNSGKGGDSASTNPADESAKGPNLTEISKKITDSNAFVLAVKEVETL
VLSIDELAKKAIGQKIDNNNGLAALNNQNGSLLAGAYAISTLITEKLSKLKNLEELKTEIAKAKKC
SEEFTNKLKSGHADLGKQDATDDHAKAAILKTHATTDKGAKEFKDLFESVEGLLKAAQVALTNSVK
ELTSPVVAESPKKP
000000000000000000000000000000000000000000000000000000000000000000
000000000000000000000000000000000000000000000000000000000000000000
000000000000000000000000000000000000000000000000000000000000000000
00001111111111
>193 MP70_MYCTU  P0A668 Immunogenic protein MPT70 precursor. -
Mycobacterium tuberculosis.
MKVKNTIAATSFAAAGLAALAVAVSPPAAAGDLVGPGCAEYAAANPTGPASVQGMSQDPVAVAASN
NPELTTLTAALSGQLNPQVNLVDTLNSGQYTVFAPTNAAFSKLPASTIDELKTNSSLLTSILTYHV
VAGQTSPANVVGTRQTLQGASVTVTGQGNSLKVGNADVVCGGVSTANATVYMIDSVLMPPA

000000000000000000000000000000111111111111111111111111111111111111
111100000000000000000000000000000011111111111111111111000000000000
0000000000000000000000000000000000000000000000000000000000000
>396 TRPB_ECO57  Q8X7B6 Tryptophan synthase beta chain (EC 4.2.1.20). - Escherichia coli O157:H7.
TTLLNPYFGEFGGMYVPQILMPALRQLEEAFVSAQKDPEFQAQFNDLLKNYAGRPTALTKCQNITA
GTNTTLYLKREDLLHGGAHKTNQVLGQALLAKRMGKTEIIAETGAGQHGVASALASALLGLKCRIY
MGAKDVERQSPNVFRMRLMGAEVIPVHSGSATLKDACNEALRDWSGSYETAHYMLGTAAGPHPYPT
IVREFQRMIGEETKAQILEREGRLPDAVIACVGGGSNAIGMFADFINETNVGLIGVEPGGHGIETG
EHGAPLKHGRVGIYFGMKAPMMQTEDGQIEESYSISAGLDFPSVGPQHAYLNSTGRADYVSITDDE
ALEAFKTLCLHEGIIPALESSHALAHALKMMRENPEKEQLLVVNLSGRGDKDIFTVHDILKARGEI
000000000000000000000000000000000000000000000000000000000000000000
000000000000000000000000000000000000000000000000000000000000000000
000000000000000000000000000000000000000000000000000000000000000000
000000000000000000000000000000000000000000000000000000000000000000
001111111111111111111111111111000000000000000000000000000000000000
000000000000000000000000000000000000000000000000000000000000000000
>262 MSA2_PLAFC  Q99317 Merozoite surface antigen 2 precursor (MSA-2) (Allelic form 1). - Plasmodium falciparum (isolate Camp / Malaysia).
MKVIKTLSIINFFIFVTFNIKNESKYSNTFINNAYNMSIRRSMAESKPPTGTGGSGSAGSGAGASA
GNGANPGADAERSPSTPATPATPATTTTTTTTNDAEASTSTSSENPNHKNAETNPKGKGEVQKPNQ
ANKETQNNSNVQQDSQTKSNVPPTQDADTKSPTAQPEQAENSAPTAEQTESPELQSAPENKGTGQH
GHMHGSRNNHPQNTSDSQKECTDGNKENCGAATSLLNNSSNIASINKFVVLISATLVLSFAIFI
000000000000000000000000001111111100000000000000000000000000000000
000000000000000000000000000000000000000000000000000000000000000000
000000000000000000000000000000000000000000000000000000000000000011
1111110000001111111100000000000000000000000000000000000000000000
>95 AAO62007
Mycobacterium_tuberculosis_6_kDa_early_secretory_antigenic_target_(ESAT-6)
MTEQQWNFAGIEAAASAIQGNVTSIHSLLDEGKQSLTKLAAAWGGSGSEAYQGVQQKWDATATELN
NALQNLARTISEAGQAMASTEGNVTGMFA
111111111111111111110000000000000000000000000000000000000000000000
00000000000000000000000000000
>200 AAQ55744
Drosophila_melanogaster_DNA_directed_RNA_polymerase_II_largest-subunit
INISQVIACVGQQNVEGKRIPFGFRKRTLPHFIKDDYGPESRGFVENSYLAGLTPSEFFFHAMGGR

EGLIDTAVKTAETGYIQRRLIKAMESVMVHYDGTVRNSVGQLIQLRYGEDGLCGETVEFQTLPTIK
LSNKAFEKRFRFDATNERYLRRIFNENILKELMGSGEVISYLEKEWDQLQKDREALRQIFPSGENK
VV
000000000000000000000000000000000001111111111111110000000000000000
000000000000000000000000000000000000000000000000000000000000000000
000000000000000000000000000000000000000000000000000000000000000000
00
>653 HS70_LEIDO Leishmania_donovani_Heat_Shock_protein_70-kDa
MTFDGAIGIDLGTTYSCVGVWQNERVDIIANDQGNRTTPSYVAFTDSERLIGDAAKNQVAMNPHNT
VFDAKRLIGRKFNDSVVQSDMKHWPFKVTTKGDDKPMIAVQYRGEEKTFTPEEISSMVLLKMKETA
EAYLGKQVKKAVVTVPAYFNDSQRQATKDAGTIAGLEVLRIINEPTRAIAYGLDKGDDGKERNVLI
FDLGGGTFDVSLLTIDGGIFEVKATNGDTHLGGEDFDNRLVTFFTEEFKRKNKGKNLASSHRALRG
LRTACERAKRTLSSATQATIEIDALFENVDFQATITRARFEELCGDLFRSTIQPVERVLQDAKMDK
RSVHDVVLVGGSTRIPKVQSLVSDFFGGKELNKSINPDEAVAYGAAVQAFILTGGKSKQTEGLLLL
DVTPLTLGIETAGGVMTALIKRNTTIPTKKSQIFSTYADNQPGVHIQVFEGERAMTKDCHLLGTFD
LSGIPPAPRGVPQIEVTFDLDANGILNVSAEEKGTGKRNQITITNDKGRLSKDEIERMVNDAMKYE
ADDRAQRDRVEAKNGLENYAYSMKNTLGDSNVSGKLDDSDKATLNKEIDVTLEWLSSNQEATKEEY
EHKQKELESVCNPIMTKMYQSMGGAGGGMPGGMPDMSGMSGGAGPAGGASSGPKVEEVD
000000000000000000000000000000000000000000000000000000000000000000
000000000000000000000000000000000000000000000000000000000000000000
000000000000000000000000000000000000000000000000000000000000000000
000000000000000000000000000000000000000000000000000000000000000000
000000000000000000000000000000000000000000000000000000000000000000
000000000000000000000000000000000000000000000000000000000000000000
000000000000000000000000000000000000000000000000000000000000000000
000000000000000000000000000000000000000000000000000000000000011111
111111111000000000000000000000000000000000000000000000000000000000
00000000000000000000000000000000000000000000000000000000000
>92 K11B_LEIIN Kinetoplastid_membrane_protein-11
MATTYEEFSAKLDRLGEEFNRKMQEQNAKFFADKPDESTLSPEMKEHYEKFERMIKEHTEKFNKKM
HEHSEHFKQKFAELLEQQKAAQYPSK
000000000000000000000000000001111111111111111111110000000000000000
00000000000000000000000000
>735 O56652 Adeno_associated_virus_2-VP-2
MAADGYLPDWLEDTLSEGIRQWWKLKPGPPPPKPAERHKDDSRGLVLPGYKYLGPFNGLDKGEPVN
EADAAALEHDKAYDRQLDSGDNPYLKYNHADAEFQERLKEDTSFGGNLGRAVFQAKKRVLEPLGLV
EEPVKTAPGKKRPVEHSPVEPDSSSGTGKAGQQPARKRLNFGQTGDADSVPDPQPLGQPPAAPSGL
GTNTMATGSGAPMADNNEGADGVGNSSGNWHCDSTWMGDRVITTSTRTWALPTYNNHLYKQISSQS

GASNDNHYFGYSTPWGYFDFNRFHCHFSPRDWQRLINNNWGFRPKRLNFKLFNIQVKEVTQNDGTT
TIANNLTSTVQVFTDSEYQLPYVLGSAHQGCLPPFPADVFMVPQYGYLTLNNGSQAVGRSSFYCLE
YFPSQMLRTGNNFTFSYTFEDVPFHSSYAHSQSLDRLMNPLIDQYLYYLSRTNTPSGTTTQSRLQF
SQAGASDIRDQSRNWLPGPCYRQQRVSKTSADNNNSEYSWTGATKYHLNGRDSLVNPGPAMASHKD
DEEKFFPQSGVLIFGKQGSEKTNVDIEKVMITDEEEIRTTNPVATEQYGSVSTNLQRGNRQAATAD
VNTQGVLPGMVWQDRDVYLQGPIWAKIPHTDGHFHPSPLMGGFGLKHPPPQILIKNTPVPANPSTT
FSAAKFASFITQYSTGQVSVEIEWELQKENSKRWNPEIQYTSNYNKSVNVDFTVDTNGVYSEPRPI
GTRYLTRNL
000000000000000000000000000000000000000000000000000000000000000000
000000000000000000000000000000000000001111111111111111111111111111
000000000000000000000000000000000000000000000000000000000000000000
000000000000000000000000000000000000000000000000000000000000000000
000000000000000000000000000000000000000000000000000000000000000000
000000000000000000000000000000000000000000000000000000000000000000
000000000000000000000000000000000000000000000000000000000000000000
000000000000000000000000000000000000000000000000000000000000000000
000000000000000000000000000000000000000000000000000000000000000000
000000000000000000000000000000000000000000000000000000000000000000
000000000000000000000000000000000000000000000000000000000000000000
000000000
>533 O92917 Adeno_associated_virus_2-VP-3
MATGSGAPMADNNEGADGVGNSSGNWHCDSTWMGDRVITTSTRTWALPTYNNHLYKQISSQSGASN
DNHYFGYSTPWGYFDFNRFHCHFSPRDWQRLINNNWGFRPKRLNFKLFNIQVKEVTQNDGTTTIAN
NLTSTVQVFTDSEYQLPYVLGSAHQGCLPPFPADVFMVPQYGYLTLNNGSQAVGRSSFYCLEYFPS
QMLRTGNNFTFSYTFEDVPFHSSYAHSQSLDRLMNPLIDQYLYYLSRTNTPSGTTTQSRLQFSQAG
ASDIRDQSRNWLPGPCYRQQRVSKTSADNNNSEYSWTGATKYHLNGRDSLVNPGPAMASHKDDEEK
FFPQSGVLIFGKQGSEKTNVDIEKVMITDEEEIRTTNPVATEQYGSVSTNLQRGNRQAATADVNTQ
GVLPGMVWQDRDVYLQGPIWAKIPHTDGHFHPSPLMGGFGLKHPPPQILIKNTPVPANPSTTFSAA
KFASFITQYSTGQVSVEIEWELQKENSKRWNPEIQYTSNYNKSVNVDFTVDTNGVYSEPRPIGTRY
LTRNL
000000000000000000000000000000000000000000000000000000000000000000
000000000000000000000000000000000000000000000000000000000000000000
000000000000000000000000000000000000000000000000000000000000000000
000000000000000000000000000000000000000000000000000000000000000000
000000000000000000000000000000000000000000000000000000000000000000
000000000000000000000000000000000000000000000000000000000000000000
000000000000000000000000000000000000000000000000000000000000000000
000000000000000000000000000000001111111111111111111100000000000000

00000
>379 P34_SOYBN Soybean_Gly_Bd_30K
MGFLVLLLFSLLGLSSSSSISTHRSILDLDLTKFTTQKQVSSLFQLWKSEHGRVYHNHEEEAKRLE
IFKNNSNYIRDMNANRKSPHSHRLGLNKFADITPQEFSKKYLQAPKDVSQQIKMANKKMKKEQYSC
DHPPASWDWRKKGVITQVKYQGGCGRGWAFSATGAIEAAHAIATGDLVSLSEQELVDCVEESEGSY
NGWQYQSFEWVLEHGGIATDDDYPYRAKEGRCKANKIQDKVTIDGYETLIMSDESTESETEQAFLS
AILEQPISVSIDAKDFHLYTGGIYDGENCTSPYGINHFVLLVGYGSADGVDYWIAKNSWGFDWGED
GYIWIQRNTGNLLGVCGMNYFASYPTKEESETLVSARVKGHRRVDHSPL
000000000000000000000000000000000000000000000000000000000000000000
000000000000000000000000000000000000000000000000000000000000000000
000000000000000000001111111111111111110000000000000000000000000000
000000000000000000000000000000000000001111111111111111100000000000
000000000000000000000000000000000000000000000000000000000000000000
0000000000000000000000000000000000000000000000000
>153 Q25763 Plasmodium_falciparum_RAP-1
KNVIVYNAIISGIHEKIKHFLKLVPRHNFLLDYHFNSVFEKEIKPAKKYSTSHIYFDPTVASYAYY
NLDRRTMVTIINDYFEAKKKELTVIVSRMKTDMLSLQNEESKIPNDKSANSKLATRLMKKFKAEIR
DFFKEMRIQYAKLINIRYRSH
000000000000000000000000000000000000000000000000000000000000000000
000000000000000000000000000000000000000000000000000001111111111111
111000000000000000000
>149 Q25784 Plasmodium_falciparum_Merozite_surface_antigen
MKKGSKVTELTSNAHMNGSIQKKKEENELENNKKLNHKNNLEENCVIKSKDVVKGGDQKNEGQAKK
KNNNNKKKKGTKENDMDKINQSGNTNDDKKKANKNNTTEGNEKNKGSNNKKYVGNKDENMKVELMD
VTKNNNSTNSGINNSSN
000000000000000000000000000000000000000000000000000000000000000000
000000000000000000000000000000000000000000000000000000000000000000
00001111111111000
>171 Q26003 Plasmodium_falciparum_Rhoptry_Protein_RAP-1
ASPSVVKTSTPSGTQTSGSKSSSPSSTKSSSPSNVKSASPHGESNSSEESTTKSSKRSASVAGIVG
ADEEAPPAPKNTLTPLEELYPTNVNLFNYKYSLNNMEENINILKNEGDLVAQKEEFEYDENMEKAK
QDKKKALEKIGKESDEEPFMFSENKFLENQVKERNVAGS
000000000000000000000000000000000000000000000000000000000000000000
000000000111111111111100000000000000000000000000111111111111111111
111111100000000000000000000000000000000
>574 Q26020
Plasmodium_falciparum_Thrombospondin_related_anonymous_protein_(TR
AP)

MNHLGNVKYLVIVFLIFFDLFLVNGRDVQNNIVDEIKYREEVCNDEVDLYLLMDCSGSIRRHNWVN
HAVPLAMKLIQQLNLNDNAIHLYASVFSNNAREIIRLHSDASKNKEKALIIIKSLLSTNLPYGKTN
LTDALLQVRKHLNDRINRENANQLVVILTDGIPDSIQDSLKESRKLSDRGVKIAVFGIGQGINVAF
NRFLVGCHPSDGKCNLYADSAWENVKNVIGPFMKAVCVEVEKTASCGVWDEWSPCSVTCGKGTRSR
KREILHEGCTSELQEQCEEERCLPKREPLDVPDEPEDDQPRPRGDNFAVEKPNENIIDNNPQEPSP
NPEEGKGENPNGFDLDENPENPPNPPNPPNPPNPPNPPNPDIPEQEPNIPEDSEKEVPSDVPKNPE
DDREENFDIPKKPENKHDNQNNLPNDKSDRYIPYSPLSPKVLDNERKQSDPQSQDNNGNRHVPNSE
DRETRPHGRNNENRSYNRKHNNTPKHPEREEHEKPDNNKKKAGSDNKYKIAGGIAGGLALLACAGL
AYKFVVPGAATPYAGEPAPFDETLGEEDKDLDEPEQFRLPEENEWN
000000000000000000000000000000000000000000000000000000000000000000
000000000000000000000000000000000000000000000000000000000000000000
000000000000000000000000000000000000000000000000000000000000000000
001111111111111111111100000000000000000000000000000000000000000000
000000000000000000000000000000000000000000000000000000000000000000
000000000000000000000000000000000000000000000000000000000000000000
000000000000000000000000111111111111111000000000000000000000000011
111111111111100000000000000000000000000000000000000000000000000000
0000000000000000000000000000000000000000000000
>278 Q47105 Escherichia_coli_Nonfimbrial_adhesin_CS31A
MKKTLIALAVAVSAVSGAAHAWTTGDFNGSFDMNGTITADAYKDKWEWMVGGALSFNNTIKEMTGD
SKLLTITQSEPAPILLGRTKEAFAASIVGVGAIPLIAFSDYEGNGVALQSSGDNGKGFFELPMKDD
SGNNLGSVKVNVTSAGLFSYSEISTGLVGITSVASGDNTSIYYGGLVSPAIRAGKDAASAVSKFGN
YNHTQLLGQLQAVNPNAGNRGQVNKNSAVSQNMVMTTGDVIASSYALGIDQGQTIEATFTNPVVST
TQWSAPLNVAVTYN
000000000000000000000000001111111111111111000000000000011111111111
111111111111111000000000111111111111111111111111111111111111111000
000000000000000000000000000001111111111111111111111111111111110000
011111111111111111111111111111011111111111111111111111111111111111
11100000000000
>593 Q51189 Neisseria_meningitidis_P64k
MALVELKVPDIGGHENVDIIAVEVNVGDTIPVDDTLITLETDKATMDVPAEVAGVVKEVKVKVGDK
ISEGGLIVVVEAERAASPKAAPVAAPAQEAPKAAAPAPQAAQFGGSADAEYDVVVLGGGPGGYSAA
FAAADEGLKVAIVERYKTLGGVCLNVGCIPSKALLHNAAVIDEVRHLAANGIKYPEPELDIDMLRA
YKNGVVSRLTGGLAGMAKGRKVDVIQGDGQFLDSHHLEVSLTAGDAYELAAPTGEKKIVAFKNCII
AAGSRVTKLPFIPEDPRIIDSSGALALKEVPGKLLIIGGGIIGLEMGTVYSTLGSRLDVVEMMDGL
MQGADRDLVKVWQKQNEYRFDNIMVNTKTVAVEPKEDGVYVTFEGANAPKEPQRYDAVLVAAGRAP
NGKLISAEKAGVAVTDRGFIEVDKQMRTNVPHIYAIGDIVGQPMLAHKAVHEGHVAAENCAGHKAY
FDARVIPGVAYTSPEVAWVGETELSAKASGRKITKANFPWAASGRAIANGCDNGFTKLIFDAETGR

IIGGGIVGPNGGDMIGEVCLAIEMGCDAADIGKTIHPHPTLGESIGMAAEVALGTCTDLPPQKKK
000000000000000000000000000000000000000000000000000000000000000000
000000000000000000000000000000000000000000000000000000000000000000
000000000000000000000000000000000000000000000000000000000000000000
000000000000000000000000000000000000000000000000000000000000000000
000000000000000000000000000000000000000000000000000000000000000000
000000000000000000000000000000000000000000000000000000000000000000
000000000000000000000000000000000000000000000000000000000000000000
0000000000000000000000000000000000000000000000000000000111111111111
11111111100000000000000000000000000000000000000000000000000000000
>90 Q80883 Human_papillomavirus_type_16_E6_protein
VYDFAFRDLCIVYRDGNPYAVCDKCLKLYSKISEYRHYCYSLYGTTLEQQYNKPLCDLLIRCINCQ
KPLCPEEKQRHLDKKQRFHNIRGR
000000000000000000000000000011111111111111111111000000000000000000
000000000000000000000000
>494 Q81005 Human_papillomavirus_type_16_Major_capsid_protein_L1
TVYLPPVPVSKVVSTDEYVARTNIYYHAGTSRLLAVGHPYFPIKKPNNNKILVPKVSGLQYRVFRI
HLPDPNKFGFPDTSFYNPDTQRLVWACVGVEVGRGQPLGVGISGHPLLNKLDDTENASAYAANAGV
DNRECISMDYKQTQLCLIGCKPPIGEHWGKGSPCTNVAVNPGDCPPLELINTVIQDGDMVDTGFGA
MDFTTLQANKSEVPLDICTSICKYPDYIKMVSEPYGDSLFFYLRREQMFVRHLFNRAGTVGENVPD
DLYIKGSGSTANLASSNYFPTPSGSMVTSDAQIFNKPYWLQRAQGHNNGICWGNQLFVTVVDTTRS
TNMSLCAAISTSETTYKNTNFKEYLRHGEEYDLQFIFQLCKITLTADVMTYIHSMNSTILEDWNFG
LQPPPGGTLEDTYRFVTSQAIACQKHTPPAPKEDPLKKYTFWEVNLKEKFSADLDQFPLGRKFLLQ
AGLKAKPKFTLGKRKATPTTSSTSTTAKRKKR
000000000000000000000000000000000000000000000000000000000000000000
000000000000000000000000000000000000000000000000000000000000000000
000000000000000000000000000000000000000000000000000000000000000000
000000000000000000000000000000000000000000000000000000000000000000
000000000000000000000000000000000000000000000000000000000000000000
000000000000000000000000000000000000000000000000000000000000000000
000000000000000000000000000000000000000000000000000000000000111111
11110000000000000000000000000000
>77 Q8B5P5 Human_papillomavirus_type_16_E7_protein
MHGDTPTLHEYMLDLQPETTDLYCYEQLSDSSEEEDEIDGPAGQAEPDRAHYNIVTFCCKCDSTLR
LCVQSTHVDIR
011111111111111111110000000000000000000000000111111111111111111111
00000000000
>198 Q8QQW1 Grapevine_virus_A_capsid_protein

MAHYAKRVEIRAIIKELVLAKAQPTEDASESGYDRNMYLNTLFGYIALVGTSKKAVHYGEVDIVGP
KASKKTGIDPRGKMVVSEMVGRMRTLSVAVSEGPVKGATLRQMCEPFAQNAYDFLVVMAEMGTYSQ
LASKMTRSGFKSPQVMFDFASGLDLKTLTLQEATVIQSMHSRLFRTEGAKGVFNAQSSVGEQAVEI
000000000000000000000000000000011111111110000000000000000000111111
111111111111111000000000000000000000000000000000000000000000000000
000000000001111111111110000000000000000000000000000000000000000000
>488 Q8UZC2 Dengue_virus_type_2_E_Protein
MRCIGISNRDFVEGVSGGSWVDIVLEHGSCVTTMEKNKPTLDFELIKTEAKQPATLRKYCIEAKLT
NTTTESRCPTQGEPSLNEEQDKRFVCKHSMVDRGWGNGCGLFGKGGIVTCAMFTCKKNMEGKIVQP
ENLEYTIVITPHSGEEHAVGKEIKITPQSSITEAELTGYGTVTMECSPRTGLDFNEMVLLQMEDKA
WLVHRQWFLDLPLPWLPGADTQGSNWIQKETLVTFKNPHAKKQDVVVLGSQEGAMHTALTGATEIQ
MSSGNLLFTGHLKCRLRMDKLQLKGMSYSMCTGKFKIVKEIAETQHGTIVIRVQYEGDGSPCKIPF
EIMDLEKRHVLGRLITVNPIVTEKDSPVNIEAEPPFGDSYIIIGVEPGQLKLNWFKKGSSIGQMFE
TTMRGAKRMAILGDTAWDFGSLGGVFTSIGKALHQVFGAIYGAAFSGVSWTMKILIGVIITWIGMN
SRSTSLSVSLVLVGVITLYLGVMVQA
000000000000000000000000000000000000000000000000000000000000000000
000000000000000000000000000000000000000000000000000000000000000000
000000000000000000000000000000000000000000000000000000000000000000
000000000000000000000000000000000000000000000000000000000000000000
000000000000000000000000000000000000000000000000000000000000000000
000000000001111111111100000000000000000000000000000000000000000000
000000000000000000000000000000000000000000000000000000000000000000
00000000000000000000000000
>397 Q93P53
Chlamydia_trachomatis_Major_outer_membrane_protein,_serovar_C
MKKLLKSVLVFAALSSASSLQALPVGNPAEPSLMIDGILWEGFGGDPCDPCTTWCDAISMRVGYYG
DFVFDRVLKTDVNKEFQMGAAPTTSDVAGLQNDPTTNVARPNPAYGKHMQDAEMFTNAAYMALNIW
DRFDVFCTLGATTGYLKGNSASFNLVGLFGTKTQSSSFNTAKLIPNTALNEAVVELYINTTFAWSV
GARAALWECGCATLGASFQYAQSKPKVEELNVLCNASEFTINKPKGYVGAEFPLNITAGTEAATGT
KDASIDYHEWQASLALSYRLNMFTPYIGVKWSRVSFDADTIRIAQPKLAEAILDVTTLNPTIAGKG
SVVSAGTDNELADTMQIVSLQLNKMKSRKSCGIAVGTTIVDADKYAVTVEARLIDERAAHVNAQFR
F
000000000000000000000000000000000000000000000000000000000000000000
000000000000000000000000000000000000000000000000000000000000000000
000000000000000000000000000000000000000000000000000000000000000000
000000000000000000000000000000000000000000000000000000000000000000
000000000000000000000000000000000000000000000001111111111111111111
111111111111000000000000000000000000000000000000000000000000000000

0
>274 Q9JNQ0
Group_A_M1_Streptococcus_inhibitor_of_complement(Sic)_extracellula
r_protein
MNIRNKIENSKTLLFTSLVAVALLGATQPVSAETYTSRNFDWSGDDWSGDDWPEDDWSGDGLSQYG
WSKYGWSSDKEEWPEDWPEDDWSSDKKDETEDKTRPPYGEALGTGYEKRDDWGGPGTVATDPYTPP
YGGALGTGYEKRDDWRGPGHIPKPENEQSPNPSHIPEPPQIEWPQWNGFDGFDGLSSGPSDWGQSE
DTSRFPSEPRVTEKPQHTPQKNPQESDFDRGFSAGLKAKNSGRGIDFEGFQYGGWSDEYKKGYMQA
FGTPYTPSAT
000000000000000000000000000000000000000000001111111111111110011111
111111111111111111111111100000000000000000111111111111110000000000
000000000000000000000000000000000001111111111111111111111110000000
000000001111111111111110000000000000000000000000111111111111111000
0000000000
>238 Q9L8G3 Mycoplasma_agalactiae_AvgC_(30-37)
MKKSKFLLLGSVASMAAIPFVAAKCGGTKEENKKPAEMPGGTEQPGKPGDTDQPAQPNPGTTPSTP
AKPGKTPERMAQDTDVSGADISGSIKTPDTTATKTKLSDALKTIANNNLGKVQVSKEDKDKKDKED
KTGGSTSTDNKDPKSPGESESVPMESSKIDLKKLEESVKQELNKLAKKDVLSEDVLDVLKKAKGLE
SLKLGDLSKVEFNEKDKKLTIESHKDSKLVTGKYEFTLEK
000000000000000000000000000001111111100000000000000000000000000000
000000000000000000000000000000000000000000000000000000000000000000
000000000000000000000000000000000000000000000000000000000000000000
0000000000000000000000000000000000000000
>771 Q9NGD0 Leishmania_infantum_GRP94
MANSSLLRVVLVALLLLGSVTVSAGDGRGTPIAFQAEVSKMLDILVNSLYTNRAVFLRELISNGSD
ALDKIRVLYLTSPKEPLTKDGEAPTMDLRISFDKEKSELILRDGGVGMTKEELAKHLGSLGTSGTK
HFLEKLQEGVGAGGGDQNNLIGQFGVGFYSVFLVGDRVRVASKSDDSDEQYVWESKGDGQYFLYPD
PRGNTLGRGTEITIELKPDAEQFLSAETIKKTIHQYSEFINFPIYVQEEVEVASTAATPEPAAEEG
SLDEGAVEEDPDKEGDTQGVVKERRWTLVNENRPIWTRPIGNVTEEEYHTFYKAFSGDYRDPLYFN
HFKVEGEVDFDSILFVPTTVDPASFSDDNSVPNTNIKLYVRRVFITDEFRDLLPRYLNFVKGIVDS
NDLPLNVSREVLQESRILRVIKKKLVRKTLSMFADIAAQDEAIANGKQVESPAPSGHTHLKKPAYT
KFWELYGKHLRLGVMLDSNNRNRLTKLFRYKSSRSESEYISLQTYVDRMKKGQKGIYYLSGDSVAR
IKKSPVLEDAVNHDVEVIFMTDAIDEYVVSQLTDFAGKKLINLAKEGVQLEESDARQRVADRKRKE
KYDSFFTHLRALFGYSEVRKVILTKRMTNEAFLVSSGENQITARLASIMRGQSMSLANQQMTAERV
LEVNYRHPLVDEMFKRFTVDEDDEVATDIAWVLYDTANLQAEFPVADVAAYSKRINRLLRSSVDLS
ADDSLLPPDDAEYTVSDTEAEEEEEQPKVDANADEEAEAVGEDDL
000000000000000000001111111111111111111100000000000000000000000000
000000000000000000000000000000000000000000000000000000000000000000

000000000000000000000000000000000000000000000000000000000000000000
001111111111111111111100000000000000000000000000000000000000001111
111111111111111111111111111111111111000000000000000000000000000000
000000000000000000000000000000111111111111111111110000000000000000
000000000000000000000000000000000000000000001111111111111111111100
000000000000000000111111111111111111110000000000000000000000000000
000000000000000000000000000000000000000000000000000011111111111111
111111000000000000000000001111111111111111111111111111111111111111
000000000000000000000000000000000000000000000000000000000000111111
111111111111111111111111111111111100000000000
>374 SBP_CRYJA Japanese_Cedar_Pollen_Major_Allergen_(Cry_j_1)
MDSPCLVALLVLSFVIGSCFSDNPIDSCWRGDSNWAQNRMKLADCAVGFGSSTMGGKGGDLYTVTN
SDDDPVNPAPGTLRYGATRDRPLWIIFSGNMNIKLKMPMYIAGYKTFDGRGAQVYIGNGGPCVFIK
RVSNVIIHGLHLYGCSTSVLGNVLINESFGVEPVHPQDGDALTLRTATNIWIDHNSFSNSSDGLVD
VTLSSTGVTISNNLFFNHHKVMLLGHDDAYSDDKSMKVTVAFNQFGPNCGQRMPRARYGLVHVANN
NYDPWTIYAIGGSSNPTILSEGNSFTAPNESYKKQVTIRIGCKTSSSCSNWVWQSTQDVFYNGAYF
VSSGKYEGGNIYTKKEAFNVENGNATPQLTKNAGVLTCSLSKRC
000000000000000000000000000000000000000000000000000000000000000000
000000000000000000000000000000000000000000000000000000000000000000
000000000000000000000000000001111111111111111111000000000000000000
000000000000000000000000000000000000000000000000000000000000000000
000000000000000000000000000000000000000000000000000000000000000000
00000000000000000000000000000000000000000000
