# Supplementary material for: Prediction of B-cell epitopes using evolutionary information and propensity scales
Source: BMC Bioinformatics. 2013 Jan 21;14(Suppl 2):S10. doi: 10.1186/1471-2105-14-S2-S10 (PMC3549808; doi:10.1186/1471-2105-14-S2-S10)
Supplement: Additional file 3 — The AntiJen#2 dataset. [file 1471-2105-14-S2-S10-S3.pdf]

Additional file 3. AntiJen dataset #2.

> P04921 GLPC_HUMAN
MWSTRSPNSTAWPLSLEPDPGMASASTTMHTTTIAEPDPGMSGWPDGRMETSTPTIMDIVVIAGVI
AAVAIVLVSLLFVMLRYMYRHKGTYHTNEAKGTEFAESADAALQGDPALQDAGDSSRKEYFI
000000000000111111111111000000000000000000000000000000000000000000
00000000000000000000000000000000000000000111111111100000000000
> ETXB_STAAU ETXB_STAAU
MYKRLFISHVILIFALILVISTPNVLAESQPDPKPDELHKSSKFTGLMENMKVLYDDNHVSAINVK
SIDQFLYFDLIYSIKDTKLGNYDNVRVEFKNKDLADKYKDKYVDVFGANYYYQCYFSKKTNDINSH
QTDKRKTCMYGGVTEHNGNQLDKYRSITVRVFEDGKNLLSFDVQTNKKKVTAQELDYLTRHYLVKN
KKLYEFNNSPYETGYIKFIENENSFWYDMMPAPGDKFDQSKYLMMYNDNKMVDSKDVKIEVYLTTK
KK
000000000000000000000000000111111111111111101111111111111110000011
111111111111111111111100111111111111111111111110000000011111111111
110000000000000011111111111110111111110000000000000000000000000011
111111111111111111111111111111111111111111111101111111111111111111
11
> P04153 UMUD_ECO57
MLFIKPADLREIVTFPLFSDLVQCGFPSPAADYVEQRIDLNQLLIQHPSATYFVKASGDSMIDGGI
SDGDLLIVDSAITASHGDIVIAAVDGEFTVKKLQLRPTVQLIPMNSAYSPITISSEDTLDVFGVVI
HVVKAMR
000000000000000000000000000000000111111111111110000000000000000000
000000000000000000000000000000000000000000000000000000000000000000
0000000
> P81402 NLTP1_PRUPE
ITCGQVSSALAPCIPYVRGGGAVPPACCNGIRNVNNLARTTPDRQAACNCLKQLSASVPGVNPNNA
AALPGKCGVHIPYKISASTNCATVK
000000000011111111111111111111111111111111111000000000000000000001
1111111110000011111111111
> P01111 RASN_HUMAN
MTEYKLVVVGAGGVGKSALTIQLIQNHFVDEYDPTIEDSYRKQVVIDGETCLLDILDTAGQEEYSA
MRDQYMRTGEGFLCVFAINNSKSFADINLYREQIKRVKDSDDVPMVLVGNKCDLPTRTVDTKQAHE
LAKSYGIPFIETSAKTRQGVEDAFYTLVREIRQYRMKKLNSSDDGTQGCMGLPCVVM
000000000000000000000000001111111111111100000000000000000000000000
000000000000000000000000000000000000000000000000000000000000000000
000000000000000000000000000000000000000000000000000000000
> Q57165 ESXA_MYCBO

MTEQQWNFAGIEAAASAIQGNVTSIHSLLDEGKQSLTKLAAAWGGSGSEAYQGVQQKWDATATELN
NALQNLARTISEAGQAMASTEGNVTGMFA
001111111111111000000000000000000000000000000000000000000000000000
00000000000000000000000000000
> P01574 IFNB_HUMAN
MTNKCLLQIALLLCFSTTALSMSYNLLGFLQRSSNFQCQKLLWQLNGRLEYCLKDRMNFDIPEEIK
QLQQFQKEDAALTIYEMLQNIFAIFRQDSSSTGWNETIVENLLANVYHQINHLKTVLEEKLEKEDF
TRGKLMSSLHLKRYYGRILHYLKAKEYSHCAWTIVRVEILRNFYFINRLTGYLRN
000000000000000000000111111111111000000000000000000000000000000000
000000000000000000000000000000000000000000000000000000000000000000
0000000001111111111110000000000000000001111111111110000
> P02754 LACB_BOVIN
MKCLLLALALTCGAQALIVTQTMKGLDIQKVAGTWYSLAMAASDISLLDAQSAPLRVYVEELKPTP
EGDLEILLQKWENGECAQKKIIAEKTKIPAVFKIDALNENKVLVLDTDYKKYLLFCMENSAEPEQS
LACQCLVRTPEVDDEALEKFDKALKALPMHIRLSFNPTQLEEQCHI
000000000000000000000000000011111111111111000000001111111111111111
111111111110000000000000000000111111111111111111111111111111111000
1111111111111111111111000000000000111111111111
> P02752 RBP_CHICK
MLRFAITLFAVITSSTCQQYGCLEGDTHKANPSPEPNMHECTLYSESSCCYANFTEQLAHSPIIKV
SNSYWNRCGQLSKSCEDFTKKIECFYRCSPHAARWIDPRYTAAIQSVPLCQSFCDDWYEACKDDSI
CAHNWLTDWERDESGENHCKSKCVPYSEMYANGTDMCQSMWGESFKVSESSCLCLQMNKKDMVAIK
HLLSESSEESSSMSSSEEHACQKKLLKFEALQQEEGEERR
000000000000000000000000001111111100000000000000000000000011111111
000000111111110000111111111111111100111111110000000000000011111110
000000000000000000111111110000000000001111111100000000001111111100
0000000000000000001111111100000000000000
> P17082 RRAS2_HUMAN
MAAAGWRDGSGQEKYRLVVVGGGGVGKSALTIQFIQSYFVTDYDPTIEDSYTKQCVIDDRAARLDI
LDTAGQEEFGAMREQYMRTGEGFLLVFSVTDRGSFEEIYKFQRQILRVKDRDEFPMILIGNKADLD
HQRQVTQEEGQQLARQLKVTYMEASAKIRMNVDQAFHELVRVIRKFQEQECPPSPEPTRKEKDKKG
CHCVIF
000000000000000000000000000000000000000011111100000000000000000000
000000000000000000000000000000000000000000000000000000000000000000
000000000000000000000000000000000000000000000000000000000000000000
000000
> P79085 ALTA1_ALTAL
MQFTTIASLFAAAGLAAAAPLESRQDTASCPVTTEGDYVWKISEFYGRKPEGTYYNSLGFNIKATN

GGTLDFTCSAQADKLEDHKWYSCGENSFMDFSFDSDRSGLLLKQKVSDDITYVATATLPNYCRAGG
NGPKDFVCQGVADAYITLVTLPKSS
000000000000000000000000000000000000000111111111100011111111111000
000000000000000000011111111110000000000000000000000111111111110000
0000000000000000000000000
> P02023 HBB_HUMAN
MVHLTPEEKSAVTALWGKVNVDEVGGEALGRLLVVYPWTQRFFESFGDLSTPDAVMGNPKVKAHGK
KVLGAFSDGLAHLDNLKGTFATLSELHCDKLHVDPENFRLLGNVLVCVLAHHFGKEFTPPVQAAYQ
KVVAGVANALAHKYH
000000000001111111111111111111111111111111111100000000000000000000
000001111111111111110000000000000001111111111111111111111111000001
111111111111111
> P02185 MYG_PHYCA
VLSEGEWQLVLHVWAKVEADVAGHGQDILIRLFKSHPETLEKFDRFKHLKTEAEMKASEDLKKHGV
TVLTALGAILKKKGHHEAELKPLAQSHATKHKIPIKYLEFISEAIIHVLHSRHPGDFGADAQGAMN
KALELFRKDIAAKYKELGYQG
000000000000001111111100000000000000000001111111111111011111110000
000000000000000000000000000111111000000000000011111111111111000000
001111110000111111100
> P01922 HBA_HUMAN
MVLSPADKTNVKAAWGKVGAHAGEYGAEALERMFLSFPTTKTYFPHFDLSHGSAQVKGHGKKVADA
LTNAVAHVDDMPNALSALSDLHAHKLRVDPVNFKLLSHCLLVTLAAHLPAEFTPAVHASLDKFLAS
VSTVLTSKYR
000000000001111111111111110000000000000000000000000111111111111111
000000000000000111111111111111000001111111111111110000011111111111
1111000000
> P01911 HB2G_HUMAN
GDTRPRFLWQPKRECHFFNGTERVRFLDRYFYNQEESVRFDSDVGEFRAVTELGRPDAEYWNSQKD
ILEQARAAVDTYCRHNYGVVESFTVQRRVQPKVTVYPSKTQPLQHHNLLVCSVSGFYPGSIEVRWF
LNGQEEKAGGVSTGLIQDDWTFQTLVMLETVPRSGEVYTCQVEHPSVTSPLTVEWRARSESAQSKM
111111111111111111000000000000000000000000000001111111111111111111
111111100000000011111111111000000000111111111111111100001111111111
100000000000000000001111111111100000000000000111111111111111111111
> P09621 CH10_MYCTU
AKVNIKPLEDKILVQANEAETTTASGLVIPDTAKEKPQEGTVVAVGPGRWDEDGEKRIPLDVAEGD
TVIYSKYGGTEIKYNGEEYLILSARDVLAVVSK
000000000000000000000000000000000000000000000011111111111110000000
000000000000000000000000000000000

> P02724 GLPA_HUMAN
MYGKIIFVLLLSAIVSISASSTTGVAMHTSTSSSVTKSYISSQTNDTHKRDTYAATPRAHEVSEIS
VRTVYPPEEETGERVQLAHHFSEPEITLIIFGVMAGVIGTILLISYGIRRLIKKSPSDVKPLPSPD
TDVPLSSVEIENPETSDQ
000000000000000000000000000000000000000000000000000001111111111100
011111111111100000000000000000000000000000000000000000000000000000
000111111111100000

> P26948 CAF1_YERPE
MKKISSVIAIALFGTIATANAADLTASTTATATLVEPARITLTYKEGAPITIMDNGNIDTELLVGT
LTLGGYKTGTTSTSVNFTDAAGDPMYLTFTSQDGNNHQFTTKVIGKDSRDFDISPKVNGENLVGDD
VVLATGSQDFFVRSIGSKGGKLAAGKYTDAVTVTVSNQ
000000000000000000000000000000000000000000000000000000000000000000
000000000000000000000000000000000000000000000000000000000000000000
00000000011111111111111111111111100000

> P02399 RLA2_ARTSA
MRYVAAYLLAALSGNADPSTADIEKILSSVGIECNPSQLQKVMNELKGKDLEALIAEGQTKLASMP
TGGAPAAAAGGAATAPAAEAKEAKKEEKKEESEEEDEDMGFGLFD
000000000000000000000000000000000000000000000000000000000000000000
000000000000000000000001111111111111111111111

> P01484 SCX2_ANDAU
MNYLVMISLALLFVTGVESVKDGYIVDDVNCTYFCGRNAYCNEECTKLKGESGYCQWASPYGNACY
CYKLPDHVRTKGPGRCHGR
000000000000000000000011111111000000000000000111111111000000000000
0000111111111100000

> P03146 CORA_HBVYW
MDIDPYKEFGATVELLSFLPSDFFPSVRDLLDTASALYREALESPEHCSPHHTALRQAILCWGELM
TLATWVGVNLEDPASRDLVVSYVNTNMGLKFRQLLWFHISCLTFGRETVIEYLVSFGVWIRTPPAY
RPPNAPILSTLPETTVVRRRGRSPRRRTPSPRRRRSQSPRRRRSQSRESQC
000000000000000000000000000000000000000000000000000000000000000000
000000011111111110000000000000000000000000000000000000000000000000
000000000000000000000000000000000000000000000000000

> P13856 RSR1_YEAST
MRDYKLVVLGAGGVGKSCLTVQFVQGVYLDTYDPTIEDSYRKTIEIDNKVFDLEILDTAGIAQFTA
MRELYIKSGMGFLLVYSVTDRQSLEELMELREQVLRIKDSDRVPMVLIGNKADLINERVISVEEGI
EVSSKWGRVPFYETSALLRSNVDEVFVDLVRQIIRNEMESVAVKDARNQSQQFSKIESPSTRLPSS
AKQDTKQSNNKQSSKGLYNKSSQGQAKVKQSTPVNEKHKPSHAVPKSGSSNRTGISATSQQKKKKK
NASTCTIL
000000000000000000000000000001111111000000000000000000000000000000

000000000000000000000000000000000000000000000000000000000000000000
000000000000000000000000000000000000000000000000000000000000000000
000000000000000000000000000000000000000000000000000000000000000000
00000000
> P04980 TOX4_BORPA
MLRRFPTRTTAPGQGGARRSRVRALAWLLASGAMTHLSPALADVPYVLVKTNMVVTSVAMKPYEVT
PTRMLVCGIAAKLGAAASSPDAHVPFCFGKDLKRPGSSPMEVMLRAVFMQQRPLRMFLGPKQLTFE
GKPALELIRMVECSGKQDCP
000000000000000000000000000000000000000000000000000000000000111111
111111100111111111111111110000000000000000000000000000000000000000
11111111111111111100
> P16055 TA15_TREPA
MVKRGRFALCLAVLLGACSFSSIPNGTYRATYQDFDENGWKDFLEVTFDGGKMVQVVYDYQHKEGR
FKSQDADYHRVMYASSGIGPEKAFRELADALLEKGNPEMVDVVTGATVSSQSFRRLGRALLQSARR
GEKEAIISR
000000000111111111111111111111111100000000000000111111111111111111
101111111111111111111111111111100000001111111111111111111111111111
111000000
> P22629 SAV_STRAV
MRKIVVAAIAVSLTTVSITASASADPSKDSKAQVSAAEAGITGTWYNQLGSTFIVTAGADGALTGT
YESAVGNAESRYVLTGRYDSAPATDGSGTALGWTVAWKNNYRNAHSATTWSGQYVGGAEARINTQW
LLTSGTTEANAWKSTLVGHDTFTKVKPSAASIDAAKKAGVNNGNPLDAVQQ
000000000000000000000000000000000000000111111111000000001111111111
100000000000000000000000000000000000011111111111100001111111100000
000001111111100000000000000000000000000000000000000
> P00374 DYR_HUMAN
VGSLNCIVAVSQNMGIGKNGDLPWPPLRNEFRYFQRMTTTSSVEGKQNLVIMGKKTWFSIPEKNRP
LKGRINLVLSRELKEPPQGAHFLSRSLDDALKLTEQPELANKVDMVWIVGGSSVYKEAMNHPGHLK
LFVTRIMQDFESDTFFPEIDLEKYKLLPEYPGVLSDVQEEKGIKYKFEVYEKND
000000000000011111111000001111111000000000001111110000000000000000
111111100000000000000000000000000000000000000000000000000000000000
111111000000000000001111110000000000000000111111000000
> P02247 HEMTM_THEZO
GWEIPEPYVWDESFRVFYEQLDEEHKKIFKGIFDCIRDNSAPNLATLVKVTTNHFTHEEAMMDAAK
YSEVVPHKKMHKDFLEKIGGLSAPVDAKNVDYCKEWLVNHIKGTDFKYKGKL
000111111100000111111000000000000000000000000000000011111111101111
1111111000001111111101111111110000000000001111111000
> P17522 COAT_PLRV1

MSTVVVKGNVNGGVQQPRMRRRQSLRRRANRVQPVVMVTAPGQPRRRRRRRGGNRRSRRTGVPRGR
GSSETFVFTKDNLVGNTQGSFTFGPSLSDCPAFKDGILKAYHEYKITSILLQFVSEASSTSSGSIA
YELDPHCKVSSLQSYVNKFQITKGGAKTYQARMINGVEWHDSSEDQCRILWKGNGKSSDSAGSFRV
TIKVALQNPK
11111111110000000000000000000000000111111100000011111110000000000
00011111111000011111111000000000000000000001111110111111000111111
10000000000000000000000000000000011111111111100011111111100001111
1111110000
> P04389 RNMG_ASPFU
MVAIKNLFLLAATAVSVLAAPSPLDARATWTCINQQLNPKTNKWEDKRLLYSQAKAESNSHHAPLS
DGKTGSSYPHWFTNGYDGNGKLIKGRTPIKFGKADCDRPPKHSQNGMGKDDHYLLEFPTFPDGHDY
KFDSKKPKEDPGPARVIYTYPNKVFCGIVAHQRGNQGDLRLCSH
00000000000000000000000000000111111111111111111100000000000000000
00000000001111111111000011111111110111111111100111111111111111111
00000000001111111111111110111111111111111111
> Q99317 MSA2_PLAFC
MKVIKTLSIINFFIFVTFNIKNESKYSNTFINNAYNMSIRRSMAESKPPTGTGGSGSAGSGAGASA
GNGANPGADAERSPSTPATPATPATTTTTTTTNDAEASTSTSSENPNHKNAETNPKGKGEVQKPNQ
ANKETQNNSNVQQDSQTKSNVPPTQDADTKSPTAQPEQAENSAPTAEQTESPELQSAPENKGTGQH
GHMHGSRNNHPQNTSDSQKECTDGNKENCGAATSLLNNSSNIASINKFVVLISATLVLSFAIFI
00000000000000000000000000111111110000000000000000000000000000000
00000000000000000000000000000000000000000000000000000000000000000
00000000000000000000000000000000000000000000000000000000000000011
1111110000001111111100000000000000000000000000000000000000000000
> P02701 AVID_CHICK
MVHATSPLLLLLLLSLALVAPGLSARKCSLTGKWTNDLGSNMTIGAVNSRGEFTGTYITAVTATSN
EIKESPLHGTENTINKRTQPTFGFTVNWKFSESTTVFTGQCFIDRNGKEVLKTMWLLRSSVNDIGD
DWKATRVGINIFTRLRTQKE
00000000000000000000000000000111111111100000000011111111000000000
00000000000000000111111111100000111111111111111111100000000000000
00000000000000000000
> P17081 RHOQ_HUMAN
MAHGPGALMLKCVVVGDGAVGKTCLLMSYANDAFPEEYVPTVFDHYAVSVTVGGKQYLLGLYDTAG
QEDYDRLRPLSYPMTDVFLICFSVVNPASFQNVKEEWVPELKEYAPNVPFLLIGTQIDLRDDPKTL
ARLNDMKEKPICVEQGQKLAKEIGACCYVECSALTQKGLKTVFDEAIIAILTPKKHTVKKRIGSRC
INCCLIT
00000000000000000000000000000000000111111100000000000000000000000
00000000000000000000000000000000000000000000000000000000000000000

000000000000000000000000000000000000000000000000000000000000000000
0000000
> P14557 BLA1_ECOLI
MRYIRLCIISLLATLPLAVHASPQPLEQIKLSESQLSGRVGMIEMDLASGRTLTAWRADERFPMMS
TFKVVLCGAVLARVDAGDEQLERKIHYRQQDLVDYSPVSEKHLADGMTVGELCAAAITMSDNSAAN
LLLATVGGPAGLTAFLRQIGDNVTRLDRWETELNEALPGDARDTTTPASMAATLRKLLTSQRLSAR
SQRQLLQWMVDDRVAGPLIRSVLPAGWFIADKTGAGERGARGIVALLGPNNKAERIVVIYLRDTPA
SMAERNQQIAGIGAALIEHWQR
000000000000000000000111111111111111111111111111111111000000000000
000000000000000001111111111111111111111111111100000000000000000000
000000000000000000000000000111111111111111111111111111111111111111
111111111111000000000000000000000000000000000000000000000000000000
0000011111111111110000
> P06749 RHOA_BOVIN
MAAIRKKLVIVGDGACGKTCLLIVFSKDQFPEVYVPTVFENYVADIEVDGKQVELALWDTAGQEDY
DRLRPLSYPDTDVILMCFSIDSPDSLENIPEKWTPEVKHFCPNVPIILVGNKKDLRNDEHTRRELA
KMKQEPVKPEEGRDMANRIGAFGYMECSAKTKDGVREVFEMATRAALQARRGKKKSGCLVL
000000000000000000000000000000011111110000000000000000000000000000
000000000000000000000000000000000000000000000000000000000000000000
0000000000000000000000000000000000000000000000000000000000000
> Q50769 MP70_MYCBO
MKVKNTIAATSFAAAGLAALAVAVSPPAAAGDLVGPGCAEYAAANPTGPASVQGMSQDPVAVAASN
NPELTTLTAALSGQLNPQVNLVDTLNSGQYTVFAPTNAAFSKLPASTIDELKTNSSLLTSILTYHV
VAGQTSPANVVGTRQTLQGASVTVTGQGNSLKVGNADVVCGGVSTANATVYMIDSVLMPPA
000000000000000000000000000000111111111111111111111111111111111111
111111100000000000000000000000000001111111111111111111000000000000
0000000011111111111111000000000000000000000000000000000000000
> P00304 MPAA3_AMBEL
GKVYLVGGPELGGWKLQSDPRAYALWSARQQFKTTDVLWFNFTTGEDSVAEVWREEAYHACDIKDP
IRLEPGGPDRFTLLTPGSHFICTKDQKFVACVPGR
111111111111111000001111111111111111111111111000001111111111111111
11111111100000000000000000000000000
> TSST_STAAU TSST_STAAU
MNKKLLMNFFIVSPLLLATTATDFTPVPLSSNQIIKTAKASTNDNIKDLLDWYSSGSDTFTNSEVL
DNSLGSMRIKNTDGSISLIIFPSPYYSPAFTKGEKVDLNTKRTKKSQHTSEGTYIHFQISGVTNTE
KLPTPIELPLKVKVHGKDSPLKYGPKFDKKQLAISTLDFEIRHQLTQIHGLYRSSDKTGGYWKITM
NDGSTYQSDLSKKFEYNTEKPPINIDEIKTIEAEIN
000000000000000000000000000000000000000011111111111111111111111000

000000000000000000000000001111111111100000000011111111111000001111
111111111110000000000000000000000000111111111110000000000000111111
111110000000000000011111111111111000
> P15252 REF_HEVBR
AEDEDNQQGQGEGLKYLGFVQDAATYAVTTFSNVYLFAKDKSGPLQPGVDIIEGPVKNVAVPLYNR
FSYIPNGALKFVDSTVVASVTIIDRSLPPIVKDASIQVVSAIRAAPEAARSLASSLPGQTKILAKV
FYGEN
111111111100000000000000000000000011111111111111111111000001111111
111111100000000000000011111111111111111110000000000000000000000000
00000
> P13472 TYB10_HORSE
MADKPDMGEIASFDKAKLKKTETQEKNTLPTKETIEQEKRSEIS
01111111111111000001111111111111111111111111
> P13829 OS25_PLAFO
MNKLYSLFLFLFIQLSIKYNNAKVTVDTVCKRGFLIQMSGHLECKCENDLVLVNEETCEEKVLKCD
EKTVNKPCGDFSKCIKIDGNPVSYACKCNLGYDMVNNVCIPNECKNVTCGNGKCILDTSNPVKTGV
CSCNIGKVPNVQDQNKCSKDGETKCSLKCLKENETCKAVDGIYKCDCKDGFIIDNESSICTAFSAY
NILNLSIMFILFSVCFFIM
000000000000000000000000000000000000000000000000000000000000000000
000000000000000000000000000000000000000000000000011111111111111111
100000000000000000000000000000000000000000000000000000000000000000
0000000000000000000
> P02971 FMC1_ECOLI
MKFKKTIGAMALTTMFVAVSASAVEKNITVTASVDPVIDLLQADGNALPSAVKLAYSPASKTFESY
RVMTQVHTNDATKKVIVKLADTPQLTDVLNSTVQMPISVSWGGQVLSTTAKEFEAAALGYSASGVN
GVSSSQELVISAAPKTAGTAPTAGNYSGVVSLVMTLGS
000000000000000000000000000000000001111111100000000000000000000000
000000000000000000000000000000000000000000000000000000000000000000
00000000000000000000000000000000000000
> P08878 PA2A_CRODU
MRALWIVAVLLVGVEGSLVEFETLMMKIAGRSGISYYSSYGCYCGAGGQGWPQDASDRCCFEHDCC
YAKLTGCDPTTDVYTYRQEDGEIVCGEDDPCGTQICECDKAAAICFRNSMDTYDYKYLQFSPENCQ
GESQPC
000000000000000000000000000000000000011111111111111111111100000000
000000000000000000000000000000000000001111111111111100000000000000
000000
> P17964 RAP2B_HUMAN
MREYKVVVLGSGGVGKSALTVQFVTGSFIEKYDPTIEDFYRKEIEVDSSPSVLEILDTAGTEQFAS

MRDLYIKNGQGFILVYSLVNQQSFQDIKPMRDQIIRVKRYERVPMILVGNKVDLEGEREVSYGEGK
ALAEEWSCPFMETSAKNKASVDELFAEIVRQMNYAAQPNGDEGCCSACVIL
000000000000000000000000000001111110000000000000000000000000000000
000000000000000000000000000000000000000000000000000000000000000000
000000000000000000000000000000000000000000000000000
> O14658 RAC3_HUMAN
MQAIKCVVVGDGAVGKTCLLISYTTNAFPGEYIPTVFDNYSANVMVDGKPVNLGLWDTAGQEDYDR
LRPLSYPQTDVFLICFSLVSPASFENVRAKWYPEVRHHCPHTPILLVGTKLDLRDDKDTIERLRDK
KLAPITYPQGLAMAREIGSVKYLECSALTQRGLKTVFDEAIRAVLCPPPVKKPGKKCTVF
000000000000000000000000000001111110000000000000000000000000000000
000000000000000000000000000000000000000000000000000000000000000000
000000000000000000000000000000000000000000000000000000000000
> P19429 TNNI3_HUMAN
ADGSSDAAREPRPAPAPIRRRSSNYRAYATEPHAKKKSKISASRKLQLKTLLLQIAKQELEREAEE
RRGEKGRALSTRCQPLELAGLGFAELQDLCRQLHARVDKVDEERYDIEAKVTKNITEIADLTQKIF
DLRGKFKRPTLRRVRISADAMMQALLGARAKESLDLRAHLKQVKKEDTEKENREVGDWRKNIDALS
GMEGRKKKFES
000000000011111111111111111111111111111111000000000000000000000000
000000000000000000001111111111111000000000011111111110000000000000
000000000000001111111111111111111000000000000000000001111111111000
00000000000
> O61379 TPM_PANST
MKLEKDNAMDRADTLEQQNKEANIRAEKAEEEVHNLQKRMQQLENDLDQVQESLLKANTQLEEKDK
ALSNAEGEVAALNRRIQLLEEDLERSEERLNTATTKLAEASQAADESERMRKVLENRSLSDEERMD
ALENQLKEARFLAEEADRKYDEVARKLAMVEADLERAEERAETGESKFVELEEELRVVGNNLKSLE
VSEEKANQREEAYKEQIKTLTNKLKAAEARAEFAERSVQKLQKEVDRLEDELVNEKEKYKSITDEL
DQTFSELSGY
000000000000000000000000000000000000000111111111111111110000000000
000000000000000000000000000000000000000000000000000000000000000000
000000000011111111100000000000000000000000000000000000000000000000
000000000000000000000000000000000000000000000000000000000000000000
0000000000
> P06484 VGLG_HHV11
MSQGAMRAVVPIIPFLLVLVGVSGVPTNVSSTTQPQLQTTGRPSHEAPNMTQTGTTDSPTAISLTT
PDHTPPMPSIGLEEEEEEEGAGDGEHLEGGDGTRDTLPQSPGPAFPLAEDVEKDKPNRPVVPSPDP
NNSPARPETSRPKTPPTIIGPLATRPTTRLTSKGRPLVPTPQHTPLFSFLTASPALDTLFVVSTVI
HTLSFLCIGAMATHLCGGWSRRGRRTHPSVRYVCLPSERG
000000000000000000000000000000000000000000000000000000000000000000

000000000000000111111111111111111100111111111111111111111111111100
000000000000000000000000000111111111111111100000000000000000000000
0000000000000000000000000000000000000000
> P06781 RHO2_YEAST
MSEKAVRRKLVIIGDGACGKTSLLYVFTLGKFPEQYHPTVFENYVTDCRVDGIKVSLTLWDTAGQE
EYERLRPFSYSKADIILIGFAVDNFESLINARTKWADEALRYCPDAPIVLVGLKKDLRQEAHFKEN
ATDEMVPIEDAKQVARAIGAKKYMECSALTGEGVDDVFEVATRTSLLMKKEPGANCCIIL
000000000000000000000000000000000111111100000000000000000000000000
000000000000000000000000000000000000000000000000000000000000000000
000000000000000000000000000000000000000000000000000000000000
> P02662 CAS1_BOVIN
MKLLILTCLVAVALARPKHPIKHQGLPQEVLNENLLRFFVAPFPEVFGKEKVNELSKDIGSESTED
QAMEDIKQMEAESISSSEEIVPNSVEQKHIQKEDVPSERYLGYLEQLLRLKKYKVPQLEIVPNSAE
ERLHSMKEGIHAQQKEPMIGVNQELAYFYPELFRQFYQLDAYPSGAWYYVPLGTQYTDAPSFSDIP
NPIGSENSEKTTMPLW
000000000000000000000000000000000111111111111000000000000000000011
111111111111111100000000000000000011111111111111111000000000000000
000000011111111110000001111111111000000000000000000000111111111111
0000011111111111
> P13811 ELBH_ECOLI
MNKVKFYVLFTALLSSLCAHGAPQSITELCSEYHNTQIYTINDKILSYTESMAGKREMVIITFKSG
ATFQVEVPGSQHIDSQKKAIERMKDTLRITYLTETKIDKLCVWNNKTPNSIAAISMEN
000000000000000000000000000000000000000000000000000000011111111111
1111000000000000000000000000000000001111111111111110000000
> P04156 PRIO_HUMAN
MANLGCWMLVLFVATWSDLGLCKKRPKPGGWNTGGSRYPGQGSPGGNRYPPQGGGGWGQPHGGGWG
QPHGGGWGQPHGGGWGQPHGGGWGQGGGTHSQWNKPSKPKTNMKHMAGAAAAGAVVGGLGGYMLGS
AMSRPIIHFGSDYEDRYYRENMHRYPNQVYYRPMDEYSNQNNFVHDCVNITIKQHTVTTTTKGENF
TETDVKMMERVVEQMCITQYERESQAYYQRGSSMVLFSSPPVILLISFLIFLIVG
000000000000000000000000000111111111111111111111111111111111111111
000000000000001111111111111111111111111111111111001111111111111111
111111111111111111111111111111111110000000000000000000011111111111
1111111111111100000000000000000000000000000000000000000
> P87020 PRA1_CANAL
MNYLLFCLFFAFSVAAPVTVTRFVNASPTGYDWRADWVKGFPIDSSCNATQYNQLSTGLQEAQLLA
EHARDHTLRFGSKSPFFRKYFGNDTASAEVVGHFENVVGADKSSILFLCDDLDDKCKNDGWAGYWR
GSNHSDQTIICDLSFVTRRYLSQLCSGGYTVSKSKTNIFWAGDLLHRFWHLKSIGQLVIEHYADTY
EEVLELAQENSTYAVRNSNSLIYYALDVYAYDVTIPGEGCNGDGTSYKKSDFSSFEDSDSGSDSGA

SSTASSSHQHTDSNPSATTDANSHCHTHADGEVHC
00000000000000000000000000000111111111000000000000000000000000000
00000111111111111111111110000000000000000000000000000001111111110
00000000000000000000000000000000000000000000000000000000000000000
00000000000000000000001111111111110000000000000000000000000000000
00000000000000000000000001111111110
> P01375 TNFA_HUMAN
MSTESMIRDVELAEEALPKKTGGPQGSRRCLFLSLFSFLIVAGATTLFCLLHFGVIGPQREEFPRD
LSLISPLAQAVRSSSRTPSDKPVAHVVANPQAEGQLQWLNRRANALLANGVELRDNQLVVPSEGLY
LIYSQVLFKGQGCPSTHVLLTHTISRIAVSYQTKVNLLSAIKSPCQRETPEGAEAKPWYEPIYLGG
VFQLEKGDRLSAEINRPDYLDFAESGQVYFGIIAL
00000000000000000000000000000000000000000000000000000000000000000
00000000000001111111111111111111111001111111111111111111111110000
00000000000000000000000000000000000000000000000011111111000000000
00000000011111111111100000000000000
> P01556 CHTB_VIBCH
MIKLKFGVFFTVLLSSAYAHGTPQNITDLCAEYHNTQIYTLNDKIFSYTESLAGKREMAIITFKNG
AIFQVEVPGSQHIDSQKKAIERMKDTLRIAYLTEAKVEKLCVWNNKTPHAIAAISMAN
00000000000000000000011111111001111110000011111100011111111000000
001111111111111111111110011111100000011111100000111111110
> P09012 SNRPA_HUMAN
AVPETRPNHTIYINNLNEKIKKDELKKSLYAIFSQFGQILDILVSRSLKMRGQAFVIFKEVSSATN
ALRSMQGFPFYDKPMRIQYAKTDSDIIAKMKGTFVERDRKREKRKPKSQETPATKKAVQGGGATPV
VGAVQGPVPGMPPMTQAPRIMHHMPGQPPYMPPPGMIPPPGLAPGQIPPGAMPPQQLMPGQMPPAQ
PLSENPPNHILFLTNLPEETNELMLSMLFNQFPGFKEVRLVPGRHDIAFVEFDNEVQAGAARDALQ
GFKITQNNAMKISFAKK
00000000000000000000000000000000011111111111111111111111000000000
00000000000000000000000000000000000000000000000000000000000000000
00000000000000000000000000000000000000000000000000000000000000000
00000000000000000000000000000000000000000000000000000000111111111
11111111111111111
> P03266 VCO7_ADE02
MSILISPSNNTGWGLRFPSKMFGGAKKRSDQHPVRVRGHYRAPWGAHKRGRTGRTTVDDAIDAVVE
EARNYTPTPPPVSTVDAAIQTVVRGARRYAKMKRRRRRVARRHRRRPGTAAQRAAAALLNRARRTG
RRAAMRAARRLAAGIVTVPPRSRRRAAAAAAAAISAMTQGRRGNVYWVRDSVSGLRVPVRTRPPRN
00000000000000000000000000000000000000000000000000000000000000011
11111111111100000000000000000000000000000000000000000000000000000
00000000000000000000000000000000000000000000000000000000000000000

> Q08137 OSPC2_BORBU
MKKNTLSAILMTLFLFISCNNSGKGGDSASTNPADESAKGPNLTEISKKITDSNAFVLAVKEVETL
VLSIDELAKKAIGQKIDNNNGLAALNNQNGSLLAGAYAISTLITEKLSKLKNLEELKTEIAKAKKC
SEEFTNKLKSGHADLGKQDATDDHAKAAILKTHATTDKGAKEFKDLFESVEGLLKAAQVALTNSVK
ELTSPVVAESPKKP
000000000000000000000000000000000000000000000000000000000000000000
000000000000000000000000000000000000000000000000000000000000000000
000000000000000000000000000000000000000000000000000000000000000000
00001111111111
> Q39967 ALL5_HEVBR
ASVEVESAATALPKNETPEVTKAEETKTEEPAAPPASEQETADATPEKEEPTAAPAEPEAPAPETE
KAEEVEKIEKTEEPAPEADQTTPEEKPAEPEPVAEEEPKHETKETETEAPAAPAEGEKPAEEEKPI
TEAAETATTEVPVEKTEE
000000000000011111111011111111111101111111111111111111110000000000
000000111111111100001111111111000000000000000000000000000000000011
111111111100000000
> O82803 SRPP_HEVBR
MAEEVEEERLKYLDFVRAAGVYAVDSFSTLYLYAKDISGPLKPGVDTIENVVKTVVTPVYYIPLEA
VKFVDKTVDVSVTSLDGVVPPVIKQVSAQTYSVAQDAPRIVLDVASSVFNTGVQEGAKALYANLEP
KAEQYAVITWRALNKLPLVPQVANVVVPTAVYFSEKYNDVVRGTTEQGYRVSSYLPLLPTEKITKV
FGDEAS
011111111111111110000000000011111111110011111111110000000001111111
111100000000000000111111111100000000111111111100000111111111100000
000000000000000000000000001111111111000000000000000000000000000000
000000
> Q9ZMZ4 UREA_HELPJ
MKLTPKELDKLMLHYAGELARKRKEKGIKLNYVEAVALISAHIMEEARAGKKTAAELMQEGRTLLK
PDDVMDGVASMIHEVGIEAMFPDGTKLVTVHTPIEANGKLVPGELFLKNEDITINEGKKAVSVKVK
NVGDRPVQIGSHFHFFEVNRCLDFDREKTFGKRLDIASGTAVRFEPGEEKSVELIDIGGNRRIFGF
NALVDRQADNESKKIALHRAKERGFHGAKSDDNYVKTIKE
111111111110000000000000000000000000000000000000000000000000000000
000000000000000000000111111111111111000000000000000000011111111111
111000000000000000000000000000000000000000000000000000111111111100
0000000000000000000000000000000000000000
> P03126 VE6_HPV16
MHQKRTAMFQDPQERPRKLPQLCTELQTTIHDIILECVYCKQQLLRREVYDFAFRDLCIVYRDGNP
YAVCDKCLKFYSKISEYRHYCYSLYGTTLEQQYNKPLCDLLIRCINCQKPLCPEEKQRHLDKKQRF
HNIRGRWTGRCMSCCRSSRTRRETQL

000111111111111111000000000000000000000000000000000000000000000000
000000000000000000000000000000000000000000000000000000000000000000
00000000000000000000000000
> P03100 VL1_HPV6A
MWRPSDSTVYVPPPNPVSKVVATDAYVTRTNIFYHASSSRLLAVGHPYFSIKRANKTVVPKVSGYQ
YRVFKVVLPDPNKFALPDSSLFDPTTQRLVWACTGLEVGRGQPLGVGVSGHPFLNKYDDVENSGSG
GNPGQDNRVNVGMDYKQTQLCMVGCAPPLGEHWGKGKQCTNTPVQAGDCPPLELITSVIQDGDMVD
TGFGAMNFADLQTNKSDVPIDICGTTCKYPDYLQMAADPYGDRLFFFLRKEQMFARHFFNRAGEVG
EPVPDTLIIKGSGNRTSVGSSIYVNTPSGSLVSSEAQLFNKPYWLQKAQGHNNGICWGNQLFVTVV
DTTRSTNMTLCASVTTSSTYTNSDYKEYMRHVEEYDLQFIFQLCSITLSAEVMAYIHTMNPSVLED
WNFGLSPPPNGTLEDTYRYVQSQAITCQKPTPEKEKPDPYKNLSFWEVNLKEKFSSELDQYPLGRK
FLLQSGYRGRSSIRTGVKRPAVSKASAAPKRKRAKTKR
000000000000000000000000000000000000000000000000000000000000000000
000000000000000000000000000000000000000000000000000000000000000000
000000000000000000000000000000000000000000000000000000000000111111
111111111111111111111100000000000000000000000000000000000000000000
000000000000000000000000000000000000000000000000000000000000000000
000000000000000000000000000000000000000000000000000000000000000000
000000000000000000001111111111111111111100000000000000000000000000
00000000000000000000000000000000000000
> P03412 YPX1_BLVJ
ASVVGWGPHSLHACPALVLSNDVTIDAWCPLCGPHERLQFERIDTTLTCETHRINWTADGRPCGLN
GTLFPRLHVSETRPQGPRRLWINCPLPAVRAQPGPVSLSPFERSPFQPYQCQLPSASSDGCPIIGH
GLLPWNNLVTHPVLGKVLILNQMANFSLLPSFDTLLVDPLRLSVFAPDTRGAIRYLSTLLTLCPAT
CILPLGEPFSPNVPICRFPRDSNEPPLSEFELPPIQTPGLSWSVPAIDLFLTGPPSPCDRLHVWSS
PQALQRFLHDPTLTWSELVASRKIRLDSPLKLQLLENEWLSRLF
000000000000000000000000000000000000000000000000000000000000000000
000000000000000000000000000000000000000000000000000000000000000111
111111111111111110000000000000000000000000000000000000000000000000
000000000000000000000000000000000000000000000000000000000000011111
11111111111111000000000000000000000000000000
> P02893 CSP_PLAFA
MMRKLAILSVSSFLFVEALFQEYQCYGSSSNTRVLNELNYDNAGTNLYNELEMNYYGKQENWYSLK
KNSRSLGENDDGNNNNGDNGREGKDEDKRDGNNEDNEKLRKPKHKKLKQPGDGNPDPNANPNVDPN
ANPNVDPNANPNVDPNANPNANPNANPNANPNANPNANPNANPNANPNANPNANPNANPNANPNAN
PNANPNANPNVDPNANPNANPNANPNANPNANPNANPNANPNANPNANPNANPNANPNANPNANPN
ANPNANPNANPNANPNANPNANPNKNNQGNGQGHNMPNDPNRNVDENANANNAVKNNNNEEPSDKH
IEQYLKKIKNSISTEWSPCSVTCGNGIQVRIKPGSANKPKDELDYENDIEKKICKMEKCSSVFNVV

NSSIGLIMVLSFLFLN
000000000000000000000000000000000000000000000000000000000000000000
000000000000000000000111111111111111111111111111111111111111111111
111111111111110000000000000000000000000000000000000000000000000000
000000000000000000000000000000000000000000000000000000000000000000
000000000000000000001111111111111111111111111111111111111111111000
000000000000000000000000000000000000000000000000000000000000000000
0000000000000000
> P14917 URE1_HELPJ
MKKISRKEYVSMYGPTTGDKVRLGDTDLIAEVEHDYTIYGEELKFGGGKTLREGMSQSNNPSKEEL
DLIITNALIVDYTGIYKADIGIKDGKIAGIGKGGNKDMQDGVKNNLSVGPATEALAGEGLIVTAGG
IDTHIHFISPQQIPTAFASGVTTMIGGGTGPADGTNATTITPGRRNLKWMLRAAEEYSMNLGFLAK
GNASNDASLADQIEAGAIGFKIHEDWGTTPSAINHALDVADKYDVQVAIHTDTLNEAGCVEDTMAA
IAGRTMHTFHTEGAGGGHAPDIIKVAGEHNILPASTNPTIPFTVNTEAEHMDMLMVCHHLDKSIKE
DVQFADSRIRPQTIAAEDTLHDMGIFSITSSDSQAMGRVGEVITRTWQTADKNKKEFGRLKEEKGD
NDNFRIKRYLSKYTINPAIAHGISEYVGSVEVGKVADLVLWSPAFFGVKPNMIIKGGFIALSQMGD
ANASIPTPQPVYYREMFAHHGKAKYDANITFVSQAAYDKGIKEELGLERQVLPVKNCRNITKKDMQ
FNDTTAHIEVNPETYHVFVDGKEVTSKPANKVSLAQLFSIF
000000000000000000000000000000000000000000000000000000000000000000
000000000000000000000000000000000000000000000000000000000000000000
011111111111111111111111111111111111000000000000000000000000000000
000000000000000000000111111111110000000000000000000000000000000000
000000000000000000000000000000000000000000000000000000001111111111
111111111000000000000000000000000000000000000000000000000011111111
111111101111111111000000000111111111111000011111111111111111111111
111111111100000000000000000000000000000000000000000000000000000000
00000000000000000000000000000000000000000
> P03135 COA3_AAV2
MATGSGAPMADNNEGADGVGNSSGNWHCDSTWMGDRVITTSTRTWALPTYNNHLYKQISSQSGASN
DNHYFGYSTPWGYFDFNRFHCHFSPRDWQRLINNNWGFRPKRLNFKLFNIQVKEVTQNDGTTTIAN
NLTSTVQVFTDSEYQLPYVLGSAHQGCLPPFPADVFMVPQYGYLTLNNGSQAVGRSSFYCLEYFPS
QMLRTGNNFTFSYTFEDVPFHSSYAHSQSLDRLMNPLIDQYLYYLSRTNTPSGTTTQSRLQFSQAG
ASDIRDQSRNWLPGPCYRQQRVSKTSADNNNSEYSWTGATKYHLNGRDSLVNPAMASHKDDEEKFF
PQSGVLIFGKQGSEKTNVNIEKVMITDEEEIGTTNPVATEQYGSVSTNLQRGNRQAATADVNTQGV
LPGMVWQDRDVYLQGPIWAKIPHTDGHFHPSPLMGGFGLKHPPPQILIKNTPVPANPSTTFSAAKF
ASFITQYSTGHGQRGDRVGAAEGKQQTLESRNSVHFQLQQVC
000000000000000000000000000000000000001111111111111111111100000000
000000000000000000000000000000000000000000000000000000000000000000

000000000000000000000000000000000000000000000000000000000000000000
000000000000000000000000000000000000000000000000000000000000000011
111111111111111111111111110000000000000000000000000000000000000000
000000000000000000000000000000000000000000000000000000000000000000
000000000000000000000000000000000000000000000000000000000000000000
000000000000000000000000000000000000000000

> P02686 MBP_HUMAN

MGNHAGKRELNAEKASTNSETNRGESEKKRNLGELSRTTSEDNEVFGEADANQNNGTSSQDTAVTD
SKRTADPKNAWQDAHPADPGSRPHLIRLFSRDAPGREDNTFKDRPSESDELQTIQEDSAATSESLD
VMASQKRPSQRHGSKYLATASTMDHARHGFLPRHRDTGILDSIGRFFGGDRGAPKRGSGKDSHHPA
RTAHYGSLPQKSHGRTQDENPVVHFFKNIVTPRTPPPSQGKGRGLSLSRFSWGAEGQRPGFGYGGR
ASDYKSAHKGFKGVDAQGTLSKIFKLGGRDSRSGSPMARR
000000000000000000000000000000000000000000000000000000000000000000
000000000000000000000000000000000000000000000000000000000000000000
000000000000000000000000000000000000000000000000000000000000000000
000000000000000000000000000000000000000000000000000000000000000000
0111111111111111111110000000000000000000

> P81294 MPAJ1_JUNAS

MASPCLIAVLVFLCAIVSCYSDNPIDSCWRGDSNWDQNRMKLADCAVGFGSSTMGGKGGDFYTVTS
TDDNPVNPTPGTLRYGATREKALWIIFSQNMNIKLKMPLYVAGHKTIDGRGADVHLGNGGPCLFMR
KVSHVILHSLHIHGCNTSVLGDVLVSESIGVEPVHAQDGDAITMRNVTNAWIDHNSLSDCSDGLID
VTLGSTGITISNNHFFNHHKVMLLGHDDTYDDDKSMKVTVAFNQFGPNAGQRMPRARYGLVHVANN
NYDPWNIYAIGGSSNPTILSEGNSFTAPSESYKKEVTKRIGCESPSACANWVWRSTRDAFINGAYF
VSSGKTEETNIYNSNEAFKVENGNAAPQLTKNAGVVT
000000000000000000000000000000000000000000000000000000000000000000
000000000000000000000000011111111111110000000000000000000000000000
000000000000000000000000000000000000000000000000000000000000000000
000000000000000000000000000000000000111111111111111111000000000000
000000000000000000000000000000000000000000000000111111111111111110
0000000000000000000000000000000000000

> P02710 ACHA_TORCA

MILCSYWHVGLVLLLFSCCGLVLGSEHETRLVANLLENYNKVIRPVEHHTHFVDITVGLQLIQLIS
VDEVNQIVETNVRLRQQWIDVRLRWNPADYGGIKKIRLPSDDVWLPDLVLYNNADGDFAIVHMTKL
LLDYTGKIMWTPPAIFKSYCEIIVTHFPFDQQNCTMKLGIWTYDGTKVSISPESDRPDLSTFMESG
EWVMKDYRGWKHWVYYTCCPDTPYLDITYHFIMQRIPLYFVVNVIIPCLLFSFLTGLVFYLPTDSG
EKMTLSISVLLSLTVFLLVIVELIPSTSSAVPLIGKYMLFTMIFVISSIIITVVVINTHHRSPSTH
TMPQWVRKIFIDTIPNVMFFSTMKRASKEKQENKIFADDIDISDISGKQVTGEVIFQTPLIKNPDV
KSAIEGVKYIAEHMKSDEESSNAAEEWKYVAMVIDHILLCVFMLICIIGTVSVFAGRLIELSQEG

000000000000000000000000111111111111111100000011111111111111111111
111111111111111111111111111111111111111100000000000000000111111111
111111100000011111111111111111111111111111110000000000000000000011111
111111111111111111111111111111111111000000000000000000000000000000
000000000000000000000000000000000000000000000000000000000000000000
000000000000000000000000000000000000000000000000000000000000000000
00000000000000000000000000000000000000000000000000000000000000000
> P00178 CP2B4_RABIT
MEFSLLLLLAFLAGLLLLLFRGHPKAHGRLPPGPSPLPVLGNLLQMDRKGLLRSFLRLREKYGDVF
TVYLGSRPVVVLCGTDAIREALVDQAEAFSGRGKIAVVDPIFQGYGVIFANGERWRALRRFSLATM
RDFGMGKRSVEERIQEEARCLVEELRKSKGALLDNTLLFHSITSNIICSIVFGKRFDYKDPVFLRL
LDLFFQSFSLISSFSSQVFELFPGFLKHFPGTHRQIYRNLQEINTFIGQSVEKHRATLDPSNPRDF
IDVYLLRMEKDKSDPSSEFHHQNLILTVLSLFFAGTETTSTTLRYGFLLMLKYPHVTERVQKEIEQ
VIGSHRPPALDDRAKMPYTDAVIHEIQRLGDLIPFGVPHTVTKDTQFRGYVIPKNTEVFPVLSSAL
HDPRYFETPNTFNPGHFLDANGALKRNEGFMPFSLGKRICLGEGIARTELFLFFTTILQNFSIASP
VPPEDIDLTPRESGVGNVPPSYQIRFLAR
111111001111111101111111100000000000001111111111000000000000000000
111111000000111111110000011111111000001111111111111111111111111000
000000001111111111111111000000000000000000001111110111111111110000
000000000000000000011111100000011111100000000000000000000000000000
000000000000000000000000000000000000000000000000001111111110000001
111111111110111111000111111110001111111100011111100000001111111111
111111111111111111111101111111111100000000000000000000000000111111
00011111111111110111111110000
> P37837 TALDO_HUMAN
MSSSPVKRQRMESALDQLKQFTTVVADTGDFHAIDEYKPQDATTNPSLILAAAQMPAYQELVEEAI
AYGRKLGGSQEDQIKNAIDKLFVLFGAEILKKIPGRVSTEVDARLSFDKDAMVARARRLIELYKEA
GISKDRILIKLSSTWEGIQAGKELEEQHGIHCNMTLLFSFAQAVACAEAGVTLISPFVGRILDWHV
ANTDKKSYEPLEDPGVKSVTKIYNYYKKFSYKTIVMGASFRNTGEIKALAGCDFLTISPKLLGELL
QDNAKLVPVLSAKAAQASDLEKIHLDEKSFRWLHNEDQMAVEKLSDGIRKFAADAVKLERMLTERM
FNAENGK
000000000011111111111111111111111111111111111000000000000000000000
000011111111111111111111111110000011111111111111100000000000000000
000000000000000000000000000000000000000000000000000000000000000000
001111111111111110000000000000001111111111111110000000000000001111
111111111111111111111000000000000000000000000011111111111111100000
0000000
> P17101 VMSA_HBVW9

GGWSSKPRKGMGTNLSVPNPLGFFPDHQLDPVFGANSNNPDWDFNPIKDHWPAANQVGVGAFGPGF
TPPHGGVLGWSPQAQGMLTPVSTIPPPASANRQSGRQPTPISPPLRDSHPQAMQWNSTAFHQALQD
PRVRGLYFPAGGSSSGTVNPAPNIASHISSISARTGDPVTNMENITSGFLGPLPVLQAGFFLLTRI
LTIPQSLDSWWTSLNFLGGSPVCLGQNSRSPTSNHSPTSCPPICPGYRWMCLRRFIIFLFILLLCL
IFLLVLLDYQGMLPVCPLILGSTTTSTGPCKTCTTPAQGNSMFPSCCCTKPTDGNCTCIPIPSSWA
FAKYLWEWASVRFSWLSLLVPFVQWFVGLSPTVWLSAIWMMWYWGPSLYSIVSSFIPLLPIFFCLW
VYI
000000000000000000000000000000000000000000000000000000000000000000
000000000000000000000000000000000000000000000000000000000000000000
000000000000000000000000000000000000000000000000000000000000000000
000000000000000000000000000000000000000000000000000000000000000000
000000000000000000000000000000000000000000000000000000000000000000
000000000000000001111111111111110000000000000000000000000000000000
000
> Q40240 MPA5A_LOLPR
MAVQKYTVALFLRRGPRGGPGRSYAADAGYTPAAAATPATPAATPAGGWREGDDRRAEAAGGRQRL
ASRQPWPPLPTPLRRTSSRSSRPPSPSPPRASSPTSAAKAPGLIPKLDTAYDVAYKAAEAHPRGQV
RRLRHCPHRSLRVIAGALEVHAVKPATEEVLAAKIPTGELQIVDKIDAAFKIAATAANAAPTNDKF
TVFESAFNKALNECTGGAMRPTSSSPPSRPRSSRPTPPPSPAAPEVKYAVFEAALTKAITAMTQAQ
KAGKPAAAAATAAATVATAAATAAAVLPPPLLVVQSLISLLIYY
000000000000000000000000000000000000000000000000000000000000000000
000000000000000000000000000000000000000000000000000000000000000000
000000000000011111111111110000000000011111111111100001111111111110
000111111111111000000000000000000000000000000000000000000000111111
11111100000000000000000000000000000000000000
> P04405 GLYG2_SOYBN
MAKLVLSLCFLLFSGCFALREQAQQNECQIQKLNALKPDNRIESEGGFIETWNPNNKPFQCAGVAL
SRCTLNRNALRRPSYTNGPQEIYIQQGNGIFGMIFPGCPSTYQEPQESQQRGRSQRPQDRHQKVHR
FREGDLIAVPTGVAWWMYNNEDTPVVAVSIIDTNSLENQLDQMPRRFYLAGNQEQEFLKYQQQQQG
GSQSQKGKQQEEENEGSNILSGFAPEFLKEAFGVNMQIVRNLQGENEEEDSGAIVTVKGGLRVTAP
AMRKPQQEEDDDDEEEQPQCVETDKGCQRQSKRSRNGIDETICTMRLRQNIGQNSSPDIYNPQAGS
ITTATSLDFPALWLLKLSAQYGSLRKNAMFVPHYTLNANSIIYALNGRALVQVVNCNGERVFDGEL
QEGGVLIVPQNFAVAAKSQSDNFEYVSFKTNDRPSIGNLAGANSLLNALPEEVIQHTFNLKSQQAR
QVKNNNPFSFLVPPQESQRRAVA
001111111111111110000000000000000000000000000000000000000000000000
000000000000000011111111111111111111111000000000000000000000000000
000000000000000000000000000000000000000000000111111111111111111111
111111000000000000000000000000000000000000000000000000001111111111

111110000000000000000000000000000000000000000000000000000000000000
000000111111111111111111111111111111111111111111111000000000000000
000000000000000000000000000000000000000000000000000000111111111111
11100000000000000000000
> Q02168 VP5_AHSV4
MGKFTSFLKRAGNATKRALTSDSAKKMYKLAGKTLQRVVESEVGSAAIDGVMQGAIQSIIQGENLG
DSIKQAVILNVAGTLESAPDPLSPGEQLLYNKVSEIEKMEKEDRVIETHNAKIEEKFGKDLLAIRK
IVKGEVDAEKLEGNEIKYVEKALSGLLEIGKDQSERITKLYRALQTEEDLRTRDETRMINEYREKF
DALKEAIEIEQQATHDEAIQEMLDLSAEVIETASEEVPIFGAGAANVIATTRAIQGGLKLKEIVDK
LTGIDLSHLKVADIHPHIIEKAMLRDTVTDKDLAMAIKSKVDVIDEMNVETQHVIDAVLPIVKQEY
EKHDNKYHVRIPGALKIHSEHTPKIHIYTTPWDSDSVFMCRAIAPHHQQRSFFIGFDLEIEYVHFE
DTSVEGHILHGGAITVEGRGFRQAYTEFMNAAWGMPTTPELHKRKLQRSMGTHPIYMGSMDYAISY
EQLVSNAMRLVYDSELQMHCLRGPLKFQRRTLMNALLYGVKIA
000000000000000000000000001111111111111111110000111111111111111000
000111111111111111111111111111111000000000000000000000000000000000
000000000000000000000000000000000000000001111111111111111100111111
111111111100111111111111111111111100000000000000000000000000000000
000000000000000000000000000000000000000000000000001111111111111000
000000000000000000000000000000000000000000000000000000000000000000
000000000000000000000000000000000000000000000000000000000000000000
0000000000000000000000000000000000000000000
> P23732 OM1A_CHLTR
MKKLLKSVLVFAALSSASSLQALPVGNPAEPSLMIDGILWEGFGGDPCDPCTTWCDAISMRMGYYG
DFVFDRVLKTDVNKEFQMGAAPTTRDVAGLEKDPVVNVARPNPAYGKHMQDAEMFTNAAYMALNIW
DRFDVFCTLGATTGYLKGNSASFNLVGLFGTKTQSSGFDTANIVPNTALNQAVVELYTDTTFAWSV
GARAALWECGCATLGASFQYAQSKPKVEELNVLCNASEFTINKPKGYVGAEFPLDITAGTEAATGT
KDASIDYHEWQASLALSYRLNMFTPYIGVKWSRVSFDADTIRIAQPKLAKPVLDTTTLNPTIAGKG
TVVSSAENELADTMQIVSLQLNKMKSRKSCGIAVGTTIVDADKYAVTVETRLIDERAAHVNAQFRF
000000000000000000000000000000000000000000000000000000000000000000
000000000000000000000000011111111110000000000000000000000000000000
000000000000000000000000000000000000000000000000000000000000000000
000000000000000000000000000000000000000000000000000000000000000000
000000000000000000000000000000000000000000000001111111111111111111
111111111110000000000000000000000000000000000000000000000000000000
> P08780 OM1C_CHLTR
MKKLLKSVLVFAALSSASSLQALPVGNPAEPSLMIDGILWEGFGGDPCDPCTTWCDAISMRVGYYG
DFVFDRVLKTDVNKEFQMGAAPTTSDVAGLQNDPTINVARPNPAYGKHMQDAEMFTNAAYMALNIW
DRFDVFCTLGATTGYLKGNSASFNLVGLFGTKTQSSSFNTAKLIPNTALNEAVVELYINTTFAWSV

GARAALWECGCATLGASFQYAQSKPKVEELNVLCNASEFTINKPKGYVGAEFPLNITAGTEAATGT
KDASIDYHEWQASLALSYRLNMFTPYIGVKWSRVSFDADTIRIAQPKLAEAILDVTTLNRTTAGKG
SVVSAGTDNELADTMQIVSLQLNKMKSRKSCGIAVGTTIVDADKYAVTVEARLIDERAAHVNAQFR
F
000000000000000000000000000000000000000000000000000000000000000000
000000000000000000000000000111111110000000000000000000000000000000
000000000000000000000000000000111111111111100000000000000000000000
000000000000000000000000000000000000000000000000000000000000000000
000000000000000000000000000000000000000000000000000000000000000000
000000000000000000000000000000000000000000000000000000000000000000
0
> P21793 PGS2_BOVIN
MKATIIFLLVAQVSWAGPFQQKGLFDFMLEDEASGIGPEEHFPEVPEIEPMGPVCPFRCQCHLRVV
QCSDLGLEKVPKDLPPDTALLDLQNNKITEIKDGDFKNLKNLHTLILINNKISKISPGAFAPLVKL
ERLYLSKNQLKELPEKMPKTLQELRVHENEITKVRKSVFNGLNQMIVVELGTNPLKSSGIENGAFQ
GMKKLSYIRIADTNITTIPQGLPPSLTELHLDGNKITKVDAASLKGLNNLAKLGLSFNSISAVDNG
SLANTPHLRELHLNNNKLVKVPGGLADHKYIQVVYLHNNNISAIGSNDFCPPGYNTKKASYSGVSL
FSNPVQYWEIQPSTFRCVYVRAAVQLGNYK
000000000000000000000000000000001111111111100000000000000000000000
000000000000000000000000000000000000000000000000000000000000000000
000000000000000001111111111100000000000111111111111100000000000000
000000000000000000000000000000000000000000000000000000000000000000
001111111111111100000000000000000000000000000000000000000000000000
000000000000000000000000000000
> P15310 PHLC1_CLOPE
MKRKICKALICATLATSLWAGASTKVYAWDGKIDGTGTHAMIVTQGVSILENDLSKNEPESVRKNL
EILKENMHELQLGSTYPDYDKNAYDLYQDHFWDPDTDNNFSKDNSWYLAYSIPDTGESQIRKFSAL
ARYEWQRGNYKQATFYLGEAMHYFGDIDTPYHPANVTAVDSAGHVKFETFAEERKEQYKINTAGCK
TNEDFYADILKNKDFNAWSKEYARGFAKTGKSIYYSHASMSHSWDDWDYAAKVTLANSQKGTAGYI
YRFLHDVSEGNDPSVGKNVKELVAYISTSGEKDAGTDDYMYFGIKTKDGKTQEWEMDNPGNDFMTG
SKDTYTFKLKDENLKIDDIQNMWIRKRKYTAFPDAYKPENIKIIANGKVVVDKDINEWISGNSTYN
IK
000000000000000000000000000011111110000000000000000111111100000000
000000111111000000000000000001111110000000001111111000000000000000
000000000000000000001111111111100000000000000000000000000000000000
000001111111000000000111111110000000000000000000000000000000000111
111110000000000000000000000000000000000000000000000000000011111111
000000000000000000000000000000000000000000000000000000000000000000

00

> P04013 VL2_HPV11

MKPRARRRKRASATQLYQTCKATGTCPPDVIPKVEHTTIADQILKWGSLGVFFGGLGIGTGAGSGG
RAGYIPLGSSPKPAITGGPAARPPVLVEPVAPSDPSIVSLIEESAIINAGAPEVVPPTQGGFTITS
SESTTPAILDVSVTNHTTTSVFQNPLFTEPSVIQPQPPVEASGHILISAPTITSQHVEDIPLDTFV
VSSSDSGPTSSTPLPRAFPRPRVGLYSRALQQVQVTDPAFLSTPQRLVTYDNPVYEGEDVSLQFTH
ESIHNAPDEAFMDIIRLHRPAITSRRGLVRFSRIGQRGSMYTRSGQHIGARIHYFQDISPVTQAAE
EIELHPLVAAENDTFDIYAEPFDPIPDPVQHSVTQSYLTSTPNTLSQSWGNTTVPLSIPSDWFVQS
GPDITFPTASMGTPFSPVTPALPTGPVFITGSDFYLHPTWYFARRRRKRIPLFFTDVAA
000000000000000000000000000000000000000000000000111111111111111111
111111111111111111000000000000000000111111111111111111111111100000
000000000000011111111111111111000000000000000000111111111111111111
110000000000000000000000000000000000000000000000000000000000000000
000000000000000000000000000000000000000000000000000000000000000000
000000000000000000000000111111111111100000000000000000000000000000
00000000000000000000000000000000000000000000000000000000000

> P01012 OVAL_CHICK

GSIGAASMEFCFDVFKELKVHHANENIFYCPIAIMSALAMVYLGAKDSTRTQINKVVRFDKLPGFG
DSIEAQCGTSVNVHSSLRDILNQITKPNDVYSFSLASRLYAEERYPILPEYLQCVKELYRGGLEPI
NFQTAADQARELINSWVESQTNGIIRNVLQPSSVDSQTAMVLVNAIVFKGLWEKAFKDEDTQAMPF
RVTEQESKPVQMMYQIGLFRVASMASEKMKILELPFASGTMSMLVLLPDEVSGLEQLESIINFEKL
TEWTSSNVMEERKIKVYLPRMKMEEKYNLTSVLMAMGITDVFSSSANLSGISSAESLKISQAVHAA
HAEINEAGREVVGSAEAGVDAASVSEEFRADHPFLFCIKHIATNAVLFFGRCVSP
000000000000000000000000000000000000011111111111100000000000000000
000000000000000000000000111111111111111100000000000000000000000000
000000000000000000000000000000000000000000000000000000001111111111
111100000000000000000000000000000000001111111111111111111111111100
000000000000000000000000000000000000000000000000000000000000000000
0000000000000000000000000000000000000000000000000000000

> P11089 FLA1_BORBU

MIINHNTSAINASRNNGINAANLSKTQEKLSSGYRINRASDDAAGMGVSGKINAQIRGLSQASRNT
SKAINFIQTTEGNLNEVEKVLVRMKELAVQSGNGTYSDADRGSIQIEIEQLTDEINRIADQAQYNQ
MHMLSNKSASQNVRTAEELGMQPAKINTPASLSGSQASWTLRVHVGANQDEAIAVNIYAANVANLF
SGEGAQTAQAAPVQEGVQQEGAQQPAPATAPSQGGVNSPVNVTTTVDANTSLAKIENAIRMISDQR
ANLGAFQNRLESIKDSTEYAIENLKASYAQIKDATMTDEVVAATTNSILTQSAMAMIAQANQVPQY
VLSLLR
000000000000000000000000000000000000000000000000000000000000000000
000000000000000000000000000000000000000000000000000000000000000000

000000000000000000000000000000000000000000000000000000000000000000
000000000000001111111111110000000000000000000000000000000000000000
000000000000000000000000000000000000000000000000000000000000000000
000000
> P27455 OMP1_CHLPN
MKKLLKSALLSAAFAGSVGSLQALPVGNPSDPSLLIDGTIWEGAAGDPCDPCATWCDAISLRAGFY
GDYVFDRILKVDAPKTFSMGAKPTGSAAANYTTAVDRPNPAYNKHLHDAEWFTNAGFIALNIWDRF
DVFCTLGASNGYIRGNSTAFNLVGLFGVKGTTVNANELPNVSLSNGVVELYTDTSFSWSVGARGAL
WECGCATLGAEFQYAQSKPKVEELNVICNVSQFSVNKPKGYKGVAFPLPTDAGVATATGTKSATIN
YHEWQVGASLSYRLNSLVPYIGVQWSRATFDADNIRIAQPKLPTAVLNLTAWNPSLLGNATALSTT
DSFSDFMQIVSCQINKFKSRKACGVTVGATLVDADKWSLTAEARLINERAAHVSGQFRF
000000000000000000000000000000000000000000000000000000000000000000
000000000000111111111111100000000000000000000000000000000000000000
000000000000000000000000001111111111111111111111000000000000000000
000000000000000000000000000000000000000000111111111111111111000000
000000000000000000000000000000000000000000000111111111111111111111
10000000000000000000000000000000000000000000000000000000000
> P13794 PORF_PSEAE
MKLKNTLGVVIGSLVAASAMNAFAQGQNSVEIEAFGKRYFTDSVRNMKNADLYGGSIGYFLTDDVE
LALSYGEYHDVRGTYETGNKKVHGNLTSLDAIYHFGTPGVGLRPYVSAGLAHQNITNINSDSQGRQ
QMTMANIGAGLKYYFTENFFAKASLDGQYGLEKRDNGHQGEWMAGLGVGFNFGGSKAAPAPEPVAD
VCSDSDNDGVCDNVDKCPDTPANVTVDANGCPAVAEVVRVQLDVKFDFDKSKVKENSYADIKNLAD
FMKQYPSTSTTVEGHTDSVGTDAYNQKLSERRANAVRDVLVNEYGVEGGRVNAVGYGESRPVADNA
TAEGRAINRRVEAEVEAEAK
000000000000000000000000000000000000000000000000000000000000000000
000000000000000000000000000000000000000000000000000000000000000000
000000000000000000000000000000000000000000000000000000000000000000
000000000000000000000000000000000000000000000000000000000000000000
000000000000000000001111111111111100000000000000000000000000000011
11111111111100000000
> P16155 OM1F_CHLTR
MKKLLKSVLVFAALSSASSLQALPVGNPAEPSLMIDGILWEGFGGDPCDPCTTWCDAISMRMGYYG
DFVFDRVLKTDVNKEFEMGEALAGASGNTTSTLSKLVERTNPAYGKHMQDAEMFTNAACMTLNIWD
RFDVFCTLGATSGYLKGNSASFNLVGLFGDGVNATKPAADSIPNVQLNQSVVELYTDTTFAWSVGA
RAALWECGCATLGASFQYAQSKPKIEELNVLCNAAEFTINKPKGYVGKEFPLDLTAGTDAATGTKD
ASIDYHEWQASLSLSYRLNMFTPYIGVKWSRASFDSDTIRIAQPRLVTPVVDITTLNPTIAGCGSV
AGANTEGQISDTMQIVSLQLNKMKSRKSCGIAVGTTIVDADKYAVTVETRLIDERAAHVNAQFRF
000000000000000000000000000000000000000000000000000000000000000000

00000000000000000000000000000000000000000000000000000000000000000
00000000000000000000000000001111111111000000000000000000000000000
00000000000000000000000000000000000000000000001111111111000000000
00000000000000000000000000000000000000000000000000000000000011111
1111111110000000000000000000000000000000000000000000000000000000
> P03120 VE2_HPV16
METLCQRLNVCQDKILTHYENDSTDLRDHIDYWKHMRLECAIYYKAREMGFKHINHQVVPTLAVSK
NKALQAIELQLTLETIYNSQYSNEKWTLQDVSLEVYLTAPTGCIKKHGYTVEVQFDGDICNTMHYT
NWTHIYICEEASVTVVEGQVDYYGLYYVHEGIRTYFVQFKDDAEKYSKNKVWEVHAGGQVILCPTS
VFSSNEVSSPEIIRQHLANHPAATHTKAVALGTEETQTTIQRPRSEPDTGNPCHTTKLLHRDSVDS
APILTAFNSSHKGRINCNSNTTPIVHLKGDANTLKCLRYRFKKHCTLYTAVSSTWHWTGHNVKHKS
AIVTLTYDSEWQRDQFLSQVKIPKTITVSTGFMSI
00000000000000000000000000000000000000000000000000000000000000000
00000000000000000000000000000000000000000000000000000111111111111
11111111000000000000000000000000000000000000000111111111111111111
11000000000000000000000000000000000000000111111111111111111110000
00000000000000000000000000000000000000000000000000000000000000000
00000000000000000000000000000000000
> P17388 VL1_HPV31
MSLWRPSEATVYLPPVPVSKVVSTDEYVTRTNIYYHAGSARLLTVGHPYYSIPKSDNPKKIVVPKV
SGLQYRVFRVRLPDPNKFGFPDTSFYNPETQRLVWACVGLEVGRGQPLGVGISGHPLLNKFDDTEN
SNRYAGGPGTDNRECISMDYKQTQLCLLGCKPPIGEHWGKGSPCSNNAITPGDCPPLELKNSVIQD
GDMVDTGFGAMDFTALQDTKSNVPLDICNSICKYPDYLKMVAEPYGDTLFFYLRREQMFVRHFFNR
SGTVGESVPTDLYIKGSGSTATLANSTYFPTPSGSMVTSDAQIFNKPYWMQRAQGHNNGICWGNQL
FVTVVDTTRSTNMSVCAAIANSDTTFKSSNFKEYLRHGEEFDLQFIFQLCKITLSADIMTYIHSMN
PAILEDWNFGLTTPPSGSLEDTYRFVTSQAITCQKTAPQKPKEDPFKDYVFWEVNLKEKFSADLDQ
FPLGRKFLLQAGYRARPKFKAGKRSAPSASTTTPAKRKKTKK
00000000000000000000000000000000000000000000000000000000000000000
00000000000000000000000000000000000000000000000000000000000000000
00000000000000000000000000000000000000000000000000000000000000000
00000000000000000000000000000000000000000000000000000000000000000
00000000000000000000000000000000000000000000000000000000000000000
00000000000000000000000000000000000000000000000000000000000000000
00000000000000000000000000111111111111111111110000000000000000000
000000000000000000000000000000000000000000
> P11990 TACY_STRPN
ANKAVNDFILAMNYDKKKLLTHQGESIENRFIKEGNQLPDEFVVIERKKRSLSTNTSDISVTATND
SRLYPGALLVVDETLLENNPTLLAVDRAPMTYSIDLPGLASSDSFLQVEDPSNSSVRGAVNDLLAK

WHQDYGQVNNVPARMQYEKITAHSMEQLKVKFGSDFEKTGNSLDIDFNSVHSGEKQIQIVNFKQIY
YTVSVDAVKNPGDVFQDTVTVEDLKQRGISAERPLVYISSVAYGRQVYLKLETTSKSDEVEAAFEA
LIKGVKVAPQTEWKQILDNTEVKAVILGGDPSSGARVVTGKVDMVEDLIQEGSRFTADHPGLPISY
TTSFLRDNVVATFQNSTDYVETKVTAYRNGDLLLDHSGAYVAQYYITWNELSYDHQGKEVLTPKAW
DRNGQDLTAHFTTSIPLKGNVRNLSVKIRECTGLAWEWWRTVYEKTDLPLVRKRTISIWGTTLYPQ
VEDKVEND
000000000000000000000000000000000000000000000000000000000000000000
000000000000000000000000000000000000000000000000000000000000000000
000000000000000000000000000000000000000000000000000000000000000001
111111111111111111111111111111111111111111111111100000000000000000
000000000000000000000000000000000111111111111111111111111111111111
111111111111111111111111111111111111111111111111111111111111111111
111111111111111111111111111111111111111111111111111111111111111111
11111111
> P03099 VL1_HPV1A
MYNVFQMAVWLPAQNKFYLPPQPITRILSTDEYVTRTNLFYHATSERLLLVGHPLFEISSNQTVTI
PKVSPNAFRVFRVRFADPNRFAFGDKAIFNPETERLVWGLRGIEIGRGQPLGIGITGHPLLNKLDD
AENPTNYINTHANGDSRQNTAFDAKQTQMFLVGCTPASGEHWTSSRCPGEQVKLGDCPRVQMIESV
IEDGDMMDIGFGAMDFAALQQDKSDVPLDVVQATCKYPDYIRMNHEAYGNSMFFFARREQMYTRHF
FTRGGSVGDKEAVPQSLYLTADAEPRTTLATTNYVGTPSGSMVSSDVQLFNRSYWLQRCQGQNNGI
CWRNQLFITVGDNTRGTSLSISMKNNASTTYSNANFNDFLRHTEEFDLSFIVQLCKVKLTPENLAY
IHTMDPNILEDWQLSVSQPPTNPLEDQYRFLGSSLAAKCPEQAPPEPQTDPYSQYKFWEVDLTERM
SEQLDQFPLGRKFLYQSGMTQRTATSSTTKRKTVRVSTSAKRRRKA
000000000000000000000000000000000000000000000000000000000000000000
000000000000000000000000000000000000000000000000000000000000000000
000000000000000000000000000000000000000000000000000000000000000000
000000000000000000000000000000000000000000000000000000000000000000
000000000000000000000000000000000000000000000000000000000000000000
000000000000000000000000000000000000000000000000000000000000000000
000000000000000000000000000000111111111111111111111000000000000000
0000000000000000000000000000000000000000000000
> P03106 VL2_HPV6B
MAHSRARRRKRASATQLYQTCKLTGTCPPDVIPKVEHNTIADQILKWGSLGVFFGGLGIGTGSGTG
GRTGYVPLQTSAKPSITSGPMARPPVVVEPVAPSDPSIVSLIEESAIINAGAPEIVPPAHGGFTIT
SSETTTPAILDVSVTSHTTTSIFRNPVFTEPSVTQPQPPVEANGHILISAPTVTSHPIEEIPLDTF
VVSSSDSGPTSSTPVPGTAPRPRVGLYSRALHQVQVTDPAFLSTPQRLITYDNPVYEGEDVSVQFS
HDSIHNAPDEAFMDIIRLHRPAIASRRGLVRYSRIGQRGSMHTRSGKHIGARIHYFYDISPIAQAA
EEIEMHPLVAAQDDTFDIYAESFEPGINPTQHPVTNISDTYLTSTPNTVTQPWGNTTVPLSLPNDL

FLQSGPDITFPTAPMGTPFSPVTPALPTGPVFITGSGFYLHPAWYFARKRRKRIPLFFSDVAA
000000000000000000000000000000000000000000000000000000000000000000
000000000000000000000000000000000000000111111111111111111111110000
000000000000000000111111111111111111111110111111111111111111111111
101111111111111111100000000000000000000000000000000000000000000000
000000000000000000000000000000000000000000000000000000000000000000
000000000000000000001111111111111111100000000000000000000000000000
000000000000000000000000000000000000000000000000000000000000000
> P24025 VMSA_HBVIA
GQNLSTSNPLGFFPDHQLDPAFRANTANPDWDFNPNKDSWPDANKVGAGAFGLGFTPPHGGLLGWS
PQAQGILQTLPANPPPASTNRQSGRQPTPLSPPLRNTHPQAMQWNSTTFHQTLQDPRVRGLYLPAG
GSSSGTVNPVPTTVSPISSIFSRIGDPALNMENITSGFLGPLLVLQAGFFLLTKILTIPKSLDSWW
TSLNFLGGTTVCLGQNSQSPTSNHSPTSCPPTCPGYRWMCLRRFIIFLFILLLCLIFLLVLLDYQG
MLPVCPLIPGSSTTSTGPCRTCTTPAQGTSMYPSCCCTKPSDGNCTCIPIPSSWAFGKFLWEWASA
RFSWLSLLVPFVQWFVGLSPTVWLLVIWMMWYWGPKLFTILSPFLPLLPIFFCLWVYI
000000000000000000000000000000000000000000000000000000000000000000
000000000000000000000000000000000000000001111111111110000000000000
000000000000000000000000000000000000000000000000000000000000000000
000000000000000000000000000000000000000000000000000000000000000000
000000000000000000000000000000000000000000000000000000000000000000
0000000000000000000000000000000000000000000000000000000000
> P03102 VL1_CRPVK
MAVWLSTQNKFYLPPQPVTKIPSTDEYVTRTNVFYYASSDRLLTVGHPYYEIRDKGTMLVPKVSPN
QYRVFRIKLPDPNKFAFGDKQLYDPEKERLVWCLRGIEVNRGQPLGVSVTGNPIFNKFDDVENPTK
YYNNHADQQDYRKSMAFDPKQVQLLMLGCVPATGEHWAQAKQCAEDPPQQTDCPPIELVNTVIEDG
DMCEIGFGAMDHKTLQASLSEVPLELAQSISKYPDYLKMQKDQFGDSMFFYARREQMYARHFFSRA
GGDKENVKSRAYIKRTQMQGEANANIATDNYCITPSGSLVSSDSQVFNRAYWLQKAQGMNNGVCWD
NQIFVTVVDNTRGTILSLVTKSKEQIKKTHGKTVHFSSYLRHVEEYELQFVLQLCKVKLTPENLSY
LHSMHPTIIDNWQLSVSAQPSGTLEDQYRYLQSIATKCPPPEPPKENTDPYKNYKFWEVDLSEKLS
DQLDQYPLGRKFLNQSGLQRIGTKRPAPAPVSIVKSSKRKRRT
000000000000000000000000000000000000000000000000000000000000000000
000000000000000000000000000000000000000000000000000000000000000000
000000000000000000000000000000000000000000000000000000000000000000
000000000000000000000000000000000000000000000000000000000000000000
000000001111110000000000000000000000000000000000000000000000000000
000000000000000000000000000000000000000000000000000000000000000000
000000000000000000000000000000000000000000000000000000000000000000
0000000000000000000000000000000000000000000

> Q8X7B6 TRPB_ECO57
TTLLNPYFGEFGGMYVPQILMPALRQLEEAFVSAQKDPEFQAQFNDLLKNYAGRPTALTKCQNITA
GTNTTLYLKREDLLHGGAHKTNQVLGQALLAKRMGKTEIIAETGAGQHGVASALASALLGLKCRIY
MGAKDVERQSPNVFRMRLMGAEVIPVHSGSATLKDACNEALRDWSGSYETAHYMLGTAAGPHPYPT
IVREFQRMIGEETKAQILEREGRLPDAVIACVGGGSNAIGMFADFINETNVGLIGVEPGGHGIETG
EHGAPLKHGRVGIYFGMKAPMMQTEDGQIEESYSISAGLDFPSVGPQHAYLNSTGRADYVSITDDE
ALEAFKTLCLHEGIIPALESSHALAHALKMMRENPEKEQLLVVNLSGRGDKDIFTVHDILKARGEI
000000000000000000000000000000000000000000000000000000000000000000
000000000000000000000000000000000000000000000000000000000000000000
000000000000000000000000000000000000000000000000000000000000000000
000000000000000000000000000000000000000000000000000000000000000000
000001111111111111000000000000000000000000000000000000000000000000
000000000000000000000000000000000000000000000000000000000000000000
> P42375 CH60_PORGI
MAKEIKFDMESRDLLKKGVDALANAVKVTLGPKGRNVILSKTYGAPHITKDGVSVAKEIELECPFE
NMGAQLVKEVASKTNDDAGDGTTTATILAQSIIGVGLKNVTAGANPMDLKRGIDKAVKAVVTHIAG
MAKEVGDDFQKIEHVAKISANGDENIGSLIAEAMRKVKKEGVITVEEAKGTDTTVEVVEGMQFDRG
YISPYFVTNTDKMEVQMENPFILIYDKKISVLKEMLPILEQTVQTGKPLLIIAEDIDSEALATLVV
NRLRGSLKICAVKAPGFGDRRKAMLEDIAILTGGTVISEETGLKLENATMDMLGTAEKVTVDKDNT
TIVNGAGNKEGIASRITQIKAQIENTTSDYDREKLQERLAKLAGGVAVLYVGAASEVEMKEKKDRV
EDALSATRAAIEEGTVPGGGTAYIRAIAALEGLKGENEDETTGIEIVKRAIEEPLRQIVANAGKEG
AVVVQKVKEGKDDFGYNARTDVFENLYTTGVIDPAKVTRVALENAASIAGMFLTTECVIADKKEDN
PAAPAMPGGMGGMGGMM
000000000001111111111000000000000000000000000000000000000000000000
000000111111111100000000000000000000000000000000000000000000000000
000000000111111111100000000001111111111111100000000000000000111111
111100000000000000000001111111111000000000000000000000000000000111
111111100000011111111111111110000000000000000000000000000000000000
000001111111111111100000000000000011111111111111000000000000000000
000000000000000000000000111111111000000001111111111000000000000000
000000000000000000000000000000111111111100000111111111000000000000
00000000000000000
> Q00663 CARP_CANTR
MATIFLFTKNVFIALAFALFAQGLTIPDGIEKRTDKVVSLDFTVIRKPFNATAHRLIQKRSDVPTT
LINEGPSYAADIVVGSNQQKQTVVIDTGSSDLWVVDTDAECQVTYSGQTNNFCKQEGTFDPSSSSS
AQNLNQDFSIEYGDLTSSQGSFYKDTVGFGGISIKNQQFADVTTTSVDQGIMGIGFTAVEAGYNLY
SNVPVTLKKQGIINKNAYSCDLNSEDASTGKIIFGGVDNAKYTGTLTALPVTSSVELRVHLGSINF
DGTSVSTNADVVLDSGTTITYFSQSTADKFARIVGATWDSRNEIYRLPSCDLSGDAVVNFDQGVKI

TVPLSELILKDSDSSICYFGISRNDANILGDNFLRRAYIVYDLDDKTISLAQVKYTSSSDISAL
000000000000000000000000000000000000000000000000000000000000000000
000000000000000000000000000000000000000000000000000000000000000000
000000000000000000000000111111111111110000000000000000000000000000
000000000000000000000000000000000000000000000000000000000000000000
000000000000000000000000000000000000000000000000000000000000000000
0000000000000000000000000000000000000000000000000000000000000000
> P05787 K2C8_HUMAN
SIRVTQKSYKVSTSGPRAFSSRSYTSGPGSRISSSSFSRVGSSNFRGGLGGGYGGASGMGGITAVT
VNQSLLSPLVLEVDPNIQAVRTQEKEQIKTLNNKFASFIDKVRFLEQQNKMLETKWSLLQQQKTAR
SNMDNMFESYINNLRRQLETLGQEKLKLEAELGNMQGLVEDFKNKYEDEINKRTEMENEFVLIKKD
VDEAYMNKVELESRLEGLTDEINFLRQLYEEEIRELQSQISDTSVVLSMDNSRSLDMDSIIAEVKA
QYEDIANRSRAEAESMYQIKYEELQSLAGKHGDDLRRTKTEISEMNRNISRLQAEIEGLKGQRASL
EAAIADAEQRGELAIKDANAKLSELEAALQRAKQDMARQLREYQELMNVKLALDIEIATYRKLLEG
EESRLESGMQNMSIHTKTTGGYAGGLSSAYGGLTSPGLSYSLGSSFGSGAGSSSFSRTSSSRAVVV
KKIETRDGKLVSESSDVLPK
000000000000000000000000000000000000000000000000000000000000000000
000000000000000000000000000000000000000000000000000000000000000000
000000000000000000000000000000000000000000000000000000000000000000
000000000000000000000000000000000000000000000000000000000000000000
000000000000000000000000000000000000000000000000000000000000000000
000000111111111111111111111111100000000000000000000000000000000000
000000000000000000000000000000000000000000000000000000000000000000
00000000000000000000
> P17203 CH60_CHLTR
VAKNIKYNEEARKKIQKGVKTLAEAVKVTLGPKGRHVVIDKSFGSPQVTKDGVTVAKEVELADKHE
NMGAQMVKEVASKTADKAGDGTTTATVLAEAIYTEGLRNVTAGANPMDLKRGIDKAVKVVVDQIRK
ISKPVQHHKEIAQVATISANNDAEIGNLIAEAMEKVGKNGSITVEEAKGFETVLDIVEGMNFNRGY
LSSYFATNPETQECVLEDALVLIYDKKISGIKDFLPVLQQVAESGRPLLIIAEDIEGEALATLVVN
RIRGGFRVCAVKAPGFGDRRKAMLEDIAILTGGQLISEELGMKLENANLAMLGKAKKVIVSKEDTT
IVEGMGEKEALEARCESIKKQIEDSSSDYDKEKLQERLAKLSGGVAVIRVGAATEIEMKEKKDRVD
DAQHATIAAVEEGILPGGGTALIRCIPTLEAFLPMLTNEDEQIGARIVLKALSAPLKQIAANAGKE
GAIIFQQVMSRSANEGYDALRDAYTDMLEAGILDPAKVTRSALESAASVAGLLLTTEALIAEIPEE
KPAAAPAMPGAGMDY
000000000000000000000000000111111111111000000000000000000000000000
000000000000000000000000001111111111110000000000000000000000000000
000000000000000001111111111110000000000000000000000000000000000000
000000000000000000000000000011111111111100000000000000000000000000

000000000000000000000000000000000000000000000000000000000000000000
000000000000000000000000000000000000000000000000000000000000000000
000000000000011111111111100000000000011111111111100011111111111111
111111111111111111000000000000000000000000000000000000000000000000
000000000000000
> P06458 VL1_BPV2
MALWQQGQKLYLPPTPVSKVLCSETYVQRKSIFYHAETERLLTVGHPYYQVTVGDKTVPKVSANQF
RVFKIQLPDPNQFALPDRTVHNPSKERLVWAVIGVQVSRGQPLGGTVTGHPTFNALLDAENVNRKV
TAQTTDDRKQTGLDAKQQQILLLGCTPAEGEYWTTARPCVTDRLENGACPPLELKNKHIEDGDMME
IGFGAADFKTLNASKSDLPLDIQNEICLYPDYLKMAEDAAGNSMFFFARKEQVYVRHIWTRGGSEK
EAPSKDFYLKNGRGEETLKIPSVHFGSPSGSLVSTDNQIFNRPYWLFRAQGMNNGIAWNNLLFLTV
GDNTRGTNLSISVAADGNALSEYDTGKFNLYHRHMEEYKLAFILELCSVEITAQTLSHLQGLMPSV
LQNWEIGVQPPASSILEDTYRYIESPATKCASNVIPPKEDPYAGLKFWSIDLKEKLSLDLDQFPLG
RRFLAQQGAGCSTVRKRAVATRNSSKPAKRKKIKA
000000000000000000000000000000000000000001111111111111110000000000
000000000000000000000000000000000000000000000000000000000000000000
000000000000000000000000000000000000000000000000000000000000000000
000000000000000000000000000000000000000000000000000000000000000000
000000000000000000000000000000000000000000000000000000000000000000
000000000000000000000000000000000000000000000000000000000000000000
000000000000000000000000000000000000000000000000000000000000000000
00000000000000000000111111111111111
> P37708 NCAP_RINDL
MASLLKSLALFKKNKDKPPLAAGSGGAIRGIKHVIIVPIPGDSSITTRSRLLDCLVKMVGDPDISG
PKLTGALISILSLFVESPGQLIQRITDDPDISIKLVEVIQSDKTQSGLTFASRGASMDDEADRYFT
YDEPNGGEERQSYWFENREIQDIEVQDPEGFNMILATILAQIWILLAKAVTTPDTAADSELRRWVK
YTQQRRVIGEFRLDKGWLDTVRNRIAEDLSLRRFMVALILDIKRTPGNKPRIAEMICDIDTYIVEA
GLASFILTIKFGIETMYPALGLHEFAGELSTIESLMNLYQQMGELAPYMVILENSIQNKFSAGAYP
LLWSYAMGVGVELESSMGGLNFGRSYFDPAYFRLGQEMVRRSAGKVSSNLASELGITEEEAKLVSE
IAAYTGDDRNSRTSGPKQTQVSFLRTDQGGEIQHNASKKDEARVLQVRKETWASSRSDRYKEDTDN
EAVSPSVKTLIDVDTTPEADTDPLGNKKSAEALLKLQAMASILEDPTLGNDSPRTYNDKDLLS
000000000000000000000000000000000000000000000000000000000000000000
000000000000000000000000000000000000000000000000000000000000000000
000000000000000000000000000000000000000000000000000000000000000000
000000000000000000000000000000000000000000000000000000000000000000
000000000000000000000000000000000000000000000000000000000000000000
000000000000000000000000000000000000000000000000000000000000000000
000000000000000000000000000000000000000000000000000000000000000000

0000000111111111111111100000000000000000000000000000000000000000
> P03145 VMSA_HPBDU
GQHPAKSMDVRRIEGGEILLNQLAGRMIPKGTLTWSGKFPTLDHVLDHVQTMEEINTLQNQGAWPA
GAGRRVGLSNPTPQEIPQPQWTPEEDQKAREAFRRYQEERPPETTTIPPSSPPQWKLQPGDDPLLG
NQSLLETHPLYQSEPAVPVIKTPPLKKKMSGTFGGILAGLIGLLVSFFLLIKILEILRRLDWWWIS
LSSPKGKMQCAFQDTGAQISPHYVGSCPWGCPGFLWTYLRLFIIFLLILLVAAGLLYLTDNGSTIL
GKLQWASVSALFSSISSLLPSDPKSLVALTFGLSLIWMTSSSATQTLVTLTQLATLSALFYKS
00000111111111111111111111111000000000011111111111000001111111100
00011111111100111111111111000000000000000001111111110011111111111
11000000000000000000000000000000000000000000000000000000000000000
00000000000000000000000000000000000000000000000000000000000000000
000000000000000000000000000000000000000000000000000000000000000
> P79017 ALL2_ASPFU
MAALLRLAVLLPLAAPLVATLPTSPVPIAARATPHEPVFFSWDAGAVTSFPIHSSCNATQRRQIEA
GLNEAVELARHAKAHILRWGNESEIYRKYFGNRPTMEAVGAYDVIVNGDKANVLFRCDNPDGNCAL
EGWGGHWRGANATSETVICDRSYTTRRWLVSMCSQGYTVAGSETNTFWASDLMHRLYHVPAVGQGW
VDHFADGYDEVIALAKSNGTESTHDSEALQYFALEAYAFDIAAPGVGCAGESHGPDQGHDTGSASA
PASTSTSSSSSGSGSGATTTPTDSPSATIDVPSNCHTHEGGQLHCT
00000000000000000000000000000000000000000000000000000011111111111
10000000000000000000111111111111111000000000000000000000000000011
11111111111111100111111111111111000000000001111111111111111111111
11111111111000000000000000000011111111111100000000000000000000000
0000000000000000000000000000000011111111111111
> P36342 VGLD_CHV1
MGSGIAAVLLSLAVALARVPAGEGEYVPVERSLTRVNPGRFRGAHLPPLEQKTDPPDVRRVYHVQP
FVENPFQTPSVPVAVYYAVLERACRSVLLWAPTEAVQVVRGAPEATRSDARYNLTVAWYRTSDDCA
IPILVMEYAECQYDKPLGACPVRNLPRWSFYDSFSATGDDDLGLLMHAPAFETAGTYVRLVKVNGW
VEVTQFIFEHRGKGPCRYTLPLRILPAACLRAPVFEQGVTVDAIGMLPRFIPENQRIVAVYSLQAA
GWHGPKAPFTSTLLPPEVVETANVTRPELAPEERGTSRTPGDEPAPAVAAQLPPNWHVPEASDVTI
QGPAPAPSGHTGAVVGALAGAGLAAGVVVLAVYLVRRRGRAAGKHVRLPELLEEAHGPARRGAPY
00000000000000000000000000000000000000000000000000000000000000000
00000000000000000000000000000000000000000000000000000000000000000
00000000000000000000000000000000000000000000000000000000000000000
00000000000000000000000000000000000000000000000000000000000000000
00000000000000000000000000000000000000000000000000000000000000000
0000000000000000000000000000000000000000000000000000000111111111
> P30690 OMPB1_NEIMB
MKKSLIALTLAALPVAAMADVTLYGTIKAGVETSRSVFHQNGQVTEVTTATGIVDLGSKIGFKGQE

DLGNGLKAIWQVEQKASIAGTDSGWGNRQSFIGLKGGFGKLRVGRLNSVLKDTGDINPWDSKSDYL
GVNKIAEPEARLISVRYDSPEFAGLSGSVQYALNDNAGRHNSESYHAGFNYKNGGFFVQYGGAYKR
HHQVQEGLNIEKYQIHRLVSGYDNDALYASVAVQQQDAKLTDASNSHNSQTEVAATLAYRFGNVTP
RVSYAHGFKGLVDDADIGNEYDQVVVGAEYDFSKRTSALVSAGWLQEGKGENKFVATAGGVGLRHK
F
000000000000000000000000000000011111111111111111100000000000000000
011111111111111111111111111111100000000000000000000000000000000000
000000000000011111111111111111000000111111111111100000000000000000
000000000000000000000000000000000000000000000000000000011111111111
111111111111100000000000000000000000000000000000000000000000000000
0
> P10332 OM1E_CHLPS
MKKLLKSALLFAATGSALSLQALPVGNPAEPSLLIDGTMWEGASGDPCDPCATWCDAISIRAGYYG
DYVFDRVLKVDVNKTFSGMAATPTQATGNASNTNQPEANGRPNIAYGRHMQDAEWFSNAAFLALNI
WDRFDIFCTLGASNGYFKSSSAAFNLVGLIGFSATSSTSTELPMQLPNVGITQGVVEFYTDTSFSW
SVGARGALWECGCATLGAEFQYAQSNPKIEVLNVTSSPAQFVIHKPRGYKGASSNFPLPITAGTTE
ATDTKSATIKYHEWQVGLALSYRLNMLVPYIGVNWSRATFDADTIRIAQPKLKSEILNITTWNPSL
LGSTTTLPNNGGKDVLSDVLQIASIQINKMKSRKACGVAVGATLIDADKWSITGEARLINERAAHM
NAQFRF
000000000000000000000000000000000000000000000000000000000000000000
000000000000000000000000000000000000000000000000000000000000000000
000000000000000000000000000000000000000000000000000000000000000000
000000000000000000000000000000000000000000000000000000000000000000
000000000000000000000000000000000000000000000000000000000000000000
000000000000000001111111111110000000000000000000000000000000000000
000000
> P23114 OM1N_CHLTR
MKKLLKSVLVFAALSSASSLQALPVGNPAEPSLMIDGILWEGFGGDPCDPCTTWCDAISMRVGYYG
DFVFDRVLKTDVNKEFQMGAEPTTSDTAGLSNDPTTNVARPNPAYGKHMQDAEMFTNAAYMALNIW
DRFDVFCTLGATTGYLKGNSASFNLVGLFGTKTQSTNFNTAKLVPNTALNQAVVELYTDTTFAWSV
GARAALWECGCATLGASFQYAQSKPKVEELNVLCDASEFTINKPKGYVGAEFPLDITAGTEAATGT
KDASIDYHEWQASLALSYRLNMFTPYIGVKWSRVSFDADTIRIAQPKLAEAVLDVTTLNPTIAGKG
SVVASGSENELADTMQIVSLQLNKMKSRKSCGIAVGTTIVDADKYAVTVETRLIDERAAHVNAQFR
F
000000000000000000000000000000000000000000000000000000000000000000
000000000000000000000000000000000000000000000000000000000000000000
000000000000000000000000000000000000000000000000000000000000000000
000000000000000000000000000000000000000000000000011111110000000000

000000000000000000000000000000000000000000000000000000011111111000
000000000000000000000000000000000000000000000000000000000000000000
0
> P02931 OMPF_ECOLI
MMKRNILAVIVPALLVAGTANAAEIYNKDGNKVDLYGKAVGLHYFSKGNGENSYGGNGDMTYARLG
FKGETQINSDLTGYGQWEYNFQGNNSEGADAQTGNKTRLAFAGLKYADVGSFDYGRNYGVVYDALG
YTDMLPEFGGDTAYSDDFFVGRVGGVATYRNSNFFGLVDGLNFAVQYLGKNERDTARRSNGDGVGG
SISYEYEGFGIVGAYGAADRTNLQEAQPLGNGKKAEQWATGLKYDANNIYLAANYGETRNATPITN
KFTNTSGFANKTQDVLLVAQYQFDFGLRPSIAYTKSKAKDVEGIGDVDLVNYFEVGATYYFNKNMS
TYVDYIINQIDSDNKLGVGSDDTVAVGIVYQF
000000000000000000000000000000000000000000000000000000000000000000
111111111111111111110000000000000000000000000000001111111111111111
111111111111111111111111000000000000000000000000000000000000000000
000000000000000000000000000000000000000000000000000000000000000000
001111111111111111111100000000001111111111111111111100000000001111
11111111111111110000000000000000
> P03107 VL2_HPV16
MRHKRSAKRTKRASATQLYKTCKQAGTCPPDIIPKVEGKTIAEQILQYGSMGVFFGGLGIGTGSGT
GGRTGYIPLGTRPPTATDTLAPVRPPLTVDPVGPSDPSIVSLVEETSFIDAGAPTSVPSIPPDVSG
FSITTSTDTTPAILDINNTVTTVTTHNNPTFTDPSVLQPPTPAETGGHFTLSSSTISTHNYEEIPM
DTFIVSTNPNTVTSSTPIPGSRPVARLGLYSRTTQQVKVVDPAFVTTPTKLITYDNPAYEGIDVDN
TLYFSSNDNSINIAPDPDFLDIVALHRPALTSRRTGIRYSRIGNKQTLRTRSGKSIGAKVHYYYDL
STIDPAEEIELQTITPSTYTTTSHAASPTSINNGLYDIYADDFITDTSTTPVPSVPSTSLSGYIPA
NTTIPFGGAYNIPLVSGPDIPINITDQAPSLIPIVPGSPQYTIIADAGDFYLHPSYYMLRKRRKRL
PYFFSDVSLAA
000000000000000000000000000000000000000000000000000000000000000000
000000000000000000000000000000000000000001111111111111000000000000
000000000000000000000000000000000000000000000000000000000000000000
000000000000000000000000000000000000000000000000000000000000000000
000000000000000000000000000000000000000000000000000000000000000000
000000000000000000000000000000000000000000000000000000000000000000
000000000000000000000000000000000000000000000000000000000000000000
00000000000
> P25486 VL1_HPV2A
MSCGLNDVNVSTISLQMALWRPNESKVYLPPTPVSKVISTDVYVTRTNVYYHGGSSRLLTVGHPYY
SIKKSNNKVAVPKVSGYQYRVFHVKLPDPNKFGLPDADLYDPDTQRLLWACVGVEVGRGQPLGVGV
SGHPYYNRLDDTENAHTPDTADDGRENISMDYKQTQLFILGCKPPIGEHWSKGTTCNGSSAAGDCP
PLQFTNTTIEDGDMVETGFGALDFATLQSNKSDVPLDICTNTCKYPDYLKMAAEPYGDSMFFSLRR

EQMFTRHFFNLGGKMGDTIPDELYIKSTSVPTPGSHVYTSTPSGSMVSSEQQLFNKPYWLRRAQGH
NNGMCWGNRVFLTVVDTTRSTNVSLCATEASDTNYKATNFKEYLRHMEEYDLQFIFQLCKITLTPE
IMAYIHNMDPQLLEDWNFGVPPPPSASLQDTYRYLQSQAITCQKPTPPKTPTDPYASLTFWDVDLS
ESFSMDLDQFPLGRKFLLQRGAMPTVSRKRAAVSGTTPPTSKRKRVRR
000000000000000000000000000000000000000000000000000000000000000000
000000000000000000000000000000000000000000000000000000000000000000
000000000000000000000000000000000000000000000000000000000000000000
000000000000000000000000000000000000000000000000000000000000000000
000000000000000000001111110000000000000000000000000000000000000000
000000000000000000000000000000000000000000000000000000000000000000
000000000000000000000000000000000000000000000000000000000000000000
000000000000000000000000000000000000000000000000
> P00183 CPXA_PSEPU
TTETIQSNANLAPLPPHVPEHLVFDFDMYNPSNLSAGVQEAWAVLQESNVPDLVWTRCNGGHWIAT
RGQLIREAYEDYRHFSSECPFIPREAGEAYDFIPTSMDPPEQRQFRALANQVVGMPVVDKLENRIQ
ELACSLIESLRPQGQCNFTEDYAEPFPIRIFMLLAGLPEEDIPHLKYLTDQMTRPDGSMTFAEAKE
ALYDYLIPIIEQRRQKPGTDAISIVANGQVNGRPITSDEAKRMCGLLLVGGLDTVVNFLSFSMEFL
AKSPEHRQELIERPERIPAACEELLRRFSLVADGRILTSDYEFHGVQLKKGDQILLPQMLSGLDER
ENACPMHVDFSRQKVSHTTFGHGSHLCLGQHLARREIIVTLKEWLTRIPDFSIAPGAQIQHKSGIV
SGVQALPLVWDPATTKAV
000000111111100000000000000000000000000000000000000001111110001111
111111000000000000000001111111011111100001111111111000011111100000
000000001111110111111100000000000000000001111110111111111110000000
000000000000011111100000001111111111100000000000000000000000000011
111111100000000000000000000000000011111100001111111111100011111100
000000000000000000000000000001111110111111000000000000000000000011
111100000011111100
> P04012 VL1_HPV11
MWRPSDSTVYVPPPNPVSKVVATDAYVKRTNIFYHASSSRLLAVGHPYYSIKKVNKTVVPKVSGYQ
YRVFKVVLPDPNKFALPDSSLFDPTTQRLVWACTGLEVGRGQPLGVGVSGHPLLNKYDDVENSGGY
GGNPGQDNRVNVGMDYKQTQLCMVGCAPPLGEHWGKGTQCSNTSVQNGDCPPLELITSVIQDGDMV
DTGFGAMNFADLQTNKSDVPLDICGTVCKYPDYLQMAADPYGDRLFFYLRKEQMFARHFFNRAGTV
GEPVPDDLLVKGGNNRSSVASSIYVHTPSGSLVSSEAQLFNKPYWLQKAQGHNNGICWGNHLFVTV
VDTTRSTNMTLCASVSKSATYTNSDYKEYMRHVEEFDLQFIFQLCSITLSAEVMAYIHTMNPSVLE
DWNFGLSPPPNGTLEDTYRYVQSQAITCQKPTPEKEKQDPYKDMSFWEVNLKEKFSSELDQFPLGR
KFLLQSGYRGRTSARTGIKRPAVSKPSTAPKRKRTKTKK
000000000000000000000000000000000000000000000000000000000000000000
000000000000000000000000000000000000000000000000000000000000000000

000000000000000000000000000000000000000000000000000000000000000000
000000000000000000000000000000000000000000000000000000000000000000
000000000000000000000000000000000000000000000000000000000000000000
000000000000000000000000000000000000000000000000000000000000000000
000000000000000000000111111111111111111111000000000000000000000000
000000000000000000000000000000000000000
> P03101 VL1_HPV16
MQVTFIYILVITCYENDVNVYHIFFQMSLWLPSEATVYLPPVPVSKVVSTDEYVARTNIYYHAGTS
RLLAVGHPYFPIKKPNNNKILVPKVSGLQYRVFRIHLPDPNKFGFPDTSFYNPDTQRLVWACVGVE
VGRGQPLGVGISGHPLLNKLDDTENASAYAANAGVDNRECISMDYKQTQLCLIGCKPPIGEHWGKG
SPCTNVAVNPGDCPPLELINTVIQDGDMVDTGFGAMDFTTLQANKSEVPLDICTSICKYPDYIKMV
SEPYGDSLFFYLRREQMFVRHLFNRAGAVGENVPDDLYIKGSGSTANLASSNYFPTPSGSMVTSDA
QIFNKPYWLQRAQGHNNGICWGNQLFVTVVDTTRSTNMSLCAAISTSETTYKNTNFKEYLRHGEEY
DLQFIFQLCKITLTADVMTYIHSMNSTILEDWNFGLQPPPGGTLEDTYRFVTSQAIACQKHTPPAP
KEDPLKKYTFWEVNLKEKFSADLDQFPLGRKFLLQAGLKAKPKFTLGKRKATPTTSSTSTTAKRKK
RKL
000000000000000000000000000000000000000000000000000000000000000000
000000000000000000000000000000000000000011111111111111111111000000
000000000000000000111111111111111101111111111111111111111111111111
111111111111111111000000000000000000000000011111111111111111111000
000000000000000000000000000000111111111111111100000000000000000000
111111111111111011111111111111111111000000000000000000000000000000
000000000000000000000000000000000000000011111111111111111111111111
111111111000000000111111111111111111111111111111111111000000000000
000
> P00187 CP1A2_RABIT
AMSPAAPLSVTELLLVSAVFCLVFWAVRASRPKVPKGLKRLPGPWGWPLLGHLLTLGKNPHVALAR
LSRRYGDVFQIRLGSTPVVVLSGLDTIKQALVRQGDDFKGRPDLYSSSFITEGQSMTFSPDSGPVW
AARRRLAQDSLKSFSIASNPASSSSCYLEEHVSQEAENLIGRFQELMAAVGRFDPYSQLVVSAARV
IGAMCFGRRFPQGSEEMLDVVRNSSKFVETASSGSPVDFFPILRYLPNRPLQRFKDFNQRFLRFLQ
KTVREHYEDFDRNSIQDITGALFKHSEKNSKANSGLIPQEKIVNLVNDIFGAGFDTITTALSWSLM
YLVTNPRRQRKIQEELDAVVGRARQPRLSDRPQLPYLEAFILELFRHTSFVPFTIPHSTTRDTTLN
GFHIPKECCIFINQWQINHDPQLWGDPEEFRPERFLTADGAAINKPLSEKVTLFGLGKRRCIGETL
ARWEVFLFLAILLQRLEFSVPPGVPVDLTPIYGLTMKHPRCEHVQARPRFSDQ
000000000000000000000000000000000000000011111110000000000000000000
000000000000000000000000000011111000000011111000000011111111111111
111111111000000000000000000000000000000000000000000000000001111111
110001111111110000001111110000000000000000111111111111111111000000

111111000000011111111000000000000000000000000000000000000000000000
011111111111100111111111111000111111000000000000000000000000000000
000000000000000000000000111111111110000000000000000000000000011111
10000000000000000000000000000000000111111111001111111
> P05455 LA_HUMAN
MAENGDNEKMAALEAKICHQIEYYFGDFNLPRDKFLKEQIKLDEGWVPLEIMIKFNRLNRLTTDFN
VIVEALSKSKAELMEISEDKTKIRRSPSKPLPEVTDEYKNDVKNRSVYIKGFPTDATLDDIKEWLE
DKGQVLNIQMRRTLHKAFKGSIFVVFDSIESAKKFVETPGQKYKETDLLILFKDDYFAKKNEERKQ
NKVEAKLRAKQEQEAKQKLEEDAEMKSLEEKIGCLLKFSGDLDDQTCREDLHILFSNHGEIKWIDF
VRGAKEGIILFKEKAKEALGKAKDANNGNLQLRNKEVTWEVLEGEVEKEALKKIIEDQQESLNKWK
SKGRRFKGKGKGNKAAQPGSGKGKVQFQGKKTKFASDDEHDEHDENGATGPVKRAREETDKEEPAS
KQQKTENGAGDQ
111111111111111111110000000000000000000000000000000000000000000000
000000000000000000000000000000000000000000000000000000000000000000
000000000000111111111111111111110000111111111111111111111111111111
110000000000000000000000000000000000000000000000000000111111111111
111111111111111111110000111111111111111111111111111111111111111111
110000000000000000111111111111111111111111111111111111111111111111
111111111111
> P13467 OM1H_CHLTR
MKKLLKSVLVFAALSSASSLQALPVGNPAEPSLMIDGILWEGFGGDPCDPCATWCDAISMRVGYYG
DFVFDRVLKTDVNKEFQMGAAPTTNDAADLQNDPKTNVARPNPAYGKHMQDAEMFTNAAYMALNIW
DRFDVFCTLGATTGYLKGNSASFNLVGLFGTKTKSSDFNTAKLVPNIALNRAVVELYTDTTFAWSV
GARAALWECGCATLGASFQYAQSKPKVEELNVLCNASEFTINKPKGYVGAEFPLDITAGTEAATGT
KDASIDYHEWQASLALSYRLNMFTPYIGVKWSRVSFDADTIRIAQPKLAEAILDVTTLNPTIAGKG
TVVASGSDNDLADTMQIVSLQLNKMKSRKSCGIAVGTTIVDADKYAVTVETRLIDERAAHVNAQFR
F
000000000000000000000000000000000000000000000000000000000000000000
000000000000000000000011111111111000000000000000000000000000000000
000000000000000000000000000000000000000000000000000000000000000000
000000000000000000000000000000000000000000000000000000000000000000
000000000000000000000000000000000000000000000000000000000000000000
000000000000000000000000000000000000000000000000000000000000000000
0
> P06794 VL1_HPV18
MCLYTRVLILHYHLLPLYGPLYHPRPLPLHSILVYMVHIIICGHYIILFLRNVNVFPIFLQMALWR
PSDNTVYLPPPSVARVVNTDDYVTPTSIFYHAGSSRLLTVGNPYFRVPAGGGNKQDIPKVSAYQYR
VFRVQLPDPNKFGLPDTSIYNPETQRLVWACAGVEIGRGQPLGVGLSGHPFYNKLDDTESSHAATS

NVSEDVRDNVSVDYKQTQLCILGCAPAIGEHWAKGTACKSRPLSQGDCPPLELKNTVLEDGDMVDT
GYGAMDFSTLQDTKCEVPLDICQSICKYPDYLQMSADPYGDSMFFCLRREQLFARHFWNRAGTMGD
TVPQSLYIKGTGMPASPGSCVYSPSPSGSIVTSDSQLFNKPYWLHKAQGHNNGVCWHNQLFVTVVD
TTPSTNLTICASTQSPVPGQYDATKFKQYSRHVEEYDLQFIFQLCTITLTADVMSYIHSMNSSILE
DWNFGVPPPPTTSLVDTYRFVQSVAITCQKDAAPAENKDPYDKLKFWNVDLKEKFSLDLDQYPLGR
KFLVQAGLRRKPTIGPRKRSAPSATTSSKPAKRVRVRARK
000000000000000000000000000000000000000000000000000000000000000000
000000000000000000000000000000000000000000000000000000000000000000
000000000000000000000000000000000000000000000000000000000000000000
000000000000000000000000000000000000000000000000000000000000000000
000000000000000000000000000000000000000000000000000000000000000000
000111111000000000000000000000000000000000000000000000000000000000
000000000000000000000000000000000000000000000000000000000000000000
000000000000000000000111111111111111111111000000000000000000000000
0000000000000000000000000000000000000000
> P19542 OM1L_CHLTR
MKKLLKSVLVFAALSSASSLQALPVGNPAEPSLMIDGILWEGFGGDPCDPCTTWCDAISMRMGYYG
DFVFDRVLQTDVNKEFQMGAKPTATTGNAAAPSTCTARENPAYGRHMQDAEMFTNAAYMALNIWDR
FDVFCTLGATSGYLKGNSASFNLVGLFGDNENQSTVKKDAVPNMSFDQSVVELYTDTTFAWSVGAR
AALWECGCATLGASFQYAQSKPKVEELNVLCNAAEFTINKPKGYVGKEFPLDLTAGTDAATGTKDA
SIDYHEWQASLALSYRLNMFTPYIGVKWSRASFDADTIRIAQPKLATAIFDTTTLNPTIAGAGEVK
ANAEGQLGDTMQIVSLQLNKMKSRKSCGIAVGTTIVDADKYAVTVETRLIDERAAHVNAQFRF
000000000000000000000000000000000000000000000000000000000000000000
000000000000000000000000000000000000000000000000000000000000000000
000000000000000000000000000000000000111111100000000000000000000000
000000000000000000000000000000000000000000000000000000000000000000
000000000000000000000000000000000000000000000011111111111111000000
000000000011111111100000000000000000000000000000000000000000000
> P77793 YDDV_ECO57
MEMYFKRMKDEWTGLVEQADPPIRAKAAEIAVAHAHYLSIEFYRIVRIDPHAEEFLSNEQVERQLK
SAMERWIINVLSAQVDDVERLIQIQHTVAEVHARIGIPVEIVEMGFRVLKKILYPVIFSSDYSAAE
KLQVYHFSINSIDIAMEVMTRAFTFSDSSASKEDENYRIFSLLENAEEEKERQIASILSWEIDIIY
KILLDSDLGSSLPLSQADFGLWFNHKGRHYFSGIAEVGHISRLIQDFDGIFNQTMRNTRNLNNRSL
RVKFLLQIRNTVSQIITLLRELFEEVSRHEVGMDVLTKLLNRRFLPTIFKREIAHANRTGTPLSVL
IIDVDKFKEINDTWGHNTGDEILRKVSQAFYDNVRSSDYVFRYGGDEFIIVLTEASENETLRTAER
IRSRVEKTKLKAANGEDIALSLSIGAAMFNGHPDYERLIQIADEALYIAKRRGRNRVELWKASL
000000000000000000000000000000000000000000000000000000000000111111
111111111111111111111111110000000000000000000000000000000000000000

000000000000000000000000000000000000000000000000000000000000000000
000000000000000000000000000000000000000000000000000000000000000000
000000000000000000000000000000000000000000000000000000000000000000
000000000000000000000000000000000000000000000000000000000000000000
0000000000000000000000000000000000000000000000000000000000000000
> P27313 NCAP_PUUMS
MSDLTDIQEDITRHEQQLIVARQKLKDAERAVEVDPDDVNKNTLQARQQTVSALEDKLADYKRRMA
DAVSRKKMDTKPTDPTGIEPDDHLKERSSLRYGNVLDVNAIDIEEPSGQTADWYTIGVYVIGFTLP
IILKALYMLSTRGRQTVKENKGTRIRFKDDTSFEDINGIRRPKHLYVSMPTAQSTMKAEELTPGRF
RTIVCGLFPTQIQVRNIMSPVMGVIGFSFFVKDWSERIREFMEKECPFIKPEVKPGTPAQEIEMLK
RNKIYFMQRQDVLDKNHVADIDKLIDYAASGDPTSPDNIDSPNAPWVFACAPDRCPPTCIYVAGMA
ELGAFFSILQDMRNTIMASKTVGTAEEKLKKKSSFYQSYLRRTQSMGIQLDQRIILLFMLEWGKEM
VDHFHLGDDMDPELRGLAQALIDQKVKEISNQEPLKI
000000000111111111111111111111100000000000000000000111111111111111
111110000000111111111111110111111111111110000000000000000000111111
111111100000001111111111111100000000000000000000000000000000000000
000000000000000000000000000000000000000000000000111111111111111110
111111111111111111110000000000000000000000000000000000000000000000
000000000000000000000000000000000000000000000000000000000000000000
0000000000001111111111111100000000000
> P03103 VL1_BPV1
MALWQQGQKLYLPPTPVSKVLCSETYVQRKSIFYHAETERLLTIGHPYYPVSIGAKTVPKVSANQY
RVFKIQLPDPNQFALPDRTVHNPSKERLVWAVIGVQVSRGQPLGGTVTGHPTFNALLDAENVNRKV
TTQTTDDRKQTGLDAKQQQILLLGCTPAEGEYWTTARPCVTDRLENGACPPLELKNKHIEDGDMME
IGFGAANFKEINASKSDLPLDIQNEICLYPDYLKMAEDAAGNSMFFFARKEQVYVRHIWTRGGSEK
EAPTTDFYLKNNKGDATLKIPSVHFGSPSGSLVSTDNQIFNRPYWLFRAQGMNNGIAWNNLLFLTV
GDNTRGTNLTISVASDGTPLTEYDSSKFNVYHRHMEEYKLAFILELCSVEITAQTVSHLQGLMPSV
LENWEIGVQPPTSSILEDTYRYIESPATKCASNVIPAKEDPYAGFKFWNIDLKEKLSLDLDQFPLG
RRFLAQQGAGCSTVRKRRISQKTSSKPAKKKKK
000000000000000000000000000111111111100000011111111110000000000111
111110000000000000111111111111111111011111100111111111111111111111
111111111111111111111111111111111111111110000000000001111110001111
111111111111111111111111100001111111000011111111111111111111100011
111111111100000000111111111111111111111111111111111011111111111111
101111111111110000000001111111111111011111111111100000000000000011
111111111100011111111110000000000000000000000000111111000111111111
111111000000011111111110000000000
> P03105 VL2_HPV1A

MYRLRRKRAAPKDIYPSCKISNTCPPDIQNKIEHTTIADKILQYGSLGVFLGGLGIGTARGSGGRI
GYTPLGEGGGVRVATRPTPVRPTIPVETVGPSEIFPIDVVDPTGPAVIPLQDLGRDFPIPTVQVIA
EIHPISDIPNIVASSTNEGESAILDVLRGNATIRTVSRTQYNNPSFTVASTSNISAGEASTSDIVF
VSNGSGDRVVGEDIPLVELNLGLETDTSSVVQETAFSSSTPIAERPSFRPSRFYNRRLYEQVQVQD
PRFVEQPQSMVTFDNPAFEPELDEVSIIFQRDLDALAQTPVPEFRDVVYLSKPTFSREPGGRLRVS
RLGKSSTIRTRLGTAIGARTHFFYDLSSIAPEDSIELLPLGEHSQTTVISSNLGDTAFIQGETAED
DLEVISLETPQLYSEEELLDTNESVGENLQLTITNSEGEVSILDLTQSRVRPPFGTEDTSLHVYYP
NSSKGTPIINPEESFTPLVIIALNNSTGDFELHPSLRKRRKRAYV
00000000000000000000000000000000000000000000000000000000000000000
00000000000000000000000000000000001111111000000000000000000000000
00000000000000000000000000000000000000000000000000000000000000000
00000000000000000000000000000000000000000000000000000000000000000
00000000000000000000000000000000000000000000000000000000000000000
00000000000000000000000000000000000000000000000000000000000000000
00000000000000000000000000000000000000000000000000000000000000000
000000000000000000000000000000000000000000000
> P23700 OM6_CHLPN
MSKLIRRVVTVLALTSMASCFASGGIEAAVAESLITKIVASAETKPAPVPMTAKKVRLVRRNKQPV
EQKSRGAFCDKEFYPCEEGRCQPVEAQQESCYGRLYSVKVNDDCNVEICQSVPEYATVGSPYPIEI
LAIGKKDCVDVVITQQLPCEAEFVSSDPETTPTSDGKLVWKIDRLGAGDKCKITVWVKPLKEGCCF
TAATVCACPELRSYTKCGQPAICIKQEGPDCACLRCPVCYKIEVVNTGSAIARNVTVDNPVPDGYS
HASGQRVLSFNLGDMRPGDKKVFTVEFCPQRRGQITNVATVTYCGGHKCSANVTTVVNEPCVQVNI
SGADWSYVCKPVEYSISVSNPGDLVLHDVVIQDTLPSGVTVLEAPGGEICCNKVVWRIKEMCPGET
LQFKLVVKAQVPGRFTNQVAVTSESNCGTCTSCAETTTHWKGLAATHMCVLDTNDPICVGENTVYR
ICVTNRGSAEDTNVSLILKFSKELQPIASSGPTKGTISGNTVVFDALPKLGSKESVEFSVTLKGIA
PGDARGEAILSSDTLTSPVSDTENTHVY
00000000000000000000011111111111111111111100111111111111111111111
11111111110000001111111111111100000000000000000000000000000000000
00000000000000000000000000000000000000000000000000000000000000000
00000000000000000000000000000000000000000000000000000000000000000
00000000000000000000000000000000000000000000000000000000000000000
00000000000000000000000000000000000000000000000000000000000000000
00000000000000000000000000000000000000000000000000000000000000000
00000000000000000000000000000000000000000000000000000000000000000
0000000000000000000000000000
> P02977 M5_STRP5
MARENTNKHYWLRKLKKGTASVAVALSVLGAGLVVNTNEVSAAVTRGTINDPQRAKEALDKYELEN
HDLKTKNEGLKTENEGLKTENEGLKTENEGLKTEKKEHEAENDKLKQQRDTLSTQKETLEREVQNT

QYNNETLKIKNGDLTKELNKTRQELANKQQESKENEKALNELLEKTVKDKIAKEQENKETIGTLKK
ILDETVKDKIAKEQENKETIGTLKKILDETVKDKLAKEQKSKQNIGALKQELAKKDEANKISDASR
KGLRRDLDASREAKKQLEAEHQKLEEQNKISEASRKGLRRDLDASREAKKQLEAEQQKLEEQNKIS
EASRKGLRRDLDASREAKKQVEKALEEANSKLAALEKLNKELEESKKLTEKEKAELQAKLEAEAKA
LKEQLAKQAEELAKLRAGKASDSQTPDTKPGNKAVPGKGQAPQAGTKPNQNKAPMKETKRQLPSTG
ETANPFFTAAALTVMATAGVAAVVKRKEEN
000000000000000000000000000000000000000000000000000000000000000000
000000000000000000000000000000000000000000000000000000000000000000
000000000000000000000000000000000000000000000000000000000000000000
000000000000000000000000000000000000000000000000000000000000000000
111111111111111100000000000000011111111111111111100000000000000000
000000000000000000000000000000000000000000000000000000000000000000
000000000000000000000000000000000000000000000000000000000000000000
000000000000000000000000000000
> P08342 VL2_BPV4
MVRAARRKRASEDDLYRGCRMGQDCPIDIKNKYEHNTLADRILKWVSSFLYFGQLGISSGKGTGGS
TGYTPLGGRGGGGVTSGKGANVVRPTVIVDALGPTGVPIDPAVPDSSIVPLLESSGGSTTLDATPG
AEIEIIAEVHPPPVYEGPEVTIGDIEEPPILEVVPETHPTSRVRSTTSKHDNPAFTAYVASAQLPG
ETSASDNVYILHGFNGDFVGQADPEGDTIFEEIPLEEFGVPDMPPSTSTPTSSFRSVLNKFQRRLY
NRKLVQQVKITNRNTFLKQPSQFVQWEFDNPAYVDDSLSLIFQQDLDEVSAAPDADFQDIVKLSRP
VFTTKEGLVRLSRLGQRGTIKTRMACK
000000000000000000001111111111111111111100000000000000000000000000
000000000000001111111111111111111111111111111111111111000000000011
111111111111111111111111111111111111110000000000000000000000111111
111111111000000000000000000000000000000000000000000000000000000000
000000000000000000000000000000000000000000000000000000000000000000
000000000000000000000000000
> P23421 OM1B_CHLTR
MKKLLKSVLVFAALSSASSLQALPVGNPAEPSLMIDGILWEGFGGDPCDPCTTWVDAISMRMGYYG
DFVFDRVLKTDVNKEFQMGAKPTTTTGNAVAPSTLTARENPAYGRHMQDAEMFTNAACMALNIWDR
FDVFCTLGASSGYLKGNSASFNLVGLFGNNENQTKVSNGAFVPNMSLDQSVVELYTDTAFAWSVGA
RAALWECGCATLGASFQYAQSKPKVEELNVLCNAAEFTINKPKGYVGKELPLDLTAGTDAATGTKD
ASIDYHEWQASLALSYRLNMFTPYIGVKWSRASFDADTIRIAQPKSAETIFDVTTLNPTIAGAGDV
KTSAEGQLGDTMQIVSLQLNKMKSRKSCGIAVGTTIVDADKYAVTVETRLIDERAAHVNAQFRF
000000000000000000000000000000000000000000000000000000000000000000
000000000000000000000001111111111000000000000000000000000000000000
000001111111111110000000011111111111111111111000000000000000000000
000000000000000000000000000000000000011111111111100000000000000000

00000000000000000000000000000000111111111111011111111111111111111
0000000000000000000000000000000000000000000000000000000000000000
> P05889 GAG_HV1W2
GARASVLSGGELDKWEKIRLRPGGKKKYRLKHIVWASRELERFAVNPGLLETSEGCRQILGQLQPS
LQTGSEELRSLYNTVATLYCVHQRIEKKDTKEALDKIEEEQNKCKKKAQQAAADTGNSSQVSQNYP
IVQNLQGQMVHQAISPRTLNAWVKVVEEKAFSPEVIPMFSALSEGATPQDLNTMLNTVGGHQAAMQ
MLKETINEEAAEWDRLHPVHAGPIAPGQMREPRGSDIAGTTSTLQEQIGWMTNNPPIPVGEIYKRW
IILGLNKIVRMYSPTSILDIRQGPKEPFRDYVDRFYKTLRAEQATQEVKNWMTETLLVQNANPDCK
TILKALGPAATLEEMMTACQGVGGPGHKARVLAEAMSQVTNPTTIMMQKGNFRNQRKT
000000000000000000000000000000000000000000000000000000000000000000
000000000000000000000000000000000000000000000000000000000000000000
000000000000000000000000000000000000000000001111111111111100000000
000000000000000000000000000000000000000000000000000000000000000000
000000000000000000000000000000000000000000000000000000000000000000
0000000000000000000000000000000000000000000000000000000000
> P00433 PER1A_ARMRU
MHFSSSSTLFTCITLIPLVCLILHASLSDAQLTPTFYDNSCPNVSNIVRDTIVNELRSDPRIAASI
LRLHFHDCFVNGCDASILLDNTTSFRTEKDAFGNANSARGFPVIDRMKAAVESACPRTVSCADLLT
IAAQQSVTLAGGPSWRVPLGRRDSLQAFLDLANANLPAPFFTLPQLKDSFRNVGLNRSSDLVALSG
GHTFGKNQCRFIMDRLYNFSNTGLPDPTLNTTYLQTLRGLCPLNGNLSALVDFDLRTPTIFDNKYY
VNLEEQKGLIQSDQELFSSPNATDTIPLVRSFANSTQTFFNAFVEAMDRMGNITPLTGTQGQIRLN
CRVVNSNSLLHDMVEVVDFVSSM
000000000000000000000000000000000000000000011111111110011111101111
110000000000001111110000000000000000000000000000000000000000000111
111111000011111110000000000000000001111111111000000000000000000000
000000000000000000000000000011111100000000000000000000000000000000
000000000000000000000000000111111111100000000000000000000000000000
01111111000000000000000
> P05193 AMPC_CITFR
MMKKSICCALLLTASFSTFAAAKTEQQIADIVNRTITPLMQEQAIPGMAVAIIYEGKPYYFTWGKA
DIANNHPVTQQTLFELGSVSKTFNGVLGGDRIARGEIKLSDPVTKYWPELTGKQWRGISLLHLATY
TAGGLPLQIPGDVTDKAELLRFYQNWQPQWTPGAKRLYANSSIGLFGALAVKSSGMSYEEAMTRRV
LQPLKLAHTWITVPQSEQKNYAWGYLEGKPVHVSPGQLDAEAYGVKSSVIDMARWVQANMDASHVQ
EKTLQQGIELAQSRYWRIGDMYQGLGWEMLNWPLKADSIINGSDSKVALAALPAVEVNPPAPAVKA
SWVHKTGSTGGFGSYVAFVPEKNLGIVMLANKSYPNPARVEAAWRILEKLQ
000000000000000000001111111111111111111111000000000000000000000000
000000000000000000000000000000000000000000000000000000000000000000
000000000000000000000000000000000000000000000000000000000000000000

00000000000000000000000000000111111111111111111000000000000000000
00000000000000000000000000000000000000001111111111111111000000000
00000000111111111111111111111100000000000000000000
> P06597 OM1M_CHLTR
MKKLLKSVLVFAALSSASSLQALPVGNPAEPSLMIDGILWEGFGGDPCDPCTTWCDAISMRMGYYG
DFVFDRVLQTDVNKEFQMGAKPTTATGNAAAPSTCTARENPAYGRHMQDAEMFTNAAYMALNIWDR
FDVFCTLGATSGYLKGNSASFNLVGLFGDNENHATVSDSKLVPNMSLDQSVVELYTDTTFAWSAGA
RAALWECGCATLGASFQYAQSKPKVEELNVLCNAAEFTINKPKGYVGQEFPLDLKAGTDGVTGTKD
ASIDYHEWQASLALSYRLNMFTPYIGVKWSRASFDADTIRIAQPKSATTVFDVTTLNPTIAGAGDV
KASAEGQLGDTMQIVSLQLNKMKSRKSCGIAVGTTIVDADKYAVTVETRLIDERAAHVNAQFRF
00000000000000000000000000000000000000000000000000000000000000000
00000000000000000000000000000000000000000000000000000000000000000
00000000000000000000000000001111111111111000000000000000000000000
00000000000000000000000000000000000000000000000000000000000000000
00000000000000000000000000000000000000000000111111111111111111111
111111111000000000000000000000000000000000000000000000000000000
> P28871 CARP2_CANAL
MFLKNIFIALAIALLVDATPTTTKRSAGFVALDFSVVKTPKAFPVTNGQEGKTSKRQAVPVTLHNE
QVTYAADITVGSNNQKLNVIVDTGSSDLWVPDVNVDCQVTYSDQTADFCKQKGTYDPSGSSASQDL
NTPFKIGYGDGSSSQGTLYKDTVGFGGVSIKNQVLADVDSTSIDQGILGVGYKTNEAGGSYDNVPV
TLKKQGVIAKNAYSLYLNSPDAATGQIIFGGVDNAKYSGSLIALPVTSDRELRISLGSVEVSGKTI
NTDNVDVLLDSGTTITYLQQDLADQIIKAFNGKLTQDSNGNSFYEVDCNLSGDVVFNFSKNAKISV
PASEFAASLQGDDGQPYDKCQLLFDVNDANILGDNFLRSAYIVYDLDDNEISLAQVKYTSASSISA
LT
00000000000000000000000000000000000000000000000000011111111111110
00000000111111111111000000000000000000000000000000000000000000000
00000000001111111111111111111111110000000000000000000000000000000
00000000000000000000000000000000000000000000000000000000000000000
00000000000000000000000000000000000000000000000000000000000000000
00000000000011111111111100000000000000000000000000111111111111100
00
> P17451 OM1E_CHLTR
MKKLLKSVLVFAALSSASSLQALPVGNPAEPSLMIDGILWEGFGGDPCDPCTTWCDAISMRMGYYG
DFVFDRVLKTDVNKEFQMGDKPTSTTGNATAPTTLTARENPAYGRHMQDAEMFTNAACMALNIWDR
FDVFCTLGASSGYLKGNSASFNLVGLFGDNENQSTVKTNSVPNMSLDQSVVELYTDTAFSWSVGAR
AALWECGCATLGASFQYAQSKPKVEELNVLCNAAEFTINKPKGYVGQEFPLALIAGTDAATGTKDA
SIDYHEWQASLALSYRLNMFTPYIGVKWSRASFDADTIRIAQPKSATAIFDTTTLNPTIAGAGDVK
ASAEGQLGDTMQIVSLQLNKMKSRKSCGIAVGTTIVDADKYAVTVETRLIDERAAHVNAQFRF

000000000000000000000000000000000000000000000000000000000000000000
000000000000000000000000000000000000000000000000000000000000000000
000000000000000000000000000000111111111111100000000000000000000000
000000000000000000000000000000000000000000000000000000000000000000
000000000000000000000000000000000000000000001111111111111111100000
000000000000000000000000000000000000000000000000000000000000000
> P03275 FIBP_ADE02
MKRARPSEDTFNPVYPYDTETGPPTVPFLTPPFVSPNGFQESPPGVLSLRVSEPLDTSHGMLALKM
GSGLTLDKAGNLTSQNVTTVTQPLKKTKSNISLDTSAPLTITSGALTVATTAPLIVTSGALSVQSQ
APLTVQDSKLSIATKGPITVSDGKLALQTSAPLSGSDSDTLTVTASPPLTTATGSLGINMEDPIYV
NNGKIGIKISGPLQVAQNSDTLTVVTGPGVTVEQNSLRTKVAGAIGYDSSNNMEIKTGGGMRINNN
LLILDVDYPFDAQTKLRLKLGQGPLYINASHNLDINYNRGLYLFNASNNTKKLEVSIKKSSGLNFD
NTAIAINAGKGLEFDTNTSESPDINPIKTKIGSGIDYNENGAMITKLGAGLSFDNSGAITIGNKND
DKLTLWTTPDPSPNCRIHSDNDCKFTLVLTKCGSQVLATVAALAVSGDLSSMTGTVASVSIFLRFD
QNGVLMENSSLKKHYWNFRNGNSTNANPYTNAVGFMPNLLAYPKTQSQTAKNNIVSQVYLHGDKTK
PMILTITLNGTSESTETSEVSTYSMSFTWSWESGKYTTETFATNSYTFSYIAQE
000000000000000000000000000000000000000000000000000000000000000000
000000000000000000000000000000000000000000000000000000000000000000
000000000000000000000000000000000000000000000000000000000000000000
000000000000000000000000000000000000000000000000000000000000000000
000000000000000000000000000000000000000000000000000000000000000000
000000000000000000000000000000000000000000000000000000000000000000
000000000000001111111111000000000000111111110000111111110000001111
111111110000000000000000001111111111100000000000000011111111110000
000000111111111111111111111111000000000000111111111100000
> P06727 APOA4_HUMAN
MFLKAVVLTLALVAVAGARAEVSADQVATVMWDYFSQLSNNAKEAVEHLQKSELTQQLNALFQDKL
GEVNTYAGDLQKKLVPFATELHERLAKDSEKLKEEIGKELEELRARLLPHANEVSQKIGDNLRELQ
QRLEPYADQLRTQVNTQAEQLRRQLTPYAQRMERVLRENADSLQASLRPHADELKAKIDQNVEELK
GRLTPYADEFKVKIDQTVEELRRSLAPYAQDTQEKLNHQLEGLTFQMKKNAEELKARISASAEELR
QRLAPLAEDVRGNLKGNTEGLQKSLAELGGHLDQQVEEFRRRVEPYGENFNKALVQQMEQLRQKLG
PHAGDVEGHLSFLEKDLRDKVNSFFSTFKEKESQDKTLSLPELEQQQEQQQEQQQEQVQMLAPLES
000000000000000000000000000000000000000000000000000000000000000000
000000000000000000000000000000000000000000000000000000000000000000
000000000000000000000000000000000000000000000000000000000000000000
000000000000000000000000000000000000000000000000000000000011111111
111111000000000000000000000000000000000000000000000000000000000000
000000000000000000000000000000000000000000000000000000000000000000

> P20287 G3P_SCHMA

MSRAKVGINGFGRIGRLVLRAAFLKNTVDVVSVNDPFIDLEYMVYMIKRDSTHGTFPGEVSTENGK
LKVNGKLISVHCERDPANIPWDKDGAEYVVESTGVFTTIDKAQAHIKNNRAKKVIISAPSADAPMF
VVGVNENSYEKSMSVVSNASCTTNCLAPLAKVIHDKFEIVEGLMTTVHSFTATQKVVDGPSSKLWR
DGRGAMQNIIPASTGAAKAVGKVIPALNGKLTGMAFRVPTPDVSVVDLTCRLGKGASYEEIKAAVK
AAASGPLKGILEYTEDEVVSSDFVGSTSSSIFDAKAGISLNNNFVKLVSWYDNEFGYSCRVVDLIT
HMHKVDHA
000000000000000000000000000000111111111111000000000000000111111111
111111111111111100000000000000000111111111111111000000000000000000
000000000000000000000000000000111111111100000000000000000000000000
000000000000000000000000000000000000000000000000000000000000000000
000111111111111111111111100000000000000000000000000000000000000000
00000000

> P14283 PERT_BORPE

MNMSLSRIVKAAPLRRTTLAMALGALGAAPAAHADWNNQSIVKTGERQHGIHIQGSDPGGVRTASG
TTIKVSGRQAQGILLENPAAELQFRNGSVTSSGQLSDDGIRRFLGTVTVKAGKLVADHATLANVGD
TWDDDGIALYVAGEQAQASIADSTLQGAGGVQIERGANVTVQRSAIVDGGLHIGALQSLQPEDLPP
SRVVLRDTNVTAVPASGAPAAVSVLGASELTLDGGHITGGRAAGVAAMQGAVVHLQRATIRRGDAP
AGGAVPGGAVPGGAVPGGFGPGGFGPVLDGWYGVDVSGSSVELAQSIVEAPELGAAIRVGRGARVT
VSGGSLSAPHGNVIETGGARRFAPQAAPLSITLQAGAHAQGKALLYRVLPEPVKLTLTGGADAQGD
IVATELPSIPGTSIGPLDVALASQARWTGATRAVDSLSIDNATWVMTDNSNVGALRLASDGSVDFQ
QPAEAGRFKVLTVNTLAGSGLFRMNVFADLGLSDKLVVMQDASGQHRLWVRNSGSEPASANTLLLV
QTPLGSAATFTLANKDGKVDIGTYRYRLAANGNGQWSLVGAKAPPAPKPAPQPGPQPPQPPQPQPE
APAPQPPAGRELSAAANAAVNTGGVGLASTLWYAESNALSKRLGELRLNPDAGGAWGRGFAQRQQL
DNRAGRRFDQKVAGFELGADHAVAVAGGRWHLGGLAGYTRGDRGFTGDGGGHTDSVHVGGYATYIA
DSGFYLDATLRASRLENDFKVAGSDGYAVKGKYRTHGVGASLEAGRRFTHADGWFLEPQAELAVFR
AGGGAYRAANGLRVRDEGGSSVLGRLGLEVGKRIELAGGRQVQPYIKASVLQEFDGAGTVHTNGIA
HRTELRGTRAELGLGMAAALGRGHSLYASYEYSKGPKLAMPWTFHAGYRYSW
000000000000000000000000000000000000000000000000000000000000000000
000000000000000000000000000000000000000000000000000000000000000000
000000000000000000000000000000000000000000000000000000000000000000
000000000000000000000000000000000000000000000000000000000111111111
111111111111111111111111111111111111000000000000000000000000000000
000000000000000000000000000000000000000000000000000000000000000000
000000000000000000000000000000000000000000000000000000000000000000
000000000000000000000000000000000000000000000000000000000000000000
000000000000000000000000000000000000000000000000000000000000000000
000000000000000000000000000000000000000000000000000000000000000000

000000000000000000000000000000000000000000000000000000000000000000
000000000000000000000000000000000000000000000000000000000000000000
000000000000000000000000000000000000000000000000000000000000000000
0000000000000000000000000000000000000000000000000000
> ENV_SIVVG ENV_SIVVG
MKLTLLIGILLIGIGVVLNTRQQWVTVFYGVPVWKNSSVQAFCMTPTTRLWATTNSIPDDHDYTEV
PLNITEPFEAWADRNPLVAQAGSNIHLLFEQTLKPCVKLSPLCIKMSCVELNSSEPTTTPKSTTAS
TTNITASTTTLPCVQNKTSTVLESCNETIIEKELNEEPASNCTFAMAGYVRDQKKKYSVVWNDAEI
MCKKGNNSNRECYMIHCNDSVIKEACDKTYWDELRLRYCAPAGFALLKCNDYDYAGFKTNCSNVSV
VHCTNLINTTVTTGLLLNGSYSENRTQIWQKHRVSNDSVLVLFNKHYNLTVTCKRPGNKTVLPVTI
MAGLVFHSQRYNTRLRQAWCHFQGNWRGAWKEVKNEIVKLPKDRYQGTNDTEEIYLQRLFGDPEAA
NLWFNCQGEFFYCKMDWFLNYLNNRTVDPDHNPCNGTKGKGKAPGPCAQRTYVACHIRSVINDWYT
LSRKTYAPPREGHLQCTSTVTGMSVELNYNSKNRTNVTLSPQIETIWAAELGRYKLVEITPIGFAP
TEVRRYTGGHDRTKRVPFVLGFLGFLGAAGTAMGAAATALTVQSQHLLAGILQQQKNLLAVEAQQ
QMLKLTIWGVKNLNARVTALEKYLEDQARLNAWGCAWKQVCHTTVPWQWNNRTPDWNNMTWLEWER
QISYLEGNITTQLEEARAQEEKNLDAYQKLSSWSDFWSWFDFSKWLNILKIGFLDVLGIIGLRLLY
TVYSCIARVRQGYSPLSPQIHIHPWKGQPDNAEGPGEGGDKRKNSSEPWQKESGTAEWKSNWCKRL
TNWCSISSIWLYNSCLTLLVHLRSAFQYIQYGLGELKAAAQEAVVALARLAQNAGYQIWLACRSAY
RAIINSPRRVRQGLEGILN
000000000000000000000000000000000000000000000000000000000000000000
000000000000000000000000111111111111111000000000000000000000000000
000000000000000000000000000000000000000000000000000000000000000000
000000000000000000000000000000000000000000000000000000000000000000
000000000000000000000000001111111111111110000000000111111111111111
111111111100000000000000000000000000000000000000000000000000000000
000000000000001111111111111110000000000000000000000000000000000000
000000000000000000000000000000000000000000000000000000000011111111
111111111111111110000000000000000000000000000000000000000000000000
000000000000000000000111111111111111111111111100000111111111111111
111110000000000000000000000000111111111111111000000000000000000000
000000000000000000000000000000000000000000000000000000000000000000
000000000000000000000000000000000000000000000000000001111111111111
1111111000000000000
> P06830 HEMA_MEASH
MSPQRDRINAFYKDNPHPKGSRIVINREHLMIDRPYVLLAVLFVMFLSLIGLLAIAGIRLHRAAIY
TAEIHKSLSTNLDVTNSIEHQVKDVLTPLFKIIGDEVGLRTPQRFTDLVKFISDKIKFLNPDREYD
FRDLTWCINPPERIKLDYDQYCADVAAEELMNALVNSTLLETRTTNQFLAVSKGNCSGPTTIRGQF
SNMSLSLLDLYLGRGYNVSSIVTMTSQGMYGGTYPVEKPNLSSKRSELSQLSMYRVFEVSVIRNPG

LGAPVFHMTNYLEQPVSNDLSNCMVALGELKLAALCHGEDSITIPYQGSGKGVSFQLVKLGVWKSP
TGMQSWVPLSTDDPVIDRLYLSSHRGVIADNQAKWAVPTTRTDDKLRMETCFQQACKGKIQALCEN
PECVPLKDNRIPSYGVLSVDLSLTVELKIKIASGFGPLITHGSGMDLYKSNHNNVYWLTIPPMKNL
ALGVINTLEWIPRFKVSPYLFTVPIKEAGEDCHAPTYLPAEVDGDVKLSSNLVILPGQDLQYVLAT
YDTSRVEHAVVYYVYSPGRSFSYFYPFRLPIKGVPIELQVECFTWDQKLWCRHFCVLADSESGGHI
THSGMVGMGVSCTVTREDGTNRR
000000000000000000000000000000000000000000000000111111111111111111
111111000000000000000000000000000000111111111111111000001111111111
111110000000000000000000000000000000000000000000001111111111111110
000000000000000000000000000000000000000000001111111111111110000000
000000000000000000000000000011111111111111100000000000000000000000
000000000000000000000000000000000000000000000000000000000000000000
000000000000000000000000000000000000000000000000000000000000000000
111111111111111000000000000000000000000000000000000000000000000000
000000000000000000000000000000000011111111111111111111111110000000
00000000000000000000000
> P11193 VP4_ROTHW
MASLIYRQLLTNSYSVDLHDEIEQIGSEKTQNVTINPSPFAQTRYAPVNWGHGEINDSTTVEPILD
GPYQPTTFTPPNDYWILINSNTNGVVYESTNNSDFWTAVVAIEPHVNPVDRQYLIFGESKQFNVSN
DSNKWKFLEMFRSSSQNEFYNRRTLTSDTRFVGILKYGGRVWTFHGETPRATTDSSSTANLNNISI
TIHSEFYIIPRSQESKCNEYINNGLPPIQNTRNVVPLPLSSRSIQYKRAQVNEDIIVSKTSLWKEM
QYNRDIIIRFKFGNSIVKMGGLGYKWSEISYKAANYQYNYLRDGEQVTAHTTCSVNGVNNFSYNGG
SLPTDFGISRYEVIKENSYVYVDYWDDSKAFRNMVYYVRSLAANLNSVKCTGGSYNFSIPVGAWPVM
NGGAVSLHFAGVTLSTQFTDFVSLNSLRFRFSLTVDEPPFSILRTRTVNLYGLPAANPNNGNEYYE
ISGRFSLIYLVPTNDDYQTPIMNSVTVRQDLERQLTDLREEFNSLSQEIAMAQLIDLALLPLDMFS
MFSGIKSTIDLTKSMATSVMKKFRKSKLATSISEMTNSLSDAASSASRNVSIRSNLSAISNWTNVS
NDVSNVTNSLNDISTQTSTISKKLRLKEMITQTEGMSFDDISAAVLKTKIDMSTQIGKNTLPDIVT
EASEKFIPKRSYRILKDDEVMEINTEGKFFAYKINTFDEVPFDVNKFAELVTDSPVISAIIDFKTL
KNLNDNYGITRTEALNLIKSNPKMLRNFINQNHPIIRNRIEQLILQCRL
111111111100000000000000000000000011111111100000000000111111111111
000000000000000000000000000000000000000000000000000000000000000000
000000000000000000000000000000000000000000000000000000000000000000
000000000000000000000000111111111111000000000000000000000000000000
000000000000000000000000000000000000000000000000000000000000000000
000000000000000000000000000000000000000000000000000000000000000000
000000000000000000000000000000000000000000000000000000000000000000
000000000000000000000000000000000000000000000000000000000000000000
000000000000000000000000000000000000000000000000000000000000000000

000000000000000000000000000000000000000000000000000000000000000000
000000000000000000000000000000000000000000000000000000000000000000
0000000000000000000000000000000000000000000000000
> P09259 VGLE_VZVD
MGTVNKPVVGVLMGFGIITGTLRITNPVRASVLRYDDFHTDEDKLDTNSVYEPYYHSDHAESSWVN
RGESSRKAYDHNSPYIWPRNDYDGFLENAHEHHGVYNQGRGIDSGERLMQPTQMSAQEDLGDDTGI
HVIPTLNGDDRHKIVNVDQRQYGDVFKGDLNPKPQGQRLIEVSVEENHPFTLRAPIQRIYGVRYTE
TWSFLPSLTCTGDAAPAIQHICLKHTTCFQDVVVDVDCAENTKEDQLAEISYRFQGKKEADQPWIV
VNTSTLFDELELDPPEIEPGVLKVLRTEKQYLGVYIWNMRGSDGTSTYATFLVTWKGDEKTRNPTP
AVTPQPRGAEFHMWNYHSHVFSVGDTFSLAMHLQYKIHEAPFDLLLEWLYVPIDPTCQPMRLYSTC
LYHPNAPQCLSHMNSGCTFTSPHLAQRVASTVYQNCEHADNYTAYCLGISHMEPSFGLILHDGGTT
LKFVDTPESLSGLYVFVVYFNGHVEAVAYTVVSTVDHFVNAIEERGFPPTAGQPPATTKPKEITPV
NPGTSPLLRYAAWTGGLAAVVLLCLVIFLICTAKRMRVKAYRVDKSPYNQSMYYAGLPVDDFEDSE
STDTEEEFGNAIGGSHGGSSYTVYIDKTR
0000000000000000000000000000000000000000000000000000000Z11111111111
111111111111111111111111111111111111111000000000011111111111111111
111000000000000000000000000000000000000000000000000000000000000000
000000000000000000000000000000000000000000000000000000000000000000
000000000000000000000000000000000000000000000000000000000000000000
000000000000000000000000000000000000000000000000000000000000000000
000000000000000000000000000000000000000000000000000000000000000000
000000000000000000000000000000000000000000000000000000000000000000
000000000000000000000000000000000000000000000000000000000000000000
00000000000000000000000000000
> P41355 HEMA_RINDR
MSPPRDRVDAYYKDNFQFKNTRVVLNKEQLLIERPCMLLTVLFVMFLSLVGLLAIAGIRLHRAAVN
TAKINNDLTTSIDITKSIEYQVKDVLTPLFKIIGDEVGLRTPQRFTDLTKFISDKIKFLNPDKEYD
FRDINWCINPPERIKIDYDQYCAHTAAEDLITMLVNSSLTGTTVPRTSLVNLGRNCTGPTTTKGQF
SNISLTLSGIYSGRGYNISSMITITGKGMYGSTYLVGKYNQRARRPSKVWHQDYRVFEVGIIRELG
VGTPGFHMTNYLELPRQPELETCMLALGESKLAALCLADSPVALHYGRVGDDNKIRFVKLGVWASP
ADRDTLATLSAIDPTLDGLYITTHRGIIAAGTAIWAVPVTRTDDQVKMGKCRLEACRDRPPPFCNS
TDWEPLEAGRIPAYGVLTIKLGLADEPKVDIISEFGPLITHDSGMDLYTSFDGTKYWLTTPPLQNS
ALGTVNTLVLEPSLKISPNILTLPIRSGGGDCYIPTYLSDRADDDVKLSSNLVILPSRDLQYVSAT
YDISRVEHAIVYHIYSTGRLSSYYYPFKLPIKGDPVSLQIECFPWDRKLWCHHFCSVVDSGTGEQV
THIGVVGIKITCNGK
000000000000000000000000000000000000000000000000000000000000000000
000000000000000000000000000000000000000000000000000000000000000000
000000000000000000000000000000000000000000000000000000000000000000

000000000000000000000000000000000000000000000000000000000000000000
000000000000000000000000000000000000000000000000000000000000000000
000000000000000000000000000000000000000000000000000000000000000000
000000000000000000000000000000000000000000000000000000000000000000
000000000000000000000000000000000000000000000000000000000000000000
0000000000000000000000000000000000000000000000111111111000000000000
000000000000000
> P31627 ENV_CAEVG
MDAGASYMRLTGEENWVEVTMDEEKERKGKDVQQGKYRPQVSKPIINRDTNTSFAYKGIFLWGIQI
TMWILLWTNMCVRAEDYITLISDPYGFSPIKNVSGVPVTCVTKEFARWGCQPLGAYPDPEIEYRNV
SQEIVKEVYQENWPWNTYHWPLWQMENVRYWLKENIAENKKRKNSTKKGIEELLAGTIRGRFCVPY
PFALLKCTKWCWYPAEIDQETGRARKIKINCTEARAVSCTEEMPLASIHRAYWDEKDRESMAFMNI
RACDSNLRCQKRPGGCVEGYPIPVGANIIPENMKYLRGQKSQYGGIKDKNGELKLPLTVRVWVKLA
NVSTWVNGTPPYWQNRINGSKGINGTLWGQLSGMHHLGFNLSQTGKWCNYTGKIKIGQETFSYHYK
PNWNCTGNWTQHPVWQVMRDLDMVEHMTGECVQRPQRHNITVDRNQTITGNCSVTNWDGCNCSRSG
NYLYNSTTGGLLVIICRNNNTITGIMGTNTNWTTMWRIYRNCSGCENATLDRKETGTLGGVANKNC
SLPHKNESNKWTCAPRQREGKTDSLYIAGGKKFWTREKAQYSCENNIGELDGMLHQQILLQKYQVI
KVRAYTYGVIEMPENYAKTRIINRRKRELSHTRKKRGVGLVIMLVIMAIVAAAGASLGVANAIQQS
YTKAAVQTLANATAAQQDALEATYAMVQHVAKGVRILEARVARVEAITDRIMLYQELDCWHYHQYC
VTSTRADVAKYINWTRFKDNCTWQQWERELQGYDGNLTMLLRESARQTQLAEEQVRRIPDVWESLK
EVFDWSGWFSWLKYIPIIVVGLVGCILIRAVICVCQPLVQIYRTLSTPTYQRVTVIMEKRADVAGE
NQDFGDGLEESDDSKTDQKVTVQKAWSRAWELWQNSPWKEPWKRSLLKLLILPLTMGIWINGRLGE
HLKNKKERVDCETWGKGD
000000000000000000000000000000000000000000000000000000000000000000
000000000000000000000000000000000000000000000000011111111111111000
000000000000000000000000000000000000000000000000000000000000000000
000000000000000000000000000000000000000000000000000000000000000000
000000000000000000000000000000000000000000000000000000000000000000
000000000000000000000000000000000000000000000000000000000000000000
000000000000000000000000000000000000000000000000000000000000000000
000000000000000000000000000000000000000000000000000000000000000000
000000000000000000000000000000000000000000000000000000000000000000
000000000000000000000000000000000000000000000000000000000000000000
000000000000000000000000000000000000000000000000000000000000000000
000000000000000000000000000000000000000000000000000000000000000000
000000000000000000000000000000000000000000000000000000000000000000
000000000000000000000000000000000000000000000000000000000000000000
000000000000000000000000000000000000000000000000000000000000000000
000000000000000000

> P17821 DNAK_CHLTR

SEKRKSNKIIGIDLGTTNSCVSVMEGGQPKVIASSEGTRTTPSIVAFKGGETLVGIPAKRQAVTNP
EKTLASTKRFIGRKFSEVESEIKTVPYKVAPNSKGDAVFDVEQKLYTPEEIGAQILMKMKETAEAY
LGETVTEAVITVPAYFNDSQRASTKDAGRIAGLDVKRIIPEPTAAALAYGIDKEGDKKIAVFDLGG
GTFDISILEIGDGVFEVLSTNGDTHLGGDDFDGVIINWMLDEFKKQEGIDLSKDNMALQRLKDAAE
KAKIELSGVSSTEINQPFITIDANGPKHLALTLTRAQFEHLASSLIERTKQPCAQALKDAKLSASD
IDDVLLVGGMSRMPAVQAVVKEIFGKEPNKGVNPDEVVAIGAAIQGGVLGGEVKDVLLLDVIPLSL
GIETLGGVMTPLVERNTTIPTQKKQIFSTAADNQPAVTIVVLQGERPMAKDNKEIGRFDLTDIPPA
PRGHPQIEVTFDIDANGILHVSAKDAASGREQKIRIEASSGLKEDEIQQMIRDAELHKEEDKQRKE
ASDVKNEADGMIFRAEKAVKDYHDKIPAELVKEIEEHIEKVRQAIKEDASTTAIKAASDELSTHMQ
KIGEAMQAQSASAAASSAANAQGGPNINSEDLKKHSFSTRPPAGGSASSTDNIEDADVEIVDKPE
000000000000000000000000000000000000000000000000000000000000000000
000000000000000000000000000000000000000000000000000000000000000000
000000000000000000000000000000000000000000000000000000000000000000
000000000000000000000000000000000000000000000000000000000000000000
000000000000000000000000000000000000000000000000000000000000000000
000000000000000000000000000011111111111110000000000000000000000000
000000000000000000000000000000000000000000000000000000000000000000
000000000000000000000000000000000000000000000000000000000000000000
000000000000000000000000000000000000000000000000000000000000000000
00000000000000000000000000000000000000000000000000000000000000000

> P27404 COAT_FCVC6

MCSTCANVLKYYDWDPHIKLVINPNKFLHVGFCDNPLMCCYPELLPEFGTMWDCDQSPLQVYLESI
LGDDEWSSTHEAIDPVVPPMHWDEAGKIFQPHPGVLMHHLICKVAEGWDPNLPLFRLEADDGSITT
PEQGTMVGGVIAEPNAQMSTAADMATGKSVDSEWEAFFSFHTSVNWSTSETQGKILFKQSLGPLLN
PYLTHLAKLYVAWSGSVDVRFSISGSGVFGGKLAAIVVPPGIDPVQSTSMLQYPHVLFDARQVEPV
IFSIPDLRSTLYHLMSDTDTTSLVIMVYNDLINPYANDSNSSGCIVTVETKPGPDFKFHLLKPPGS
MLTHGSIPSDLIPKSSSLWIGNRFWSDITDFVIRPFVFQANRHFDFNQETAGWSTPRFRPITITIS
VKESAKLGIGVATDYIVPGIPDGWPDTTIPGELVPVGDYAITNGTNNDITTAAQYDAATEIRNNTN
FRGMYICGSLQRAWGDKKISNTAFITTGTVDGAKLIPSNTIDQTKIAVFQDTHANKHVQTSDDTLA
LLGYTGIGEEAIGADRDRVVRISVLPERGARGGNHPIFHKNSIKLGYVIRSIDVFNSQILHTSRQL
SLNHYLLSPDSFAVYRIIDSNGSWFDIGIDNDGFSFVGVSSIGKLEFPLTASYMGIQLAKIRLASN
IRSVMTKL

000000000000000000000000000000000000000000000000000000000000000000
000000000000000000000000000000000000000000000000000000000000000000
000000000000000000000000000000000000000000000000000000000000000000
000000000000000000000000000000000000000000000000000000000000000000
000000000000000000000000000000000000000000000000000000000000000000

000000000000000000000000000000000000000000000000000000000000000000
000000000000000011111111111000000000000000000000000000000000000000
000000001111111111111000000000000000000000000000000000000000000000
000000000000000000000000000000000000000000000000000000000000000000
000000000000000000000000000000000000000000000000000000000000000000
00000000
> P52501 COA1_PAVPK
MAPPAKRARGLTLPGYKYLGPGNSLDQGEPTNPSDAAAKEHDEAYDKYIKSGKNPYFYFSAADEKF
IKETEHAKDYGGKIGHYFFRAKRAFAPKLSETDSPTTSQQPEVRRSPRKHPGSKPPGKRPAPRHIF
INLAKKKAKGTSNTNSNSMSENVEQHNPINAGTELSATGNESGGGGGGGGGRGAGGVGVSTGSFNN
QTEFQYLGEGLVRITAHASRLIHLNMPEHETYKRIHVLNSESGVAGQMVQDDAHTQMVTPWSLIDA
NAWGVWFNPADWQLISNNMTEINLVSFEQEIFNVVLKTITESATSPPTKIYNNDLTASLMVALDTN
NTLPYTPAAPRSETLGFYPWLPTKPTQYRYYLSCTRNLNPPTYTGQSQQITDSIQTGLHSDIMFYT
IENAVPIHLLRTGDEFSTGIYHFDTKPLKLTHSWQTNRSLGLPPKLLTEPTTEGDQHPGTLPAANT
RKGYHQTINNSYTEATAIRPAQVGYNTPYMNFEYSNGGPFLTPIVPTADTQYNDDEPNGAIRFTMG
YQHGQLTTSSQELERYTFNPQSKCGRAPKQQFNQQAPLNLENTNNGTLLPSDPIGGKPNMHFMNTL
NTYGPLTALNNTAPVFPNGQIWDKELDTDLKPRLHVTAPFVCKNNPPGQLFVKIAPNLTDDFNADS
PQQPRIITYSNFWWKGTLTFTAKMRSSNMWNPIQQHTTTAENIGNYIPTNIGGIKMFPEYSQLIPR
KLY
000000000000000000000000000000000000000000000000000000000000000000
000000000000000000000000000000000000000000000000000000000000000000
000000000000111111111111111111111111000000000000000000000000000000
000000000000000000000000000000000000000000000000000000000000000000
000000000000000000000000000000000111111111111111000000000000000000
000000000000000000000000000000000000011111111111111111111111111000
000000000000000000000000000000000000000001111111111111111111111111
111111111111000000000000000000001111111111111111111111111111111110
000000000000000000000000000000000000001111111111111111111000000000
000000000000000000000000000000000000000000000000000000000000000000
000000000000000000000000000000000000000000000000000000000000000000
000
> P22303 ACES_HUMAN
MRPPQCLLHTPSLASPLLLLLLWLLGGGVGAEGREDAELLVTVRGGRLRGIRLKTPGGPVSAFLGI
PFAEPPMGPRRFLPPEPKQPWSGVVDATTFQSVCYQYVDTLYPGFEGTEMWNPNRELSEDCLYLNV
WTPYPRPTSPTPVLVWIYGGGFYSGASSLDVYDGRFLVQAERTVLVSMNYRVGAFGFLALPGSREA
PGNVGLLDQRLALQWVQENVAAFGGDPTSVTLFGESAGAASVGMHLLSPPSRGLFHRAVLQSGAPN
GPWATVGMGEARRRATQLAHLVGCPPGGTGGNDTELVACLRTRPAQVLVNHEWHVLPQESVFRFSF
VPVVDGDFLSDTPEALINAGDFHGLQVLVGVVKDEGSYFLVYGAPGFSKDNESLISRAEFLAGVRV

GVPQVSDLAAEAVVLHYTDWLHPEDPARLREALSDVVGDHNVVCPVAQLAGRLAAQGARVYAYVFE
HRASTLSWPLWMGVPHGYEIEFIFGIPLDPSRNYTAEEKIFAQRLMRYWANFARTGDPNEPRDPKA
PQWPPYTAGAQQYVSLDLRPLEVRRGLRAQACAFWNRFLPKLLSATDTLDEAERQWKAEFHRWSSY
MVHWKNQFDHYSKQDRCSDL
00000000000000000000000000000000000000000000000000000000000000000
00000000000000000000000000000000000000000000000000000000000000000
00000000111111111111000000000000000000001111111111000000000000000
00000111111111100000000000000000000000000000000000000000000000000
00000000001111111111110000000000000000000000000000000000001111111
11110000000000000000000000000000111111111100000000000000000000000
00000000000000000000000000000000000000000000000000001111111111111
11000000000000000000000000000000000000111111111111111000000001111
11111111000000000000000001111111111000000000000000000111111111111
11111111111000000000
> P13290 VGLG_HHV2H
MHAIAPRLLLLFVLSGLPGTRGGSGVPGPINPPNSDVVFPGGSPVAQYCYAYPRLDDPGPLGSADA
GRQDLPRRVVRHEPLGRSFLTGGLVLLAPPVRGFGAPNATYAARVTYYRLTRACRQPILLRQYGGC
RGGEPPSPKTCGSYTYTYQGGGPPTRYALVNASLLVPIWDRAAETFEYQIELGGELHVGLLWVEVG
GEGPGPTAPPQAARAEGGPCVPPVPAGRPWRSVPPVWYSAPNPGFRGLRFRERCLPPQTPAAPSDL
PRVAFAPQSLLVGITGRTFIRMARPTEDVGVLPPHWAPGALDDGPYAPFPPRPRFRRALRTDPEGV
DPDVRAPRTGRRLMALTEDTSSDSPTSAPEKTPLPVSATAMAPSVDPSAEPTAPATTTPPDEMATQ
AATVAVTPEETAVASPPATASVESSPLPAAAAATPGAGHTNTSSASAAKTPPTTPAPTTPPPTSTH
ATPRPTTPGPQTTPPGPATPGPVGASAAPTADSPLTASPPATAPGPSAANVSVAATTATPGTRGTA
RTPPTDPKTHPHGPADAPPGSPAPPPPEHRGGPEEFEGAGDGEPPEDDDSATGLAFRTPNPNKPPP
ARPGPIRPTLPPGILGPLAPNTPRPPAQAPAKDMPSGPTPQHIPLFWFLTASPALDILFIISTTIH
TAAFVCLVALAAQLWRGRAGRRRYAHPSVRYVCLPPERD
00000000000000000000000000000000000000000000000000000000000000000
00000000000000000000000000000000000000000000000000000000000000000
00000000000000000000000000000000000000000000000000000000000000000
00111111111111111000000000000000000000000000000000000000000000000
00000000000000000000000000000000000000000000001111111111111100000
00000000000000000000000000000000000000000000000000000000000000000
00000000000000000000000000000000000000000000000000000000000000000
00000000000000000000000000000000000000000000000000000000000001111
11111111111111111111111111111111111111111110111111111111111111110
00111111111111111111111111110000000000000000000000000000000000000
000000000000000000000000000000000000000
> P03211 EBN1_EBV

MSDEGPGTGPGNGLGEKGDTSGPEGSGGSGPQRRGGDNHGRGRGRGRGRGGGRPGAPGGSGSGPRH
RDGVRRPQKRPSCIGCKGTHGGTGAGAGAGGAGAGGAGAGGGAGAGGGAGGAGGAGGAGAGGGAGA
GGGAGGAGGAGAGGGAGAGGGAGGAGAGGGAGGAGGAGAGGGAGAGGGAGGAGAGGGAGGAGGAGA
GGGAGAGGAGGAGGAGAGGAGAGGGAGGAGGAGAGGAGAGGAGAGGAGAGGAGGAGAGGAGGAGAG
GAGGAGAGGGAGGAGAGGGAGGAGAGGAGGAGAGGAGGAGAGGAGGAGAGGGAGAGGAGAGGGGRG
RGGSGGRGRGGSGGRGRGGSGGRRGRGRERARGGSRERARGRGRGRGEKRPRSPSSQSSSSGSPPR
RPPPGRRPFFHPVGEADYFEYHQEGGPDGEPDVPPGAIEQGPADDPGEGPSTGPRGQGDGGRRKKG
GWFGKHRGQGGSNPKFENIAEGLRALLARSHVERTTDEGTWVAGVFVYGGSKTSLYNLRRGTALAI
PQCRLTPLSRLPFGMAPGPGPQPGPLRESIVCYFMVFLQTHIFAEVLKDAIKDLVMTKPAPTCNIR
VTVCSFDDGVDLPPWFPPMVEGAAAEGDDGDDGDEGGDGDEGEEGQE
000000000000000000000000000000000000000000000000000001111111111111
111111111111111111111111111111111110000000000000000000000000000000
000000000000000000000000000000000000000000000000000000000000000000
000000000000000000000000000000000000000000000000000000000000000000
000000000000000000000000000000000000000000000000000011111111111111
100000000000000000000000000000001111111111111111111111111111111111
111111111111111000000000000000000000000000000000000000000000000000
000000000000000000000000000000000000000011111111111111111100000000
000000000000000000000000000000000000000000000000000000000000000000
00000000000000000000000000000000000000000000000
> P28081 HEMA_MEASY
MSPQRDRTNAFYKDNPHPKGSRIVINREHLMIDRPYVLLAILFVMFLSLIGLLAIAGIRLHQAAIH
TAEIHKSLSTNLDVTNSIEHQVKDVLTPLFKIIGDEVGLRTPQRFTDLVKFISDKIKFLNPDREYD
FRDLNWCINPPERIKLDYDQYCADVAAEELMNALVNSTLLETRTTNQFLAVSKGNCSGPTTIRGQF
SNMSTSLLDLYLSRGYNVSSIVTMTSQGMYGGTYLVEKPNLSSKRSELSQLSMYRVFEVGVIRNPG
LGAPVFHMTNYFEQPVSNDLSNCMVALGEFKLAALCHREDSITIPYQGSGKGVSFQLVNLGVWKSP
TDMQSWIPLSTDDPVIDRLYLSSHRGVIADNQAKWAVPTTRTDDKLRMETCFQQACKGKIQALCEN
PEWAPLKDNRIPSYGVLSVDLSPTVELKIKIASGFGPLITHGSGMDLYKSNHNNVYWLTIPPMKNL
ALGVINTLEWIPRFKVSPNLFTVPIKEAGKDCHAPTYLPAEVDGDVKLSSNLVILPGQDLQYVLAT
YDTSRVEHAVVYYVYSPGRSFSYFYPFRLPIRGVPIELQVECFTWDQKLWCRHFCVLANSESGGHI
THSGMVGMGVSCTVTREDGTNRRQSC
000000000000000000000000000000000000000000000000000000000000000000
000000000000000000000000000000000000000000000000000000000000000000
000000000000000000000000000000000000000000000000000000000000000000
000000000000000000000000000000000000000000000000000000000000000000
000000000000000000000000000000000000000000000000000000000000000000
000000000000000000000000000000000000000000000000000011111111111111
111100000000000000000000000000000000000000000000000000000000000000

000000000000000000000000000000000000000000000000000000000000000000
000000000000000000000000000000000000000000000000000000000000000000
00000000000000000000000000
> P12930 COAT_PAVCN
MAPPAKRARRGKGVLVKWGEGKDLITMCFFIGLVPPGYKYLGPGNSLDQGEPTNPSDAAAKEHDEA
YAAYLRSGKNPYLYFSPADQRFIDQTKDAKDWGGKIGHYFFRAKKAIAPVLTDTPDHPSTSRPTKP
TKRSKPPPHIFINLAKKKKAGAGQVKRDNLAPMSDGAVQPDGGQPAVRNERATGSGNGSGGGGGGG
SGGVGISTGTFNNQTEFKFLENGWVEITANSSRLVHLNMPESENYRRVVVNNMDKTAVNGNMALDD
IHAQIVTPWSLVDANAWGVWFNPGDWQLIVNTMSELHLVSFEQEIFNVVLKTVSESATQPPTKVYN
NDLTASLMVALDSNNTMPFTPAAMRSETLGFYPWKPTIPTPWRYYFQWDRTLIPSHTGTSGTPTNI
YHGTDPDDVQFYTIENSVPVHLLRTGDEFATGTFFFDCKPCRLTHTWQTNRALGLPPFLNSLPQSE
GATNFGDIGVQQDKRRGVTQMGNTNYITEATIMRPAEVGYSAPYYSFEASTQGPFKTPIAAGRGGA
QTYENQAADGDPRYAFGRQHGQKTTTTGETPERFTYIAHQDTGRYPEGDWIQNINFNLPVTNDNVL
LPTDPIGGKTGINYTNIFNTYGPLTALNNVPPVYPNGQIWDKEFDTDLKPRLHVNAPFVCQNNCPG
QLFVKVAPNLTNEYDPDASANMSRIVTYSDFWWKGKLVFKAKLRASHTWNPIQQMSINVDNQFNYV
PSNIGGMKIVYEKSQLAPRKLY
000000000000000000000000000000000000000000000000000000000000000000
000000000000000000000000000000000000000000000000000000000000000000
000000000000000000000000000001111111111111111111111100000000000000
000000000000000000000000000000000000000000000000000000001111111111
111100000000000000000000000000000000000000000000000000000000000000
000001111111111110000000000000000000000000000000000000000000000000
000000000000000000000000000000000000000000000000000000111111111111
111111111111000000000000000000000000000000000000000000000000000000
000000000000000000000000000000000000000000000000000000000000000000
000000000000000000000000000000000000000000000000000000000000000000
011111111111100000000000000000000000000000000000000011111111111000
0000000000000011111111
> Q25641 CRPI_PERAM
MKTALVFAAVVAFVAARFPDHKDYKQLADKQFLAKQRDVLRLFHRVHQHNILNDQVEVGIPMTSKQ
TSATTVPPSGEAVHGVLQEGHARPRGEPFSVNYEKHREQAIMLYDLLYFANDYDTFYKTACWARDR
VNEGMFMYSFSIAVFHRDDMQGVMLPPPYEVYPYLFVDHDVIHMAQKYWMKNAGSGEHHSHVIPVN
FTLRTQDHLLAYFTSDVNLNAFNTYYRYYYPSWYNTTLYGHNIDRRGEQFYYTYKQIYARYFLERL
SNDLPDVYPFYYSKPVKSAYNPNLRYHNGEEMPVRPSNMYVTNFDLYYIADIKNYEKRVEDAIDFG
YAFDEHMKPHSLYHDVHGMEYLADMIEGNMDSPNFYFYGSIYHMYHSMIGHIVDPYHKMGLAPSLE
HPETVLRDPVFYQLWKRVDHLFQKYKNRLPRYTHDELAFEGVKVENVDVGKLYTYFEQYDMSLDMA
VYVNNVDQISNVDVQLAVRLNHKPFTYNIEVSSDKAQDVYVAVFLGPKYDYLGREYDLNDRRHYFV
EMDRFPYHVGAGKTVIERNSHDSNIIAPERDSYRTFYKKVQEAYEGKSQYYVDKGHNYCGYPENLL

IPKGKKGGQAYTFYVIVTPYVKQDEHDFEPYNYKAFSYCGVGSERKYPDNKPLGYPFDRKIYSNDF
YTPNMYFKDVIIFHKKYDEVGVQGH
000000000000000000000000000000000000000000000000000000000000000000
000000000000000000000000000000000000000000000000000000000000000000
000000000000000000000000000000000000000000000000000000000000000000
000000000000000000000000000000000000000000000000000000000000000000
000000000000000000000000000000000000000000000000000000000000000000
000000000000000000000000000000000000000000000000000000000000000000
000000000000000000000000000000000000000000000000000000000000000000
000111111000000000000000000000000000000000000000000000000000000000
000000000000000000000000000000000000000000000000000111111111111111
111111111110000000000000000000000000000000000000000000000000000000
0000000000000000000000000
> P05877 ENV_HV1MN
MRVKGIRRNYQHWWGWGTMLLGLLMICSATEKLWVTVYYGVPVWKEATTTLFCASDAKAYDTEVHN
VWATQACVPTDPNPQEVELVNVTENFNMWKNNMVEQMHEDIISLWDQSLKPCVKLTPLCVTLNCTD
LRNTTNTNNSTANNNSNSEGTIKGGEMKNCSFNITTSIRDKMQKEYALLYKLDIVSIDNDSTSYRL
ISCNTSVITQACPKISFEPIPIHYCAPAGFAILKCNDKKFSGKGSCKNVSTVQCTHGIRPVVSTQL
LLNGSLAEEEVVIRSENFTDNAKTIIVHLNESVQINCTRPNYNKRKRIHIGPGRAFYTTKNIIGTI
RQAHCNISRAKWNDTLRQIVSKLKEQFKNKTIVFNQSSGGDPEIVMHSFNCGGEFFYCNTSPLFNS
TWNGNNTWNNTTGSNNNITLQCKIKQIINMWQEVGKAMYAPPIEGQIRCSSNITGLLLTRDGGKDT
DTNDTEIFRPGGGDMRDNWRSELYKYKVVTIEPLGVAPTKAKRRVVQREKRAAIGALFLGFLGAAG
STMGAASVTLTVQARLLLSGIVQQQNNLLRAIEAQQHMLQLTVWGIKQLQARVLAVERYLKDQQLL
GFWGCSGKLICTTTVPWNASWSNKSLDDIWNNMTWMQWEREIDNYTSLIYSLLEKSQTQQEKNEQE
LLELDKWASLWNWFDITNWLWYIKIFIMIVGGLVGLRIVFAVLSIVNRVRQGYSPLSLQTRPPVPR
GPDRPEGIEEEGGERDRDTSGRLVHGFLAIIWVDLRSLFLFSYHHRDLLLIAARIVELLGRRGWEV
LKYWWNLLQYWSQELKSSAVSLLNATAIAVAEGTDRVIEVLQRAGRAILHIPTRIRQGLERALL
000000000000000000000000000000000000000000000000000000000000000000
000000000000000000000000000000000000000000000000000000000000000000
000000000000000000000000000000000000000000000000000000000000000000
000000000000000000000000000000000000000000000000000000000000000000
000000000000000000000000000000000000000000001111111111111110000000
000000000000000000000000000000000000000000000000000000000000000000
000000000000000000000000000000000000000000000000000000000000000000
000000000000000000000000000000000000000000000000000000000000000000
000000000000000000000000000000000000000000000000000000000000000000
000000000000000000000000000000000000000000000000000000000000000000
000000000000000000000000000000000000000000000000000000000000000000

000000000000000000000000000000000000000000000000000000000000000000
0000000000000000000000000000000000000000000000000000000000000000
> P03114 VE1_HPV16
MADPAGTNGEEGTGCNGWFYVEAVVEKKTGDAISDDENENDSDTGEDLVDFIVNDNDYLTQAETET
AHALFTAQEAKQHRDAVQVLKRKYLVSPLSDISGCVDNNISPRLKAICIEKQSRAAKRRLFESEDS
GYGNTEVETQQMLQVEGRHETETPCSQYSGGSGGGCSQYSSGSGGEGVSERHTICQTPLTNILNVL
KTSNAKAAMLAKFKELYGVSFSELVRPFKSNKSTCCDWCIAAFGLTPSIADSIKTLLQQYCLYLHI
QSLACSWGMVVLLLVRYKCGKNRETIEKLLSKLLCVSPMCMMIEPPKLRSTAAALYWYKTGISNIS
EVYGDTPEWIQRQTVLQHSFNDCTFELSQMVQWAYDNDIVDDSEIAYKYAQLADTNSNASAFLKSN
SQAKIVKDCATMCRHYKRAEKKQMSMSQWIKYRCDRVDDGGDWKQIVMFLRYQGVEFMSFLTALKR
FLQGIPKKNCILLYGAANTGKSLFGMSLMKFLQGSVICFVNSKSHFWLQPLADAKIGMLDDATVPC
WNYIDDNLRNALDGNLVSMDVKHRPLVQLKCPPLLITSNINAGTDSRWPYLHNRLVVFTFPNEFPF
DENGNPVYELNDKNWKSFFSRTWSRLSLHEDEDKENDGDSLPTFKCVSGQNTNTL
000000000000000000000000000000000000000000000000000000000000000000
000000000000000000000000000000000000000000000000000000000000000000
000000000000000000000000000000000000000000000000000000000000000000
000000000000000000000000000000000000000000000000000000000000000000
000000000000000000000000000000000000000000000000000000000000000000
000000000000000000000000000000000000000000000000000000000000000000
000000000000000000000000000000000000000000000000000000000000000000
000000000000000000000000000000000000000001111111111111111111100000
000000000000000000000000000000000000000000000000000000000000000001
1111111111111111100000000000000000000000000000000000000
> P17547 ADHE_ECO57
MAVTNVAELNALVERVKKAQREYASFTQEQVDKIFRAAALAAADARIPLAKMAVAESGMGIVEDKV
IKNHFASEYIYNAYKDEKTCGVLSEDDTFGTITIAEPIGIICGIVPTTNPTSTAIFKSLISLKTRN
AIIFSPHPRAKDATNKAADIVLQAAIAAGAPKDLIGWIDQPSVELSNALMHHPDINLILATGGPGM
VKAAYSSGKPAIGVGAGNTPVVIDETADIKRAVASVLMSKTFDNGVICASEQSVVVVDSVYDAVRE
RFATHGGYLLQGKELKAVQDVILKNGALNAAIVGQPAYKIAELAGFSVPENTKILIGEVTVVDESE
PFAHEKLSPTLAMYRAKDFEDAVEKAEKLVAMGGIGHTSCLYTDQDNQPARVSYFGQKMKTARILI
NTPASQGGIGDLYNFKLAPSLTLGCGSWGGNSISENVGPKHLINKKTVAKRAENMLWHKLPKSIYF
RRGSLPIALDEVITDGHKRALIVTDRFLFNNGYADQITSVLKAAGVETEVFFEVEADPTLSIVRKG
AELANSFKPDVIIALGGGSPMDAAKIMWVMYEHPETHFEELALRFMDIRKRIYKFPKMGVKAKMIA
VTTTSGTGSEVTPFAVVTDDATGQKYPLADYALTPDMAIVDANLVMDMPKSLCAFGGLDAVTHAME
AYVSVLASEFSDGQALQALKLLKEYLPASYHEGSKNPVARERVHSAATIAGIAFANAFLGVCHSMA
HKLGSQFHIPHGLANALLICNVIRYNANDNPTKQTAFSQYDRPQARRRYAEIADHLGLSAPGDRTA
AKIEKLLAWLETLKAELGIPKSIREAGVQEADFLANVDKLSEDAFDDQCTGANPRYPLISELKQIL
LDTYYGRDYVEGETAAKKEAAPAKAEKKAKKSA

000000000000000000000000000000000000000000000000000000000000000000
000000000000000000000000000000000000000000000000000000000000000000
000000000000000000000000000000000000000000000000000000000000000000
000000000000000000000000000000000000000000000000000000000000000000
000000000000000000000000000000000000000000000000000000000000000000
000000000000000000000000000000000000000000000000000000000000000000
000000000000000000000000000000000000000000000000000000000000000000
000000000000000000000000000000000000000000000000000000000000000000
000000000000000000000000000000000000000000000000000000000000000000
000000000000000000000000000000000000000000000000000000000000000000
000000000000000000000000000000000000000000000000000000000000000000
000000000000000000000000000000000000000000000000000000000000000000
000000000000000000000000000000000000000000000000000000011111111111
111000000000000000000000000000000
> P10515 ODP2_HUMAN
MSPHCSTTYLRTLGRTTMFWKTTEGRDGKMAVQEFSEFGLLLQLLGSPGRRYYSLPPHQKVPLPSL
SPTMQAGTIARWEKKEGDKINEGDLIAEVETDKATVGFESLEECYMAKILVAEGTRDVPIGAIICI
TVGKPEDIEAFKNYTLDSSAAPTPQAAPAPTPAATASPPTPSAQAPGSSYPPHMQVLLPALSPTMT
MGTVQRWEKKVGEKLSEGDLLAEIETDKATIGFEVQEEGYLAKILVPEGTRDVPLGTPLCIIVEKE
ADISAFADYRPTEVTDLKPQVPPPTPPPVAAVPPTPQPLAPTPSTPCPATPAGPKGRVFVDPLAKK
LAVEKGIDLTQVKGTGPDGRITKKDIDSFVPSKVAPAPAAVVPPTGPGMAPVPTGVFTDIPISNIR
RVIAQRLMQSKQTIPHYYLSIDVNMGEVLLVRKELNKILEGRSKISVNDFIIKASALACLKVPEAN
SSWMDTVIRQNHVVDVSVAVSTPAGLITPIVFNAHIKGVETIANDVVSLATKAREGKLQPHEFQGG
TFTISNLGMFGIKNFSAIINPPQACILAIGASEDKLVPADNEKGFDVASMMSVTLSCDHRVVDGAV
GAQWLAEFRKYLEKPITMLL
000000000000000000000000000000000000000000000000000000000000000000
000000000000000000000000000011111111111000000000000000000000000000
000000000000000000000000000000000000000000011111111111111111110000
000000000000000000000000000000001111111111111000000000000000001111
111111110000000000000011111111111111100000000000000000000000000000
000000000000000000000000000000000000000000000000000000000000000000
000000000000000000000000000000000000000000000000000000000000000000
000000000000000000000000000000000000000000000000000000000000000000
000000000000000000000000000000000000000000000000000000000000000000
00000000000000000000
> P12640 VGLB_BHV1C
MAARGGAERAAGAGDGRRGQRRHLRPGRVLAALRGPAAPGAGGARAALAAALLWATWALLLAAPAA
GRPATTPPAPPPEEAASPAPPASPSPPGPDGDDAASPDNSTDVRAALRLAQAAGENSRFFVCPPPS

GATVVRLAPARPCPEYGLGRNYTEGIGVIYKENIAPYTFKAYIYKNVIVTTTWAGSTYAAITNQYT
DRVPVGMGEITDLVDKKWRCLSKAEYLRSGRKVVAFDRDDDPWEAPLKPARLSAPGVRGWHTTDDV
YTALGSAGLYRTGTSVNCIVEEVEARSVYPYDSFALSTGDIIYMSPFYGLREGAHREHTSYSPERF
QQIEGYYKRDMATGRRLKEPVSRNFLRTQHVTVAWDWVPKRKNVCSLAKWREADEMLRDESRGNFR
FTARSLSATFVSDSHTFALQNVPLSDCVIEEAEAAVERVYRERYNGTHVLSGSLETYLARGGFVVA
FRPMLSNELAKLYLQELARSNGTLEGLFAAAAPKPGPRRARRAAPSAPGGPGAANGPAGDGDAGGR
VTTVSSAEFAALQFTYDHIQDHVNTMFSRLATSWCLLQNKERALWAEAAKLNPSAAASAALDRRAA
ARMLGDAMAVTYCHELGEGRVFIENSMRAPGGVCYSRPPVSFAFGNESEPVEGQLGEDNELLPGRE
LVEPCTANHKRYFRFGADYVYYENYAYVRRVPLAELEVISTFVDLNLTVLEDREFLPLEVYTRAEL
ADTGLLDYSEIQRRNQLHELRFYDIDRVVKTDGNMAIMRGLANFFQGLGAVGQAVGTVVLGAAGAA
LSTVSGIASFIANPFGALATGLLVLAGLVAAFLAYRYISRLRSNPMKALYPITTRALKDDARGATA
PGEEEEEFDAAKLEQAREMIKYMSLVSAVERQEHKAKKSNKGGPLLATRLTQLALRRRAPPEYQQL
PMADVGGA
000000000000000000000000000000000000000000000000000000000000000000
000000000000000000000000000000000000000000000000000000000000000000
000000000000000000000000000000000000000000000000000000000000000000
000000000000000000000000000000000000000000000000000000000000000000
000000000000000000000000000000000000000000000000000000000000000000
111111111111111111111000000000000000000000000000000000000000000000
000000000000000000000000000000000000000000000000000000000000000000
000000000000111111111111111111111111111111111000000000000000000000
000000000000000000000000000000000000000000000000000000000000000000
000000000000000000000000000000000000000000000000000000000000000000
000000000000000000000000000000000000000000000000000000000000000000
000000000000000000000000000000000000000000000000000000000000000000
000000000000000000000000000000000000000000000000000000000000000000
000000000000000000000000000000000000000000000000000000000000000000
00000000
> P11387 TOP1_HUMAN
MSGDHLHNDSQIEADFRLNDSHKHKDKHKDREHRHKEHKKEKDREKSKHSNSEHKDSEKKHKEKEK
TKHKDGSSEKHKDKHKDRDKEKRKEEKVRASGDAKIKKEKENGFSSPPQIKDEPEDDGYFVPPKED
IKPLKRPRDEDDADYKPKKIKTEDTKKEKKRKLEEEEDGKLKKPKNKDKDKKVPEPDNKKKKPKKE
EEQKWKWWEEERYPEGIKWKFLEHKGPVFAPPYEPLPENVKFYYDGKVMKLSPKAEEVATFFAKML
DHEYTTKEIFRKNFFKDWRKEMTNEEKNIITNLSKCDFTQMSQYFKAQTEARKQMSKEEKLKIKEE
NEKLLKEYGFCIMDNHKERIANFKIEPPGLFRGRGNHPKMGMLKRRIMPEDIIINCSKDAKVPSPP
PGHKWKEVRHDNKVTWLVSWTENIQGSIKYIMLNPSSRIKGEKDWQKYETARRLKKCVDKIRNQYR
EDWKSKEMKVRQRAVALYFIDKLALRAGNEKEEGETADTVGCCSLRVEHINLHPELDGQEYVVEFD
FLGKDSIRYYNKVPVEKRVFKNLQLFMENKQPEDDLFDRLNTGILNKHLQDLMEGLTAKVFRTYNA

SITLQQQLKELTAPDENIPAKILSYNRANRAVAILCNHQRAPPKTFEKSMMNLQTKIDAKKEQLAD
ARRDLKSAKADAKVMKDAKTKKVVESKKKAVQRLEEQLMKLEVQATDREENKQIALGTSKLNYLDP
RITVAWCKKWGVPIEKIYNKTQREKFAWAIDMADEDYEF
000000000000000000000000000000000000000000000000000000000000000000
000000000000000000000000000000000000000000000000000000000000000000
000000000000000000000000000000000000000000000000000000000000000000
000000111111111111111111110000000000000000000000000000000000000000
000000000000000000000000000000000000000000000000000000000000000000
000000000000000000111111111111111111110000000000000000000000000000
111111111111111111110000000000000000000000000000000000000000000000
000000000000000000000000000000000000000000000000000000111111111111
111111100000000000000000000000000000000000000000000000000000000000
000000000000000000000000000000000000000000000000000000000000000000
000000000000000000000000000000000000000000000000000000000000000000
000000000000000000000000000000000000000000000000000000000000000000
000000000000000000000000000000000000000
> P43238 ALL12_ARAHY
MRGRVSPLMLLLGILVLASVSATHAKSSPYQKKTENPCAQRCLQSCQQEPDDLKQKACESRCTKLE
YDPRCVYDPRGHTGTTNQRSPPGERTRGRQPGDYDDDRRQPRREEGGRWGPAGPREREREEDWRQP
REDWRRPSHQQPRKIRPEGREGEQEWGTPGSHVREETSRNNPFYFPSRRFSTRYGNQNGRIRVLQR
FDQRSRQFQNLQNHRIVQIEAKPNTLVLPKHADADNILVIQQGQATVTVANGNNRKSFNLDEGHAL
RIPSGFISYILNRHDNQNLRVAKISMPVNTPGQFEDFFPASSRDQSSYLQGFSRNTLEAAFNAEFN
EIRRVLLEENAGGEQEERGQRRWSTRSSENNEGVIVKVSKEHVEELTKHAKSVSKKGSEEEGDITN
PINLREGEPDLSNNFGKLFEVKPDKKNPQLQDLDMMLTCVEIKEGALMLPHFNSKAMVIVVVNKGT
GNLELVAVRKEQQQRGRREEEEDEDEEEEGSNREVRRYTARLKEGDVFIMPAAHPVAINASSELHL
LGFGINAENNHRIFLAGDKDNVIDQIEKQAKDLAFPGSGEQVEKLIKNQKESHFVSARPQSQSQSP
SSPEKESPEKEDQEEENQGGKGPLLSILKAFN
000000000000000000000001111111111110000000000001111111111000000000
000000000000000000000111111111111111111111111111111000011111111111
111111111111111111111000000000000000000000000000000000000000000000
000000000000000000000000000000000000000000000000000000000000000000
000000000000000000000000000011111111111100000000000000000000111111
111100000000111111111111000000000000000000000000000000000000001111
111111000000111111111100000000000000000000000000000000000000011111
111111111100000000000000000000000001111111111000000000000000001111
111111000011111111110011111111111111111100000000011111111110000000
01111111111110000000000000000000
> P02458 CO2A1_HUMAN
MIRLGAPQSLVLLTLLVAAVLRCQGQDVRQPGPKGQKGEPGDIKDIVGPKGPPGPQGPAGEQGPRG

DRGDKGEKGAPGPRGRDGEPGTLGNPGPPGPPGPPGPPGLGGNFAAQMAGGFDEKAGGAQLGVMQG
PMGPMGPRGPPGPAGAPGPQGFQGNPGEPGEPGVSGPMGPRGPPGPPGKPGDDGEAGKPGKAGERG
PPGPQGARGFPGTPGLPGVKGHRGYPGLDGAKGEAGAPGVKGESGSPGENGSPGPMGPRGLPGERG
RTGPAGAAGARGNDGQPGPAGPPGPVGPAGGPGFPGAPGAKGEAGPTGARGPEGAQGPRGEPGTPG
SPGPAGASGNPGTDGIPGAKGSAGAPGIAGAPGFPGPRGPPDPQGATGPLGPKGQTGKPGIAGFKG
EQGPKGEPGPAGPQGAPGPAGEEGKRGARGEPGGVGPIGPPGERGAPGNRGFPGQDGLAGPKGAPG
ERGPSGLAGPKGANGDPGRPGEPGLPGARGLTGRPGDAGPQGKVGPSGAPGEDGRPGPPGPQGARG
QPGVMGFPGPKGANGEPGKAGEKGLPGAPGLRGLPGKDGETGAEGPPGPAGPAGERGEQGAPGPSG
FQGLPGPPGPPGEGGKPGDQGVPGEAGAPGLVGPRGERGFPGERGSPGAQGLQGPRGLPGTPGTDG
PKGASGPAGPPGAQGPPGLQGMPGERGAAGIAGPKGDRGDVGEKGPEGAPGKDGGRGLTGPIGPPG
PAGANGEKGEVGPPGPAGSAGARGAPGERGETGPPGTSGIAGPPGADGQPGAKGEQGEAGQKGDAG
APGPQGPSGAPGPQGPTGVTGPKGARGAQGPPGATGFPGAAGRVGPPGSNGNPGPPGPPGPSGKDG
PKGARGDSGPPGRAGEPGLQGPAGPPGEKGEPGDDGPSGAEGPPGPQGLAGQRGIVGLPGQRGERG
FPGLPGPSGEPGQQGAPGASGDRGPPGPVGPPGLTGPAGEPGREGSPGADGPPGRDGAAGVKGDRG
ETGAVGAPGAPGPPGSPGPAGPTGKQGDRGEAGAQGPMGPSGPAGARGIQGPQGPRGDKGEAGEPG
ERGLKGHRGFTGLQGLPGPPGPSGDQGASGPAGPSGPRGPPGPVGPSGKDGANGIPGPIGPPGPRG
RSGETGPAGPPGNPGPPGPPGPPGPGIDMSAFAGLGPREKGPDPLQYMRADQAAGGLRQHDAEVDA
TLKSLNNQIESIRSPEGSRKNPARTCRDLKLCHPEWKSGDYWIDPNQGCTLDAMKVFCNMETGETC
VYPNPANVPKKNWWSSKSKEKKHIWFGETINGGFHFSYGDDNLAPNTANVQMTFLRLLSTEGSQNI
TYHCKNSIAYLDEAAGNLKKALLIQGSNDVEIRAEGNSRFTYTALKDGCTKHTGKWGKTVIEYRSQ
KTSRLPIIDIAPMDIGGPEQEFGVDIGPVCFL
000000000000000000000000000000000000000000000000000000000000000000
000000000000000000000000000000000000000000000000000000000000000000
000000000000000000000000000000000000000000000000000000000000000000
000000000000000000000000000000000000000000000000000000000000001111
111111000000000000000000000111111110000000000000000000000000000000
000000000000000000000000000000000000000000000000000000000000000000
000000000000000000000000000000000000000000000000000000000000000000
000000000000000000000000000000000000111111111111111000000000000000
000000000000000000000000000000000000000000000000000000000000000000
000000000000000000000000000000000000000000000000000000000000000000
000000000000000000000000000000000000000000000000000000000000000000
000000000000000000000000000000000000000000000000000000000000000000
000000000000000000000000000000000000000000000000000000000000000000
000000000000000000000000000000000000000000000000000000000000000000
000000000000000000000000000000000000000000000000000000000000000000
000000000000000000000000000000000000000000000000000000000000000000
000000000000000000000000000000000000000000000000000000000000000000

000000000000000000000000000000000000000000000000000000000000000000
000000000000000000000000000000000000000000000000000000000000000000
000000000000000000000000000000000000000000000000000000000000000000
000000000000000000000000000000000000000000000000000000000000000000
00000000000000000000000000000000

> P07564 POLG_DEN2J

MNNQRKKARSTPFNMLKRERNRVSTVQQLTKRFSLGMLQGRGPLKLFMALVAFLRFLTIPPTAGIL
KRWGTIKKSKAINVLRGFRKEIGRMLNILNRRRRTAGVIIMLIPTAMAFHLTTRNGEPHMIVGRQE
KGKSLLFKTEDGVNMCTLMAIDLGELCEDTITYKCPLLRQNEPEDIDCWCNSTSTWVTYGTCATTG
EHRREKRSVALVPHVGMGLETRTETWMSSEGAWKHVQRIETWILRHPGFTIMAAILAYTIGTTHFQ
RALIFILLTAVAPSMTMRCIGISNRDFVEGVSGGSWVDIVLEHGSCVTTMAKNKPTLDFELIKTEA
KQPATLRKYCIEAKLTNTTTESRCPTQGEPSLNEEQDKRFLCKHSMVDRGWGNGCGLFGKGGIVTC
AMFTCKKNMEGKVVLPENLEYTIVITPHSGEEHAVGNDTGKHGKEIKITPQSSITEAELTGYGTVT
MECSPRTGLDFNEMVLLQMEDKAWLVHRQWFLDLPLPWLPGADTQGSNWIQKETLVTFKNPHAKKQ
DVVVLGSQEGAMHTALTGATEIQMSSGNLLFTGHLKCRLRMDKLQLKGMSYSMCTGKFKIVKEIAE
TQHGTIVIRVQYEGDGSPCKIPFEIMDLEKRHVLGRLITVNPIVTEKDSPVNIEAEPPFGDSYIII
GVEPGQLKLNWFKKGSSIGQMFETTMRGAKRMAILGDTAWDFGSLGGVFTSIGKALHQVFGAIYGA
AFSGVSWTMKILIGVIITWIGMNSRSTSLSVSLVLVGVVTLYLGAMVQADSGCVVSWKNKELKCGS
GIFITDNVHTWTEQYKFQPESPSKLASAIQKAHEEGICGIRSVTRLENLMWKQITPELNHILSENE
VKLTIMTGDIKGIMQAGKRSLRPQPTELKYSWKTWGKAKMLSTESHNQTFLIDGPETAECPNTNRA
WNSLEVEDYGFGVFTTNIWLKLREKQDVFCDSKLMSAAIKDNRAVHADMGYWIESALNDTWKMEKA
SFIEVKSCHWPKSHTLWSNGVLESEMIIPKNFAGPVSQHNYRPGYHTQTAGPWHLGKLEMDFDFCE
GTTVVVTEDCGNRGPSLRTTTASGKLITEWCCRSCTLPPLRYRGEDGCWYGMEIRPLKEKEENLVN
SLVTAGHGQIDNFSLGVLGMALFLEEMLRTRVGTKHAILLVAVSFVTLITGNMSFRDLGRVMVMVG
ATMTDDIGMGVTYLALLAAFKVRPTFAAGLLLRKLTSKELMMATIGIALLSQSTIPETILELTDAL
ALGMMVLKIVRNMEKYQLAVTIMAILCVPNAVILQNAWKVSCTILAAVSVSPLLLTSSQQKADWIP
LALTIKGLNPTAIFLTTLSRTSKKRSWPLNEAIMAVGMVSILASSLLKNDIPMTGPLVAGGLLTVC
YVLTGRSADLELERAADVKWEDQAEISGSSPILSITISEDGSMSIKNEEEEQTLTILIRTGLLVIS
GVFPVSIPITAAAWYLWEVKKQRAGVLWDVPSPPPVGKAELEDGAYRIKQRGILGYSQIGAGVYKE
GTFHTMWHVTRGAVLMHKGKRIEPSWADVKKDLISYGGGWKLEGEWKEGEEVQVLALEPGKNPRAV
QTKPGLFKTNTGTIGAVSLDFSPGTSGSPIVDRKGKVVGLYGNGVVTRSGAYVSAIAQTEKSIEDN
PEIEDDIFRKKRLTIMDLHPGAGKTKRYLPAIVREAIKRGLRTLILAPTRVVAAEMEEALRGLPIR
YQTPAIRAEHTGREIVDLMCHATFTMRLLSPVRVPNYNLIIMDEAHFTDPASIAARGYISTRVEMG
EAAGIFMTATPPGSRDPFPQSNAPIMDEEREIPERSWNSGHEWVTDFKGKTVWFVPSIKAGNDIAA
CLRKNGKKVIQLSRKTFDSEYVKTRANDWDFVVTTDISEMGANFKAERVIDPRRCMKPVILTDGEE
RVILAGPMPVTHSSAAQRRGRIGRNPKNENDQYIYMGEPLENDEDCAHWKEAKMLLDNINTPEGII
PSMFEPEREKVDAIDGEYRLRGEARKTFVDLMRRGDLPVWLAYRVAAEGINYADRRWCFDGIKNNQ
ILEENVEVEIWTKEGERKKLKPRWLDARIYSDPLALKEFKEFAAGRKSLTLNLITEMGRLPTFMTQ

KARDALDNLAVLHTAEAGGRAYNHALSELPETLETLLLLTLLATVTGGIFLFLMSGKGIGKMTLGM
CCIITASILLWYAQIQPHWIAASIILEFFLIVLLIPEPEKQRTPQDNQLTYVVIAILTVVAATMAN
EMGFLEKTKKDLGLGSITTQESESNILDIDLRPASAWTLYAVATTFVTPMLRHSIENSSVNVSLTA
IANQATVLMGLGKGWPLSKIHIGVPLLAIGCYSQVNPITLTAALLLLVAHYAIIGPGLQAKATREA
QKRAAAGIMKNPTVDGITVIDLDPIPYDPKFEKQLGQVMLLILCVTQVLMMRTTWALCEALTLATG
PISTLWEGNPGRFWNTTIAVSMANIFRGSYLAGAGLLFSIMKNTTNTRRGTGNIGETLGEKWKSRL
NALGKSEFQIYKKSGIQEVDRTLAKEGIKRGETDHHAVSRGSAKLRWFVERNMVTPEGKVVDLGCG
RGGWSYYCGGLKNVREVKGLTKGGPGHEEPIPMSTYGWNLVRLQSGVDVFFTPPEKCDTLLCDIGE
SSPNPTIEAGRTLRVLNLVENWLNNNTQFCIKVLNPYMPSVIEKMETLQRKYGGALVRNPLSRNST
HEMYWVSNASGNIVSSVNMISRMLINRFTMKHKKATYETDVDLGSGTRNIGIESEIPNLDIIGKRI
EKIKQEHETSWHYDQDHPYKTWAYHGSYETKQTGSASSMVNGVVRLLTKPWDVVPMVTQMAMTDTT
PFGQQRVFKEKVDTRTQEPKEGTKKLMKITAEWLWKELGKKKTPRMCTREEFTRKVRSNAALGAIF
TDENKWKSAREAVEDSRFWELVDRERNLHLEGKCETCVYNMMGKREKKLGEFGKAKGSRAIWYMWL
GARFLEFEALGFLNEDHWFSRGNSLSGVEGEGLHKLGYILRDVSKKEGGAMYADDTAGWDTRITLE
DLKNEEMVTNHMEGEHKKLAEAIFKLTYQNKVVRVQRPTPRGTVMDIISRRDQRGSGQVGTYGLNT
FTNMEAQLIRQMEGEGIFKSIQHLTVTEEIAVQNWLARVGRERLSRMAISGDDCVVKPLDDRFASA
LTALNDMGKVRKDIQQWEPSRGWNDWTQVPFCSHHFHELVMKDGRVLVVPCRNQDELIGRARISQG
AGWSLKETACLGKSYAQMWTLMYFHRRDLRLAANAICSAVPSHWVPTSRTTWSIHAKHEWMTTEDM
LAVWNRVWIQENPWMEDKTPVESWEEVPYLGKREDQWCGSLIGLTSRATWAKNIQTAINQVRSLIG
NEEYTDYMPSMKRFRREEEEAGVLW
000000000000000000000000000000000000000000000000000000000000000000
000000000000000000000000000000000000000000000000111111111110000000
000000000000000000000000000000000000000000000000000000000000000000
000000000000000000000000000000000000000000000000000000000000000000
000000000000000000000000000000000000000000000000000000000000000000
000000000000000000000000000000000000000000000000000000000000000000
000000000000000000000000000000000000000000000000000000000000000000
000000000000000000000000000000000000000000000000000000000000000000
000000000000000000000000011111111100000000000000000000000000000000
000000000000000000000000000000000011111111111000000000000000000000
000000000000000000000000000000000000000000000000000000000000000000
000000000000000000000000000000000000000000000000000000000000000000
000000000000000000000000000000000000000000000000000000000000000000
000000000000000000000000000000000000000000000000000000000000000000
000000000000000000000000000000000000000000000000000000000000000000
000000000000000000000000000000000000000000000000000000000000000000
000000000000000000000000000000000000000000000000000000000000000000
000000000000000000000000000000000000000000000000000000000000000000

000000000000000000000000000000000000000000000000000000000000000000
000000000000000000000000000000000000000000000000000000000000000000
000000000000000000000000000000000000000000000000000000000000000000
000000000000000000000000000000000000000000000000000000000000000000
000000000000000000000000000000000000000000000000000000000000000000
000000000000000000000000000000000000000000000000000000000000000000
000000000000000000000000000000000000000000000000000000000000000000
000000000000000000000000000000000000000000000000000000000000000000
000000000000000000000000000000000000000000000000000000000000000000
000000000000000000000000000000000000000000000000000000000000000000
000000000000000000000000000000000000000000000000000000000000000000
000000000000000000000000000000000000000000000000000000000000000000
000000000000000000000000000000000000000000000000000000000000000000
000000000000000000000000000000000000000000000000000000000000000000
000000000000000000000000000000000000000000000000000000000000000000
000000000000000000000000000000000000000000000000000000000000000000
000000000000000000000000000000000000000000000000000000000000000000
000000000000000000000000000000000000000000000000000000000000000000
000000000000000000000000000000000000000000000000000000000000000000
000000000000000000000000000000000000000000000000000000000000000000
000000000000000000000000000000000000000000000000000000000000000000
000000000000000000000000000000000000000000000000000000000000000000
000000000000000000000000000000000000000000000000000000000000000000
000000000000000000000000000000000000000000000000000000000000000000
000000000000000000000000000000000000000000000000000000000000000000
000000000000000000000000000000000000000000000000000000000000000000
000000000000000000000000000000000000000000000000000000000000000000
000000000000000000000000000000000000000000000000000000000000000000
000000000000000000000000000000000000000000000000000000000000000000
000000000000000000000000000000000000000000000000000000000000000000
000000000000000000000000000000000000000000000000000000000000000000
000000000000000000000000000000000000000000000000000000000000000000
000000000000000000000000000000000000000000000000000000000000000000
0000000000000000000000000

> P13470 GTFC_STRMU

MEKKVRFKLRKVKKRWVTVSVASAVVTLTSLSGSLVKADSTDDRQQAVTESQASLVTTSEAAKETL
TATDTSTATSATSQPTATVTDNVSTTNQSTNTTANTANFDVKPTTTSEQSKTDNSDKIIATSKAVN
RLTATGKFVPANNNTAHSRTVTDKIVPIKPKIGKLKQPSSLSQDDIAALGNVKNIRKVNGKYYYYK

EDGTLQKNYALNINGKTFFFDETGALSNNTLPSKKGNITNNDNTNSFAQYNQVYSTDAANFEHVDH
YLTAESWYRPKYILKDGKTWTQSTEKDFRPLLMTWWPDQETQRQYVNYMNAQLGIHQTYNTATSPL
QLNLAAQTIQTKIEEKITAEKNTNWLRQTISAFVKTQSAWNSDSEKPFDDHLQKGALLYSNNSKLT
SQANSNYRILNRTPTNQTGKKDPRYTADRTIGGYEFLLANDVDNSNPVVQAEQLNWLHFLMNFGNI
YANDPDANFDSIRVDAVDNVDADLLQIAGDYLKAAKGIHKNDKAANDHLSILEAWSYNDTPYLHDD
GDNMINMDNRLRLSLLYSLAKPLNQRSGMNPLITNSLVNRTDDNAETAAVPSYSFIRAHDSEVQDL
IRNIIRAEINPNVVGYSFTMEEIKKAFEIYNKDLLATEKKYTHYNTALSYALLLTNKSSVPRVYYG
DMFTDDGQYMAHKTINYEAIETLLKARIKYVSGGQAMRNQQVGNSEIITSVRYGKGALKATDTGDR
TTRTSGVAVIEGNNPSLRLKASDRVVVNMGAAHKNQAYRPLLLTTDNGIKAYHSDQEAAGLVRYTN
DRGELIFTAADIKGYANPQVSGYLGVWVPVGAAADQDVRVAASTAPSTDGKSVHQNAALDSRVMFE
GFSNFQAFATKKEEYTNVVIAKNVDKFAEWGVTDFEMAPQYVSSTDGSFLDSVIQNGYAFTDRYDL
GISKPNKYGTADDLVKAIKALHSKGIKVMADWVPDQMYALPEKEVVTATRVDKYGTPVAGSQIKNT
LYVVDGKSSGKDQQAKYGGAFLEELQAKYPELFARKQISTGVPMDPSVKIKQWSAKYFNGTNILGR
GAGYVLKDQATNTYFSLVSDNTFLPKSLVNPNHGTSSSVTGLVFDGKGYVYYSTSGNQAKNAFISL
GNNWYYFDNNGYMVTGAQSINGANYYFLSNGIQLRNAIYDNGNKVLSYYGNDGRRYENGYYLFGQQ
WRYFQNGIMAVGLTRIHGAVQYFDASGFQAKGQFITTADGKLRYFDRDSGNQISNRFVRNSKGEWF
LFDHNGVAVTGTVTFNGQRLYFKPNGVQAKGEFIRDADGHLRYYDPNSGNEVRNRFVRNSKGEWFL
FDHNGIAVTGTRVVNGQRLYFKSNGVQAKGELITERKGRIKYYDPNSGNEVRNRYVRTSSGNWYYF
GNDGYALIGWHVVEGRRVYFDENGVYRYASHDQRNHWDYDYRRDFGRGSSSAVRFRHSRNGFFDNF
FRF
000000000000000000000000000000000000000000000000000000000000000000
000000000000000000000000000000000000000000000000000000000000000000
000000000000000000000000000000000000000000000000000000000000000000
000000000000000000000000000000000000000000000000000000000000000000
000000000000000000000000000000000000000000000000000000000000000000
000000000000000000000000000000000000000000000000000000000000000000
000000000000000000000000000000000000001111111111111111110000000000
000000000000000000000000000000000000000000000000000000000000000000
000000000000000000000000000000000000000000000000000000000000000000
000000000000000000000000000000000000000000000000000000000000000000
000000000000000000000000000000000000000000000000000000000000000000
000000000000000000000000000000000000000000000000000000000000000000
000000000000000000000000000000000000000000000000000000000000000000
000000000000000000000000000000000000000000000000000000000000000000
000000000000000000000000000000000000000000000000000000000000000000
000000000000000000000000000000000000000000000000000000000000000000
000000000000000000000000000000000000000000000000000000000000000000
000000000000000000000000000000000000000000000000000000000000000000

000000000000000000000000000000000000000000000000000000000000000000
000000000000000000000000000000000000000000000000000000000000000000
000000000000000000000000000000000000000000000000000000000000000000
000000000000000000000000000000000000000000000000000000000000000000
000

> O00555 CAC1A_HUMAN

MARFGDEMPARYGGGGSGAAAGVVVGSGGGRGAGGSRQGGQPGAQRMYKQSMAQRARTMALYNPIP
VRQNCLTVNRSLFLFSEDNVVRKYAKKITEWPPFEYMILATIIANCIVLALEQHLPDDDKTPMSER
LDDTEPYFIGIFCFEAGIKIIALGFAFHKGSYLRNGWNVMDFVVVLTGILATVGTEFDLRTLRAVR
VLRPLKLVSGIPSLQVVLKSIMKAMIPLLQIGLLLFFAILIFAIIGLEFYMGKFHTTCFEEGTDDI
QGESPAPCGTEEPARTCPNGTKCQPYWEGPNNGITQFDNILFAVLTVFQCITMEGWTDLLYNSNDA
SGNTWNWLYFIPLIIIGSFFMLNLVLGVLSGEFAKERERVENRRAFLKLRRQQQIERELNGYMEWI
SKAEEVILAEDETDGEQRHPFDGALRRTTIKKSKTDLLNPEEAEDQLADIASVGSPFARASIKSAK
LENSTFFHKKERRMRFYIRRMVKTQAFYWTVLSLVALNTLCVAIVHYNQPEWLSDFLYYAEFIFLG
LFMSEMFIKMYGLGTRPYFHSSFNCFDCGVIIGSIFEVIWAVIKPGTSFGISVLRALRLLRIFKVT
KYWASLRNLVVSLLNSMKSIISLLFLLFLFIVVFALLGMQLFGGQFNFDEGTPPTNFDTFPAAIMT
VFQILTGEDWNEVMYDGIKSQGGVQGGMVFSIYFIVLTLFGNYTLLNVFLAIAVDNLANAQELTKD
EQEEEEAANQKLALQKAKEVAEVSPLSAANMSIAVKEQQKNQKPAKSVWEQRTSEMRKQNLLASRE
ALYNEMDPDERWKAAYTRHLRPDMKTHLDRPLVVDPQENRNNNTNKSRAAEPTVDQRLGQQRAEDF
LRKQARYHDRARDPSGSAGLDARRPWAGSQEAELSREGPYGRESDHHAREGSLEQPGFWEGEAERG
KAGDPHRRHVHRQGGSRESRSGSPRTGADGEHRRHRAHRRPGEEGPEDKAERRARHREGSRPARGG
EGEGEGPDGGERRRRHRHGAPATYEGDARREDKERRHRRRKENQGSGVPVSGPNLSTTRPIQQDLG
RQDPPLAEDIDNMKNNKLATAESAAPHGSLGHAGLPQSPAKMGNSTDPGPMLAIPAMATNPQNAAS
RRTPNNPGNPSNPGPPKTPENSLIVTNPSGTQTNSAKTARKPDHTTVDIPPACPPPLNHTVVQVNK
NANPDPLPKKEEEKKEEEEDDRGEDGPKPMPPYSSMFILSTTNPLRRLCHYILNLRYFEMCILMVI
AMSSIALAAEDPVQPNAPRNNVLRYFDYVFTGVFTFEMVIKMIDLGLVLHQGAYFRDLWNILDFIV
VSGALVAFAFTGNSKGKDINTIKSLRVLRVLRPLKTIKRLPKLKAVFDCVVNSLKNVFNILIVYML
FMFIFAVVAVQLFKGKFFHCTDESKEFEKDCRGKYLLYEKNEVKARDREWKKYEFHYDNVLWALLT
LFTVSTGEGWPQVLKHSVDATFENQGPSPGYRMEMSIFYVVYFVVFPFFFVNIFVALIIITFQEQG
DKMMEEYSLEKNERACIDFAISAKPLTRHMPQNKQSFQYRMWQFVVSPPFEYTIMAMIALNTIVLM
MKFYGASVAYENALRVFNIVFTSLFSLECVLKVMAFGILNYFRDAWNIFDFVTVLGSITDILVTEF
GNNFINLSFLRLFRAARLIKLLRQGYTIRILLWTFVQSFKALPYVCLLIAMLFFIYAIIGMQVFGN
IGIDVEDEDSDEDEFQITEHNNFRTFFQALMLLFRSATGEAWHNIMLSCLSGKPCDKNSGILTREC
GNEFAYFYFVSFIFLCSFLMLNLFVAVIMDNFEYLTRDSSILGPHHLDEYVRVWAEYDPAAWGRMP
YLDMYQMLRHMSPPLGLGKKCPARVAYKRLLRMDLPVADDNTVHFNSTLMALIRTALDIKIAKGGA
DKQQMDAELRKEMMAIWPNLSQKTLDLLVTPHKSTDLTVGKIYAAMMIMEYYRQSKAKKLQAMREE
QDRTPLMFQRMEPPSPTQEGGPGQNALPSTQLDPGGALMAHESGLKESPSWVTQRAQEMFQKTGTW
SPEQGPPTDMPNSQPNSQSVEMREMGRDGYSDSEHYLPMEGQGRAASMPRLPAENQRRRGRPRGNN

LSTISDTSPMKRSASVLGPKARRLDDYSLERVPPEENQRHHQRRRDRSHRASERSLGRYTDVDTGL
GTDLSMTTQSGDLPSKERDQERGRPKDRKHRQHHHHHHHHHHPPPPDKDRYAQERPDHGRARARDQ
RWSRSPSEGREHMAHRQGSSSVSGSPAPSTSGTSTPRRGRRQLPQTPSTPRPHVSYSPVIRKAGGS
GPPQQQQQQQQQQQAVARPGRAATSGPRRYPGPTAEPLAGDRPPTGGHSSGRSPRMERRVPGPARS
ESPRACRHGGARWPASGPHVSEGPPGPRHHGYYRGSDYDEADGPGSGGGEEAMAGAYDAPPPVRHA
SSGATGRSPRTPRASGPACASPSRHGRRLPNGYYPAHGLARPRGPGSRKGLHEPYSESDDDWC
000000000000000000000000000000000000000000000000000000000000000000
000000000000000000000000000000000000000000000000000000000000000000
000000000000000000000000000000000000000000000000000000000000000000
000000000000000000000000000000000000000000000000000000000000000000
000000000000000000000000000000000000000000000000000000000000000000
000000000000000000000000000000000000000000000000000000000000000000
000000000000000000000000000000000000000000000000000000000000000000
000000000000000000000000000000000000000000000000000000000000000000
000000000000000000000000000000000000000111111111110000000000000000
000000000000000000000000000000000000000000111111111110000000000000
000000000000000000000000000000000000000000000000000000000000000000
000000000000000000000000000000000000000000000000000000000000000000
000000000000000000000000000000000000000000000000000000000000000000
000000000000000000000000000000000000000000000000000000000000000000
000000000000000000000000000000000000000000000000000000000000000000
000000000000000000000000000000000000000000000000000000000000000000
000000000000000000000000000000000000000000000000000000000000000000
000000000000000000000000000000000000000000000000000000000000000000
000000000000000000000000000000000000000000000000000000000000000000
000000000000000000000000000000000000000000000000000000000000000000
000000000000000000000000000000000000000000000000000000000000000000
000000000000000000000000000000000000000000000000000000000000000000
000000000000000000000000000000000000000000000000000000000000000000
000000000000000000000000000000000000000000000000000000000000000000
000000000000000000000000000000000000000000000000000000000000000000
000000000000000000000000000000000000000000000000000000000000000011
111111111111111111100000000000000000000000000000000000000000000000
000000000000000000000000000000000000000000000000000000000000000000
000000000000000000000000000000000000000000000000000000000000000000
000000000000000000000000000000000000000000000000000000000000000000
000000000000000000000000000000000000000000000000000000000000000000
000000000000000000000000000000000000000000000000000000000000000000

000000000000000000000000000000000000000000000000000000000000000000
000000000000000000000000000000000000000000000000000000000000000000
000000000000000000000000000000000000000000000000000000000000000000
000000000000000000000000000000000000000000000000000000000000000000
000000000000000000000000000000000000000000000000000000000000000000
000000000000000000000000000000000000000000000000000000000000000
> Q02880 TOP2B_HUMAN
MAKSGGCGAGAGVGGGNGALTWVTLFDQNNAAKKEESETANKNDSSKKLSVERVYQKKTQLEHILL
RPDTYIGSVEPLTQFMWVYDEDVGMNCREVTFVPGLYKIFDEILVNAADNKQRDKNMTCIKVSIDP
ESNIISIWNNGKGIPVVEHKVEKVYVPALIFGQLLTSSNYDDDEKKVTGGRNGYGAKLCNIFSTKF
TVETACKEYKHSFKQTWMNNMMKTSEAKIKHFDGEDYTCITFQPDLSKFKMEKLDKDIVALMTRRA
YDLAGSCRGVKVMFNGKKLPVNGFRSYVDLYVKDKLDETGVALKVIHELANERWDVCLTLSEKGFQ
QISFVNSIATTKGGRHVDYVVDQVVGKLIEVVKKKNKAGVSVKPFQVKNHIWVFINCLIENPTFDS
QTKENMTLQPKSFGSKCQLSEKFFKAASNCGIVESILNWVKFKAQTQLNKKCSSVKYSKIKGIPKL
DDANDAGGKHSLECTLILTEGDSAKSLAVSGLGVIGRDRYGVFPLRGKILNVREASHKQIMENAEI
NNIIKIVGLQYKKSYDDAESLKTLRYGKIMIMTDQDQDGSHIKGLLINFIHHNWPSLLKHGFLEEF
ITPIVKASKNKQELSFYSIPEFDEWKKHIENQKAWKIKYYKGLGTSTAKEAKEYFADMERHRILFR
YAGPEDDAAITLAFSKKKIDDRKEWLTNFMEDRRQRRLHGLPEQFLYGTATKHLTYNDFINKELIL
FSNSDNERSIPSLVDGFKPGQRKVLFTCFKRNDKREVKVAQLAGSVAEMSAYHHGEQALMMTIVNL
AQNFVGSNNINLLQPIGQFGTRLHGGKDAASPRYIFTMLSTLARLLFPAVDDNLLKFLYDDNQRVE
PEWYIPIIPMVLINGAEGIGTGWACKLPNYDAREIVNNVRRMLDGLDPHPMLPNYKNFKGTIQELG
QNQYAVSGEIFVVDRNTVEITELPVRTWTQVYKEQVLEPMLNGTDKTPALISDYKEYHTDTTVKFV
VKMTEEKLAQAEAAGLHKVFKLQTTLTCNSMVLFDHMGCLKKYETVQDILKEFFDLRLSYYGLRKE
WLVGMLGAESTKLNNQARFILEKIQGKITIENRSKKDLIQMLVQRGYESDPVKAWKEAQEKAAEED
ETQNQHDDSSSDSGTPSGPDFNYILNMSLWSLTKEKVEELIKQRDAKGREVNDLKRKSPSDLWKED
LAAFVEELDKVESQEREDVLAGMSGKAIKGKVGKPKVKKLQLEETMPSPYGRRIIPEITAMKADAS
KKLLKKKKGDLDTAAVKVEFDEEFSGAPVEGAGEEALTPSVPINKGPKPKREKKEPGTRVRKTPTS
SGKPSAKKVKKRNPWSDDESKSESDLEETEPVVIPRDSLLRRAAAERPKYTFDFSEEEDDDADDDD
DDNNDLEELKVKASPITNDGEDEFVPSDGLDKDEYTFSPGKSKATPEKSLHDKKSQDFGNLFSFPS
YSQKSEDDSAKFDSNEEDSASVFSPSFGLKQTDKVPSKTVAAKKGKPSSDTVPKPKRAPKQKKVVE
AVNSDSDSEFGIPKKTTTPKGKGRGAKKRKASGSENEGDYNPGRKTSKTTSKKPKKTSFDQDSDVD
IFPSDFPTEPPSLPRTGRARKEVKYFAESDEEEDDVDFAMFN
000000000000000000000000000000000000000000000000000000000000000000
000000000000000000000000000000000000000000000000000000000000000000
000000000000000000000000000000000000000000000000000000000000000000
000000000000000000000000000000000000000000000000000000000000000000
000000000000000000000000000000000000000000000000000000000000000000
000000000000000000000000000000000000000000000000000000000000000000

000000000000000000000000000000000000000000000000000000000000000000
000000000000000000000000000000000000000000000000000000000000000000
000000000000000000000000000000000000000000000000000000000000000000
000000000000000000000000000000000000000000000000000000000000000000
000000000000000000000000000000000000000000000000000000000000000000
000000000000000000000000000000000000000000000000000000000000000000
000000000000000000000000000000000000000000000000000000000000000000
000000000000000000000000000000000000000000000000111111111111111111
110000000000000000000000000000000000000000000000000000000000000000
000000000000000000000000000000000000000000000000000000000000000000
000000000000000000000000000000000000000000000000000000000000000000
000000000000000000000000000000000000000000000000000000000000000000
000000000000000000000000000000000000000000000000000000000000000000
000000000000000000000000000000000000000000000000000000000000000000
000000000000000000000000000000000000000000000000000000000000000000
000000000000000000000000000000000000000000000000000000000000000000
000000000000000000000000000000000000000000000000000000000000000000
000000000000000000000000000000000000000000000000000000000000000000
000000000000000000000000000000000000000000

> P26664 POLG_HCV1

STNPKPQKKNKRNTNRRPQDVKFPGGGQIVGGVYLLPRRGPRLGVRATRKTSERSQPRGRRQPIPK
ARRPEGRTWAQPGYPWPLYGNEGCGWAGWLLSPRGSRPSWGPTDPRRRSRNLGKVIDTLTCGFADL
MGYIPLVGAPLGGAARALAHGVRVLEDGVNYATGNLPGCSFSIFLLALLSCLTVPASAYQVRNSTG
LYHVTNDCPNSSIVYEAADAILHTPGCVPCVREGNASRCWVAMTPTVATRDGKLPATQLRRHIDLL
VGSATLCSALYVGDLCGSVFLVGQLFTFSPRRHWTTQGCNCSIYPGHITGHRMAWDMMMNWSPTTA
LVMAQLLRIPQAILDMIAGAHWGVLAGIAYFSMVGNWAKVLVVLLLFAGVDAETHVTGGSAGHTVS
GFVSLLAPGAKQNVQLINTNGSWHLNSTALNCNDSLNTGWLAGLFYHHKFNSSGCPERLASCRPLT
DFDQGWGPISYANGSGPDQRPYCWHYPPKPCGIVPAKSVCGPVYCFTPSPVVVGTTDRSGAPTYSW
GENDTDVFVLNNTRPPLGNWFGCTWMNSTGFTKVCGAPPCVIGGAGNNTLHCPTDCFRKHPDATYS
RCGSGPWITPRCLVDYPYRLWHYPCTINYTIFKIRMYVGGVEHRLEAACNWTRGERCDLEDRDRSE
LSPLLLTTTQWQVLPCSFTTLPALSTGLIHLHQNIVDVQYLYGVGSSIASWAIKWEYVVLLFLLLA
DARVCSCLWMMLLISQAEAALENLVILNAASLAGTHGLVSFLVFFCFAWYLKGKWVPGAVYTFYGM
WPLLLLLLALPQRAYALDTEVAASCGGVVLVGLMALTLSPYYKRYISWCLWWLQYFLTRVEAQLHV
WIPPLNVRGGRDAVILLMCAVHPTLVFDITKLLLAVFGPLWILQASLLKVPYFVRVQGLLRFCALA
RKMIGGHYVQMVIIKLGALTGTYVYNHLTPLRDWAHNGLRDLAVAVEPVVFSQMETKLITWGADTA
ACGDIINGLPVSARRGREILLGPADGMVSKGWRLLAPITAYAQQTRGLLGCIITSLTGRDKNQVEG
EVQIVSTAAQTFLATCINGVCWTVYHGAGTRTIASPKGPVIQMYTNVDQDLVGWPAPQGSRSLTPC
TCGSSDLYLVTRHADVIPVRRRGDSRGSLLSPRPISYLKGSSGGPLLCPAGHAVGIFRAAVCTRGV

AKAVDFIPVENLETTMRSPVFTDNSSPPVVPQSFQVAHLHAPTGSGKSTKVPAAYAAQGYKVLVLN
PSVAATLGFGAYMSKAHGIDPNIRTGVRTITTGSPITYSTYGKFLADGGCSGGAYDIIICDECHST
DATSILGIGTVLDQAETAGARLVVLATATPPGSVTVPHPNIEEVALSTTGEIPFYGKAIPLEVIKG
GRHLIFCHSKKKCDELAAKLVALGINAVAYYRGLDVSVIPTSGDVVVVATDALMTGYTGDFDSVID
CNTCVTQTVDFSLDPTFTIETITLPQDAVSRTQRRGRTGRGKPGIYRFVAPGERPSGMFDSSVLCE
CYDAGCAWYELTPAETTVRLRAYMNTPGLPVCQDHLEFWEGVFTGLTHIDAHFLSQTKQSGENLPY
LVAYQATVCARAQAPPPSWDQMWKCLIRLKPTLHGPTPLLYRLGAVQNEITLTHPVTKYIMTCMSA
DLEVVTSTWVLVGGVLAALAAYCLSTGCVVIVGRVVLSGKPAIIPDREVLYREFDEMEECSQHLPY
IEQGMMLAEQFKQKALGLLQTASRQAEVIAPAVQTNWQKLETFWAKHMWNFISGIQYLAGLSTLPG
NPAIASLMAFTAAVTSPLTTSQTLLFNILGGWVAAQLAAPGAATAFVGAGLAGAAIGSVGLGKVLI
DILAGYGAGVAGALVAFKIMSGEVPSTEDLVNLLPAILSPGALVVGVVCAAILRRHVGPGEGAVQW
MNRLIAFASRGNHVSPTHYVPESDAAARVTAILSSLTVTQLLRRLHQWISSECTTPCSGSWLRDIW
DWICEVLSDFKTWLKAKLMPQLPGIPFVSCQRGYKGVWRVDGIMHTRCHCGAEITGHVKNGTMRIV
GPRTCRNMWSGTFPINAYTTGPCTPLPAPNYTFALWRVSAEEYVEIRQVGDFHYVTGMTTDNLKCP
CQVPSPEFFTELDGVRLHRFAPPCKPLLREEVSFRVGLHEYPVGSQLPCEPEPDVAVLTSMLTDPS
HITAEAAGRRLARGSPPSVASSSASQLSAPSLKATCTANHDSPDAELIEANLLWRQEMGGNITRVE
SENKVVILDSFDPLVAEEDEREISVPAEILRKSRRFAQALPVWARPDYNPPLVETWKKPDYEPPVV
HGCPLPPPKSPPVPPPRKKRTVVLTESTLSTALAELATRSFGSSSTSGITGDNTTTSSEPAPSGCP
PDSDAESYSSMPPLEGEPGDPDLSDGSWSTVSSEANAEDVVCCSMSYSWTGALVTPCAAEEQKLPI
NALSNSLLRHHNLVYSTTSRSACQRQKKVTFDRLQVLDSHYQDVLKEVKAAASKVKANLLSVEEAC
SLTPPHSAKSKFGYGAKDVRCHARKAVTHINSVWKDLLEDNVTPIDTTIMAKNEVFCVQPEKGGRK
PARLIVFPDLGVRVCEKMALYDVVTKLPLAVMGSSYGFQYSPGQRVEFLVQAWKSKKTPMGFSYDT
RCFDSTVTESDIRTEEAIYQCCDLDPQARVAIKSLTERLYVGGPLTNSRGENCGYRRCRASGVLTT
SCGNTLTCYIKARAACRAAGLQDCTMLVCGDDLVVICESAGVQEDAASLRAFTEAMTRYSAPPGDP
PQPEYDLELITSCSSNVSVAHDGAGKRVYYLTRDPTTPLARAAWETARHTPVNSWLGNIIMFAPTL
WARMILMTHFFSVLIARDQLEQALDCEIYGACYSIEPLDLPPIIQRLHGLSAFSLHSYSPGEINRV
AACLRKLGVPPLRAWRHRARSVRARLLARGGRAAICGKYLFNWAVRTKLKLTPIAAAGQLDLSGWF
TAGYSGGDIYHSVSHARPRWIWFCLLLLAAGVGIYLLPNR
000000000000000000000000000000000000000000000000000000000000000000
000000000000000000000000000000000000000000000000000000000000000000
000000000000000000000000000000000000000000000000000000000000000000
000000000000000000000000000000000000000000000000000000000000000000
0000000000000000000000000000000000000000000000000111111111110000000
000000000000000000000000000000000000000000000000000000000000000000
000000000000000000000000000000000000000000000000000000000000000000
000000000000000000000000000000000000000000000000000000000000000000
000000000000000000000000000000000000000000000000000000000000000000
000000000000000000000000000000000000000000000000000000000000000000

000000000000000000000111111111000000000000000000000000000000000000
000000000000000000000000000000000000000000000000000000000000000000
000000000000000000000000000000000000000000000000000000000000000000
000000000000000000000000000000000000000000000000000000000000000000
000000000000000000000000000000000000000000000000000000000000000000
000000000000000000000000000000000000000000000000000000000000000000
000000000000000000000000000000000000000000000000000000000000000000
000000000000000000000000000000000000000000000000000000000000000000
000000000000000000000000000000000000000000000000000000000000000000
000000000000000000000000000000000000000000000000000000000000000000
000000000000000000000000000000000000000000000000000000000000000000
000000000000000000000000000000000000000000000000000000000000000000
000000000000000000000000000000000000000000000000000000000000000000
000000000000000000000000000000000000000000000000000000000000000000
000000000000000000000000000000000000000000000000000000000000000000
000000000000000000000000000000000000000000000000000000000000000000
000000000000000000000000000000000000000000000000000000000000000000
000000000000000000000000000000000000000000000000000000000000000000
000000000000000000000000000000000000000000000000000000000000000000
000000000000000000000000000000000000000000000000000000000000000000
000000000000000000000000000000000000000000000000000000000000000000
000000000000000000000000000000000000000000000000000000000000000000
000000000000000000000000000000000000000000000000000000000000000000
000000000000000000000000000000000000000000000000000000000000000000
000000000000000000000000000000000000000000000000000000000000000000
000000000000000000000000000000000000000000000000000000000000000000
000000000000000000000000000000000000000000000000000000000000000000
000000000000000000000000000000000000000000000000000000000000000000
000000000000000000000000000000000000000000000000000000000000000000
000000000000000000000000000000000000000000000000000000000000000000
000000000000000000000000000000000000000000000000000000000000000000
000000000000000000000000000000000000000000000000000000000000000000
000000000000000000000000000000000000000000000000000000000000000000
000000000000000000000000000000000000000000000000000000000000000000
000000000000000000000000000000000000000000000000000000000000000000
0000000000000000000000000000000000000000
> P15941 MUC1_HUMAN
MTPGTQSPFFLLLLLTVLTVVTGSGHASSTPGGEKETSATQRSSVPSSTEKNAVSMTSSVLSSHSP

GSGSSTTQGQDVTLAPATEPASGSAATWGQDVTSVPVTRPALGSTTPPAHDVTSAPDNKPAPGSTA
PPAHGVTSAPDTRPAPGSTAPPAHGVTSAPDTRPAPGSTAPPAHGVTSAPDTRPAPGSTAPPAHGV
TSAPDTRPAPGSTAPPAHGVTSAPDTRPAPGSTAPPAHGVTSAPDTRPAPGSTAPPAHGVTSAPDT
RPAPGSTAPPAHGVTSAPDTRPAPGSTAPPAHGVTSAPDTRPAPGSTAPPAHGVTSAPDTRPAPGS
TAPPAHGVTSAPDTRPAPGSTAPPAHGVTSAPDTRPAPGSTAPPAHGVTSAPDTRPAPGSTAPPAH
GVTSAPDTRPAPGSTAPPAHGVTSAPDTRPAPGSTAPPAHGVTSAPDTRPAPGSTAPPAHGVTSAP
DTRPAPGSTAPPAHGVTSAPDTRPAPGSTAPPAHGVTSAPDTRPAPGSTAPPAHGVTSAPDTRPAP
GSTAPPAHGVTSAPDTRPAPGSTAPPAHGVTSAPDTRPAPGSTAPPAHGVTSAPDTRPAPGSTAPP
AHGVTSAPDTRPAPGSTAPPAHGVTSAPDTRPAPGSTAPPAHGVTSAPDTRPAPGSTAPPAHGVTS
APDTRPAPGSTAPPAHGVTSAPDTRPAPGSTAPPAHGVTSAPDTRPAPGSTAPPAHGVTSAPDTRP
APGSTAPPAHGVTSAPDTRPAPGSTAPPAHGVTSAPDTRPAPGSTAPPAHGVTSAPDTRPAPGSTA
PPAHGVTSAPDTRPAPGSTAPPAHGVTSAPDTRPAPGSTAPPAHGVTSAPDTRPAPGSTAPPAHGV
TSAPDTRPAPGSTAPPAHGVTSAPDTRPAPGSTAPPAHGVTSAPDTRPAPGSTAPPAHGVTSAPDT
RPAPGSTAPPAHGVTSAPDNRPALGSTAPPVHNVTSASGSASGSASTLVHNGTSARATTTPASKST
PFSIPSHHSDTPTTLASHSTKTDASSTHHSSVPPLTSSNHSTSPQLSTGVSFFFLSFHISNLQFNS
SLEDPSTDYYQELQRDISEMFLQIYKQGGFLGLSNIKFRPGSVVVQLTLAFREGTINVHDVETQFN
QYKTEAASRYNLTISDVSVSDVPFPFSAQSGAGVPGWGIALLVLVCVLVALAIVYLIALAVCQCRR
KNYGQLDIFPARDTYHPMSEYPTYHTHGRYVPPSSTDRSPYEKVSAGNGGSSLSYTNPAVAAASAN
L
000000000000000000000000000000000000000000000000000000000000000000
000000000000000000000000000000000000000000000000000000000001111111
111111111111111111100000000000000000000000000000000000000000000000
000000000000000000000000000000000000000000000000000000000000000000
000000000000000000000000000000000000000000000000000000000000000000
000000000000000000000000000000000000000000000000000000000000000000
000000000000000000000000000000000000000000000000000000000000000000
000000000000000000000000000000000000000000000000000000000000000000
000000000000000000000000000000000000000000000000000000000000000000
000000000000000000000000000000000000000000000000000000000000000000
000000000000000000000000000000000000000000000000000000000000000000
000000000000000000000000000000000000000000000000000000000000000000
000000000000000000000000000000000000000000000000000000000000000000
000000000000000000000000000000000000000000000000000000000000000000
000000000000000000000000000000000000000000000000000000000000000000
000000000000000000000000000000000000000000000000000000000000000000
000000000000000000000000000000000000000000000000000000000000000000
000000000000000000000000000000000000000000000000000000000000000000
000000000000000000000000000000000000000000000000000000000000000000

0

> P04932 MSP1_PLAFK

MKIIFFLCSFLFFIINTQCVTHESYQELVKKLEALEDAVLTGYSLFHKEKMILNEEEITTKGASAQ
SGTSGTSGTSGPSGPSGTSPSSRSNTLPRSNTSSGASPPADASDSDAKSYADLKHRVRNYLLTIKE
LKYPQLFDLTNHMLTLCDNIHGFKYLIDGYEEINELLYKLNFYFDLLRAKLNDVCANDYCQIPFNL
KIRANELDVLKKLVFGYRKPLDNIKDNVGKMEDYIKKNKKTIENINELIEESKKTIDKNKNATKEE
EKKKLYQAQYDLSIYNKQLEEAHNLISVLEKRIDTLKKNENIKELLDKINEIKNPPPANSGNTPNT
LLDKNKKIEEHEKEIKEIAKTIKFNIDSLFTDPLELEYYLREKNKNIDISAKVETKESTEPNEYPN
GVTYPLSYNDINNALNELNSFGDLINPFDYTKEPSKNIYTDNERKKFINEIKEKIKIEKKKIESDK
KSYEDRSKSLNDITKEYEKLLNEIYDSKFNNNIDLTNFEKMMGKRYSYKVEKLTHHNTFASYENSK
HNLEKLTKALKYMEDYSLRNIVVEKELKYYKNLISKIENEIETLVENIKKDEEQLFEKKITKDENK
PDEKILEVSDIVKVQVQKVLLMNKIDELKKTQLILKNVELKHNIHVPNSYKQENKQEPYYLIVLKK
EIDKLKVFMPKVESLINEEKKNIKTEGQSDNSEPSTEGEITGQATTKPGQQAGSALEGDSVQAQAQ
EQKQAQPPVPVPVPEAKAQVPTPPAPVNNKTENVSKLDYLEKLYEFLNTSYICHKYILVSHSTMNE
KILKQYKITKEEESKLSSCDPLDLLFNIQNNIPVMYSMFDSLNNSLSQLFMEIYEKEMVCNLYKLK
DNDKIKNLLEEAKKVSTSVKTLSSSSMQPLSLTPQDKPEVSANDDTSHSTNLNNSLKLFENILSLG
KNKNIYQELIGQKSSENFYEKILKDSDTFYNESFTNFVKSKADDINSLNDESKRKKLEEDINKLKK
TLQLSFDLYNKYKLKLERLFDKKKTVGKYKMQIKKLTLLKEQLESKLNSLNNPKHVLQNFSVFFNK
KKEAEIAETENTLENTKILLKHYKGLVKYYNGESSPLKTLSEESIQTEDNYASLENFKVLSKLEGK
LKDNLNLEKKKLSYLSSGLHHLIAELKEVIKNKNYTGNSPSENNTDVNNALESYKKFLPEGTDVAT
VVSESGSDTLEQSQPKKPASTHVGAESNTITTSQNVDDEVDDVIIVPIFGESEEDYDDLGQVVTGE
AVTPSVIDNILSKIENEYEVLYLKPLAGVYRSLKKQLENNVMTFNVNVKDILNSRFNKRENFKNVL
ESDLIPYKDLTSSNYVVKDPYKFLNKEKRDKFLSSYNYIKDSIDTDINFANDVLGYYKILSEKYKS
DLDSIKKYINDKQGENEKYLPFLNNIETLYKTVNDKIDLFVIHLEAKVLNYTYEKSNVEVKIKELN
YLKTIQDKLADFKKNNNFVGIADLSTDYNHNNLLTKFLSTGMVFENLAKTVLSNLLDGNLQGMLNI
SQHQCVKKQCPQNSGCFRHLDEREECKCLLNYKQEGDKCVENPNPTCNENNGGCDADAKCTEEDSG
SNGKKITCECTKPDSYPLFDGIFCSSSNFLGISFLLILMLILYSFI

000000000000000000000000000000000000000000000000000000000000000000
000000000000000000000000000000000000000000000000000000000000000000
000000000000000000000000000000000000000000000000000000000000000000
000000000000000000000000000000000000000000000000000000000000000000
000000000000000000000000000000000000000000000000000000000000000000
000000000000000000000000000000000000000000000000000000000000000000
000000000000000000000000000000000000000000000000000000000000000000
000000000000000000000000000000000000000000000000000000000000000000
000000000000000000000000000000000000000000000000000000000000000000
000000000000000000000000000000000000000000000000000000000000000000
000000000000000000000000000000000000000000000000000000000000000000

000000000000000000000000000000000000000000000000000000000000000000
000000000000000000000000000000000000000000000000000000000000000000
000000000000000000000000000000000000000000000000000000000000000000
000000000000000000000000000000000000000000000000000000000000000000
000000000000000000000000000000000000000000000000000000000000000000
000000000000000000000000000000000000000000000000000000000000000000
000000000000000000000000000000000000000000000000000000000000000000
000000000000000000000000000000000000000000000000000000000000000000
000000000000000000000000000000000000000000000000000000000000000000
000000000000000000000000000000000000000000000000000000000000000000
000000000000000000000000000000000000000000000000000000000000000000
000000000000000000000000000000000000000000000000000000000000000000
000000000000111111111111111111100000000000000000000000000000000000
0000001111111111111111111111111111100000000000
> P28481 CO2A1_MOUSE
MIRLGAPQSLVLLTLLIAAVLRCQGQDAQEAGSCLQNGQRYKDKDVWKPSSCRICVCDTGNVLCDD
IICEDPDCLNPEIPFGECCPICPADLATASGRKLGPKGQKGEPGDIRDAPGPRGRDGEPGTPGNP
GPAGPPGPPGPPGLSAGNFAAQMAGGYDEKAGGAQMGVMQGPMGPMGPRGPPGPAGAPGPQGFQGN
PGEPGEPGVSGPMGPRGPPGPAGKPGDDGEAGKPGKSGERGLPGPMGARGFPGTPGLPGVKGHRGY
PGLDGAKGEAGAPGVKGESGSPGENGSPGPMGPRGLPGERGRTGPAGAAGARGNDGQPGPAGPPGP
VGPAGGPGFPGAPGAKGEAGPTGARGPEGAQGSRGEPGNPGSPGPAGASGNPGTDGIPGAKGSAGA
PGIAGAPGFPGPRGPPGPQGATGPLGPKGQAGEPGIAGFKGDQGPKGETGPAGPQGAPGPAGEEGK
RGARGEPGGAGPIGPPGERGAPGNRGFPGQDGLAGPKGAPGERGPSGLAGPKGANGDPGRPGEPGL
PGARGLTGRPGDAGPQGKVGPSGAPGEDGRPGPPGPQGARGQPGVMGFPGPKGANGEPGKAGEKGL
AGAPGLRGLPGKDGETGAAGPPGPSGPAGERGEQGAPGPSGFQGLPGPPGPPGEGGKQGDQGIPGE
AGAPGLVGPRGERGFPGERGSPGAQGLQGPRGLPGTPGTDGPKGAAGPDGPPGAQGPPGLQGMPGE
RGAAGIAGPKGDRGDVGEKGPEGAPGKDGGRGLTGPIGPPGPAGANGEKGEAGPPGPSGSTGARGA
PGEPGETGPPGPAGFAGPPGADGQPGAKGDQGEAGQKGDAGAPGPQGPSGAPGPQGPTGVTGPKGA
RGAQGPPGATGFPGAAGRVGPPGANGNPGPAGPPGPAGKDGPKGVRGDSGPPGRAGDPGLEGPAGA
PGEKGEPGDDGPSGLDGPPGPQGLAGQRGIVGLPGQRGERGFPGLPGPSGEPGKQGAPGASGDRGP
PGPVGPPGLTGPAGEPGREGSPGADGPPGRDGAAGVKGDRGETGALGAPGAPGPPGSPGPAGPTGK
QGDRGEAGAQGPMGPSGPAGARGIAGPQGPRGDKGESGEQGERGLKGHRGFTGLQGLPGPPGPSGD
QGASGPAGPSGPRGPPGPVGPSGKDGSNGIPGPIGPPGPRGRSGETGPVGPPGSPGPPGPPGPPGP
GIDMSAFAGLGQREKGPDPMQYMRADEADSTLRQHDVEVDATLKSLNNQIESIRSPDGSRKNPART
CQDLKLCHPEWKSGDYWIDPNQGCTLDAMKVFCNMETGETCVYPNPATVPRKNWWSSKSKEKKHIW
FGETMNGGFHFSYGDGNLAPNTANVQMTFLRLLSTEGSQNITYHCKNSIAYLDEAAGNLKKALLIQ
GSNDVEMRAEGNSRFTYTALKDGCTKHTGKWGKTVIEYRSQKTSRLPIIDIAPMDIGGAEQEFGVD
IGPVCFL

000000000000000000000000000000000000000000000000000000000000000000
000000000000000000000000000000000000000000000000000000000000000000
000000000000000000000000000000000000000000000000000000000000000000
000000000000000000000000000000000000000000000000000000000000000000
000000000000000000000000000000000000000000000000000000000000000000
001111111100000000000000000000000000000000000000000000000000000000
000000000000000000000000000000000000000000000000000000000000000000
000000000000000000000000000000000000000000000000000000000000000000
000000000000000000000000000000000000000000000000000000000000000000
000000000000000000000000000000000000000000000000000000000000000000
000000000000000000000000000000000000000000000000000000000000000000
000000000000000000000000000000000000000000000000000000000000000000
000000000000000000000000000000000000000000000000000000000000000000
000000000000000000000000000000000000000000000000000000000000000000
000000000000000000000000000000000000000000000000000000000000000000
000000000000000000000000000000000000000000000000000000000000000000
000000000000000000000000000000000000000000000000000000000000000000
000000000000000000000000000000000000000000000000000000000000000000
000000000000000000000000000000000000000000000000000000000000000000
000000000000000000000000000000000000000000000000000000000000000000
000000000000000000000000000000000000000000000000000000000000000000
000000000000000000000000000000000000000000000000000000000000000000
0000000

> P02452 CO1A1_HUMAN
MFSFVDLRLLLLLAATALLTHGQEEGQVEGQDEDIPPITCVQNGLRYHDRDVWKPEPCRICVCDNG
KVLCDDVICDETKNCPGAEVPEGECCPVCPDGSESPTDQETTGVEGPKGDTGPRGPRGPAGPPGRD
GIPGQPGLPGPPGPPGPPGPPGLGGNFAPQLSYGYDEKSTGGISVPGPMGPSGPRGLPGPPGAPGP
QGFQGPPGEPGEPGASGPMGPRGPPGPPGKNGDDGEAGKPGRPGERGPPGPQGARGLPGTAGLPGM
KGHRGFSGLDGAKGDAGPAGPKGEPGSPGENGAPGQMGPRGLPGERGRPGAPGPAGARGNDGATGA
AGPPGPTGPAGPPGFPGAVGAKGEAGPQGPRGSEGPQGVRGEPGPPGPAGAAGPAGNPGADGQPGA
KGANGAPGIAGAPGFPGARGPSGPQGPGGPPGPKGNSGEPGAPGSKGDTGAKGEPGPVGVQGPPGP
AGEEGKRGARGEPGPTGLPGPPGERGGPGSRGFPGADGVAGPKGPAGERGSPGPAGPKGSPGEAGR
PGEAGLPGAKGLTGSPGSPGPDGKTGPPGPAGQDGRPGPPGPPGARGQAGVMGFPGPKGAAGEPGK
AGERGVPGPPGAVGPAGKDGEAGAQGPPGPAGPAGERGEQGPAGSPGFQGLPGPAGPPGEAGKPGE
QGVPGDLGAPGPSGARGERGFPGERGVQGPPGPAGPRGANGAPGNDGAKGDAGAPGAPGSQGAPGL
QGMPGERGAAGLPGPKGDRGDAGPKGADGSPGKDGVRGLTGPIGPPGPAGAPGDKGESGPSGPAGP
TGARGAPGDRGEPGPPGPAGFAGPPGADGQPGAKGEPGDAGAKGDAGPPGPAGPAGPPGPIGNVGA
PGAKGARGSAGPPGATGFPGAAGRVGPPGPSGNAGPPGPPGPAGKEGGKGPRGETGPAGRPGEVGP

PGPPGPAGEKGSPGADGPAGAPGTPGPQGIAGQRGVVGLPGQRGERGFPGLPGPSGEPGKQGPSGA
SGERGPPGPMGPPGLAGPPGESGREGAPGAEGSPGRDGSPGAKGDRGETGPAGPPGAPGAPGAPGP
VGPAGKSGDRGETGPAGPAGPVGPAGARGPAGPQGPRGDKGETGEQGDRGIKGHRGFSGLQGPPGP
PGSPGEQGPSGASGPAGPRGPPGSAGAPGKDGLNGLPGPIGPPGPRGRTGDAGPVGPPGPPGPPGP
PGPPSAGFDFSFLPQPPQEKAHDGGRYYRADDANVVRDRDLEVDTTLKSLSQQIENIRSPEGSRKN
PARTCRDLKMCHSDWKSGEYWIDPNQGCNLDAIKVFCNMETGETCVYPTQPSVAQKNWYISKNPKD
KRHVWFGESMTDGFQFEYGGQGSDPADVAIQLTFLRLMSTEASQNITYHCKNSVAYMDQQTGNLKK
ALLLKGSNEIEIRAEGNSRFTYSVTVDGCTSHTGAWGKTVIEYKTTKTSRLPIIDVAPLDVGAPDQ
EFGFDVGPVCFL
000000000000000000000000000000000000000000000000000000000000000000
000000000000000000000000000000000000000000000000000000000000000000
000000000000000000000000000000000000000000000000000000000000000000
000000000000000000000000000000000000000000000000000000000000000000
000000000000000000000000000000000000000000000000000000000000000000
000000000000000000000000000000000000000000000000000000000000000000
000000001111111111100000000000000000000000000000000000000000000000
000000000000000000000000000000000000000000000000000000000000000000
000000000000000000000000000000000000000000000000000000000000000000
000000000000000000000000000000000000000000000000000000000000000000
000000000000000000000000000000000000000000000000000000000000000000
000000000000000000000000000000000000000000000000000000000000000000
000000000000000000000000000000000000000000000000000000000000000000
000000000000000000000000000000000000000000000000000000000000000000
000000000000000000000000000000000000000000000000000000000000000000
000000000000000000000000000000000000000000000000000000000000000000
000000000000000000000000000000000000000000000000000000000000000000
000000000000000000000000000000000000000000000000000000000000000000
000000000000000000000000000000000000000000000000000000000000000000
000000000000000000000000000000000000000000000000000000000000000000
000000000000000000000000000000000000000000000000000000000000000000
000000000000000000000000000000000000000000000000000000000000000000
000000000000
> P32553 VP2_AHSV4
MASEFGILMTNEKFDPSLEKTICDVIVTKKGRVKHKEVDGVCGYEWDETNHRFGLCEVEHDMSISE
FMYNEIRCEGAYPIFPRYIIDTLKYEKFIDRNDHQIRVDRDDNEMRKILIQPYAGEMYFSPECYPS
VFLRREARSQKLDRIRNYIGKRVEFYEEESKRKAILDQNKMSKVEQWRDAVNERIVSIEPKRGECY
DHGTDIIYQFIKKLRFGMMYPHYYVLHSDYCIVPNKGGTSIGSWHIRKRTEGDAKASAMYSGKGPL
NDLRVKIERDDLSRETIIQIIEYGKKFNSSAGDKQGNISIEKLVEYCDFLTTFVHAKKKEEGEDDT

ARQEIRKAWVKGMPYMDFSKPMKITRGFNRNMLFFAALDSFRKRNGVDVDPNKGKWKEHIKEVTEK
LKKAQTENGGQPCQVSIDGVNVLTNVDYGTVNHWIDWVTDIIMVVQTKRLVKEYAFKKLKSENLLA
GMNSLVGVLRCYMYCLALAIYDFYEGTIDGFKKGSNASAIIETVAQMFPDFRRELVEKFGIDLRMK
EITRELFVGKSMTSKFMEEGEYGYKFAYGWRRDGFAVMEDYGEILTEKVEDLYKGVLLGRKWEDEV
DDPESYFYDDLYTNEPHRVFLSAGKDVDNNITLRSISQAETTYLSKRFVSYWYRISQVEVTKARNE
VLDMNEKQKPYFEFEYDDFKPCSIGELGIHASTYIYQNLLVGRNRGEEILDSKELVWMDMSLLNFG
AVRSHDRCWISSSVAIEVNLRHALIVRIFSRFDMMSERETFSTILEKVMEDVKKLRFFPTYRHYYL
ETLQRVFNDERRLEVDDFYMRLYDVQTREQALNTFTDFHRCVESELLLPTLKLNFLLWIVFEMENV
EVNAAYKRHPLLISTAKGLRVIGVDIFNSQLSISMSGWIPYVERMCAESKVQTKLTADELKLKRWF
ISYYTTLKLDRRAEPRMSFKFEGLSTWIGSNCGGVRDYVIQMLPTRKPKPGALMVVYARDSRIEWI
EAELSQWLQMEGSLGLILVHDSGIINKSVLRARTLKIYNRGSMDTLILISSGVYTFGNKFLLSKLL
AKTE
000000000000000000000000000000000000000000000000000000000000000000
000000000000000000000000000000000000000000000000000000000000000000
000000000000000000000000000000000000000000000000000000000000000000
000000000000000000000000111111111111111111000000000000111111111111
111111111111111111111111111111111111111111111111111111111111111111
111111111111111111110000000000000000000001111111111111111111111111
111100000000000000000000000000000000000000000000000000000000000000
000000000000000000000000000000000000000000000000000000000000000000
000000000000000000000000000000000000000111111111111111110000000000
000000000000000000000000000000000000000000000000000000000000000000
001111111111111111111000000000000000000000000000000000000000000000
000000000000000000000000000000000000000000000000000000000000000000
000000000000000000000000000000000000000000000000000000000000000000
000000000000000000000000000000000000000000000000000000000000000000
000000000000000000000000000000000000000000000000000000000000000000
000000000000000000000000000000000000000000000000000000000000000000
0000
> P21404 POLG_CXA9
GAQVSTQKTGAHETSLSAAGNSIIHYTNINYYKDAASNSANRQDFTQDPSKFTEPVKDVMIKSLPA
LNSPTVEECGYSDRVRSITLGNSTITTQECANVVVGYGRWPTYLRDDEATAEDQPTQPDVATCRFY
TLDSIKWEKGSVGWWWKFPEALSDMGLFGQNMQYHYLGRAGYTIHLQCNASKFHQGCLLVVCVPEA
EMGGAVVGQAFSATAMANGDKAYEFTSATQSDQTKVQTAIHNAGMGVGVGNLTIYPHQWINLRTNN
SATIVMPYINSVPMDNMFRHYNFTLMVIPFVKLDYADTASTYVPITVTVAPMCAEYNGLRLAQAQG
LPTMNTPGSTQFLTSDDFQSPCALPQFDVTPSMNIPGEVKNLMEIAEVDSVVPVNNVQDTTDQMEM
FRIPVTINAPLQQQVFGLRLQPGLDSVFKHTLLGEILNYYAHWSGSMKLTFVFCGSAMATGKFLIA
YSPPGANPPKTRKDAMLGTHIIWDIGLQSSCVLCVPWISQTHYRLVQQDEYTSAGYVTCWYQTGMI

VPPGTPNSSSIMCFASACNDFSVRMLRDTPFISQDNKLQGDVEEAIERARCTVADTMRTGPSNSAS
VPALTAVETGHTSQVTPSDTMQTRHVKNYHSRSESTVENFLGRSACVYMEEYKTTDKHVNKKFVAW
PINTKQMVQMRRKLEMFTYLRFDMEVTFVITSRQDPGTTLAQDMPVLTRQIMYVPPGGPIPAKVDD
YAWQTSTNPSIFWTEGNAPARMSIPFISIGNAYSNFYDGWSNFDQRGSYGYNTLNNLGHIYVRHVS
GSSPHPITSTIRVYFKPKHTRAWVPRPPRLCQYKKAFSVDFTPTPITDTRKDINTVTTVAQSRRRG
DMSTLNTHGAFGQQSGAVYVGNYRVINRHLATHTDWQNCVWEDYNRDLLVSTTTAHGCDVIARCQC
TTGVYFCASKNKHYPVSFEGPGLVEVQESEYYPKRYQSHVLLAAGFSEPGDCGGILRCEHGVIGIV
TMGGEGVVGFADVRDLLWLEDDAMEQGVKDYVEQLGNAFGSGFTNQICEQVNLLKESLVGQDSILE
KSLKALVKIISALVIVVRNHDDLITVTAILALIGCTSSPWRWLKQKVSQYYGIPMAERQNDSWLKK
FTEMTNACKRMEWIAIKIQKFIEWLKVKILPEVREKHEFLNRLKQLPLLESQIATIEQSAPSQSDQ
EQLFSNVQYFAHYCRKYAPLYAAEAKRVFSLEKKMSNYIQFKSKCRIEPVCLLLHGSPGAGKSVAT
NLIGRSLAEKLNSSVYSLPPDPDHFDGYKQQAVVIMDDLCQNPDGKDVSLFCQMVSSVDFVPPMAA
LEEKGILFTSPFVLASTNAGSINAPTVSDSRALARRFHFDMNIEVISMYSQNGKINMPMSVKTCDE
ECCPVNFKKCCPLVCGKAIQFIDRRTQVRYSLDMLVTEMFREYNHRHSVGATLEALFQGPPIYREI
KISVAPETPPPPVIADLLKSVDSEDVREYCKEKGWLIPEVNSTLQIEKYVSRAFICLQAITTFVSV
AGIIYIIYKLFAGFQGAYTGIPNQKPKVPTLRQAKVQGPAFEFAVAMMKRNSSTVKTEYGEFTMLG
IYDRWAVLPRHAKPGPTILMNDQEVGVMDAKELVDKDGTNLELTLLKLNRNEKFRDIRGFLAKEEM
EVNEAVLAINTSKFPNMYIPVGQVTDYGFLNLGGTPTKRMLMYNFPTRAGQCGGVLMSTGKVLGIH
VGGNGHQGFSAALLKHYFNDEQGEIEFIESSKDAGFPIINTPSKTKLEPSVFHQVFEGVKEPAVLR
NGDPRLKANFEEAIFSKYIGNVNTHVDEYMLEAVDHYAGQLATLDISTEPMKLEDAVYGTEGLEAL
DLTTSAGYPYVALGIKKRDILSKKTRDLTKLKECMDKYGLNLPMITYVKDQLRSAEKVAKGKSRLI
EASSLNDSVAMRQTFGNLYKTFHLNPGIVTGSAVGCDPDLFWSKIPVMLNGHLIAFDYSGYDASLS
PVWFACLKLLLEKLGYSHKETNYIDYLCNSHHLYRDKHYFVRGGMPSGCSGTSIFNSMINNIIIRT
LMLKVYKGIDLDQFRMIAYGDDVIASYPWPIDASLLAEAGKDYGLIMTPADKGECFNEVTWTNVTF
LKRYFRADEQYPFLVHPVMPMKDIHESIRWTKDPKNTQDHVRSLCLLAWHNGEHEYEEFIRKIRSV
PVGRCLTLPAFSTLRRKWLDSF
000000000000000000000000000000000000000000000000000000000000000000
000000000000000000000000000000000000000000000000000000000000000000
000000000000000000000000000000000000000000000000000000000000000000
000000000000000000000001111111111111110000000000000000000000000000
000000000000000000000000000000000000000000000000000000000000000001
111111111111111111110000000000001111111111111110001111111111111111
111111111111110000000000000000000000000000000000000000000000000000
000000000000000000000000000000000000000000000000000000000000000000
000000000000000000000000000000000000000111111111111100000001111111
111111111111110000000000000000000000000000000000000000000000000000
000000000000000000000000000000000000000000000000000000000000000000
000000000000000000000000000000000000000000000000000000000000000000

00000000000000000000000000000000000000000000000111111111111111110000000
0011111111100000000000000000000000000000000000000000000000000000000000
0000000000000000000000000000000000000000000000000000000000000000000000
0000000000000000000000000000000000000000000000000000000000000000000000
0000000000000000000000000000000000000000000000000000000000000000000000
0000000000000000000000000000000000000000000000000000000000000000000000
0000000000000000000000000000000000000000000000000000000000000000000000
0000000000000000000000000000000000000000000000000000000000000000000000
0000000000000000000000000000000000000000000000000000000000000000000000
0000000000000000000000000000000000000000000000000000000000000000000000
0000000000000000000000000000000000000000000000000000000000000000000000
0000000000000000000000000000000000000000000000000000000000000000000000
0000000000000000000000000000000000000000000000000000000000000000000000
0000000000000000000000000000000000000000000000000000000000000000000000
0000000000000000000000000000000000000000000000000000000000000000000000
0000000000000000000000000000000000000000000000000000000000000000000000
0000000000000000000000000000000000000000000000000000000000000000000000
0000000000000000000000000000000000000000000000000000000000000000000000
0000000000000000000000000000000000000000000000000000000000000000000000
0000000000000000000000000000000000000000000000000000000000000000000000
0000000000000000000000000000000000000000000000000000000000000000000000
0000000000000000000000

> P03300 POLG_POL1M

GAQVSSQKVGAHENSNRAYGGSTINYTTINYYRDSASNAASKQDFSQDPSKFTEPIKDVLIKTAPM
LNSPNIEACGYSDRVLQLTLGNSTITTQEAANSVVAYGRWPEYLRDSEANPVDQPTEPDVAACRFY
TLDTVSWTKESRGWWWKLPDALRDMGLFGQNMYYHYLGRSGYTVHVQCNASKFHQGALGVFAVPEM
CLAGDSNTTTMHTSYQNANPGEKGGTFTGTFTPDNNQTSPARRFCPVDYLLGNGTLLGNAFVFPHQ
IINLRTNNCATLVLPYVNSLSIDSMVKHNNWGIAILPLAPLNFASESSPEIPITLTIAPMCCEFNG
LRNITLPRLQGLPVMNTPGSNQYLTADNFQSPCALPEFDVTPPIDIPGEVKNMMELAEIDTMIPFD
LSATKKNTMEMYRVRLSDKPHTDDPILCLSLSPASDPRLSHTMLGEILNYYTHWAGSLKFTFLFCG
FMMATGKLLVSYAPPGADPPKKRKEAMLGTHVIWDIGLQSSCTMVVPWISNTTYRQTIDDSFTEGG
YISVFYQTRIVVPLSTPREMDILGFVSACNDFSVRLLRDTTHIEQKALAQGLGQMLESMIDNTVRE
TVGAATSRDALPNTEASGPTHSKEIPALTAVETGATNPLVPSDTVQTRHVVQHRSRSESSIESFFA
RGACVTIMTVDNPASTTNKDKLFAVWKITYKDTVQLRRKLEFFTYSRFDMELTFVVTANFTETNNG
HALNQVYQIMYVPPGAPVPEKWDDYTWQTSSNPSIFYTYGTAPARISVPYVGISNAYSHFYDGFSK
VPLKDQSAALGDSLYGAASLNDFGILAVRVVNDHNPTKVTSKIRVYLKPKHIRVWCPRPPRAVAYY
GPGVDYKDGTLTPLSTKDLTTYGFGHQNKAVYTAGYKICNYHLATQDDLQNAVNVMWSRDLLVTES
RAQGTDSIARCNCNAGVYYCESRRKYYPVSFVGPTFQYMEANNYYPARYQSHMLIGHGFASPGDCG

GILRCHHGVIGIITAGGEGLVAFSDIRDLYAYEEEAMEQGITNYIESLGAAFGSGFTQQISDKITE
LTNMVTSTITEKLLKNLIKIISSLVIITRNYEDTTTVLATLALLGCDASPWQWLRKKACDVLEIPY
VIKQGDSWLKKFTEACNAAKGLEWVSNKISKFIDWLKEKIIPQARDKLEFVTKLRQLEMLENQIST
IHQSCPSQEHQEILFNNVRWLSIQSKRFAPLYAVEAKRIQKLEHTINNYIQFKSKHRIEPVCLLVH
GSPGTGKSVATNLIARAIAERENTSTYSLPPDPSHFDGYKQQGVVIMDDLNQNPDGADMKLFCQMV
STVEFIPPMASLEEKGILFTSNYVLASTNSSRISPPTVAHSDALARRFAFDMDIQVMNEYSRDGKL
NMAMATEMCKNCHQPANFKRCCPLVCGKAIQLMDKSSRVRYSIDQITTMIINERNRRSNIGNCMEA
LFQGPLQYKDLKIDIKTSPPPECINDLLQAVDSQEVRDYCEKKGWIVNITSQVQTERNINRAMTIL
QAVTTFAAVAGVVYVMYKLFAGHQGAYTGLPNKKPNVPTIRTAKVQGPGFDYAVAMAKRNIVTATT
SKGEFTMLGVHDNVAILPTHASPGESIVIDGKEVEILDAKALEDQAGTNLEITIITLKRNEKFRDI
RPHIPTQITETNDGVLIVNTSKYPNMYVPVGAVTEQGYLNLGGRQTARTLMYNFPTRAGQCGGVIT
CTGKVIGMHVGGNGSHGFAAALKRSYFTQSQGEIQWMRPSKEVGYPIINAPSKTKLEPSAFHYVFE
GVKEPAVLTKNDPRLKTDFEEAIFSKYVGNKITEVDEYMKEAVDHYAGQLMSLDINTEQMCLEDAM
YGTDGLEALDLSTSAGYPYVAMGKKKRDILNKQTRDTKEMQKLLDTYGINLPLVTYVKDELRSKTK
VEQGKSRLIEASSLNDSVAMRMAFGNLYAAFHKNPGVITGSAVGCDPDLFWSKIPVLMEEKLFAFD
YTGYDASLSPAWFEALKMVLEKIGFGDRVDYIDYLNHSHHLYKNKTYCVKGGMPSGCSGTSIFNSM
INNLIIRTLLLKTYKGIDLDHLKMIAYGDDVIASYPHEVDASLLAQSGKDYGLTMTPADKSATFET
VTWENVTFLKRFFRADEKYPFLIHPVMPMKEIHESIRWTKDPRNTQDHVRSLCLLAWHNGEEEYNK
FLAKIRSVPIGRALLLPEYSTLYRRWLDSF
000000000000000000000000000000000000000000000000000000000000000000
000000000000000000000000000000000000000000000000000000000000000000
000000000000000000000000000000000000000000000000000000000000000000
000000000000000000000000000000000000000000000000000000000000000000
000000000000000000000000000000000000000000000000000000000000000000
000000000000000000000000000000000000000000000000000000000000000000
000000000000000000000000000000000000000000000000000000000000000000
000000000000000000000000000000000000000000000000000000000000000000
000000000000000000000000000000000000000000000000000000000000000000
000000000000000000000000000000000000000000000000000001111111111100
000000000000000000000000000000000000000000000000000000000000000000
000000000000000000000000000000000000000000000000000000000000000000
000000000000000000000000000000000000000000000000000000000000000000
000000000000000000000000000000000000000000000000000000000000000000
000000000000000000000000000000000000000000000000000000000000000000
000000000000000000000000000000000000000000000000000000000000000000
000000000000000000000000000000000000000000000000000000000000000000
000000000000000000000000000000000000000000000000000000000000000000
000000000000000000000000000000000000000000000000000000000000000000

000000000000000000000000000000000000000000000000000000000000000000
000000000000000000000000000000000000000000000000000000000000000000
000000000000000000000000000000000000000000000000000000000000000000
000000000000000000000000000000000000000000000000000000000000000000
000000000000000000000000000000000000000000000000000000000000000000
000000000000000000000000000000000000000000000000000000000000000000
000000000000000000000000000000000000000000000000000000000000000000
000000000000000000000000000000000000000000000000000000000000000000
000000000000000000000000000000000000000000000000000000000000000000
000000000000000000000000000000000000000000000000000000000000000000
000000000000000000000000000000000000000000000000000000000000000000
000000000000000000000000000000000000000000000000000000000000000000
000000000000000000000000000000000000000000000000000000000000000000
000000000000000000000000000000000000000000000000000000000000000000
000000000000000000000000000000
> P10845 BXA1_CLOBO
PFVNKQFNYKDPVNGVDIAYIKIPNVGQMQPVKAFKIHNKIWVIPERDTFTNPEEGDLNPPPEAKQ
VPVSYYDSTYLSTDNEKDNYLKGVTKLFERIYSTDLGRMLLTSIVRGIPFWGGSTIDTELKVIDTN
CINVIQPDGSYRSEELNLVIIGPSADIIQFECKSFGHEVLNLTRNGYGSTQYIRFSPDFTFGFEES
LEVDTNPLLGAGKFATDPAVTLAHELIHAGHRLYGIAINPNRVFKVNTNAYYEMSGLEVSFEELRT
FGGHDAKFIDSLQENEFRLYYYNKFKDIASTLNKAKSIVGTTASLQYMKNVFKEKYLLSEDTSGKF
SVDKLKFDKLYKMLTEIYTEDNFVKFFKVLNRKTYLNFDKAVFKINIVPKVNYTIYDGFNLRNTNL
AANFNGQNTEINNMNFTKLKNFTGLFEFYKLLCVRGIITSKTKSLDKGYNKALNDLCIKVNNWDLF
FSPSEDNFTNDLNKGEEITSDTNIEAAEENISLDLIQQYYLTFNFDNEPENISIENLSSDIIGQLE
LMPNIERFPNGKKYELDKYTMFHYLRAQEFEHGKSRIALTNSVNEALLNPSRVYTFFSSDYVKKVN
KATEAAMFLGWVEQLVYDFTDETSEVSTTDKIADITIIIPYIGPALNIGNMLYKDDFVGALIFSGA
VILLEFIPEIAIPVLGTFALVSYIANKVLTVQTIDNALSKRNEKWDEVYKYIVTNWLAKVNTQIDL
IRKKMKEALENQAEATKAIINYQYNQYTEEEKNNINFNIDDLSSKLNESINKAMININKFLNQCSV
SYLMNSMIPYGVKRLEDFDASLKDALLKYIYDNRGTLIGQVDRLKDKVNNTLSTDIPFQLSKYVDN
QRLLSTFTEYIKNIINTSILNLRYESNHLIDLSRYASKINIGSKVNFDPIDKNQIQLFNLESSKIE
VILKNAIVYNSMYENFSTSFWIRIPKYFNSISLNNEYTIINCMENNSGWKVSLNYGEIIWTLQDTQ
EIKQRVVFKYSQMINISDYINRWIFVTITNNRLNNSKIYINGRLIDQKPISNLGNIHASNNIMFKL
DGCRDTHRYIWIKYFNLFDKELNEKEIKDLYDNQSNSGILKDFWGDYLQYDKPYYMLNLYDPNKYV
DVNNVGIRGYMYLKGPRGSVMTTNIYLNSSLYRGTKFIIKKYASGNKDNIVRNNDRVYINVVVKNK
EYRLATNASQAGVEKILSALEIPDVGNLSQVVVMKSKNDQGITNKCKMNLQDNNGNDIGFIGFHQF
NNIAKLVASNWYNRQIERSSRTLGCSWEFIPVDDGWGERPL
000000000000000000000000000000000000000000000000000000000000000000
000000000000000000000000000000000000000000000000000000000000000000

000000000000000000000000000000000000000000000000000000000000000000
000000000000000000000000000000000000000000000000000000000000000000
000000000000000000000000000000000000000000000000000000000000000000
000000000000000000000000000000000000000000000000000000000000000000
000000000000000000000000000000000000000000000000000000000000000001
111111111111111111000000000000000000000000000000000000011111111111
111111111111111111111111111111111111111111111111110000000001111111
111111111111000000000000000000000000000000000000000000000000000111
111111111111111100000000000000000000000000000000000000000000000000
000000000000000111111111111111111100000000000000000000000111111111
111111111100000000011111111111111111111111111111111100000000011111
111111111111111111111111111111111111111111000000000000000000000001
111111111111111111111111111111110000000001111111111111111111111111
111111111111111111111100000000000000000000000000000000000001111111
111111111111000000000000000000000000000000000000000000000000000111
111111111111111111111100000000000000000000000000000001111111111111
111111000000000000000000000000000000000000000000000000000000000000
00000111111111111111111111111111111111111

> P03305 POLG_FMDVO

MNTTDCFIALVQAIREIKALFLSRTTGKMELTLYNGEKKTFYSRPNNHDNCWLNAILQLFRYVEEP
FFDWVYSSPENLTLEAIKQLEDLTGLELHEGGPPALVIWNIKHLLHTGIGTASRPSEVCMVDGTDM
CLADFHAGIFLKGQEHAVFACVTSNGWYAIDDEDFYPWTPDPSDVLVFVPYDQEPLNGEWKAKVQR
KLKGAGQSSPATGSQNQSGNTGSIINNYYMQQYQNSMDTQLGDNAISGGSNEGSTDTTSTHTTNTQ
NNDWFSKLASSAFSGLFGALLADKKTEETTLLEDRILTTRNGHTTSTTQSSVGVTYGYATAEDFVS
GPNTSGLETRVVQAERFFKTHLFDWVTSDSFGRCHLLELPTDHKGVYGSLTDSYAYMRNGWDVEVT
AVGNQFNGGCLLVAMVPELYSIQKRELYQLTLFPHQFINPRTNMTAHITVPFVGVNRYDQYKVHKP
WTLVVMVVAPLTVNTEGAPQIKVYANIAPTNVHVAGEFPSKEGIFPVACSDGYGGLVTTDPKTADP
VYGKVFNPPRNQLPGRFTNLLDVAEACPTFLRFEGGVPYVTTKTDSDRVLAQFDMSLAAKQMSNTF
LAGLAQYYTQYSGTINLHFMFTGPTDAKARYMVAYAPPGMEPPKTPEAAAHCIHAEWDTGLNSKFT
FSIPYLSAADYAYTASGVAETTNVQGWVCLFQITHGKADGDALVVLASAGKDFELRLPVDARAETT
SAGESADPVTTTVENYGGETQIQRRQHTDVSFIMDRFVKVTPQNQINILDLMQIPSHTLVGALLRA
STYYFSDLEIAVKHEGDLTWVPNGAPEKALDNTTNPTAYHKAPLTRLALPYTAPHRVLATVYNGEC
RYNRNAVPNLRGDLQVLAQKVARTLPTSFNYGAIKATRVTELLYRMKRAETYCPRPLLAIHPTEAR
HKQKIVAPVKQTLNFDLLKLAGDVESNPGPFFFSDVRSNFSKLVETINQMQEDMSTKHGPDFNRLV
SAFEELAIGVKAIRTGLDEAKPWYKLIKLLSRLSCMAAVAARSKDPVLVAIMLADTGLEILDSTFV
VKKISDSLSSLFHVPAPVFSFGAPVLLAGLVKVASSFFRSTPEDLERAEKQLKARDINDIFAILKN
GEWLVKLILAIRDWIKAWIASEEKFVTMTDLVPGILEKQRDLNDPSKYKEAKEWLDNARQACLKSG
NVHIANLCKVVAPAPSKSRPEPVVVCLRGKSGQGKSFLANVLAQAISTHFTGRIDSVWYCPPDPDH

FDGYNQQTVVVMDDLGQNPDGKDFKYFAQMVSTTGFIPPMASLEDKGKPFNSKVIIATTNLYSGFT
PRTMVCPDALNRRFHFDIDVSAKDGYKINSKLDIIKALEDTHANPVAMFQYDCALLNGMAVEMKRM
QQDMFKPQPPLQNVYQLVQEVIDRVELHEKVSSHPIFKQISIPSQKSVLYFLIEKGQHEAAIEFFE
GMVHDSIKEELRPLIQQTSFVKRAFKRLKENFEIVALCLTLLANIVIMIRETRKRQKMVDDAVNEY
IEKANITTDDKTLDEAEKSPLETSGASTVGFRERTLPGQKACDDVNSEPAQPVEEQPQAEGPYAGP
LERQKPLKVRAKLPQQEGPYAGPMERQKPLKVKAKAPVVKEGPYEGPVKKPVALKVKAKNLIVTES
GAPPTDLQKMVMGNTKPVELILDGKTVAICCATGVFGTAYLVPRHLFAEKYDKIMVDGRAMTDSDY
RVFEFEIKVKGQDMLSDAALMVLHRGNRVRDITKHFRDTARMKKGTPVVGVINNADVGRLIFSGEA
LTYKDIVVCMDGDTMPGLFAYRAATKAGYCGGAVLAKDGADTFIVGTHSAGGNGVGYCSCVSRSML
LKMKAHIDPEPHHEGLIVDTRDVEERVHVMRKTKLAPTVAHGVFNPEFGPAALSNKDPRLNEGVVL
DEVIFSKHKGDTKMSEEDKALFRRCAADYASRLHSVLGTANAPLSIYEAIKGVDGLDAMEPDTAPG
LPWALQGKRRGALIDFENGTVGPEVEAALKLMEKREYKFVCQTFLKDEIRPLEKVRAGKTRIVDVL
PVEHILYTRMMIGRFCAQMHSNNGPQIGSAVGCNPDVDWQRFGTHFAQYRNVWDVDYSAFDANHCS
DAMNIMFEEVFRTEFGFHPNAEWILKTLVNTEHAYENKRITVGGGMPSGCSATSIINTILNNIYVL
YALRRHYEGVELDTYTMISYGDDIVVASDYDLDFEALKPHFKSLGQTITPADKSDKGFVLGHSITD
VTFLKRHFHMDYGTGFYKPVMASKTLEAILSFARRGTIQEKLISVAGLAVHSGPDEYRRLFEPFQG
LFEIPSYRSLYLRWVNAVCGDA
000000000000000000000000000000000000000000000000000000000000000000
000000000000000000000000000000000000000000000000000000000000000000
000000000000000000000000000000000000000000000000000000000000000000
000000000000000000000000000000000000000000000000000000000000000000
000000000000000000000000000000000000000000000000000000000000000000
000000000000000000000000000000000000000000000000000000000000000000
000000000000000000000000000000000000000000000000000000000000000000
000000000000000000000000000000000000000000000000000000000000000000
000000000000000000000000000000000000000000000000000000000000000000
000000000000000000000000000000000000000000000000000000000000000000
000000000000000000000000000000000000000000000000000000000000000000
000000000000000000000000000000000000000000000000000000000000000000
000000000000000000000000000000000000000000000000000000000000000000
000000000111111111111111100000000000000000000000000000000000000000
000011111111100000000000000000000000000000000000000000000000000000
000000000000000000000000000000000000000000000000000000000000000000
000000000000000000000000000000000000000000000000000000000000000000
000000000000000000000000000000000000000000000000000000000000000000
000000000000000000000000000000000000000000000000000000000000000000
000000000000000000000000000000000000000000000000000000000000000000
000000000000000000000000000000000000000000000000000000000000000000

000000000000000000000000000000000000000000000000000000000000000000
000000000000000000000000000000000000000000000000000000000000000000
000000000000000000000000000000000000000000000000000000000000000000
000000000000000000000000000000000000000000000000000000000000000000
000000000000000000000000000000000000000000000000000000000000000000
000000000000000000000000000000000000000000000000000000000000000000
000000000000000000000000000000000000000000000000000000000000000000
000000000000000000000000000000000000000000000000000000000000000000
000000000000000000000000000000000000000000000000000000000000000000
000000000000000000000000000000000000000000000000000000000000000000
000000000000000000000000000000000000000000000000000000000000000000
000000000000000000000000000000000000000000000000000000000000000000
000000000000000000000000000000000000000000000000000000000000000000
000000000000000000000000000000000000000000000000000000000000000000
0000000000000000000000
