# Supplementary material for: Prediction of B-cell epitopes using evolutionary information and propensity scales
Source: BMC Bioinformatics. 2013 Jan 21;14(Suppl 2):S10. doi: 10.1186/1471-2105-14-S2-S10 (PMC3549808; doi:10.1186/1471-2105-14-S2-S10)
Supplement: Additional file 4 — The HIV dataset. [file 1471-2105-14-S2-S10-S4.pdf]

Additional file 4. HIV dataset.

>288 Integrase
FLDGIDKAQDEHEKYHSNWRAMASDFNLPPVVAKEIVASCDKCQLKGEAMHGQVDCSPGIWQLDCT
HLEGKVILVAVHVASGYIEAEVIPAETGQETAYFLLKLAGRWPVKTIHTDNGSNFTGATVRAACWW
AGIKQEFGIPYNPQSQGVVESMNKELKKIIGQVRDQAEHLKTAVQMAVFIHNFKRKGGIGGYSAGE
RIVDIIATDIQTKELQKQITKIQNFRVYYRDSRNPLWKGPAKLLWKGEGAVVIQDNSDIKVVPRRK
AKIIRDYGKQMAGDDCVASRQDED
111111111111111100000000111111111110000001111111111111100000000000
000000000000000000000000000000000000000000000000000000000000000000
000000000000000000111111111111000000000000000000000000000000000000
000000000000111111111111111111111111111111111111111111111111111111
111111111100000000000000
>206 sp_P03406_NEF_HV1BR
MGGKWSKSSVVGWPTVRERMRRAEPAADGVGAASRDLEKHGAITSSNTAATNAACAWLEAQEEEEV
GFPVTPQVPLRPMTYKAAVDLSHFLKEKGGLEGLIHSQRRQDILDLWIYHTQGYFPDWQNYTPGPG
VRYPLTFGWCYKLVPVEPDKVEEANKGENTSLLHPVSLHGMDDPEREVLEWRFDSRLAFHHVAREL
HPEYFKNC
000000000011111111111111000000111111111100000000111111111111111111
111111111111110011111100000000000000000000000000000111111111111111
100000000000000111111111111111111111111111111111111111111110001111
11111111
>99 Protease
PQVTLWQRPLVTIKIGGQLKEALLDTGADDTVLEEMSLPGRWKPKMIGGIGGFIKVRQYDQILIEI
CGHKAIGTVLVGPTPVNIIGRNLLTQIGCTLNF
111111000000000000000000000000000000011111110000000000000000000000
000000000000000000000000000000000
>560 RT
PISPIETVPVKLKPGMDGPKVKQWPLTEEKIKALVEICTEMEKEGKISKIGPENPYNTPVFAIKKK
DSTKWRKLVDFRELNKRTQDFWEVQLGIPHPAGLKKKKSVTVLDVGDAYFSVPLDEDFRKYTAFTI
PSINNETPGIRYQYNVLPQGWKGSPAIFQSSMTKILEPFRKQNPDIVIYQYMDDLYVGSDLEIGQH
RTKIEELRQHLLRWGLTTPDKKHQKEPPFLWMGYELHPDKWTVQPIVLPEKDSWTVNDIQKLVGKL
NWASQIYPGIKVRQLCKLLRGTKALTEVIPLTEEAELELAENREILKEPVHGVYYDPSKDLIAEIQ
KQGQGQWTYQIYQEPFKNLKTGKYARMRGAHTNDVKQLTEAVQKITTESIVIWGKTPKFKLPIQKE
TWETWWTEYWQATWIPEWEFVNTPPLVKLWYQLEKEPIVGAETFYVDGAANRETKLGKAGYVTNRG
RQKVVTLTDTTNQKTELQAIYLALQDSGLEVNIVTDSQYALGIIQAQPDQSESELVNQIIEQLIKK
EKVYLAWVPAHKGIGGNEQVDKLVSAGIRKVL
000000000000000000000000000000000000000000000000000000000000000011

111111100000000000000000000000000000000000000000000000000000000000
000000000000000000000000000000000000000000000000000000000000000000
000000000000000000000000000000000000000000000000001111111111111110
000000000000000000000000000001111111111000000000000000000000000000
000000000000000000011111000000000000000000000111111110000000000000
000000000000000000000000000000000000000000000111111111000000000000
000000000000000000000000000000000000000000000000000000000011111111
11100001111111111111100000000000
>116 sp_P04618_REV_HV1H2
MAGRSGDSDEELIRTVRLIKLLYQSNPPPNPEGTRQARRNRRRRWRERQRQIHSISERILGTYLGR
SAEPVPLQLPPLERLTLDCNEDCGTSGTQGVGSPQILVESPTVLESGTKE
000000000000000000000000000000000000011111100000000000000000000000
00011111111111111111111111000011111111111111111111111
>86 sp_P04608_TAT_HV1H2
MEPVDPRLEPWKHPGSQPKTACTNCYCKKCCFHCQVCFITKALGISYGRKKRRQRRRAHQNSQTHQ
ASLSKQPTSQPRGDPTGPKE
011111111111111000000000000000000000000000000111111111111111111000000
00000000011111111111
>856 sp_P04578_ENV_HV1H2
MRVKEKYQHLWRWGWRWGTMLLGMLMICSATEKLWVTVYYGVPVWKEATTTLFCASDAKAYDTEVH
NVWATHACVPTDPNPQEVVLVNVTENFNMWKNDMVEQMHEDIISLWDQSLKPCVKLTPLCVSLKCT
DLKNDTNTNSSSGRMIMEKGEIKNCSFNISTSIRGKVQKEYAFFYKLDIIPIDNDTTSYKLTSCNT
SVITQACPKVSFEPIPIHYCAPAGFAILKCNNKTFNGTGPCTNVSTVQCTHGIRPVVSTQLLLNGS
LAEEEVVIRSVNFTDNAKTIIVQLNTSVEINCTRPNNNTRKRIRIQRGPGRAFVTIGKIGNMRQAH
CNISRAKWNNTLKQIASKLREQFGNNKTIIFKQSSGGDPEIVTHSFNCGGEFFYCNSTQLFNSTWF
NSTWSTEGSNNTEGSDTITLPCRIKQIINMWQKVGKAMYAPPISGQIRCSSNITGLLLTRDGGNSN
NESEIFRPGGGDMRDNWRSELYKYKVVKIEPLGVAPTKAKRRVVQREKRAVGIGALFLGFLGAAGS
TMGAASMTLTVQARQLLSGIVQQQNNLLRAIEAQQHLLQLTVWGIKQLQARILAVERYLKDQQLLG
IWGCSGKLICTTAVPWNASWSNKSLEQIWNHTTWMEWDREINNYTSLIHSLIEESQNQQEKNEQEL
LELDKWASLWNWFNITNWLWYIKLFIMIVGGLVGLRIVFAVLSIVNRVRQGYSPLSFQTHLPTPRG
PDRPEGIEEEGGERDRDRSIRLVNGSLALIWDDLRSLCLFSYHRLRDLLLIVTRIVELLGRRGWEA
LKYWWNLLQYWSQELKNSAVSLLNATAIAVAEGTDRVIEVVQGACRAIRHIPRRIRQGLERILL
000000000000000000000000000000000000000011111111111111111111111111
111111111111111111111111111111000011111111111111111111011111111111
111111111100000000000000000001111111101111111111100000000000000000
000000000000011111111111111111111000001111111110000001111111111100
000000111111111100000000000001111111111111111111111111111111111100
000000000000000000000000000000000000000000000000011111111111111011

111100000000000000000001111111111111111111100000000000000000000111
111111100111111000000000000000011111111111111111100000000000001111
111111111111111000000000000000000000000000000000111111111111101111
111111111111000000000000000000000000000000000000011111111111111111
111111100000000000000000000000000000000000000000000000000000000001
111111111100111110000000000000000000000000000000000000000000000000
0000000000000000000000000000000000000000000000000000000000000000
>132 p17
MGARASVLSGGELDRWEKIRLRPGGKKKYKLKHIVWASRELERFAVNPGLLETSEGCRQILGQLQP
SLQTGSEELRSLYNTVATLYCVHQRIEIKDTKEALDKIEEEQNKSKKKAQQAAADTGHSNQVSQNY
000000000011111111111111111111111111110000000000001111111111111111
111111111111100000000000000000000111111000000011111111111111111111
>231 p24
PIVQNIQGQMVHQAISPRTLNAWVKVVEEKAFSPEVIPMFSALSEGATPQDLNTMLNTVGGHQAAM
QMLKETINEEAAEWDRVHPVHAGPIAPGQMREPRGSDIAGTTSTLQEQIGWMTNNPPIPVGEIYKR
WIILGLNKIVRMYSPTSILDIRQGPKEPFRDYVDRFYKTLRAEQASQEVKNWMTETLLVQNANPDC
KTILKALGPAATLEEMMTACQGVGGPGHKARVL
000000000000000000001111100000000000000000001111100000000000000000
000011111111111111111111111111111110000000000000000000111111111100
000000000011111100011111111111111111110000000000000000000000000000
000000000000000000001111111111111
>137 p2p7p1p6
AEAMSQVTNSATIMMQRGNFRNQRKIVKCFNCGKEGHTARNCRAPRKKGCWKCGKEGHQMKDCTER
QANFLGKIWPSYKGRPGNFLQSRPEPTAPPEESFRSGVETTTPPQKQEPIDKELYPLTSLRSLFGN
DPSSQ
111110000000000000111111111100000000000000001111111111000000000001
111111111111111111110000000000000000000000000000000000000000000000
00000
