# Supplementary material for: Prediction of B-cell epitopes using evolutionary information and propensity scales
Source: BMC Bioinformatics. 2013 Jan 21;14(Suppl 2):S10. doi: 10.1186/1471-2105-14-S2-S10 (PMC3549808; doi:10.1186/1471-2105-14-S2-S10)
Supplement: Additional file 5 — The Pellequer dataset. [file 1471-2105-14-S2-S10-S5.pdf]

Additional file 5. Pellequer dataset.

>124 CHO sp|P01556|CHTB_VIBCH Cholera enterotoxin, beta chain precursor - Vibrio cholerae.
MIKLKFGVFFTVLLSSAYAHGTPQNITDLCAEYHNTQIYTLNDKIFSYTESLAGKREMAIITFKNG
AIFQVEVPGSQHIDSQKKAIERMKDTLRIAYLTEAKVEKLCVWNNKTPHAIAAISMAN
000000000000000000000000000000001111111111000000000011111111100000
0011111111111111100000000000000000000111111111111111000000
>104 CYT sp|P00001|CYC_HUMAN Cytochrome c - Homo sapiens (Human), and Pan troglodytes (Chimpanzee).
GDVEKGKKIFIMKCSQCHTVEKGGKHKTGPNLHGLFGRKTGQAPGYSYTAANKNKGIIWGEDTLME
YLENPKKYIPGTKMIFVGIKKKEERADLIAYLKKATNE
111100000001111111111110000000000000000000011111111110001111111111
11111110000000000000000000000000000000
>389 HBV sp|P03138|VMSA_HPBVY Major surface antigen precursor - Hepatitis B virus (subtype ayw).
MGQNLSTSNPLGFFPDHQLDPAFRANTANPDWDFNPNKDTWPDANKVGAGAFGLGFTPPHGGLLGW
SPQAQGILQTLPANPPPASTNRQSGRQPTPLSPPLRNTHPQAMQWNSTTFHQTLQDPRVRGLYFPA
GGSSSGTVNPVLTTASPLSSIFSRIGDPALNMENITSGFLGPLLVLQAGFFLLTRILTIPQSLDSW
WTSLNFLGGTTVCLGQNSQSPTSNHSPTSCPPTCPGYRWMCLRRFIIFLFILLLCLIFLLVLLDYQ
GMLPVCPLIPGSSTTSTGPCRTCMTTAQGTSMYPSCCCTKPSDGNCTCIPIPSSWAFGKFLWEWAS
ARFSWLSLLVPFVQWFVGLSPTVWLSVIWMMWYWGPSLYSILSPFLPLLPIFFCLWVYI
000000000000000000000000000000000000000000000000000000000000000000
000000000000000000000000000000000000000000000000000000000000000000
000000000000000000000000000000001111111111111110000011111111111111
000000000000111111111111111111000111111111110000000000000001111111
111111110000000000000001111111111111111111111100000000000000000000
00000000000000000000000000000000000000000000000000000000000
>165 HCG sp|P01233|CGHB_HUMAN Choriogonadotropin beta chain precursor (Chorionic gonadotrophin beta subunit) (CG-beta) - Homo sapiens (Human).
MEMFQGLLLLLLLSMGGTWASKEPLRPRCRPINATLAVEKEGCPVCITVNTTICAGYCPTMTRVLQ
GVLPALPQVVCNYRDVRFESIRLPGCPRGVNPVVSYAVALSCQCALCRRSTTDCGGPKDHPLTCDD
PRFQDSSSSKAPPPSLPSPSRLPGPSDTPILPQ
000000000000000000001111111000000000000000000000000000000001111111
111111000000000000000000000000000000000000000111111111111110000111
111111111100000000001111111111111
>187 IFB sp|P01574|INB_HUMAN Interferon beta precursor (IFN-beta)

(Fibroblast interferon) - Homo sapiens (Human).
MTNKCLLQIALLLCFSTTALSMSYNLLGFLQRSSNFQCQKLLWQLNGRLEYCLKDRMNFDIPEEIK
QLQQFQKEDAALTIYEMLQNIFAIFRQDSSSTGWNETIVENLLANVYHQINHLKTVLEEKLEKEDF
TRGKLMSSLHLKRYYGRILHYLKAKEYSHCAWTIVRVEILRNFYFINRLTGYLRN
000000000000000000000000000111111111111111100000000000000000111111
110000000000000000000000000111111111000000000000000000000001111111
1000011100000000000000000000000000000000000000000000000
>143 LEG sp|P02238|LGBA_SOYBN Leghemoglobin A (Nodulin 2) - Glycine max (Soybean).
VAFTEKQDALVSSSFEAFKANIPQYSVVFYTSILEKAPAAKDLFSFLANGVDPTNPKLTGHAEKLF
ALVRDSAGQLKASGTVVADAALGSVHAQKAVTDPQFVVVKEALLKTIKAAVGDKWSDELSRAWEVA
YDELAAAIKKA
000000000000001111111110000000000000000000000000000111111110000000
000000000000000000000000011111100000000011111111100000000000000001
11111111110
>147 LYS sp|P00698|LYC_CHICK Lysozyme C precursor (EC 3.2.1.17) (1,4-beta-N-acetylmuramidase C) (Allergen Gal d 4) (Gal d IV) - Gallus gallus (Chicken).
MRSLLILVLCFLPLAALGKVFGRCELAAAMKRHGLDNYRGYSLGNWVCAAKFESNFNTQATNRNTD
GSTDYGILQINSRWWCNDGRTPGSRNLCNIPCSALLSSDITASVNCAKKIVSDGNGMNAWVAWRNR
CKGTDVQAWIRGCRL
000000000000000000000000000000000000000000000000000000011111111111
111111000000000111111111111111110000000000000000000000000000000000
000000000000000
>118 MHR sp|P02247|HEMM_THEZO Myohemerythrin (MHR) - Themiste zostericola.
GWEIPEPYVWDESFRVFYEQLDEEHKKIFKGIFDCIRDNSAPNLATLVKVTTNHFTHEEAMMDAAK
YSEVVPHKKMHKDFLEKIGGLSAPVDAKNVDYCKEWLVNHIKGTDFKYKGKL
000111111000000111111000000000000000111111111100000001111100001111
1111110000000111111000011111100000000000000111111000
>153 MYO sp|P02185|MYG_PHYCA Myoglobin - Physeter catodon (Sperm whale) (Physeter macrocephalus).
VLSEGEWQLVLHVWAKVEADVAGHGQDILIRLFKSHPETLEKFDRFKHLKTEAEMKASEDLKKHGV
TVLTALGAILKKKGHHEAELKPLAQSHATKHKIPIKYLEFISEAIIHVLHSRHPGDFGADAQGAMN
KALELFRKDIAAKYKELGYQG
111111000000111111111100000000000000000000000000111111111111110000
000000000000000000000000000111110000000000000011111101111111000000
000000000000111111100

>185 PIL sp|P04127|PAPA_ECOLI PAP fimbrial major pilin protein precursor (PAP pili) - Escherichia coli.
MIKSVIAGAVAMAVVSFGVNNAAPTIPQGQGKVTFNGTVVDAPCSISQKSADQSIDFGQLSKSFLE
AGGVSKPMDLDIELVNCDITAFKGGNGAKKGTVKLAFTGPIVNGHSDELDTNGGTGTAIVVQGAGK
NVVFDGSEGDANTLKDGENVLHYTAVVKKSSAVGAAVTEGAFSAVANFNLTYQ
000000000000000000000000001111111100000000000000000000000001111111
111111000000000000001111111111100000000000000000111111111111111111
111111001111111111111000000000000000000000000000000000
>189 RAS sp|P01112|RASH_HUMAN Transforming protein p21/H-RAS-1 (C-H-RAS) - Homo sapiens (Human).
MTEYKLVVVGAGGVGKSALTIQLIQNHFVDEYDPTIEDSYRKQVVIDGETCLLDILDTAGQEEYSA
MRDQYMRTGEGFLCVFAINNTKSFEDIHQYREQIKRVKDSDDVPMVLVGNKCDLAARTVESRQAQD
LARSYGIPYIETSAKTRQGVEDAFYTLVREIRQHKLRKLNPPDESGPGCMSCKCVLS
111111111111111111000000000011111111111111110000000000000000000111
111111111100000000000000111111111111111111000000000000000000000000
000000000000000000000000000000000000000000000000000000000
>406 REN sp|P00797|RENI_HUMAN Renin precursor, renal (EC 3.4.23.15) (Angiotensinogenase) - Homo sapiens (Human).
MDGWRRMPRWGLLLLLWGSCTFGLPTDTTTFKRIFLKRMPSIRESLKERGVDMARLGPEWSQPMKR
LTLGNTTSSVILTNYMDTQYYGEIGIGTPPQTFKVVFDTGSSNVWVPSSKCSRLYTACVYHKLFDA
SDSSSYKHNGTELTLRYSTGTVSGFLSQDIITVGGITVTQMFGEVTEMPALPFMLAEFDGVVGMGF
IEQAIGRVTPIFDNIISQGVLKEDVFSFYYNRDSENSQSLGGQIVLGGSDPQHYEGNFHYINLIKT
GVWQIQMKGVSVGSSTLLCEDGCLALVDTGASYISGSTSSIEKLMEALGAKKRLFDYVVKCNEGPT
LPDISFHLGGKEYTLTSADYVFQESYSSKKLCTLAIHAMDIPPPTGPTWALGATFIRKFYTEFDRR
NNRIGFALAR
000000000000000000000000000000000000000000000000000000000000000000
000000000000000000000000000000000000000000000000011111111111001111
111110000000001111111111000000000000000000000000000111111110000000
111111111111000000000000000001111111100000000001111111110000000000
000000000000111111111111110000000000000000000000111111111000000000
000000000000000000000011111111000001111111111100000000000000000000
0000000000
>85 SCO sp|P01484|SCX2_ANDAU Neurotoxin II precursor (Aah II) - Androctonus australis hector (Sahara scorpion).
MNYLVMISLALLFVTGVESVKDGYIVDDVNCTYFCGRNAYCNEECTKLKGESGYCQWASPYGNACY
CYKLPDHVRTKGPGRCHGR
000000000000000000011111111111100000000000000111111111000111111100
0000111111111000000

>158 TMV sp|P03570|COAT_TMV Coat protein - Tobacco mosaic virus (vulgare) (TMV), Tobacco mosaic virus (strain B935A) (TMV), and Tobacco mosaic virus (strain Korean NC 82) (TMV).

SYSITTPSQFVFLSSAWADPIELINLCTNALGNQFQTQQARTVVQRQFSEVWKPSPQVTVRFPDSD
FKVYRYNAVLDPLVTALLGAFDTRNRIIEVENQANPTTAETLDATRRVDDATVAIRSAINNLIVEL
IRGTGSYNRSSFESSSGLVWTSGPAT

111111111100000000111111111111110111111000000000000000111111111111
110000000111111111111100000000000000111111111100000000000000000000
01111111111111001111111111
