# Supplementary material for: Prediction of B-cell epitopes using evolutionary information and propensity scales
Source: BMC Bioinformatics. 2013 Jan 21;14(Suppl 2):S10. doi: 10.1186/1471-2105-14-S2-S10 (PMC3549808; doi:10.1186/1471-2105-14-S2-S10)
Supplement: Additional file 6 — The PC dataset. [file 1471-2105-14-S2-S10-S6.pdf]

Additional file 6. PC dataset.

>sp|P04156|PRIO_HUMAN Major prion protein OS=Homo sapiens GN=PRNP PE=1 SV=1
MANLGCWMLVLFVATWSDLGLCKKRPKPGGWNTGGSRYPGQGSPGGNRYPPQGGGGWGQPHGGGWG
QPHGGGWGQPHGGGWGQPHGGGWGQGGGTHSQWNKPSKPKTNMKHMAGAAAAGAVVGGLGGYMLGS
AMSRPIIHFGSDYEDRYYRENMHRYPNQVYYRPMDEYSNQNNFVHDCVNITIKQHTVTTTTKGENF
TETDVKMMERVVEQMCITQYERESQAYYQRGSSMVLFSSPPVILLISFLIFLIVG
000000000000000000000000000000000000111111000000000111000000000000
000000000000000000000000111110000111111111111110000000001111111110
000000011111100000000000000111111110000000000000000000000000000001
1111000000000000000000000000000000000000000000000000000
>sp|P20287|G3P_SCHMA Glyceraldehyde-3-phosphate dehydrogenase OS=Schistosoma mansoni PE=2 SV=1
MSRAKVGINGFGRIGRLVLRAAFLKNTVDVVSVNDPFIDLEYMVYMIKRDSTHGTFPGEVSTENGK
LKVNGKLISVHCERDPANIPWDKDGAEYVVESTGVFTTIDKAQAHIKNNRAKKVIISAPSADAPMF
VVGVNENSYEKSMSVVSNASCTTNCLAPLAKVIHDKFEIVEGLMTTVHSFTATQKVVDGPSSKLWR
DGRGAMQNIIPASTGAAKAVGKVIPALNGKLTGMAFRVPTPDVSVVDLTCRLGKGASYEEIKAAVK
AAASGPLKGILEYTEDEVVSSDFVGSTSSSIFDAKAGISLNNNFVKLVSWYDNEFGYSCRVVDLIT
HMHKVDHA
000000000000000000000000000000111111111111100000000000000111111111
111111111111111100000000000000000111111111111111000000000000000000
000000000000000000000000000000111111111110000000000000000000000000
000000000000000000000000000000000000000000000000000000000000000000
000111111111111111111111100000000000000000000000000000000000000000
00000000
>sp|P43238|ALL12_ARAHY Allergen Ara h 1, clone P41B OS=Arachis hypogaea PE=1 SV=1
MRGRVSPLMLLLGILVLASVSATHAKSSPYQKKTENPCAQRCLQSCQQEPDDLKQKACESRCTKLE
YDPRCVYDPRGHTGTTNQRSPPGERTRGRQPGDYDDDRRQPRREEGGRWGPAGPREREREEDWRQP
REDWRRPSHQQPRKIRPEGREGEQEWGTPGSHVREETSRNNPFYFPSRRFSTRYGNQNGRIRVLQR
FDQRSRQFQNLQNHRIVQIEAKPNTLVLPKHADADNILVIQQGQATVTVANGNNRKSFNLDEGHAL
RIPSGFISYILNRHDNQNLRVAKISMPVNTPGQFEDFFPASSRDQSSYLQGFSRNTLEAAFNAEFN
EIRRVLLEENAGGEQEERGQRRWSTRSSENNEGVIVKVSKEHVEELTKHAKSVSKKGSEEEGDITN
PINLREGEPDLSNNFGKLFEVKPDKKNPQLQDLDMMLTCVEIKEGALMLPHFNSKAMVIVVVNKGT
GNLELVAVRKEQQQRGRREEEEDEDEEEEGSNREVRRYTARLKEGDVFIMPAAHPVAINASSELHL
LGFGINAENNHRIFLAGDKDNVIDQIEKQAKDLAFPGSGEQVEKLIKNQKESHFVSARPQSQSQSP
SSPEKESPEKEDQEEENQGGKGPLLSILKAFN

000000000000000000000000011111111000000000000001111111111000000001
111111100000000000000001111111111111111001111111100000000111111110
011111111111111111100000000000000000000000000000000000000000000000
000000000000000000000000000000000000000000000000000000000000000000
000000000000000000000000000000111111110000000001111111100000111111
111100000000001111111100000000000000000000000000000000000000001111
111111000000111111111100000000000000000000000000000000000000000000
111111000000000000000000000000000001111111111000000000000000001111
111111000011111111110011111111111111111000000000000000000000000000
00000000000000000000000000000000
>tr|Q19QW0|Q19QW0_CVHSA Nucleoprotein OS=Human SARS coronavirus GN=N PE=3 SV=1
MSDNGPQSNQRSAPRITFGGPTDSTDNNQNGGRNGARPKQRRPQGLPNNTASWFTALTQHGKEELR
FPRGQGVPINTNSGPDDQIGYYRRATRRVRGGDGKMKELSPRWYFYYLGTGPEASLPYGANKEGII
WVATEGALNTPKDHIGTRNPNNNAATVLQLPQGTTLPKGFYAEGSRGGSQASSRSSSRSRGNSRNS
TPGSSRGNSPARMASGGGETALALLLLDRLNQLESKVSGKGQQQQGQTVTKKSAAEASKKPRQKRT
ATKQYNVTQAFGRRGPEQTQGNFGDQDLIRQGTDYKHWPQIAQFAPSASAFFGMSRIGMEVTPSGT
WLTYHGAIKLDDKDPQFKDNVILLNKHIDAYKTFPPTEPKKDKKKKTDEAQPLPQRQKKQPTVTLL
PAADMDDFSRQLQNSMSGASADSTQA
000000000000000000000000000000000001111111111111111111100000000000
000000000000000000000000000000000000000000000000000000000000000000
000000000000000000000001111111111111111111111111000000000000000000
000000000000000000000000000000000000000000000000000000000000000000
011111111111111100000111111111111111000000000000000000000000000000
000000000000000000000000011111111111111111111000000000011111111111
11111111111111000000000000
>tr|O77685|O77685_TRIVU Zona pellucida 3 protein OS=Trichosurus vulpecula GN=ZP3 PE=2 SV=1
SKALSWATQSPAPGSSFSPPPVVAVQCEDDRLVVSVNRDFFGTGRLVQAAELTLGPSACAPVPAEP
LSKRVVFEVGLHECGSELQMTPDSLIYSTVLHYAPNLSQSPLVLRSSPVSVPIQCQYPRRDNVSSR
GIQPTWVPFHSTLSREQRLKFSLRLMADDWSTERTSSAFQLGDLIHIQADVYTGYHVPLRLFVDSC
TATLTPDPASVPYHVVIDFNGCLVDGQSRDSSSIFISPRPGQNVLRFLVDSFRFAQDSRNEIYITC
HLKVTATDQAPSPLNKACSYNSTADEWLPVEGPRDICSCCQTGTCISLSSSRKRSLADQEPGNPSE
FEADLMLGPLVLSEAENGPKSGQGNNVGGIPKWPELLLGLTVGIAATVCLLLGLIIGSHKFGSPCS
RNV
000000011111111111111111000000000000000000000001111111111111111111
111000000001111111111111111111111000111111111111000000000000000000
111111111111111110000000000000000000000000000000000000000000000000

00000000000000000000000000000111111111100000000000000000000000000
000111111111110000000000111111111110000000000000000000000001111111
111111111111111111000000000000000000000000000000000000000000000000
000
>tr|A3KF09|A3KF09_I56A1 Haemagglutinin OS=Influenza A virus (strain A/Duck/Czechoslovakia/1956 H4N6) GN=HA PE=3 SV=1
QNYTGNPVICMGHHAVANGTMVKTLADDQVEVVTAQELVESQNLPELCPSPLRLVDGQTCDIINGA
LGSPGCDHLNGAEWDVFIERPNAVDTCYPFDVPEYQSLRSILANNGKFEFIAEEFQWNTVKQNGKS
GACKRANVNDFFNRLNWLVKSDGNAYPLQNLTKINNGDYARLYIWGVHHPSTDTEQTNLYKNNPGG
VTVSTKTSQTSVVPNIGSRPLVRGQSGRVSFYWTIVEPGDLIVFNTIGNLIAPRGHYKLNNQKKST
ILNTAIPIGSCVSKCHTDKGSLSTTKPFQNISRIAVGDCPRYVKQGSLKLATGMRNIPEKASRGLF
GAIAGFIENGWQGLIDGWYGFRHQNAEGTGTAADLKSTQAAIDQINGKLNRLIEKTNDKYHQIEKE
FEQVEGRIQDLEKYVEDTKIDLWSYNAELLVALENQHTIDVTDSEMNKLFERVRRQLRENAEDKGN
GCFEIFHKCDNNCIESIRNGTYDHDIYRDEAINNRFQIQGVKLTQGYMD
000000000000000000000000000000000000000000000000000000000000000000
000000000000000000000000111111111111111000000000000001111111111111
111111111111111111111111111111111111111111111111111111000111111111
111111000111111111111111111111111111000000111111111111111111111111
111111111111111111111111111111111111111111111111111111111111111111
111000000000000000000000000000000000000000000000000000000000000000
000000000000000000000000000000000000000000111111111111111000111111
1111111111110000000000000000000001111111111111110
>tr|A5HNY9|A5HNY9_9INFA Hemagglutinin OS=Influenza A virus (A/tufted duck/Switzerland/V504/2006(H5N1)) GN=HA PE=3 SV=1
MEKIVLLLAIVSLVKSDQICIGYHANNSTEQVDTIMEKNVTVTHAQDILEKTHNGKLCDLDGVKPL
ILRDCSVAGWLLGNPMCDEFLNVPEWSYIVEKINPANDLCYPGNFNDYEELKHLLSRINHFEKIQI
IPKSSWSDHEASSGVSSACPYQGRSSFFRNVVWLIKKDNAYPTIKRSYNNTNQEDLLVLWGIHHPN
DAAEQTRLYQNPTTYISVGTSTLNQRLVPKIATRSKVNGQSGRMEFFWTILKPNDAINFESNGNFI
APENAYKIVKKGDSTIMKSELEYGNCNTKCQTPIGAINSSMPFHNIHPLTIGECPKYVKSNRLVLA
TGLRNSPQGERRRKKRGLFGAIAGFIEGGWQGMVDGWYGYHHSNEQGSGYAADKESTQKAIDGVTN
KVNSIIDKMNTQFEAVGREFNNLERRIENLNKKMEDGFLDVWTYNAELLVLMENERTLDFHDSNVK
NLYDKVRLQLRDNAKELGNGCFEFYHRCDNECMESVKNGTYDYPQYSEEARLKREEISGVKLESIG
TYQILSIYSTVASSLALAIMVAGLSLWMCSNGSLQCRICI
000000000000000000000000111111111111111111111111111111111000000000
000000000000000000111111111111111111111111000000000000000000000000
000000000000000000111111111111111000000000000000000000000000000000
111111111111110000000000000000000111111111111111111111111111111111
111111111000000000000000000000000000000000000000000000000000000000

000000000000000000000000000000000000000000000000000000000000000000
000000000000000000000000000000000000000000000000000000000000000000
000000000111111111111111111000000000000000000000000000000000000000
0000000000000000000000000000000000000000
>tr|C7FPM3|C7FPM3_9INFA Hemagglutinin OS=Influenza A virus (A/mallard/Switzerland/WV4060166/2006(H12N2)) GN=HA PE=3 SV=1
MEKFIVLSIILTTGLAYDKICIGYQTNNSTDTVNTLIEQNVPVTQVEELVHGQVNPILCSTELGSP
LVLDDCSLEGLILGNPKCDLYLNGREWSYIVERPKEMEGICYPGSIENQEELRSLFSSIKKYERVK
MFDFTKWNVTYTGTSKACNNTSNQGSFYRSMRWLTLKSGQFPVQTDEYKNTRDSDIVFTWAIHHPP
TSDEQVKLYKNPNSLSSVTTDEINRSFRPNIGPRPLIRGQQGRMDYYWAVLKPGQTVKIQTNGNLI
APEYGHLITGKSHGRILKNDLPIGQCTTECQLNEGVMNTSKPFQNTSKHYIGKCPKYIPSNSLKLA
IGLRNVPQAQDRGLFGAIAGFIEGGWPGLVAGWYGFQHQNSEGTGIAADRDSTQKAIDNMQNKLNN
VIDKMNKQFEVVKHEFSEVESRINMINSKIDDQITDIWAYNAELLVLLENQKTLDEHDANVRNLHD
RVRRVLKENAIDTGDGCFEILHKCDDGCMDTIKNGTYNHQDYEEESKLERQRINGVKLEENSTYK
000000000000000000000000000000111111111111111111111000000000000000
000000000000000000000000000000000000000000000000000000000000111111
111111111111111111111111111111111111111111111111000000001111111111
111111111111111111111111111111111111000111111111111111111000111111
111111111111111111111111111111000000000000000111111111111111000000
000111111111111111111111000000000000000000000000000000000000000000
000000000000000000000000000000000111111111111111111111111111111111
111000000000000111111111111111111111000111111111111111111111111111
>tr|D2JWZ8|D2JWZ8_9FLAV Polyprotein OS=Dengue virus 3 PE=3 SV=1
MRCVGVGNRDFVEGLSGATWVDVVLEHGGCVTTMAKNKPTLDIELQKTEATQLATLRKLCIEGKIT
NITTDSRCPTQGEAVLPEEQDQNYVCKHTYVDRGWGNGCGLFGKGSLVTCAKFQCLEPIEGKVVQY
ENLKYTVIITVHTGDQHQVGNETQGVTAEITPQASTTEAILPEYGTLGLECSPRTGLDFNEMILLT
MKNKAWMVHRQWFFDLPLPWTSGATTETPTWNRKELLVTFKNAHAKKQEVVVLGSQEGAMHTALTG
ATEIQNSGGTSIFAGHLKCRLKMDKLELKGMSYAMCTNTFVLKKEVSETQHGTILIKVEYKGEDVP
CKIPFSTEDGQGKAHNGRLITANPVVTKKEEPVNIEAEPPFGESNIVIGIGDNALKINWYKKGSSI
GKMFEATARGARRMAILGDTAWDFGSVGGVLNSLGKMVHQIFGSAYTALFSGVSWVMKIGIGVLLT
WIGLNSKNTSMSFSCIAIGIITLYLGAVVQA
000000000000000000000000000000000000000000000000001111111111111110
000011111111111111111111000000000000000000000000000000000000000011
111111111111111111111111111111111111110000000000000000000000000111
111111111111000000000000000000000000000000000000111111111111110000
000000000000000000000000000000000000000000000000000000000000000000
000000000000000000000000000000000000000000000000000000000000000000
000000000000000000000000000000000000000000000000000000000000000000

0000000000000000000000000000000
>tr|Q53246|Q53246_ORITS Rickettsia tsutsugamushi (clone Pkt5) 47 kDa protein OS=Orientia tsutsugamushi PE=4 SV=1
MKKAFYLHLIVFALQGISNVHSKSLLNQKAVLPQQKSDMHINVNSLSDIVEPLISTVVSIYAVDTN
IGISLNNKVSKYQQEVFLGSGVIIDSSGYIVTNENVIAGRENIKVKLHDGSELIAELVGSDNKINI
ALLKINSSAALSYATFGDSNQSRVGDQVIAIGSPFGLRGTVTNGIISSKGRDMGNGIVTDFIQTNA
AIHMGSFGGPMFNLEGKIIGINSIHVSYSGISFAIPSNTVLEAVECLKKGEKIRRGMLNVMLNELT
PELNENLGLKKDQNGVLITEVIKEGSAAQCGIAPGDVITKFHDKEIKTGRDLQVAVSSTMLNSERE
VELLRNGKSMTLKCKIIANKGEDSEQQSNDQSLVVNGVKFIDLTPDLVKKYNITSANNNGLFVLEV
SPNSSWGRYGLKMGLRPRDIILSVKRDDNKKDISVKTLREIVTNIKHNEIFFTVQRGDRMLYIALP
NINK
000000000000000000001111111111111111111111000000000000000000000011
111111111111111100000000000000111111111100000000000000000000000000
000000000000111111111100000000000000000011111111111111000000111111
111111111111000000000000000000000011111111110011111111110000000000
000000000000000000000000000000000000000000000000000000000000000000
001111111111111111110000001111111111000000111111111111110000000000
000000000000000000000000000000000000000000000000000000000000000000
0000
>tr|A8BQ01|A8BQ01_9PAPI L1 protein OS=Human papillomavirus type 2 GN=L1 PE=4 SV=1
MSCGLNDVNVSTISLQMALWRPNESKVYLPPTPVSKVISTDVYVTRTNVYYHGGSSRLLTVGHPYY
SIKKSNNKVAVPKVSGYQYRVFHVKLPDPNKFGLPDADLYDPDTQRLLWACVGVEVGRGQPLGVGV
SGHPYYNRLDDTENAHTPDTADDGRENISMDYKQTQLFILGCKPPIGEHWSKGTTCNGSSAAGDCP
PLQFTNTTIEDGDMVETGFGALDFATLQSNKSDVPLDICTNTCKYPDYLKMAAEPYGDSMFFSLRR
EQMFTRHFFNRGGKMGDTIPDELYIKSTSVPTPGSHVYTSTPSGSMVSSEQQLFNKPYWLRRAQGH
NNGMCWGNRVFLTVVDTTRSTNVSLCATEASDTNYKATNFKEYLRHMEEYDLQFIFQLCKITLTPE
IMAYIHNMDPQLLEDWNFGVPPPPSASLQDTYRYLQSQAITCQKPTPPKTPTDPYASLTFWDVDLS
ESFSMDLDQFPLGRKFLLQRGAMPTVSRKRAAVSGTPPPTSKRKRVRR
000000000000000000000000000000000000000011111111111111111111111111
111111111111110000000001111111111111111111111111111111111111111100
000000000000000000000000000000000000000000000000000000000000000000
000000111111111110000000000000000000111111111111111111111111111111
111111111111111111111000000000000000000000000000000000000000000000
000000000000000000011111111110000000000111111111111111111111111111
111100000000000000000000000000000000000000000000000001111111111111
111111110000000000000000000000000000000000000000
>sp|P13423|PAG_BACAN Protective antigen OS=Bacillus anthracis GN=pagA

PE=1 SV=2

IATYNFENGRVRVDTGSNWSEVLPQIQETTARIIFNGKDLNLVERRIAAVNPSDPLETTKPDMTLK
EALKIAFGFNEPNGNLQYQGKDITEFDFNFDQQTSQNIKNQLAELNATNIYTVLDKIKLNAKMNIL
IRDKRFHYDRNNIAVGADESVVKEAHREVINSSTEGLLLNIDKDIRKILSGYIVEIEDTEGLKEVI
NDRYDMLNISSLRQDGKTFIDFKKYNDKLPLYISNPNYKVNVYAVTKENT
000000000000000000000000000000000000000000001111111111111111111100
000000000000000000000000000000000000000000111111111110000000000000
111111111111111111110000000000000000000000000000000000000000000000
00000011111111111100000000000011111111111111111111
