# Supplementary material for: Prediction of B-cell epitopes using evolutionary information and propensity scales
Source: BMC Bioinformatics. 2013 Jan 21;14(Suppl 2):S10. doi: 10.1186/1471-2105-14-S2-S10 (PMC3549808; doi:10.1186/1471-2105-14-S2-S10)
Supplement: Additional file 7 — The Benchmark dataset. [file 1471-2105-14-S2-S10-S7.pdf]

Additional file 7. Benchmark dataset.

>1BGX_T
MRGMLPLFEPKGRVLLVDGHHLAYRTFHALKGLTTSRGEPVQAVYGFAKSLLKALKEDGDAVIVVF
DAKAPSFRHEAYGGYKAGRAPTPEDFPRQLALIKELVDLLGLARLEVPGYEADDVLASLAKKAEKE
GYEVRILTADKDLYQLLSDRIHVLHPEGYLITPAWLWEKYGLRPDQWADYRALTGDESDNLPGVKG
IGEKTARKLLEEWGSLEALLKNLDRLKPAIREKILAHMDDLKLSWDLAKVRTDLPLEVDFAKRREP
DRERLRAFLERLEFGSLLHEFGLLESPKALEEAPWPPPEGAFVGFVLSRKEPMWADLLALAAARGG
RVHRAPEPYKALRDLKEARGLLAKDLSVLALREGLGLPPGDDPMLLAYLLDPSNTTPEGVARRYGG
EWTEEAGERAALSERLFANLWGRLEGEERLLWLYREVERPLSAVLAHMEATGVRLDVAYLRALSLE
VAEEIARLEAEVFRLAGHPFNLNSRDQLERVLFDELGLPAIGKTEKTGKRSTSAAVLEALREAHPI
VEKILQYRELTKLKSTYIDPLPDLIHPRTGRLHTRFNQTATATGRLSSSDPNLQNIPVRTPLGQRI
RRAFIAEEGWLLVALDYSQIELRVLAHLSGDENLIRVFQEGRDIHTETASWMFGVPREAVDPLMRR
AAKTINFGVLYGMSAHRLSQELAIPYEEAQAFIERYFQSFPKVRAWIEKTLEEGRRRGYVETLFGR
RRYVPDLEARVKSVREAAERMAFNMPVQGTAADLMKLAMVKLFPRLEEMGARMLLQVHDELVLEAA
EAVARLAKEVMEGVYPLAVPLEVEVGIGEDWLSAKE
000000000000000000000000000000000000000000000000000000000000000000
000000000000000000000000000000000000000000000000000000000000000000
000000000000000000000000000000000000000000000000000000000000000000
000000000000000000000000000000000000000000000000000000000000000000
000000000000000000000000000000000000000000000000111000000000000000
000000000000000000000100000000000000000000000000000110110100010000
111100000000000000000000000000000000000000000000000000000000000100
010010001000100000111111000000000000000000011000110011000000000000
000100110010111111110000000000000000100111000000010100100000000000
000000000000000000000000000000000000000000000000000000000000000000
000000000000000000000000000000000000000000000000000000000000000000
010001000000000000000000000000000000000000000000000000000000000000
000000000000000000000000000000000000
>1DEE_H
FNKDQQSAFYEILNMPNLNEAQRNGFIQSLKDDPSQSTNVLGEAKKLNESQAPK
000000000000000000000100110110011001001100100010000000
>1E6J_P
VHQAISPRTLNAWVKVVEEKAFSPEVIPMFSALSEGATPQDLNTMLNTVGGHQAAMQMLKETINEE
AAEWDRVHPVHAGPIAPGQMREPRGSDIAGTTSTLQEQIGWMTNNPPIPVGEIYKRWIILGLNKIV
RMYSPTSILDIRQGPKEPFRDYVDRFYKTLRAEQASQEVKNWMTETLLVQNANPDCKTILKALGPA
ATLEEMMTACQ
000000000000000000000000000000000000000000000000000000000000000000

000000000000000000000000000000000000000000000000000000000000000000
000000000000000000000000000000000000000000001000000000000000011111
11011001100
>1EGJ_A
IQMAPPSLNVTKDGDSYSLRWETMKMRYEHIDHTFEIQYRKDTATWKDSKTETLQNAHSMALPALE
PSTRYWARVRVRTSRTGYNGIWSEWSEARSWDTES
000000000000000000000000111111000000000000000000000000000100000000
00000000000010110100000000000000000
>1FJ1_F
SLDEKNSVSVDLPGEMKVLVSKEKNKDGKYDLIATVDKLELKGTSDKNNGSGVLEGVKADKCKVKL
TISDDLGQTTLEVFKEDGKTLVSKKVTSKDKSSTEEKFNEKGEVSEKIITRADGTRLEYTGIKSDG
SGKAKEVLKGYVLEGTLTAEKTTLVVKEGTVTLSKNISKSGEVSVELNDTDSSAATKKTAAWNSGT
STLTITVNSKKTKDLVFTKENTITVQQYDSNGTKLEGSAVEITKLDEIKNALK
000000000000000000000000000000000000000000000000000000000000000000
000000000000000000000000000000000000000000000000000000000000000000
000000000000000000000000000000000000000000000000000111101011100000
00000101110000000000000000001111100000000000000000000
>1FSK_A
GVFNYETETTSVIPAARLFKAFILDGDNLFPKVAPQAISSVENIEGNGGPGTIKKISFPEGLPFKY
VKDRVDEVDHTNFKYNYSVIEGGPIGDTLEKISNEIKIVATPDGGSILKISNKYHTKGDHEVKAEQ
VKASKEMGETLLRAVESYLLAHSDAYN
000000000000000000000000000000000000000001111111111110000000000000
000101000100000000001000000000100000000000000000000000000000000000
000000000000000000000000000
>1H0D_C
XDNSRYTHFLTQHYDAKPQGRDDRYEESIMRRRGLTSPFKDINTFIHGNKRSIKAIGENKNGNPHR
ENLRISKSSFQVTTEKLHGGSPWPPFQYRATAGFRNVVVAGENGLPVHLDQSIFRRP
000000000000000000000000000000000111111100000000000000000000000000
000000000000000010111111101000000000000000000000000000000
>1I9R_A
NPQIAAHVISEASSKTTSVLQWAEKGYYTMSNNLVTLENGKQLTVKRQGLYYIYAQVTFASNREAS
SQAPFIASLCLKSPGRFERILLRAANTHSSAKPAGQQSIHLGGVFELQPGASVFVNVTDPSQVSHG
TGFTSFGLLKL
000000000011010000000011110100000000000000000000000000000001100000
000000000000000000000000000000001100000000000000000000000000110111
10000000000
>1IGC_A
AVTTYKLVINGKTLKGETTTKAVDAETAEKAFKQYANDNGVDGVWTYDDATKTFTVTE

000000000011111111100000000000000101111000000000000000000
>1IQD_C
ESMPLGMESKAISDAQITASSYFTNMFATWSPSKARLHLQGRSNAWRPQVNNPKEWLQVDFQKTMK
VTGVTTQGVKSLLTSMYVKEFLISSSQDGHQWTLFFQNGKVKVFQGNQDSFTPVVNCLDPPLLTRY
LRIHPQSWVHQIALRMEVLGEEAQ
00000000000000000000011111000000000000001000010110000000000000000
00000000001111010000000000000000000000000000000000000000000000000
000000000110000000000000
>1JHL_A
KVYGRCELAAAMKRMGLDNYRGYSLGNWVDAAKFESNFNTGATNRNTDGSTDYGILQINSRWWEND
GRTPGSKNLFHIPESALLSSDITASVNFAKKIVSDGDGMNAWVAWRKHDKGTDVNVWIRGCRL
00000000000000000000101000000000000000000000000000000000000000000
000000000000000000000000000000000001001000000110011110100000000
>1JRH_I
SVPTPTNVTIESYNMNPIVYWEYQIMPQVPVFTVEVKNYGVKNSEWIDAEINISHHYENISDHVGD
PSNSLWVRVKARVGQKESAYAKSEEFAVS
00000000000000000000000000000000000101111111100000000000000000001
01100101000000000000010000000
>1KEN_A
STATLALGHHAVPNGTLVKTITDDQIEVTNATELVQSSSTGKIBNNPHRILDGIDCTLIDALLGDP
HCDVFQNETWDLFVERSKAFSNDYPYDVPDYASLRSLVASSGTLEFITEGFTWTGVTQNGGSNADK
RGPGSGFFSRLNWLTKSGSTYPVLNVTMPNNDNFDKLYIWGIHHPSTNQEQTSLYVQASGRVTVST
RRSQQTIIPNIGSRPWVRGLSSRISIYWTIVKPGDVLVINSNGNLIAPRGYFKMRTGKSSIMRSDA
PIDTBISEEITPNGSIPNDKPFQNVNKITYGAEPKYVKQNTLKLATGMRNVPEKQT
00000000000000000000000000000000000000000000000000000000000000000
00000000000000000000000000000000000000000000000000000000000111000
00000000000000010100000000000000000000000000110110111000000000000
00000000000000010011100000000000000000000000000000000000000000000
00000000000000000000000000000000000000000000000000000000
>1LK3_A
NMLRDLRDAFSRVKTFFQMKDQLDNLLLKESLLEDFKGYLGAQALSEMIQFYLEEVMPQAENQDPD
IKAHVNSLGENLKTLRLRLRRCHRFLPAEKSKAVEQVKNAFNKLQEKGIYKAMSEFDIFINYIEAY
MTMK
00000000000000000010000000000000000000000000000001001100110011000
00000000000000000000000000000000000010011111110110000000000000000
0000
>1MHP_B
TQLDIVIVLDGSNSIYPWESVIAFLNDLLKRMDIGPKQTQVGIVQYGENVTHEFNLNKYSSTEEVL

VAANKIVQRGGRQTMTALGIDTARKEAFTEARGARRGVKKVMVIVTDGESHDNYRLKQVIQDCEDE
NIQRFSIAILGHYNRGNLSTEKFVEEIKSIASEPTEKHFFNVSDELALVTIVKALGERIF
000000000001110100000000000000000000000000000000001000000000000000000
000000011111110000000000000000000000000000000000010101100000000000000
000000000000101001000000000000000000000000000000000000000000000
>1N8Z_C
TQVETGTDMKLRLPASPETHLDMLRHLYQGEQVVQGNLELTYLPTNASLSFLQDIQEVQGYVLIAH
NQVRQVPLQRLRIVRGTQLFEDNYALAVLDNGDPLSPGGLRELQLRSLTEILKGGVLIQRNPQLFY
QDTILWKDIFHKNNQLALTLIDTNRSRAFHPGSPMHKGSRGWGESSEDHQSLTRTVIAGGJARIKG
PLPTDJKHEQLAAGKTGPKHSDLLAMLHFNHSGIMELHNPALVTYNTDTFESMPNPEGRYTFGASN
VTAOPYNYLSTDVGSOTLVPPLHNQEVTATQRPEKQSKPQARVRYGLGMEHLREVRAVTSANIQEF
AGRKKIFGSLAFLPESFDSNTAPLQPEQLQVFETLEEITGYLYISAWPDSLPDLSVFQNLQVIRGR
ILHNGAYSLTLQGLGISWLGLRSLRELGSGLALIHHNTHLSFVHTVPWDQLFRNPHQALLHTANRP
EDESVGEGLATHQLUARGHTWGPGPTQUVNVSQFLRGQEVVEEWRVLQGLPREYVNARHWLPXHPE
YQPQNGSVTXFGPEADQYVAZAHYKDPPFZVARAPSIWKFPDEEGAAQPCPIN
000000000000000000000000000000000000000000000000000000000000000000000
000000000000000000000000000000000000000000000000000000000000000000000
000000000000000000000000000000000000000000000000000000000000000000000
000000000000000000000000000000000000000000000000000000000000000000000
000000000000000000000000000000000000000000000000000000000000000000000
000000000000000000000000000000000000000000000000000000000000000000000
000000000000000000000000000000000000000000000000000000000000000000000
000000000000000000000000000000000000000000000000000000000000000000000
00000000000011011000000001111100000000101001010101110000
>1NL0_G
YNSGKLXXFVQGNLXRXEMXXKESFXXARXVFXNTXRTTXFWKQY
0000110111110000000000000000000001000000000000000
>1NSN_S
TKKLHKEPATLIKAIDGDTVKLMYKGQPMTFRLLLVDTPETKHPKKGVEKYGPEASAFTKKMVENA
KKIEVEFDKGQRTDKYGRGLAYIYADGKMVNEALVRQGLAKVAYVYKPNNTHEQHLRKSEAQAKKE
KLNIWS
000001000000000000000000000000000000000000000000000000001001100100010
100000000000000000000000000111100000001100000000000000011001001000000001
000000
>1OAZ_A
SDKIIHLTDDSFDTDVLKADGAILVDFWAEWCGPIEESDDRRYDLVGPCKMIAPILDEILTVAKLN
IDQNPGTAPKYGIRGIPTLLLFKNGEVAATKVGALSKGQLKEFLDANLA
000000000000000000000000000000001110100010100111000000000000000000000

0000000000000111000000000000000111100100000000000
>1ORQ_C
IGDVMEHPLVELGVSYAALLSVIVVVVECTMQLSGEYLVRLYLVDLILVIILWADYAYRAYKSGDP
AGYVKKTLYEIPALVPAGLLALIEGHLAGLGLFRLVRLLRFLRILLIISRGSKFLSAIADAADKIR
FYHLFGAVMLTVLYGAFAIYIVEYPDPNSSIKSVFDALWWAVVTATTVGYGDVVPATPIGKVIGIA
VMLTGISALTLLIGTVSNMFQKILV
000000000000000000000000000000000000110011000000000000000000000000
000000000000000000000001000111111101000100000000000000000000000000
000000000000000000000000000000000000000000000000000000000000000000
0000000000000000000000000
>1PKQ_E
QFRVIGPGHPIRALVGDEAELPERISPGKNATGMEVGWYRSPFSRVVHLYRNGKDQDAEQAPEYRG
RTELLKESIGEGKVALRIQNVRFSDEGGYTEFFRDHSYQEEAAVELKVEDPF
100000000000000000000000000010011000001000010000000110000000000000
0000000000000000000000000000000001111111100000000000
>1WEJ_F
GDVEKGKKIFVQKCAQCHTVEKGGKHKTGPNLHGLFGRKTGQAPGFTYTDANKNKGITWKEETLME
YLENPKKYIPGTKMIFAGIKKKTEREDLIAYLKKATNE
000000000000000000000000000000000001100000000000000000000101110000
00000000000000000000000000000100110011
>1YJD_C
NKILVKQSPMLVAYDNAVNLSEKYSYNLFSREFRASLHKGLDSAVEVFVVYGNYSQQLQVYSKTGF
NFDGKLGNESVTFYLQNLYVNQTDIYFEKIEVMYPPPYLDNEKSNGTIIHVK
000000000000000000000000000000010100000000000001010000000010111110
0000000000000000000000000000001011000100000000000000
>1YNT_F
PLVANQVVTIPDKKSTAAVILTPTENHFTLKJPKTALTEPPTLAYSPNRQIKPAGTTSSKTSKAVT
LSSLIPEAEDSWWTGDSASLDTAGIKLTVPIEKFPVTTQTFVVGJIKGDDAQSIMVTVTVQARASS
VVNNVARLSYGADSTLGPVKLSAEGPTTMTLVMGKDGVKVPQDNNQYNSGTTLTGNNEKSFKDILP
KLTENPWQGNASSDKGATLTIKKEAFPAESKSVIIGMTGGSPEKHHLTVKLEFA
000000000000000000000000000000000011101011001001010000000001111111
000000000000000000000000000000000000000000000111000000000000000000
000000000000000000000000000000000000000000000000000000000000000000
000000000000000000000000000000000000000000000000000000
>1YY9_A
EEKKVAQGTSNKLTQLGTFEDHFLSLQRMFNNAEVVLGNLEITYVQRNYDLSFLKTIQEVAGYVLI
ALNTVERIPLENLQIIRGNMYYENSYALAVLSNYDANKTGLKELPMRNLQEILHGAVRFSNNPALB
NVESIQWRDIVSSDFLSNMSMDFQNHLGSBQKCDPSDPNGSCWGAGEENDQKLTKIIEAQQFSGRE

RGKSPSDFGHNQHAAGGTGPRESDHLVIRKFRDEATIKDTJPPLMLYNPTTYQMDVNPEGKYSFGA
TJVKKKPRNYVVTDHGSKVRALGADSYEMEEDGVRKLKKMEGPMRKVNNGIGIGEFKDSLSINATN
IKHFKNNTSISGDLHILPVAFRGDSFTHTPPLDPQELDILKTVKEITGFLLIQAWPENRTDLHAFE
NLEIIRGRTKQHGQFSLAVVSLNITSLGLRSLKEISDGDVIISGNKNLOYANTINWKKLFGTSGQK
TKIISNRGENKOKATGQVPHALQSPEGPWGPEPRDQVSRRNVSRGRERVDKSKLLEGEPREFVENS
ESIQTHPEULPQAMNITTTGRGPDNUIQVAHYIDGPHVVKTWPAGVMGENNTLVWKYADAGHVWHL
XHPNYTYGXTGPGLRGYPT
000000000000000000000000000000000000000000000000000000000000000000
000000000000000000000000000000000000000000000000000000000000000000
000000000000000000000000000000000000000000000000000000000000000000
000000000000000000000000000000000000000000000000000000000000000000
000000000000000000000000000000000000000000000000000000000000000000
000000000000000001000100000000000000000000000000001010000000000000
000000000011011000011000000000000000000101101000000000000000000000
010111010100000000000000000000000000000000000000000000000000000000
000000000000000000000000000000000000000000000000000000000000000000
0000000000000000000
>1ZA3_R
SSPSEGLEPPGHHISEDGRDEISFKYGQDYSTHWNDLLFFLRGTRHDSGEVELSPGTTTRNTVHQI
EEGTFREEDSPEMIRKJDJTPWSDI
000011100000010111000000000000001101111011000000000000000000000000
0000000000000000000000000
>1ZTX_E
TTYGVASKAFKFLGTPADTGHGTVVLELQYTGTDGPAKVPISSVASLNDLTPVGRLVTVNPFVSVA
TANAKVLIELEPPFGDSYIVVGRGEQQINHHWHKS
001000111100000000000000000001110000000000000000000000000000000001
11100000000000000000000111000000000
>2JEL_P
MFQQEVTITAPNGLHTRPAAQFVKEAKGFTSEITVTSNGKSASAKSLFKLQTLGLTQGTVVTISAE
GEDEQKAVEHLVKLMAELE
111100000000000000000000000000000100000001000000000000000000000101
1101110011000000000
>1UJ3_C
TVAAYNLTWKSTNFKTILEWEPKPVNQVYTVQISTKSGDWKSKFFYTTDTEFDLTDEIVKDVKQTY
LARVFSYPAGNVESTGSAGEPLYENSPEFTPYLETNLGQPTIQSFEQVGTKVNVTVEDERTLVRRN
NTFLSLRDVFGKDLIYTLYYWKSSSSGKKTAKTNTNEFLIDVDKGENYGFSVQAVIPSRTVNRKST
DSPVEGM
000000000000000000000000000000000000000000000000000000000000000000

000000000000000000000000000000000000000000000000001000000000000000
000000000001001010100000000111110100000000000000000010101011011100
1000000
>1AR1_B
QDVLGDLPVIGKPVNGGMNFQPASSPLAHDQQWLDHFVLYIITAVTIFVCLLLLICIVRFNRRANP
VPARFTHNTPIEVIWTLVPVLILVAIGAFSLPILFRSQEMPNDPDLVIKAIGHQWYWSYEYPNDGV
AFDALMLEKEALADAGYSEDEYLLATDNPVVVPVGKKVLVQVTATDVIHAWTIPAFAVKQDAVPGR
IAQLWFSVDQEGVYFGQCSELCGINHAYMPIVVKAVSQEKYEAWLAGAKEEFAA
000000000000000000000000110011000000000000000000000000000000000000
000000000000000000000000000000000000000000000000000000000000000000
000000000000000000000000000000000111000000000000000000000000000000
000000111110000000000000000000000000111001000000000000
>1BJ1_V
VVKFMDVYQRSYCHPIETLVDIFQEYPDEIEYIFKPSCVPLMRDGGCENDEGLECVPTEESNITMQ
IMRIKPHQGQHIGEMSFLQHNKDEERPKVVKFMDVYQRSYCHPIETLVDIFQEYPDEIEYIFKPSC
VPLMRDGGCENDEGLECVPTEESNITMQIMRIKPHQGQHIGEMSFLQHNKDEERPK
000100010000000000000000000000010000000000000000000000000000000000
111000111111110000000000000000010001000000000000000000000001000000
00000000000000000000000000001110001111111100000000000000
>1EZV_E
KSTYRTPNFDDVLKENNDADKGRSYAYFMVGAMGLLSSAGAKSTVETFISSMTATADVLAMAKVEV
NLAAIPLGKNVVVKWQGKPVFIRHRTPHEIQEANSVDMSALKDPQTDADRVKDPQWLIMLGICTHL
GAVPIGEAGDFGGWFCPAHGSHYDISGRIRKGPAPLNLEIPAYEFDGDKVIVG
000000000000000000000000000000000000000000000000000000000000000000
000000000000000000000000001101100111110000000111001111100000000000
00000000000000000000000000000000000000000000000000000
>1FE8_C
APDKSQPLDVILLLDGSSSFPASYFDEXKSFAKAFISKANIGPRLTQVSVLQYGSITTIDVPWNVV
PEKAHLLSLVDVXQREGGPSQIGDALGFAVRYLTSEXHGARPGASKAVVILVTDVSVDSVDAAADA
ARSNRVTVFPIGIGDRYDAAQLRILAGPAGDSNVVKLQRIEDLPTXVTLGNSFLHKLKS
000000000000000000000000000000000000000000110010000000000000011111
110110011011000000000000000001110100000000000000000000000000000000
00000000000000000000000000000000000000000000000000000000000
>1OAK_A
MYESRLLDLVFLLDGSSRLSEAEFEVLKAFVVDMMERLRISQKWVRVAVVEYHDGSHAYIGLKDRK
RPSELRRIASQVKYAGSQVASTSEVLKYTLFQIFSKIDRPEASRIALLLMASQEPQRMSRNFVRYV
QGLKKKKVIVIPVGIGPHANLKQIRLIEKQAPENKAFVLSSVDELEQQRDEIVSYLEDLAPEAP
000000000000000000000000000000000000000000000000000000000000000000

0000000000000000000000000000000000000000000000000000000111011111100
0000000000000000000101100100000000000000000000000000000000000000
>2B4C_G
VVLENVTEHFNMWKNDMVEQMQEDIISLWDQSLKPAVKLTPLBVGAGSBDTSVITQAAPKISFEPI
PIHYCAPAGFAILKDNDKTFNGKGPDKNVSTVQCTHGIRPVVSTQLLLNGSLAEEEVVIRSDNFTN
NAKTIIVQLKESVEINETRPNQNTRKSIHIGPGRAFYTTGEIGDIRQAHENISRAKWNDTLKQIVI
KLREQFENKTIVFNHSSGGDPEIVMHSFNFGGEFFYGNSAQLFNSTWNNNTEGSNNTEGNTITLPG
RIKQIINMWQEVGKAMYAPPIRGQIRFSSNITGLLLTRDGGINENGTEIFRPGGGDMRDNWRSELY
KYKVVKIE
000000000000000000000000000000000001101000000000000010000000000000
000000000000000000000000000000000000000000000000000000000000000000
000000000000000000000000000000000000011011000100000000000000000000
000000000000000000000000000000000000000000000000000000000000000000
001110000000010111100000000000000000000000000000000000000000000000
00000000
>1J5O_B
PISPIETVPVKLKPGMDGPKVKQWPLTEEKIKALVEICTEMEKEGKISKIGPENPYNTPVFAIKKK
DSTKWRKLVDFRELNKRTQDFWEVQLGIPHPAGLKKKKSVTVLDVGDAYFSVPLDEDFRKYTAFTI
PSINNETPGIRYQYNVLPQGWKGSPAIFQSSMTKILEPFKKQNPDIVIYQYIDDLYVGSDLEIGQH
RTKIEELRQHLLRWGLTTPDKKHQKEPPFLWMGYELHPDKWTVQPIVLPEKDSWTVNDIQKLVGKL
NWASQIYPGIKVRQLSKLLRGTKALTEVIPLTEEAELELAENREILKEPVHGVYYDPSKDLIAEIQ
KQGQGQWTYQIYQEPFKNLKTGKYARMRGAHTNDVKQLTEAVQKITTESIVIWGKTPKFKLPIQKE
TWETWWTEYWQATWIPEWEFVNTPPLVKLWYQLE
000000000000000000000000000000000000000000000000000000000000000000
000000000000000000000000000000000000000000000000000000000000000000
000000000000000000000000000000000000000000000000000000000000000000
100000000000000000000000111110111000000000000000000000000000000000
000000000000000000000000000000000000000000000000000000000000000000
000000000000000000000000000000000000000000000000000000000000000000
0000000000000000000000000000000000
>1R3K_C
SALHWRAAGAATVLLVIVLLAGSYLAVLAERGAPGAQLITYPRALWWSVETATTVGYGDLYPVTLW
GRCVAVVVMVAGITSFGLVTAALATWFVGREQERRGH
000000000000000000000001000100111111011111000000000000000000000000
0000000000000000000000000000000000000
>1NFD_D
DSGVVQSPRHIIKEKGGRSVLTIIPISGHSNVVWYQQTLGKELKFLIQHYEKVERDKGFLPSRFSV
QQFDDYHSEMNMSALELEDSAMYFIASSLRWGDEQYFGPGTRLTVEDLRNVTPPKVSLFEPSKAEI

ANKQKATLVJLARGFFPDHVELSWWVNGKEVHSGVSTDPQAYKESNYSYSLSSRLRVSATFWHNPR
NHFRJQVQFHGLSEEDKWPEGSPKPVTQNISAEAWGRADH
000000000000010000000000000000000000000000000000000000000000000000
000000000000000000000000000000000000000000000010110100000000000000
000000000000000000000000000000000000000000000000000000000000000000
0000000000000001111111000000000000000000
>1TQC_A
GLGGYMLGSAMSRPLIHFGNDYEDRYYRENMYRYPNQVYYRPVDRYSNQNNFVHDAVNITVKQHTV
TTTTKGENFTETDIKIMERVVEQMAITQYQRESQAY
110000000000000000000000010001011000000000000000000000000010011011
111111100000000000000000000000000000
>1V7M_V
EDLRVLSKLLRDSHVLHSRLSQFPEVHPLPTPVLLPAVDFSLGEWKTQMEETKAQDILGAVTLLLE
GVMAARGQLGPTFLSSLLGQLSGQVRLLLGALQSLLGTQLPPQGRTTAHKDPNAIFLSFQHLLRGK
VRFLMLVGGSTLE
000000000000000000000000000000000000000000000000001100100000010010
001000000000000000000000010001001100111111100000000000000000000000
0000000000000
>1XIW_A
QTPYKVSISGTTVILTAPQYPGSEILWQHNDKNIGGDEDDKNIGSDEDHLSLKEFSELEQSGYYVA
YPRGSKPEDANFYLYLRARVCENBMQTPYKVSISGTTVILTAPQYPGSEILWQHNDKNIGGDEDDK
NIGSDEDHLSLKEFSELEQSGYYVAYPRGSKPEDANFYLYLRARVCENBM
000000000000000000000001000000001111110000001100000000000000000000
101111111000000000000000000000000000000000000000100000000111111000
000110000000000000000000001011111110000000000000000
>1XIW_B
MKIPIEELEDRVFVNCNTSITWVEGTVGTLLSDITRLDLGKRILDPRGIYRCNESTVQVHYRMBQS
MKIPIEELEDRVFVNCNTSITWVEGTVGTLLSDITRLDLGKRILDPRGIYRCNESTVQVHYRMBQS
000001011110100000000000000000001101000000000000000000000000000000
000001011110100000000000000000001101000000000000000000000000000000
>1Z3G_A
AVTVDTIIKNGQLVQMSNHFKIMJNEGLVHLSENTJEEKNEKKKETLGKALGEFGQKIENPDPAQV
NMYKLGMIEGYTLKEDTMVLDVNQYKNOGESGENIVEYLSEIQSAGOSPAIGKVPNPEDEKKPTKT
GETAQQLKRNTDNEVQKNVEGVYKRQCMEGFTFDKEKNVCL
000000000000000000000000000000000000000000010011000000011111111011
111001101000000000000000000000000000000000000000000000000000000000
00000000000000000000000000000000000000000
>2AEP_A

AEYRNWSKPQAKITGFAPFSKDNSIRLSAGGDIWVTREPYVSBDPDKBYQFALGQGTTLNNRHSND
TVHDRTPYRTLLMNELGVPFHLGTKQVCIAWSSSSDHDGKAWLHVCVTGHDENATASFIYDGRLVD
SIGSWSKKILRTQESEDVEINGTETVVMTDGSASGRADTKILFIEEGKIVHISPLSGSAQHVEEFS
GYPRYPGVRGVFRDNWKGSNRPIVDINVKDYSIVSSYVHSGLVGDTPRKNDSSSSSHHLNPNNEEG
GHGVKGWAFDDGNDVWMGRTISEKFRSGYETFKVIEGWSKPNSKLQINRQVIVDRGNRSGYSGIFS
VEGKSAINRIFYVELIRGRKQETEVWWTSNSIVVFIGTSGTYGTGSWPDGADINLMPI
000000000000000000000000000000000000000000000000000000000000000001
001011100000000000000000000000000000111000000000000000000000000000
000011111010000000000000000000110111100000000000000000000000000000
000000000000000000000000000000000000000000000000000000000000000000
000000000000000000000000000000000000000000000000000000000000000000
0000000000000000000000000000000000000000000000000000000000
>1IAI_I
EVKLQESGGGLVQPGGSMKLSHVASGFTFNNYWMSWVRQSPEKGLEWVAEIRLNSDNFATHYAESV
KGKFIISRDDSKSRLYLQMNSLRAEDTGIYYHVLRPLFYYAVDYWGQGTSVTVSSAKTTPPSVYPL
APGSAAQTNSMVTLGILVKGYFPEPVTVTWNSGSLSSGVHTFPAVLQSDLYTLSSSVTVPSTWRPS
ETVTINVAHPASSTKVDKKI
100000000000000000000000000011100000000000000000000101100000000000
000000000000000000000000000000000000111100000000000000000000000000
000000000000000000000000000000000000000000000000000000000000000000
00000000000000000000
>1IAI_M
DIQLTQSPAFMAASPGEKVTITFSVSSSISSSNLHWYQQKSETSPKPWIYGTSNLASGVPVRFSGS
GSGTSYSLTISSMEAEDAATYYFQQWNSYPYTFGGGTKLEIKRADAAPTVSIFPPSSEQLTSGGAS
VVGFLNNFYPKDINVKWKIDGSERQNGVLNSWTDQDSKDSTYSMSSTLTLTKDEYERHNSYTGEAT
HKTSTSPIVKSFNRNEC
000000000000000000000000001111000000000000000000000000000000000000
000000000000000000000000001000000000000000000000000000000000000000
000000000000000000000000000000000000000000000000000000000000000000
00000000000000000
>1KB5_A
QQVRQSPQSLTVWEGETAILNASYEDSTFNYFPWYQQFPGEGPALLISIRSVSDKKEDGRFTIFFN
KREKKLSLHITDSQPGDSATYFAAARYQGGRALIFGTGTTVSVSPGSAD
110000000000000000000000010000000000000000000000000000000000000000
0000000000000000000000000011111100000000000000000
>1KB5_B
VTLLEQNPRWRLVPRGQAVNLRBILKNSQYWMSWYQQDLQKQLQWLFTLRSPGDKEVKSLPGADYL
ATRVTDTELRLQVANMSQGRTLYBTCSAAPDWGASAELYFGSGTRLTV

000000000000000000000000000000100000000001011000011001010111100000
000000000000000000000000000001010000000000000000
>1QFW_A
DAPEBTLQENPFFSQPGAPILQCMGADFSRAYPTPLRSKKTMLVQKNVTSESTEBVAKSYNRVTVM
GGFKVENHTACHDSTEYYHKS
000000001011111100000000000000000000001000001010000000000000000001
000000000000000000000
>1QFW_B
KEPLRPRFRPINATLAVEKEGGPVHITVNTTIIAGYJPTMTRVLQGVLPALPQVVFNYRDVRFESI
RLPGGPRGVNPVVSYAVALSIQJALKRRSTTDKGGPKDHPLTHD
0000000000000000001000000000000000000000001011000000000000000001010
10000111111111000000000000000000000000000000
>1OB1_F
NISQHQQVKKQRPQNSGQFRHLDEREERKSLLNYKQEGDKSVENPNPTTNENNGGUDADAKTTEED
SGSNGKKITUEVTKPDSYPLFDGIFVSHHH
000000011110100000001011110100000000011000000000001100000110000000
000000000000010000000000000001
>1OTS_A
RRRQLIRQLLERDKTPLAILFMAAVVGTLVGLAAVAFDKGVAWLQNQRMGALVHTADNYPLLLTVA
FLCSAVLAMFGYFLVRKYAPEAGGSGIPEIEGALEDQRPVRWWRVLPVKFFGGLGTLGGGMVLGRE
GPTVQIGGNIGRMVLDIFRLKGDEARHTLLATGAAAGLAAAFNAPLAGILFIIEEMRPQFRYTLIS
IKAVFIGVIMSTIMYRIFNHEVALIDVGKLSDAPLNTLWLYLILGIIFGIFGPIFNKWVLGMQDLL
HRVHGGNITKWVLMGGAIGGLCGLLGFVAPATSGGGFNLIPIATAGNFSMGMLVFIFVARVITTLL
CFSSGAPGGIFAPMLALGTVLGTAFGMVAVELFPQYHLEAGTFAIAGMGALLAASIRAPLTGIILV
LEMTDNYQLILPMIITGLGATLLAQFTGGKPLYSAILARTLAKQEAEQ
000000000000000000000000000000000000000000000000000000000000000000
000000000000000000000000000000000000000000000000000000000000000000
000000000000000000000000000000000000000000000000000000000000000000
000000000000000010011000000010010111000000000000000000000000000000
000000000000000000000000000000000000000000000000000000000000000000
000000000000000000000000000000000111000000000000000000000000000000
000000000000000000000000000000000000000000000000
