# Supplementary material for: Prediction of B-cell epitopes using evolutionary information and propensity scales
Source: BMC Bioinformatics. 2013 Jan 21;14(Suppl 2):S10. doi: 10.1186/1471-2105-14-S2-S10 (PMC3549808; doi:10.1186/1471-2105-14-S2-S10)
Supplement: Additional file 8 — Supplementary tables on optimization of hybrid propensity scale method. [file 1471-2105-14-S2-S10-S8.pdf]

Additional file 8. The Sollner dataset.

Supplementary Table 1. AUC of single propensity method scales across different window size.

Window size	AA ratio	Hydrophilicity	Flexibility	Polarity	Accessible area	PSSM
**5**	0.5742	0.5417	0.5456	0.5282	0.5180	0.6366
**7**	0.5839	0.5545	0.5533	0.5372	0.5114	0.6470
**9**	0.5926	0.5593	0.5577	0.5353	0.5029	0.6549
**11**	0.5905	0.5655	0.5668	0.5353	0.4960	0.6624
**13**	0.5937	0.5723	0.5722	0.5210	0.4928	0.6688
**15**	0.6006	0.5792	0.5772	0.5197	0.4904	0.6740
**17**	0.6047	0.5845	0.5812	0.5256	0.4876	0.6774
**19**	0.6090	0.5855	0.5859	0.5442	0.4863	0.6786
**21**	0.6085	0.5876	0.5898	0.5533	0.4861	
**23**	0.6085	0.5903	0.5945	0.5613	0.4869	
**25**	0.6066	0.5912	0.5992	0.5651	0.4874	
**27**	0.6079	0.5977	0.6031	0.5693	0.4879	
**29**	0.6079	0.6033	0.6065	0.5736	0.4866	

Supplementary Table 2. AUC of hybrid propensity method scales across different window size.

window size	(1)	(2)	(3)	(4)
**5**	0.6277	0.6268	0.6070	0.6049
**7**	0.6528	0.6487	0.6281	0.6235
**9**	0.6671	0.6617	0.6414	0.6352
**11**	0.6738	0.6687	0.6494	0.6443
**13**	0.6822	0.6769	0.6582	0.6526
**15**	0.6917	0.6861	0.6697	0.6637
**17**	0.6980	0.6924	0.6779	0.6724
**19**	0.7049	0.7003	0.6863	0.6818
**21**	0.7097	0.7067	0.6921	0.6891
**23**	0.7142	0.7107	0.6974	0.6948
**25**	0.7184	0.7150	0.7022	0.6994
**27**	0.7219	0.7189	0.7060	0.7035
**29**	0.7243	0.7213	0.7086	0.7060

(1) 18 propensity scales + PSSM + AA ratio scale

(2) 18 propensity scales + PSSM

(3) 18 propensity scales + AA ratio scale

(4) 18 propensity scales

Supplementary Table 3. Optimization of parameters. (a) *c, g* (b) w_{+1} (c) feature selection (d) linear weighting factor (e) re-optimization of w_{+1}

For the calculation of AA ratio scale, logarithm was neglected before normalization.

(a)

g\c	0.01	0.1	1	10	100
**0.01**	0.6152	0.6369	0.6574	0.6862	0.7201
**0.1**	0.6418	0.6822	0.7275	0.7848	0.8590
**1**	0.7252	0.8282	0.9243	0.9684	0.9797
**10**	0.9042	0.9760	0.9825	0.9812	0.9805
**100**		0.9621	0.9622	0.9627	0.9619

(b)

w_{+1}	10	11	12	13	14
**AUC**	0.9831	0.9831	0.9831	0.9830	0.9830

w_{+1}	15	16	17	18	19
**AUC**	0.9829	0.9827	0.9826	0.9826	0.9826

(c)

Remove No.	None	1	2	3	4	5	6
**AUC**	0.9831	0.9807	0.9826	0.9829	0.9827	0.9829	0.9833

Remove No.	7	8	9	10	11	12	13
**AUC**	0.9817	0.9810	0.9836	0.9827	0.9801	0.9814	0.9827

Remove No.	14	15	16	17	18	19	20
**AUC**	0.9814	0.9828	0.9828	0.9841	0.9840	0.9832	0.9723

Remove No.	6, 9 17 18, 19	6, 9, 17, 18	9, 17, 18, 19	9, 17, 18	6, 17, 18	17, 18, 19	17, 18
**AUC**	0.9843	0.9855	0.9850	0.9854	0.9850	0.9848	0.9849

(d)

f	0.00	0.02	0.04	0.06	0.08	0.10
**AUC**	0.9855	0.9907	0.9943	0.9961	0.9972	0.9966

(e)

w_{+1}	10	11	12	13	14
**AUC**	0.9971	0.9972	0.9972	0.9972	0.9972

w_{+1}	15	16	17	18	19
**AUC**	0.9973	0.9973	0.9972	0.9971	0.9971

Supplementary Table 4. Optimization of parameters. (a) *c, g* (b) w_{+1}

For the calculation of AA ratio scale, logarithm in base of the minimum ratio value was taken before normalization.

(a)

**g\c**	**0.01**	**0.1**	**1**	**10**	**100**
**0.01**	0.6152	0.6369	0.6574	0.6862	0.7201
**0.1**	0.6418	0.6822	0.7275	0.7848	0.8590
**1**	0.7252	0.8282	0.9243	0.9684	0.9797
**10**	0.9042	0.9760	0.9825	0.9812	0.9805
**100**		0.9621	0.9622	0.9627	0.9619

(b)

w_{+1}	**10**	**11**	**12**	**13**	**14**
**AUC**	0.9829	0.9829	0.9829	0.9829	0.9829

w_{+1}	**15**	**16**	**17**	**18**	**19**
**AUC**	0.9828	0.9826	0.9825	0.9825	0.9824
